# The transformation of 20-year social participation policies of older people in China: Network analysis and text analysis

**DOI:** 10.1371/journal.pone.0308401

**Published:** 2024-08-12

**Authors:** ZiQi Mei, WeiTong Li, JunYu Chen, HaiYan Yin, YuLei Song, WenJing Tu, ZiChun Ding, YaMei Bai, ShengJi Jin, Guihua Xu

**Affiliations:** School of Nursing, Nanjing University of Chinese Medicine, Nanjing, PR China; University of Kragujevac: Univerzitet u Kragujevcu, SERBIA

## Abstract

**Background:**

Social participation of older adults is a crucial component of China’s aged care services and an important strategy for actively addressing the aging population. Analyzing policy texts on older people’s social participation can inform future policy formulation and the development of relevant programs.

**Objectives:**

This study aims to quantitatively analyze the transformation of China’s social participation policies for older people from 1999 to 2023, employing institutional network analysis and policy text analysis.

**Method:**

A two-dimensional policy analysis framework was constructed based on the perspective of “policy tools and social participation stages.” Using Rost Content Mining 6.0 and Nvivo 11.0 Plus software, 55 national-level policy texts were coded. Structural analysis of policy-issuing subjects and topic words was conducted to visualize the findings.

**Results:**

The analysis revealed that the policy-issuing subjects demonstrated strong authority but weak coordination, with a lack of communication and cooperation across subjects. The use of policy tools was imbalanced, with an over-reliance on supply-type tools and insufficient use of demand-type tools. Additionally, the lack of effective policy tools to support various social participation stages has limited policy implementation.

**Conclusion:**

With technological advancement and changing needs of the elderly population, there is a need for a more systematic and forward-looking top-level design of elderly social participation policies: accelerating the systematization and precision of technological elements in policies for elderly social participation, integrating social organizations via technological platforms to mobilize diverse stakeholder engagement, and addressing the digital divide between the elderly and new technologies is imperative.

## Introduction

In the twenty-first century, the irreversible challenge of an aging population is becoming increasingly apparent. According to the United Nations World Population Prospects, China’s senior population will constitute more than 20% of the country’s total population by 2025, marking its transition into a moderately aging society [1]. As China’s population continues to age and life expectancy increases, actively addressing aging has reached a level of national strategic importance. The ability of older adults to adapt to societal changes is critical for implementing healthy aging. Social participation, as an essential component of healthy aging, has been receiving increasing attention [2]. It is widely recognized that individuals’ engagement in activities involving interactions with others, performing daily tasks, and assuming significant roles in society constitutes social participation [3]. The World Health Organization’s (WHO) policy on aging populations emphasizes enhancing social participation as a crucial recommendation [4]. Research has demonstrated that social participation can markedly reduce the risk of depression and improve the mental health of older adults [5, 6]. Engaging in leisure activities allows older individuals to build social relationships, fulfill life values, cultivate positive emotions, and enhance subjective well-being [7]. Social participation significantly benefits the health of older adults and can transform the adverse effects of aging on economic and social development into a driving force for long-term societal progress. Therefore, promoting social activities among older individuals is essential for achieving active and successful aging. The WHO has long emphasized the need to formulate policies that care for the elderly to promote social inclusion and participation among older adults [4].

China’s policies on social participation for the elderly have undergone significant evolution, progressing from steady development to rapid growth. This evolution can be broadly divided into five distinct stages [8]. Emergence Stage (1949–1982): This initial stage commenced with the establishment of the People’s Republic of China (PRC) and the introduction of the retirement system. During this period, opportunities for social participation were not universally available; they were primarily limited to senior officials, party members, and retired cadres within the system, thereby excluding the majority of older adults. Initial Stage (1983–1998): In this phase, the scope of social participation policies for the elderly gradually expanded to include elderly intellectuals and professional technical personnel. A landmark development was the enactment of the Law of the PRC on the Protection of the Rights and Interests of the Elderly in 1996. This legislation formally defined the rights of the elderly to social participation, providing essential legal support for their involvement in social development [9, 10]. Exploration Stage (1999–2005): Following the global launch of the "Active Aging Action" by the European Union and the World Health Organization in 1999, China initiated the " Silver Age Action Program" [10, 11]. This new initiative focused on mobilizing elderly intellectuals to provide assistance to western regions. During this period, China continuously improved the scope of participants and the content and forms of activities, thereby gradually enriching its social participation policies for the elderly. Establishment Stage (2006–2016): During this phase, China officially introduced chapters dedicated to explaining the concept and significance of "actively addressing population aging" and issued supportive policies from various government departments to encourage social participation among the elderly. Throughout this stage, China actively explored and practiced "active aging," with increasing protection of the rights of the elderly to participate in society [12]. Deepening and Maturity Stage (2017-present): In this stage, the strategic importance of "actively addressing population aging" has been emphasized, with "promoting social participation of the elderly" explicitly stated as a separate goal in planning. The content and forms of social participation for the elderly have become increasingly diverse, and policies are entering a phase of deepening. The evolution of these policies reflects a shift in focus from addressing the political needs of a few retired cadres to leveraging the potential of elderly scientists and intellectuals, promoting their limited participation in contributing to society, and finally ensuring that all elderly individuals fully enjoy the right to social participation. This indicates a gradual transition from "limited participation" to "full participation" for the elderly in China. However, further optimization of related policies and measures based on relevant evidence is still needed.

In recent years, the academic exploration of social participation policies for the elderly in China has been relatively limited, primarily focusing on three areas. First, scholars have sought to draw on international experiences, analyzing advanced policies from different dimensions and proposing recommendations tailored to China’s specific context. For instance, integrating the "Active Aging Action" initiated by the World Health Organization into China’s Eleventh Five-Year Plan and formulating the National Silver Age Action Program have established pathways for building an active aging society [13]. These efforts include suggestions across six aspects: eliminating discrimination, autonomous participation, protection from violence and abuse, social security and employment, care, and justice, all aimed at enhancing awareness of active aging [14]. Second, researchers have examined the development stages and existing issues of domestic policies, analyzing the construction and implementation of China’s social participation policy system for the elderly and summarizing its characteristics and related problems. Zhao Chenghao pointed out the lack of systematic legal support for the social participation rights of the elderly in China and proposed improving legislative and enforcement mechanisms to enhance these rights [15]. Zhu Yaoyin discussed policy issues encountered in using modern information technology to serve the elderly, suggesting the expansion of learning and social platforms for the elderly and increasing opportunities and information channels for their active engagement [16]. Third, scholars have analyzed the mechanisms and effects of policy implementation. Yang Jiajia and colleagues argued that China’s elderly social participation policies face challenges related to technological elements and scientific awareness [12]. They emphasized the need for top-level design to improve participation policies, bridge the digital divide, reasonably deploy technology platforms, and foster scientific perspectives. It is evident that current policy analysis research often relies on subjective evaluations, lacking scientific and quantifiable research methods and tools to provide objective data support.

Currently, quantitative analysis methods for policy texts are widely applied in policy analysis within the field of public administration, with research paradigms becoming increasingly standardized [17]. This provides valuable insights for the present study. Existing research typically employs either structural or content-based quantitative analysis of texts, with few studies integrating both approaches. This study aims to combine these two methods, using Rothwell and Zegveld’s "three types" of policy and technical tools to analyze China’s national aging policies since the launch of the "Global Action on Active Ageing" in 1999 [18]. Additionally, this study examines policy texts in conjunction with the social participation stages of the "intention participation period-action participation period-maintenance feedback period" cycle and visualizes the evolution of policy themes to provide a more intuitive understanding of policy implications and effects. By exploring the main focus and internal logic of China’s social participation policies for the elderly, this study seeks to unveil the "black box" of processes that are often elusive within the policies themselves, thus providing references for optimizing the pathways of social participation policies for the elderly.

## Materials and methods

### Data sources

Data on social participation policy documents for older people were collected from the Chinese Health Commission’s official website, the Chinese government policy database, the Chinese Association for the Aged’s official website, the Ministry of Civil Affairs of the PRC official website, and the Peking University Law Website. Policy selection was based on the following criteria: (1) policies enforced from 1999 to September 30, 2023; (2) social participation policies issued by the central government; (3) the policy title or text included the keywords “elderly people,” “aged,” “retired people,” “participate in society,” or “population development,” or their combinations; (4) policies with ambiguous terms and not relevant to the social participation of older people were excluded; and (5) letters, directives, catalogs, and policies that arrange specific work were not included. After retrieving and removing duplicate and irrelevant documents, 55 policy texts directly or substantively related to national-level "elderly participation" were obtained. The selected policy samples are sufficient in both quantity and quality to meet the research needs, ensuring that the overall study can proceed without any impact. These included strategies, viewpoints, announcements, and decisions from the Communist Party of China (CPC) Central Committee, the State Council of the PRC, the Ministry of Civil Affairs, the Ministry of Education, and other operational government departments (Table 1).

**Table 1:** Summary of social participation policies of older people in China.

Number	Policy name	Policy-issuing subjects	Year	Policy sources
1	Decision on Strengthening the Ageing Work	The State Council of the PRC	2000	The Chinese government policy database
2	Notification on Improving Older Persons’ Education	The Organization Department of the CPC Central Committee; Ministry of Culture of the PRC; Ministry of Education of the PRC Etc.	2001	The Peking University Law Website
3	Outline of China’ s tenth five-year Plan for Aging Cause (2001–2005)	The State Council of the PRC	2001	The Chinese government policy database
4	Opinions on Proactively Advancing the Socialized Management and Service of Enterprise Retirees	The Ministry of Human Resources and Social Security; The Organization Department of the CPC Central Committee; The National Development and Reform Commission Etc.	2003	The Chinese government policy database
5	Opinions on further giving play to the role of retired professional and technical personnel	The Organization Department of the CPC Central Committee; The Propaganda Department of the CPC Central Committee; The United Front Work Department of the CPC Central Committee Etc.	2005	The Chinese government policy database
……				
51	Notice on Issuing the Implementation Plan for Strengthening Traditional Chinese Medicine Services for Elderly Health	National Administration of Traditional Chinese Medicine; National Health Commission of the PRC	2022	The Peking University Law Website
52	Opinions on Providing Judicial Services and Guarantees for Implementing the National Strategy of Actively Responding to Population Ageing	The Supreme People’s Court of the PRC	2022	The Peking University Law Website
53	The 14th Five-Year Plan for Healthy Aging	National Health Commission of the PRC; Ministry of Education of the PRC; Ministry of Science and Technology of the PRC Etc.	2022	The Chinese government policy database
54	Opinions on Promoting the Construction of a Basic Elderly Service System	General Office of the CPC Central Committee and General Office of the State Council of the PRC	2023	The Chinese government policy database
55	Action Program for the Active Development of Meals on Wheels for the Elderly	Ministry of Civil Affairs of the PRC; National Development and Reform Commission; Ministry of Finance of the PRC Etc.	2023	The Chinese government policy database

### Study design

**Fig 1:**
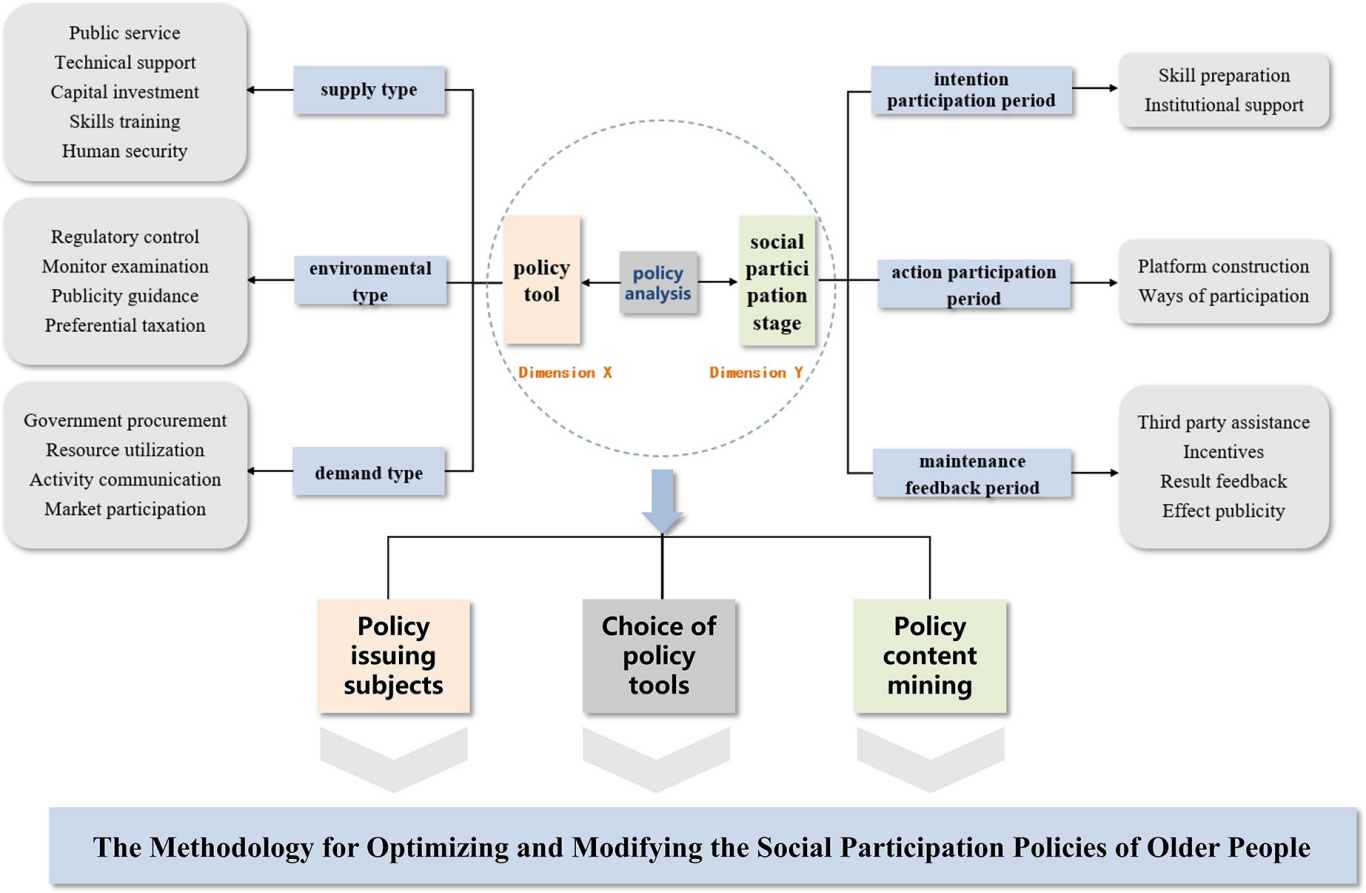
Basic analysis framework of social participation policy tools of older people.

The methods or techniques used by the government to implement policy goals are known as policy tools [19], and they are a crucial component for policy analysis. Supply type, environmental type, and demand type are the “three types” of policy tools proposed by Rothwell and Zegveld. At the macroscopic level, the three policy tools encourage and control the forms, components, and multi-subject engagement of older social participation, medical care, social security, and education. Accordingly, at the micro level of individual participation, this study linked the social participation stage with various policy analysis phases, successfully compensating for the technical shortcomings of the “three types” of policy tools in assessing individual demands. Through the integration of static policy tool research and dynamic elderly social participation stage research, this work enhanced the framework of policy tool research (Fig 1).

### Analysis approach

This study employs Social Network Analysis (SNA) to conduct structural metrics of texts and utilizes Text Analysis to measure text content [20]. This approach aims to comprehensively explain the evolution and internal logic of China’s elderly social participation policies based on the structure and content of key terms.

SNA visualizes and intuitively processes the connections between datasets through relevant software, then analyzes their distribution and development patterns. First, policy samples are tokenized using Rost Content Mining 6.0 software. Words such as "the," "and," "people," and "to," which are not closely related to the research content, are placed in a filter word list. Certain inappropriate analytical combinations are manually adjusted, and then the policy samples are analyzed using methods such as word frequency analysis. Next, word frequency analysis is conducted to statistically assess the frequency of word occurrences in policy texts, uncovering hidden features and meanings in the information texts. On this basis, a co-word matrix of core keywords in China’s elderly social participation policy content is established. This matrix is then input into UCINET 6 software to create a social network diagram. Co-occurrence analysis, burst detection, and evolution analysis are conducted through measures of density, centrality, and other metrics to identify the issues addressed by current policies and depict their evolution process and regularity. This approach helps to correctly understand and grasp the current problems and relationships within the core content of policies. By combining policy keywords within the social network, it further explores the network structure characteristics of policy keywords, thereby achieving a more scientific and reasonable understanding of policy content.

After collecting and classifying the policies on elderly social participation in China, this study employs public policy text analysis methods to uncover the underlying structure and internal logic of their evolution through coding, demonstrating prominent features of data analysis and computational thinking. First, according to the established basic analysis framework of social participation policy tools for older people (Fig 1), the content characteristics of policy texts related to elderly social participation in China are analyzed. Fifty-five social participation policies were coded independently using Nvivo 11 software. The coding rules followed the format "policy text serial number—serial number." For instance, the first item of the policy with serial number 1 was named 1–1, the first part was encoded as 1–1, 1–2, and the second part was coded as 2–1, 2–2, and so on, until the last policy. Finally, a coding Table of the analysis units of policy tools for social participation of older people was formed (Table 2), thus analyzing the main policy measures adopted by the government and the structural characteristics of policy tools, exploring the meanings and implicit information contained in policy content texts, policy environment, policy participants, policy implementation objects, and the era context of the issued policies. The advantage of public policy content analysis lies in its ability to process and analyze large volumes of text content, simultaneously discovering information that cannot be obtained solely through observation and reading [21]. Second, qualitative analysis methods such as policy text topic mining are employed for policy text content, including node analysis of topic word structures and semantic coding of phrases and sentences, achieving a visual effect. This precise analysis of the main policy measures and the use and structural characteristics of policy tools by the government provides a basis for selecting policy paths for elderly social participation in China. Based on the results of the analysis, the selection and use of policy tools for the social participation policies of older people in China are determined.

**Table 2:** Coding table of the analysis unit of policy tools.

Policy order Number	Content analysis unit	Code	Category
1	Build or expand a number of community service facilities for the elderly, welfare facilities and activity venues	1–3	Public service, Institutional support
	All elderly party members should be incorporated into the grass-roots organizations of the Party and participate in the activities of the Party organizations	1–6	Monitor examination, Institutional support
2	Encourage people from all walks of life to actively set up education for the elderly and gradually broaden the ways of socialized running schools	2–2	Publicity guidance, Institutional support
3	Actively organize and guide the elderly to learn scientific and cultural knowledge and agricultural technology	3–10	Skills training, Ways of participation
	Strengthen legislation and law enforcement, gradually form a legal protection system for safeguarding the legitimate rights and interests of older people	3–15	Regulatory control, Institutional support
……	……	……	……
54	Encourage the participation of social forces in the provision of basic elderly care services, and support property service enterprises to provide home-based community elderly care services according to local conditions.	54–2	Public service, Institutional support
55	Encouraging and guiding public welfare and charitable organizations, caring enterprises and individuals to participate in meal assistance services for the elderly in the form of charitable donations.	55–8	Market participation, Third party assistance

### Reliability test and quality control

Reliability testing is crucial for ensuring consistency in content analysis. Greater consistency indicates higher credibility in content analysis. This essential indicator ensures the trustworthiness and impartiality of the outcomes of policy analysis [22].

After the two judges coded all the policy sample categories, the results were calculated based on the reliability analysis formula. The reliability analysis of policy analysis is shown in Formula (1).

$$r=\frac{2M}{N_{1}+N_{2}}$$ (1)

“r” represented the degree to which the two judges concurred when evaluating the categories, “M” denoted the number of categories that both judges agreed upon, “N_1_” represented the number of policy coding items judged by the main judge, and N_2_” represented the number of categories judged by the secondary judge. In this study, the secondary judge coded 55 policy texts according to the policy coding category and compared the results with those of the main judge. The inconsistent results were marked as “0”, and the consistent ones were marked as “1”. Finally, the reliability value of policy analysis was obtained based on Formula (2).

$$R=\sum_{i=1}^{55}r_{i}/55$$ (2)

According to Nunnally, when the final reliability value exceeded 0.7, the previous research was credible enough [23], which indicated that the evaluation results had passed the test and could be adopted. The reliability analysis process was shown in Table 3.

**Table 3:** Reliability analysis table for coding of policy tools analysis unit.

Policy tools	Policy sample 1	Policy sample 2	……	Policy sample 54	Policy sample 55
Public service	1	1		1	1
Technical support	1	1		1	1
Capital investment	1	1		1	1
Skills training	1	1		1	1
Human security	1	1		1	1
Government procurement	1	1		1	1
Resource utilization	1	1		1	1
Activity communication	1	1		1	1
Market participation	1	1		1	1
Regulatory control	0	1		1	1
Monitor examination	1	1		1	0
Publicity guidance	1	1		0	1
Preferential taxation	1	1		1	1
Skill preparation	1	1		1	0
Institutional support	1	1		0	1
Platform construction	1	1		1	1
Ways of participation	1	1		1	1
Third party assistance	1	1		1	1
Incentives	1	0		1	1
Result feedback	1	1		1	1
Effect publicity	1	1		1	1
Reliability of each sample	91.7%	91.7%		83.3%	83.3%

## Results

### The result of policy-issuing time

**Fig 2:**
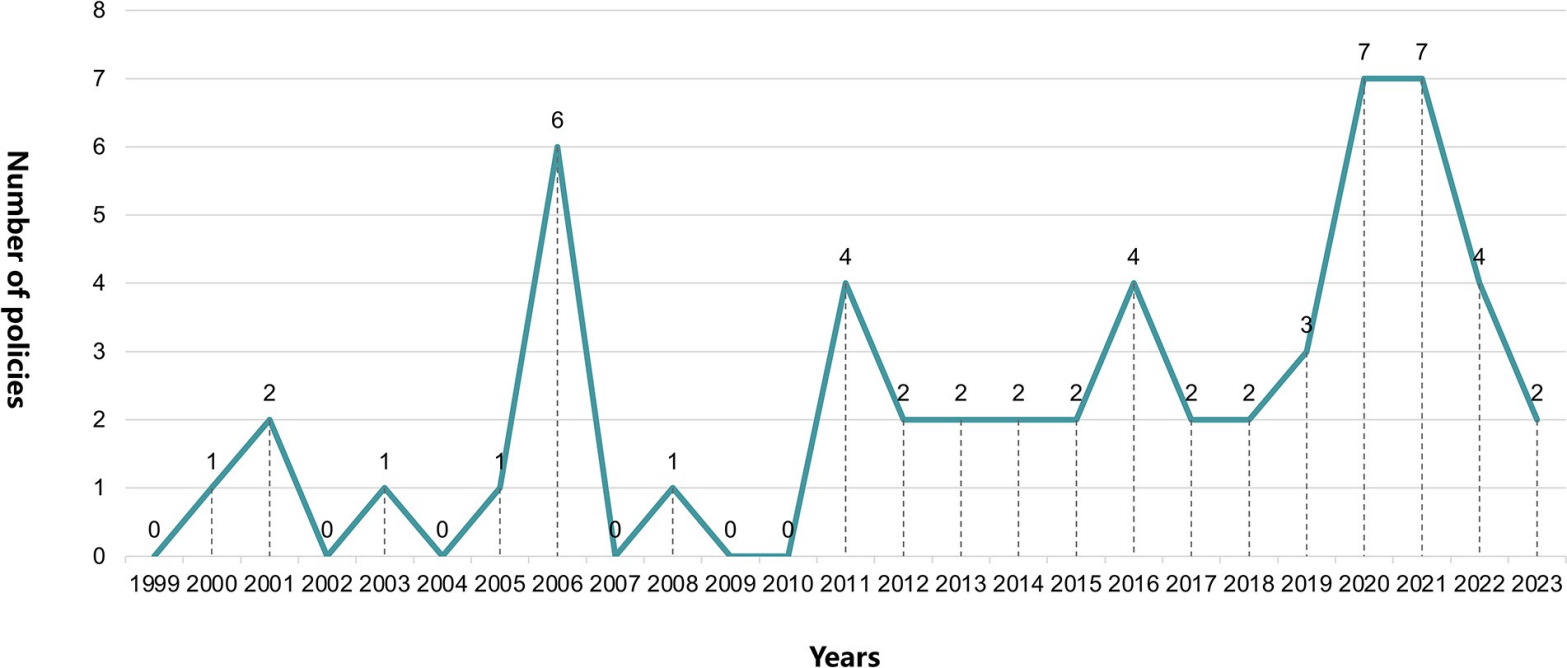
Annual quantitative development map of policy.

Analyzing the issuance timing characteristics of 55 policies reveals that, overall, from 1999 to 2023, the number of policies on elderly social participation in China shows a fluctuating upward trend, with significant turning points in 2006, 2011, 2016, and 2020. In 2006, linear development appears for the first time, with the issuance of 6 policies (Fig 2), marking the entry of China’s elderly social participation policies into the establishment stage [12]. The "Outline of the Eleventh Five-Year Plan for National Economic and Social Development of the PRC" (issued in March 2006) includes, for the first time, a dedicated chapter that officially explains the concept and significance of "actively addressing population aging." From 2007 to 2010, only one relevant policy is issued, indicating that China is still "digesting" the large number of elderly social participation policies issued in 2016, focusing on improving their implementation quality. Between 2011 and 2019, there is a noticeable increase compared to the previous stage, but overall, it exhibits a stable and gradual upward trend without a sharp rise. This indicates that China’s attention to elderly social participation gradually increases, and related policies continuously develop. From 2020 to 2022, the number of policy documents increases significantly compared to the previous two stages. Notably, the "Notice of the State Council on the Issuance of the 14th Five-Year Plan for National Development of Aging Services and the Elderly Care System" issued in 2021 explicitly proposes "promoting elderly social participation" as an independent goal, marking China’s growing determination to achieve active aging and the increasing advantages of elderly social participation.

### The result of policy-issuing subjects

Policy issuing subjects constitute the core components of the policy system, involving individuals, teams, or organizational entities that directly or indirectly participate in various stages of public policy, including the formulation, implementation, evaluation, and supervision of policy documents [24]. According to statistics, there are a total of 46 authoritative departments that issue policies on social participation of older adults, either independently or jointly. These departments are roughly divided into three levels. The highest level includes the National People’s Congress (NPC) and the Central Committee of the CPC. The second level includes the State Council, the Standing Committee of the NPC, and the Supreme People’s Court. The third level consists of departments, direct agencies, and specially designated agencies under these institutions, such as the National Development and Reform Commission, the Ministry of Education, the Ministry of Science and Technology, the Ministry of Culture and Tourism, the National Health Commission, and the National Working Commission on Aging.

### The result of network analysis

#### Analysis of social network structure of policy keywords

**Fig 3:**
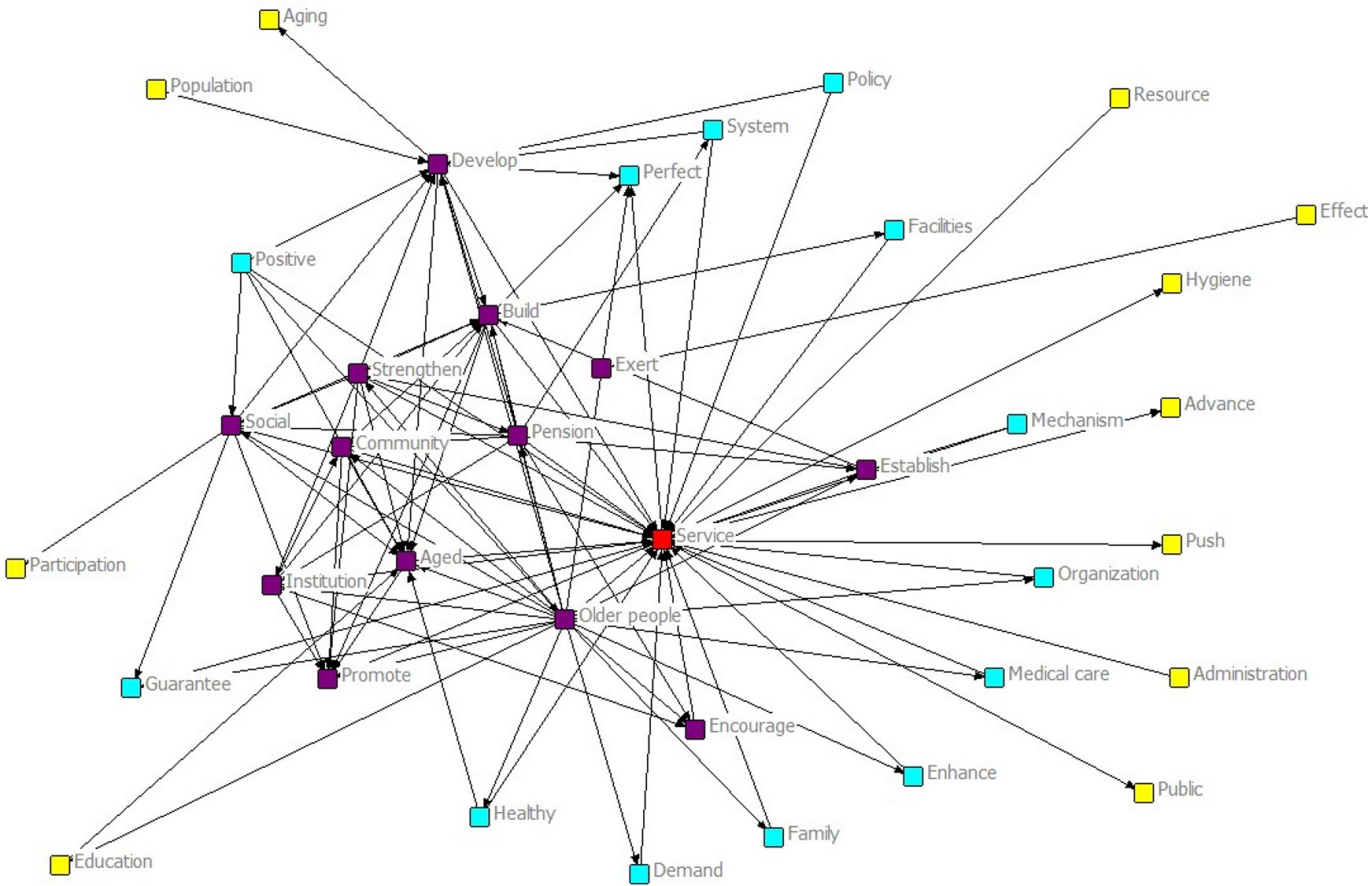
The map of social network analysis.

The map of social network analysis of the policy was developed after a statistical analysis of the keyword frequency in the policy text on social participation of older people (Fig 3). The network diagram revealed a four-layer structure similar to “core-sub-core-transition-edge.” The core layer consisted of the word “service.” The sub-core of the second layer included the words “older people,” “community,” “pension,” “social,” and “institution,” demonstrating that encouraging community service and fostering an active aging society were the primary objectives of older social engagement. The transition layer contained keywords such as “facilities,” “family,” “demand,” “medical care,” “organization,” and “policy,” indicating that the policy not only encourages the social participation of older people but also pays special attention to the participation and needs of all markets, thereby enhancing the positive degree of social participation among older people. In the edge layer, keywords such as “education,” “public,” and “hygiene” were not closely related to the core keywords, suggesting that the integration of resources is not yet clear, and the management and promotion of social participation among older people remain relatively insufficient.

#### Network density of policy issuing subjects

Network density ranges between [0, 1], with higher values indicating closer relationships [25]. This implies that the closer the value is to 1, the more the network impacts the attitudes and behaviors of the policy issuing subjects. The network density of the joint policy subjects for social participation among older people in China was 0.6258, with a mean square deviation of 2.1065.

#### Degree centrality of policy issuing subjects

Degree centrality explains the interaction between a particular node and other nodes within a network. A higher degree centrality value indicates that a node is more central and influential within the network [25]. The measured degree centrality for the overall network relationship was 1.24%, indicating a trend towards lower centrality in the network. This indicates that it is unlikely for many policy subjects to jointly participate in the formulation and release of policies.

### The result of text analysis

#### Reliability test

Based on reliability test Formulas 1 and 2 provided earlier and the outcomes presented in Table 3, the average reliability analysis result of content evaluation by the two judges was 90.3%. This demonstrates a high level of trustworthiness in the analysis findings from this study.

#### Statistics on policy tools category dimensions

**Fig 4:**
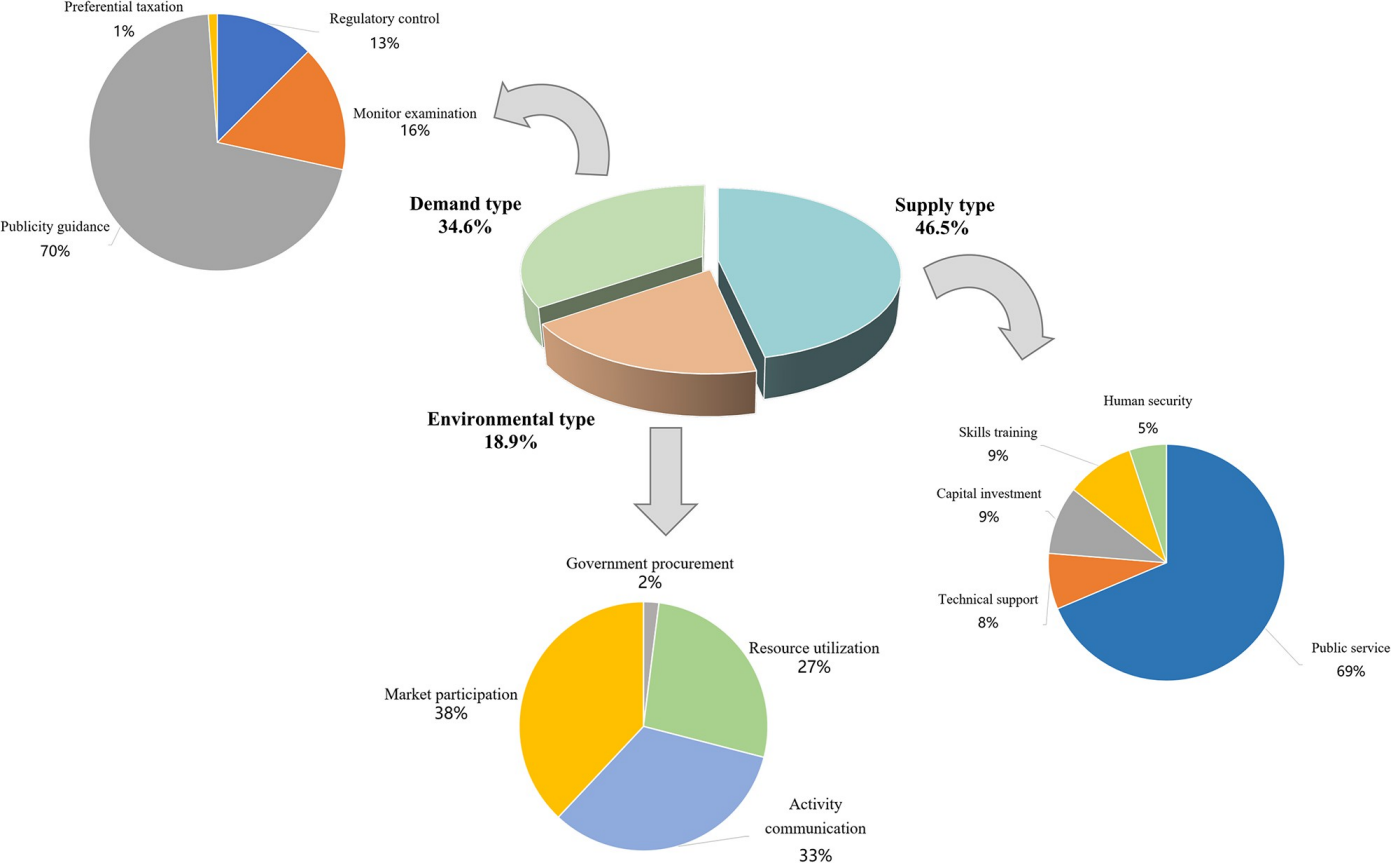
Effect map of category distribution of policy tools.

The statistical analysis results of policy tools category dimensions were detailed in Table 4 and Fig 4. Supply-type policy tools emerged as the predominant means (46.5%). Environmental-type policy tools followed at 34.6% of the 55 policy texts analyzed, while demand-based policy tools were the least utilized, accounting for only 18.9% of the total. Significant variations were observed in the use of specific technical indicators as policy tools. For example, the most frequently deployed supply-type policy tools included public services (31.9%), technological support, and human security (both 4.3%). Capital investment (3.5%) and skills training (2.4%) were comparatively less utilized. Among environmental policy tools, publicity guidance policy tools constituted 24.4%, while regulatory control and monitoring examination represented only 9.8%. Preferential taxation policies were notably sparse, with only one policy provision applying this tool. Among demand-type policy tools, market participation (7.1%) showed relatively higher usage, while activity communication (6.3%), resource utilization (5.1%), and government procurement (0.4%) were less prominent.

**Table 4:** Category allocation ratio of policy tools.

Category of tools	Name of tools	Policy coding	Quantity (item)/proportion (%)			
**Supply type**	Public service	1–1、1–2、1–3、1–4、2–1、3–1、3–2、3–3、3–4、3–5、3–6、3–7、3–8、4–1、5–1、5–2、5–3、6–1、6–2、6–3、7–1、7–2、7–3、7–4、8–1、10–1、10–2、11–1、12–1、14–1、15–1、16–1、17–1、17–2、17–3、18–1、19–1、20–1、20–2、20–3、21–1、21–2、21–3、21–4、22–1、22–2、23–1、24–1、27–1、28–1、29–1、30–1、30–2、31–1、32–1、32–2、33–1、33–2、36–1、37–1、41–1、42–1、42–2、43–1、45–1、46–1、47–1、47–2、48–1、49–1、50–1、51–1、51–2、51–3、52–1、52–2、53–1、54–2、55–1、55–2、55–3	81	31.9%	118	46.5%
	Technical support	3–11、6–5、14–2、20–4、22–3、29–3、37–2、41–2、44–1	9	4.3%		
	Capital investment	1–5、3–14、4–3、7–7、7–8、7–9、17–4、20–5、21–5、28–2、38–1	11	3.5%		
	Skills training	3–9、3–10、6–4、7–5、7–6、29–2、46–2、48–2、49–2、49–3、55–4	11	2.4%		
	Human security	3–12、3–13、4–2、5–4、6–6、24–2	6	4.3%		
**Demand type**	Government procurement	24–10	1	0.4%	48	18.9%
	Resource utilization	3–27、3–28、5–7、6–11、7–16、15–6、17–8、17–9、24–11、27–3、28–9、28–10、51–7	13	5.1%		
	Activity communication	1–9、3–24、3–25、4–5、6–9、12–3、13–2、14–3、15–5、20–7、22–5、24–6、28–7、31–5、34–3、51–6	16	6.3%		
	Market participation	1–10、3–26、6–10、7–14、7–15、9–1、20–8、22–6、24-、24–8、24–9、25–2、28–8、32–4、35–2、45–2、48–4、55–8	18	7.1%		
**Environmental type**	Regulatory control	3–15、6–7、17–5、21–6、24–3、28–3、31–2、33–3、43–2、51–4、54–3	11	4.3%	88	34.6%
	Monitor examination	1–6、3–16、3–17、3–18、3–19、7–10、7–11、15–2、21–7、25–1、31–3、42–3、44–2、50–2	14	5.5%		
	Publicity guidance	1–7、1–8、2–2、3–20、3–21、3–22、3–23、4–4、5–5、5–6、6–8、7–13、12–2、13–1、15–3、15–4、16–2、16–3、17–6、17–7、18–2、19–2、20–6、21–8、22–4、23–2、24–4、24–5、26–1、26–2、27–2、28–4、28–5、28–6、30–3、30–4、31–4、32–3、33–4、34–1、34–2、35–1、38–2、39–1、40–1、42–4、42–5、44–3、44–4、44–5、47–3、48–3、49–4、50–3、50–4、50–5、51–5、53–2、54–1、55–5、55–6、55–7	62	24.4%		
	Preferential taxation	7–12	1	0.4%		
**Total**	N/A	N/A	254	100%	254	100%

#### Statistics on social participation stage category dimensions

**Fig 5:**
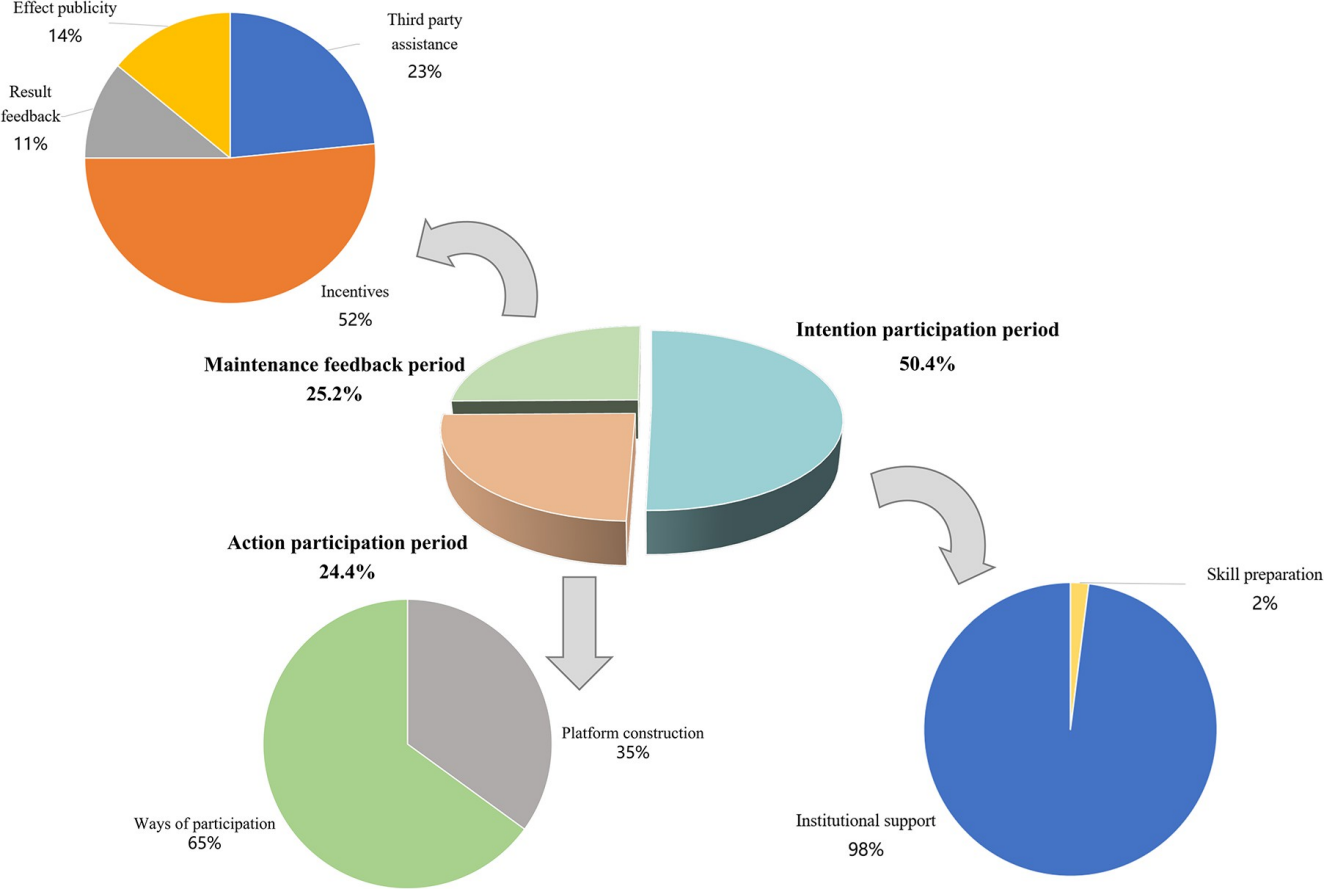
Effect map of category distribution of social participation stage.

The statistical analysis results of social participation stage category dimensions were presented in Table 5 and Fig 5. The maintenance feedback period (25.2%), action participation period (24.4%), and intention participation period (50.4%) were identified as the three primary areas of focus in current policies. Institutional support policy tools were the most prevalent, comprising nearly half of the total (49.2%), followed by participation methods (15.7%) and incentives (13.0%). Platform construction and third-party assistance policy tools were relatively evenly distributed at 8.7% and 5.9%, respectively. Effect publicity (3.5%), result feedback (2.8%), and skill preparation (1.2%) were utilized to integrate existing market resources and foster the development of human resources for older people.

**Table 5:** Category allocation ratio of social participation stage.

Category of tools	Name of tools	Policy coding	Quantity (item)/proportion (%)			
**Intention participation period**	Skill preparation	3–9、4–2、55–4	3	1.2%	128	50.4%
	Institutional support	1–1、1–2、1–3、1–4、1–5、1–6、1–9、2–1、2–2、3–1、3–13、3–14、3–15、3–16、3–17、3–18、3–20、3–23、3–4、4–1、4–4、5–2、5–4、5–5、6–1、6–10、6–11、6–6、6–7、7–1、7–10、7–12、7–3、7–4、7–5、7–6、7–7、7–8、8–1、9–1、10–1、10–2、11–1、13–1、14–1、15–3、15–6、16–1、16–2、16–3、17–1、17–2、17–3、17–4、17–5、17–9、18–2、20–1、20–2、20–3、20–5、20–8、21–1、21–2、21–4、21–5、21–6、21–8、22–1、23–1、23–2、24–1、24–11、24–3、26–1、26–2、27–1、28–10、28–3、28–8、29–1、30–1、30–3、31–1、31–2、31–3、31–5、32–1、32–2、32–3、33–1、33–2、33–3、34–2、36–1、37–2、38–1、41–1、42–1、42–3、42–5、43–2、44–5、45–2、46–1、46–2、47–1、48–1、48–4、49–1、50–1、50–3、51–1、51–3、51–4、51–7、52–1、52–2、53–1、54–2、54–3、55–1、55–2、55–3、55–7	125	49.2%		
**Action participation period**	Platform construction	3–11、3–2、3–5、5–3、6–2、6–5、7–14、7–2、12–1、14–2、15–1、20–4、22–2、22–3、27–2、28–9、30–2、43–1、45–1、47–2、49–2、51–2	22	8.7%	62	24.4%
	Participation methods	1–7、3–10、3–22、3–25、3–28、3–3、3–6、3–7、4–5、5–1、6–9、7–13、7–15、7–16、12–2、13–2、14–3、15–5、17–6、17–8、19–2、20–7、22–5、24–6、28–7、29–2、29–3、34–3、35–1、37–1、39–1、42–4、47–3、48–2、48–3、49–3、51–5、51–6、53–2、54–1	40	15.7%		
**Maintenance feedback period**	Third party assistance	1–10、3–12、3–8、12–3、22–4、22–6、24–7、24–8、24–9、25–2、27–3、32–4、40–1、55–6、55–8	15	5.9%	64	25.2%
	Incentives	1–8、3–26、3–27、4–3、5–6、5–7、6–3、6–4、6–8、7–9、15–4、17–7、18–1、19–1、20–6、21–3、24–10、24–2、24–4、28–1、28–2、28–5、30–4、33–4、35–2、38–2、41–2、42–2、44–1、44–4、50–4、50–5、55–5	33	13.0%		
	Result feedback	3–19、7–11、15–2、21–7、25–1、44–2、50–2	7	2.8%		
	Effect publicity	3–21、3–24、24–5、28–4、28–6、31–4、34–1、44–3、49–4	9	3.5%		
**Total**	N/A	N/A	254	100%	254	100%

## Discussion

The analysis of the "three types" policy tools and the "three stages" dimension of elderly participation revealed that the issuing authorities and the primary content of these policies significantly influence the social engagement of older adults. The findings from this policy analysis can be utilized to identify challenges in the policy transformation process.

### Analysis based on the dimension of policy tools

#### Overuse of supply-side policy tools

Statistical data analysis reveals that nearly half of the policy texts concerning elderly social participation in China employ supply-side policy tools (46.5%), whereas demand-side policy tools account for only 18.9%. This indicates an overuse of supply-side policy tools in policies aimed at promoting elderly social participation in China. The primary reason for this phenomenon is the government’s focus on constructing an external policy environment to facilitate various aspects of elderly participation in society through external supply [26]. Among the three types of policy tools, public services (69%) and publicity and guidance (70%) are particularly overused. These tools can indeed encourage more application in policies related to elderly social participation. However, publicity and guidance mainly serve as macro-level directions for elderly participation and do not provide substantial resource inputs to promote their participation in social activities. The overuse of public service tools is often due to the failure of relevant departments to implement previously issued policies or the defects in earlier policies that hindered the achievement of their intended goals [27]. Consequently, to fulfill related tasks, subsequent policy documents need to continue adjusting and emphasizing relevant content, leading to the overuse of publicity and public service policy tools.

#### Insufficient application of demand-side policy tools

The policy content related to elderly social participation in China shows a clear deficiency in the application of demand-side policy tools (18.9%). Demand-side policy tools, through methods such as government procurement, resource utilization, and market participation, can reduce market instability, thereby lowering the risks for elderly social participation and further encouraging their engagement [28]. Compared to environmental policy tools, demand-side tools have a greater impact and efficacy in promoting social participation. For instance, government procurement can provide services for elderly social participation by purchasing public outcomes, thereby reducing their risks and motivating market participants [29, 30]. However, only one policy item addresses this aspect. Resource utilization and activity exchanges can improve the conditions for elderly social participation and further promote attention from enterprises, universities, and research institutions. This can also alleviate the government’s burden in terms of manpower, financial resources, and venues, making it a highly promotable policy tool for encouraging elderly social participation. In the previous formulation and design of policies for elderly social participation, the Chinese government did not sufficiently consider demand-side policy tools. Therefore, in the future, it is necessary to fully coordinate and adjust the balance of the policy system to enhance its role in promoting social participation.

### Policy analysis based on the dimension of elderly participation stages

#### Lack of effective policy tool support for social participation process categories

Following an analysis of the use of policy tools in elderly social participation policies along the X-Y dimensions (policy tool dimension and social participation process dimension), this study concludes that the frequency of policy tool use in the action participation and maintenance feedback stages is relatively insufficient. Specifically, the proportions of skill preparation, outcome feedback, effect publicity, third-party assistance, and platform construction in the total sample policies are 1.2%, 2.8%, 3.5%, 5.9%, and 8.7%, respectively. As the three fundamental stages of policy text analysis in this study, the design and participation of elderly social activities require support from these three stages to provide basic skills and platform information. However, the results show that many policy tools have not been fully and effectively utilized. In terms of technical elements, there is a lack of use of tools for technical support and skill training, necessitating increased investment in these areas. Furthermore, regarding technological platforms, there is a need to strengthen the focus on constructing facilities related to activity exchanges and market participation. Therefore, it is crucial to emphasize the use of policy tools for the three stages of social participation in the design of elderly social participation policies. By effectively utilizing policy tools, the focus on the entire process of elderly social participation can be adjusted, ultimately providing support for policy establishment and development.

#### Insufficient understanding and recognition of scientific awareness

From a scientific perspective, the study reveals that the policy texts lack a sufficient understanding and recognition of the scientific view of aging. The primary reason is that the formulation and issuance of policies did not fully consider the elderly population’s participation in society, failing to accept the reality that the elderly can contribute to social governance [31]. The 18th National Congress emphasized the need to view the aging society positively, fundamentally reversing the public’s negative or even fearful attitude toward an aging society [32]. However, there is still a gap in preparing the entire society to actively respond to population aging. The government, society, universities, and enterprises are the foundational forces driving the development of elderly social participation. This study also finds that policy tools such as "third-party assistance," "resource utilization," "market participation," and "platform construction" have not received sufficient attention, indicating a need for further exploration of ways and means for elderly social participation.

#### Core focus on institutional support innovation

The core focus of current elderly social participation policies in China is on innovating institutional support for elderly participation. There is a need to further enhance the innovation and practicality of utilizing technological platforms. The development of elderly social participation still requires support and feedback from incentive measures. Additionally, the importance of outcome feedback and effect publicity in the maintenance feedback stage is not adequately recognized in policy guidance. Therefore, one solution to these issues is to effectively establish a scientific view of aging among the public [33]. During the process of elderly participation in social activities, government departments should employ various policy tools to coordinate and promote the positive development of elderly social participation. Thus, in the future formulation of elderly social participation policies, it is essential to clarify the selection and use of policy tools in a timely manner. Government departments need to consider strategic planning to layout policy content and systems. By understanding and practicing elderly social participation policies through policy subjects, the policy system and direction for encouraging elderly social participation can be reasonably planned and developed.

#### Neglect of more effective policy tools

In the application of supply-side policy tools, the policies for elderly social participation have not effectively utilized tools such as human resource assurance, technical support, funding, and skill training. Particularly, human resource assurance is mentioned in only six policy texts where the government maximizes the potential of the elderly to encourage social participation. For the elderly to participate in "Silver Age Actions" or play a role in educational and research institutions, relevant units require support from government funding, human resources, and technology. In the case of environmental policy tools, tools such as tax incentives, regulatory control, and supervision and evaluation have not received sufficient attention from government agencies, accounting for only 10.2% of overall usage. This indicates a lack of specialized tax incentives and regulatory policies to encourage elderly social participation. Especially, there is only one tax incentive policy clause. The use of environmental policy tools can provide a positive policy support environment for elderly social participation and indirectly promote their involvement [34, 35]. Social activities involving the elderly require tax incentives and regulatory support from the government to more effectively encourage various parties to prioritize and engage in the mental and physical health development of the elderly. This can help expand and enrich the avenues and content of elderly social activities, thereby enhancing their enthusiasm for participation. In terms of demand-side policy tools directly encouraging elderly social participation, policy items related to resource utilization, activity exchange, and market participation each account for less than 10%, with government procurement (0.4%) failing to stimulate elderly social participation. Therefore, future policies should be optimized and adjusted to address these weaknesses.

### Policy optimization suggestions

China’s policy on elderly social participation has evolved through five stages: emergence, initial, exploration, establishment, and deepening maturity [8]. A quantitative analysis of the national policy documents reveals that these policies address various stages of participation and involve a combination of different policy tools. With technological advancement and changing needs of the elderly population, there is a need for a more systematic and forward-looking top-level design of elderly social participation policies.

#### Accelerating the systematization and precision of technological elements in policies for elderly social participation

The process of elderly participation is not merely linear but involves the integration of various elements [36]. Government departments should reduce the frequency and reliance on supply-oriented and environment-oriented policy tools, transitioning to a greater emphasis on guidance and public service tools. Simultaneously, increasing the use of policy tools such as technical support, personal safety, skills training, activity exchange, and resource utilization is essential. Effective communication and consensus-building with stakeholders, alongside the use of new technologies to enhance policy implementation efficiency, will ensure a more focused and efficient approach to elderly social participation. For instance, in continuing education for the elderly, educational institutions should access relevant information through a government-established information service platform, optimizing resource allocation through government procurement.

#### Integrating social organizations via technological platforms to mobilize diverse stakeholder engagement

Elderly social participation involves the collective effort of government departments, market institutions, social forces, and communities [37]. Establishing a support system based on service and demand orientation, considering the benefits of participants in terms of funding, talent, and technology, is essential to accurately understanding and meeting the needs of the elderly. Governments should not only increase investment in social security and welfare but also leverage the advantages and capabilities of other market entities. Utilizing big data and the Internet of Things to establish market-based platforms for government outsourcing and service procurement will encourage the involvement of social organizations, elderly organizations, charitable organizations, and talent intermediary services in planning relevant activities. These could include partnerships, elderly volunteer services, and time banks, as well as organizing various forms of elderly talent exchange events to create localized elderly brand activities and actively provide services to the elderly.

#### Addressing the digital divide between the elderly and new technologies is imperative

Recognizing the different technological needs of early, middle, and late old age groups is essential. For elderly individuals capable of participating in society, technological education and training should be prerequisites for labor participation [38, 39]. In today’s highly advanced technological environment, leveraging "Internet+" and smart elderly care initiatives can provide internet training for elderly individuals willing to learn, enabling them to utilize various online functions. This will help prevent social isolation and digital exclusion, ensuring they are not left behind by technological advancements. Effective use of digital technology, combined with systematic innovation through cross-sector collaboration, is necessary to bridge the digital divide. Supporting the orderly development of industry-specific social organizations established by enterprises, enhancing the quality and application of smart products through industry regulation and supervision, and improving scientific literacy will ensure that technological advancements benefit the elderly. Strengthening internet-based elderly care and industry development will foster a new landscape of cross-sector innovation and collaborative development, forming a healthy ecosystem for technology-assisted elderly care.

## Limitations

There are several limitations to this study. Firstly, the coding process relies on the author’s subjective experience, which may introduce some biases. Additionally, the study lacks a thorough discussion on policy evolution. Future research could address these limitations by considering the following aspects: first, incorporating the characteristics of policy texts or activities such as waste classification to expand the analysis dimensions (e.g., temporal dimension) and constructing a multi-dimensional analysis framework. Secondly, employing more advanced analytical tools could provide a more comprehensive depiction of the current state and characteristics of policy tool utilization.

## Conclusions

In conclusion, this study introduced a new approach involving network graph analysis and policy coding analysis to examine the transformation of Chinese social participation policy texts for older people from 1999 to 2023. The network graph analysis revealed changes in the policy-issuing subjects and topic words. The results of the text analysis revealed imbalances in the use of different types of policy tools, and the policy support system for social participation of older people has undergone a transformation from single-factor management to overall system management. Through the analysis of the characteristics and evolution trends of the policy, combined with the practical experience of developed countries, this study put forward corresponding optimization countermeasures and suggestions, providing a scientific basis for the relevant analysis and research of social participation policy for older people in China. The method used in this study is a valuable means to review policy trends, which can be readily applied to other policy analyses and indicate the development direction for further policy research.

## Supporting information

S1 Appendix 55 original policy documents. (DOCX)

S2 Appendix List of policy names. (XLSX)
