# Supplementary material for: The transformation of 20-year social participation policies of older people in China: Network analysis and text analysis
Source: PLoS One. 2024 Aug 12;19(8):e0308401. doi: 10.1371/journal.pone.0308401 (PMC11318893; doi:10.1371/journal.pone.0308401)
Supplement: S1 Appendix — (DOCX) [file pone.0308401.s001.docx]

**中共中央、国务院关于加强老龄工作的决定**

老龄问题涉及政治、经济、文化和社会生活等诸多领域，是关系国计民生和国家长治久安的一个重大社会问题。全党全社会必须从改革、发展、稳定的大局出发，高度重视和切实加强老龄工作。

一、充分认识加强老龄工作的重大意义

（一）目前，我国60岁以上人口已达到1．26亿，其中65岁以上人口达到8600万，分别占总人口的10％和7％。按照国际通行标准，我国人口年龄结构已开始进入老龄化阶段。据预测，今后一个时期我国老年人口还将以较快速度增长，至2015年60岁以上人口将超过2亿，约占总人口的14％。

人口平均寿命延长，老年人口增加，是我国社会主义制度优越性的体现和社会文明进步的重要标志，是经济发展社会进步、人民生活水平提高、医疗卫生条件改善的重大成果。但是，人口老龄化也会给我国经济和社会发展带来一系列深刻影响。采取积极措施，加强老龄工作，是一项重要而紧迫的战略任务。

（二）党和人民政府历来十分关心老年人。新中国建立后特别是改革开放以来，国家颁布实施了一系列维护老年人权益的法律法规和政策，加强了尊老爱幼思想教育，初步建立了养老、医疗等社会保障制度，老年福利、卫生、文化、教育、体育等事业有了一定发展，老年人的生活水平和生活质量不断提高。老龄工作取得的进展和成绩，对推动经济建设和社会发展起到了重要作用。但是，也要清醒地看到，我国老龄工作基础还比较薄弱，不能很好地适应人口老龄化的要求。主要问题是：对人口老龄化问题认识不足，老龄工作政策、法规不够健全，社会保障制度尚不完善，社区管理和老年服务设施、服务网络建设滞后，老年思想政治工作薄弱，侵犯老年人合法权益的现象时有发生。对此，我们必须高度重视，认真解决。

（三）老年人是社会的重要组成部分，他们为中国革命和建设作出了重要贡献。满足广大老年人日益增长的物质和文化生活需要，让老年人共享经济建设和社会发展的成果，是中国共产党全心全意为人民服务根本宗旨的体现，是在新的历史条件下贯彻落实江泽民同志关于"三个代表"，重要思想的体现，也是国家和社会义不容辞的责任。在社会主义市场经济条件下，弘扬中华民族传统美德，形成敬老、养老、助老以及代际和谐的良好社会风尚，是社会主义精神文明建设的一项重要内容。正确处理和解决人口老龄化过程中出现的各种矛盾和问题，切实保障老年人的合法权益，对促进经济建设和社会发展具有重要意义。

二、老龄工作的指导思想、原则和目标

（四）我国老龄工作的指导思想是：以马克思列宁主义、毛泽东思想、邓小平理论为指导，贯彻党的十五大精神，从我国的基本国情出发，适应人口老龄化的发展趋势，完善社会保障制度，建立健全社区管理和社区服务体系，发展老年服务业，维护老年人的合法权益，加强老年思想政治工作，开创老龄工作新局面。

（五）加强老龄工作，发展老龄事业要遵循以下原则：坚持老龄事业与国民经济和社会发展相适应，促进老龄事业健康发展；坚持家庭养老与社会养老相结合，充分发挥家庭养老的积极作用，建立和完善老年社会服务体系；坚持政府引导与社会兴办相结合，按照社会主义市场经济的要求积极发展老年服务业，坚持道德规范与法律约束相结合，广泛开展敬老养老道德教育，加强老龄工作法制建设，坚持关心老年人生活以及老龄妇女的特殊问题与加强思想政治工作相结合，使广大老年人物质生活得到改善，精神文化生活更加丰富；坚持统筹规划与分类指导相结合，因地制宜地开展老龄工作，发展老龄事业。

（六）今后一个时期我国老龄事业发展的主要目标是：从我国社会主义初级阶段的基本国情出发，努力建立和完善有中国特色老年社会保障制度和社会互助制度；建立以家庭养老为基础、社区服务为依托、社会养老为补充的养老机制；逐步建立比较完善的以老年福利、生活照料、医疗保健、体育健身、文化教育和法律服务为主要内容的老年服务体系，切实提高老年人的物质和精神文化生活水平，基本实现老有所养、老有所医、老有所教、老有所学、老有所为、老有所乐。

三、切实保障老年人的合法权益

（七）全社会都要依据《中华人民共和国宪法》和《中华人民共和国老年人权益保障法》等法律法规，切实维护和保障老年人的合法权益。

要加强法制建设，进一步完善有关维护老年人权益的法律法规，加大执法和监督力度，依法处理和打击侵犯老年人合法权益的不法行为。依法取缔伤害老年人身心健康、宣传迷信邪说、侵害老年人合法权益的非法组织。

要在全社会积极开展维护老年人合法权益的法制教育和普法工作。各级司法行政和宣传部门要把老年人权益保障法等相关法律法规纳入普法计划，加大宣传力度，进一步提高全体公民维护老年人合法权益的自觉性和法律意识。老年人也要学法、懂法、守法，依法维护自身的合法权益。

要重视保护老年人合法权益，健全法律援助制度，加强老年人法律服务工作，使老年人能够就地、就近、及时地得到优质的法律服务。各级司法行政部门对需要获得律师及其他法律帮助但又无力支付法律服务费用的老年人，要按照有关规定向他们提供法律援助。各级人民法院对老年人因合法权益受到侵害提起诉讼交纳诉讼费确有困难的，要给予缓交、减交或免交的优待。

要大力弘扬中华民族传统美德，在全社会广泛开展敬老、养老、助老的道德教育，并与开展文明社区、文明村镇、文明家庭创建活动结合起来。中央和省级广播电视机构要开办老年节目，地、县级广播电视机构要结合本地情况进行转播，其他有条件的地方也可开办老年节目。中小学校要把敬老、养老、助老作为德育的重要内容纳入教育计划。要综合运用行政、法律和宣传、教育等手段，在全社会树立尊重、关心、帮助老年人的社会风尚。

（八）完善社会保障制度，逐步建立国家、社会、冢庭和个人相结合的养老保障机制，确保老年人生活、医疗等方面的基本需求。

在城镇，要建立起以基本养老保险；基本医疗保险、商业保险、社会救济、社会福利和社会互助为主要内容的比较完善的养老保障体系。逐步建立起独立于企事业单位之外、资金来源多渠道、管理服务社会化的基本养老保险制度。要进一步完善城市居民最低生活保障制度，对实际收入低于所在城市最低生活保障线的老年人，要纳入最低生活保障范围，发放最低生活保障金。积极推进医疗保险制度改革，建立覆盖城镇所有用人单位及其职工的基本医疗保险制度，落实离休和退休人员的医疗保障政策，发展各种类型的补充医疗保险，满足老年人的基本医疗需求。

在农村，要坚持以家庭养老为主，进一步完善社会救济和以保吃、保穿、保住、保医、保葬为内容的"五保"供养制度，倡导村民互助。有条件的地区可探索多种社会养老的路子。不断完善农村合作医疗制度，积极探索多种形式的农村医疗保障制度，加快农村医疗卫生组织建设，完善农村基层卫生服务网络，切实解决贫困地区老年人缺医少药问题。

（九）老年人有受赡养权利，赡养人特别是子女要依法履行赡养义务。倡导赡养人之间签订《家庭赡养协议书》，并由基层组织监督执行。要切实保障老年人住房、财产、继承等合法权益，重视和解决好老年妇女问题。要维护老年人婚姻自由的权利。要移风易俗，转变观念，支持单身老年人自由择偶结婚。对再婚老人，子女要给予理解和支持，并继续依法承担赡养义务。提倡和鼓励老年人之间建立互助关系。

（十）重视发挥老年人的作用，坚持自愿和量力、社会需求同个人志趣相结合的原则，鼓励老年人从事关心教育下一代、传授科学文化知识、开展咨询服务、参与社会公益事业和社区精神文明建设等活动。

四、发展老年服务业

（十一）要加强社区建设，依托社区发展老年服务业，进一步完善社区为老年人服务的功能。今后企事业单位的退休人员要逐步与所在单位相脱离，由社区组织管理和服务。要充分发挥社区组织在老龄事业发展中的积极作用。加快社区老年服务设施和服务网络建设，努力形成设施配套、功能完善、管理规范的社区老年服务体系。

各地要充分利用现有设施，积极兴办不同形式、不同档次的老年福利院、老年护理院、老年公寓、托老所等，为老年人提供生活照料、文化、护理、健身等多方面的服务。各部门、各单位的老年服务设施要逐步向社会开放。倡导社会互助，积极开展扶老助困志愿活动。各级人民政府要制定有关规定，在参观、游览、乘坐公共交通工具等方面，对老年人给予优待和照顾。

各级医疗卫生机构要大力开展多种形式的老年医疗保健服务，逐步建立起完善的社区卫生服务机构，健全老年医疗保障服务网络，提高服务质量。增加社区老年医疗保健设施，发展家庭病床，采取定点、巡回、上门服务等多种形式，为老年人提供预防、医疗、保健、护理、康复和心理咨询等服务。积极开展各种形式的健康教育，普及老年保健和卫生科学知识，增强老年人自我预防和保健技能。

各级文化、体育、广播电视等部门和工会、妇联等群众团体要进一步加强老年文化体育工作，发展老年文化体育事业。要建立社区老年活动中心或活动站。现有图书馆、群众艺术馆、文化馆、文化站、公共体育场所等要为老年人提供优先优惠服务，群众艺术馆、文化馆要建立老年文化活动中心，城区、乡镇的文化站要建立老年文化活动室。要组织老年人开展体育健身和文化娱乐活动，提倡科学文明健康的生活方式。各级文化部门要积极组织创作老年人喜闻乐见的优秀作品，组织开展丰富多彩的老年文化活动。出版部门要组织出版适合老年人特点的图书、音像制品和电子出版物，满足老年人的精神文化需求，丰富老年人的精神文化生活。

各地要重视发展老年教育事业，发展广播、电视、网络和函授教育，鼓励和指导社会力量按照有关规定兴办各类老年学校。各种老年教育主要为老年人提供物质文化生活所需要的知识和技能；使更多的老年人能就近参加学习。

（十二）老年服务业的发展要走社会化、产业化的道路。鼓励和引导社会各方面力量积极参与、共同发展老年服务业，逐步形成政府宏观管理、社会力量兴办、老年服务机构按市场化要求自主经营的管理体制和运行机制。

（十三）要培育和发展老年消费市场。老年人是一个庞大的社会群体，具有不同的消费需求，要积极研制开发适合老年人特点的产品和服务项目，引导老年人合理消费，满足老年人不同层次、不同类型的消费需求。

（十四）各级人民政府要把老龄事业纳人国民经济和社会发展中长期规划和年度计划。要高度重视社区建设，认真做好"十五"期间社区建设规划。要根据实际需要和建设条件，在充分利用现有设施的基础上，新建和扩建-批社区老年服务设施、福利设施和活动场所。非营利性老年福利设施建设所需资金以各级人民政府投入为主，同时应当制定政策，鼓励和引导社会力量积极兴办老年福利机构。

各级发展计划部门在制定投资计划、安排投资项目时，要加大对老年服务设施的投入。城市建设、旧城改造、居住区建设要将老年服务设施纳入规划并认真付诸实施。到"十五"末期，基本实现每个县（市）至少有一所老年活动场所，地级以上市有一批社区老年服务设施、福利设施和活动场所，街道办事处有老年综合福利服务设施。乡镇要努力办好敬老院，有条件的地方要逐步将敬老院建设成综合性多功能的老年福利服务中心。

（十五）要坚决贯彻落实党中央、国务院有关方针政策，确保城市居民最低生活保障金和离退休人员基本养老金按时足额发放，不得拖欠，并随着经济发展合理增长。要进二步完善农村"五保"供养制度，提高供养水平，扩大农村敬老院的服务范围。要特别关注特困老年人的生活，加大对特困老年人的救助力度。老年人遇到特殊困难，当地人民政府要及时给予救济。要倡导和组织社会互助，积极开展扶老助困和志愿者服务等活动。

（十六）各级财政部门要加大对老龄事业的资金投入，主要用于老年社会保障、老年福利与服务设施建设以及老年教育、人才培训、科学研究等。要将老年福利事业经费纳入财政预算。在国家发行的彩票收益中，要有一定比例用于老龄事业的投入。

（十七）国家鼓励社会力量兴办老年福利服务设施。对社会力量投资兴办的福利性、非营利性的老年服务机构和有关捐赠，要实行减免税等优惠政策，具体办法由财政部、国家税务总局制定。

（十八）金融机构要充分发挥信贷支持作用，热情关注、积极支持社区老年服务设施、活动场所和福利设施的建设，按照信贷通则加大贷款支持力度。

（十九）地方各级人民政府在编制本地区土地利用年度计划实施方案时，应统筹安排社区老年服务设施、活动场所和福利设施建设用地，并按有关法律法规规定，采用行政划拨方式或优惠有偿方式供地。要采取有效措施，对新建老年服务设施的市政基础设施配套建设费酌情给予减免，降低征地和拆迁补偿费。

（二十）加强对老龄工作者队伍的建设，特别要加强对老龄工作干部的业务培训，提高老龄工作者自身素质，培养一支热爱老龄事业、全心全意为老年人服务的干部队伍。有条件的普通院校可开设老年学专业和社区服务类专业，培养从事老龄工作和社区工作的专门人才，加强社区干部队伍建设。

六、开展生动活泼的老年思想政治工作

（二十一）进一步加强和改进老年思想政治工作，认真研究解决老年群体中的各种思想问题。要坚持把马克思列宁主义、毛泽东思想特别是邓小平理论作为老年思想政治教育的重要内容，积极开展党的基本路线、政策、形势、民主与法制和科学文化知识的教育，使广大老年人树立正确的世界观、人生观和价值观，划清科学与迷信、文明与愚昧的界限，坚定对建设有中国特色社会主义的信念，增强对改革开放和现代化建设的信心，坚定地与以江泽民同志为核心的党中央在政治上、思想上保持一致。

（二十二）积极研究和探索新形势下加强和改进老年思想政治工作的新形式、新办法。要根据老年人的特点，把思想教育与开展健康有益的文化体育活动、解决思想问题与解决实际问题结合起来。坚持以理服人，以情感人，寓教于乐，把老年思想政治工作做实、做活、做深、做细，使广大老年人以丰富健康文明的生活方式安享晚年。要总结和推广新经验，树立典型，表彰先进，弘扬正气。

（二十三）充分发挥基层党组织在老年思想政治工作中的战斗堡垒作用，重视和发挥老年党员的政治优势和先锋模范作用。所有老年党员都要编入党的基层组织，参加党组织的活动。要建立社区老年人思想教育工作机制，切实做好老年思想政治工作，保证老年人自觉贯彻执行党的路线、方针、政策。

七、加强对老龄工作的领导

（二十四）老龄工作是党政工作的重要组成部分。各级党委和人民政府要统一思想，提高认识，加强领导，把老龄工作列入日常工作议程，及时研究解决工作中出现的新情况和新问题。

 （二十五）理顺和健全老龄工作体制。全国老龄工作在全国老龄工作委员会的领导下，民政部牵头，中央和国家机关各有关部门、群众团体共同参与。地方各级党委、人民政府要参照全国老龄工作委员会的设置，尽快建立健全本地区老龄工作议事协调机构，并在民政部门建立精干的办事机构，提供必要的工作经费。各地要充分发挥各有关部门和工会、共青团、妇联等群众团体及老龄组织的作用，共同做好老龄工作。

加强老龄工作，发展老龄事业，是党中央、国务院面向新世纪作出的重大决策。各级党委和人民政府要认真贯彻落实本决定精神，在以江泽民同志为核心的党中央领导下，高举邓小平理论伟大旗帜，努力开创我国老龄事业的新局面，为实现社会主义现代化建设的宏伟目标作出更大贡献。

**中组部、文化部、教育部、民政部、全国老龄工作委员会办公室关于做好老年教育工作的通知**

（文社图发[2001]22号）

各省、自治区、直辖市党委组织部、老干部局，政府文化厅（局）、教育厅（教委）、民政厅（局），老龄工作委员会办公室，中央和国家机关各部委人事教育部门、离退休干部工作部门：多年以来，各级党委组织部门、老干部工作部门和政府文化、教育、民政及老龄等部门积极兴办各类老年大学，努力探索老年教育事业发展的新路子，做了大量工作，取得了显著成绩。有效地提高了老年人的生命生活质量，促进了社会主义物质文明和精神文明建设。

根据《关于印发全国老龄工作委员会成员单位职责的**通知**》（全国老工委发[1999]4号）规定，今后文化部将“全面负责全国老年非学历教育工作，指导各级各类老年大学的工作”。为此，现就有关问题**通知**如下：

一、各级党委组织部门、老干部工作部门和政府文化、教育、民政部门及老龄工作部门要以邓小平理论为指针，深入学习、贯彻江泽民总书记“三个代表”的重要思想，从全局性、战略性的高度，充分认识老年教育工作的重要性和做好老年教育工作的紧迫性。遵循老年教育事业发展的规律，以“老有所教”、“老有所学”、“老有所乐”、“老有所为”为目标，推动老年教育事业的健康发展。

二、各级党委、政府和有关部门要进一步采取措施巩固老年教育事业取得的成果。文化行政部门要会同有关部门认真学习和借鉴各单位发展老年教育事业的成功经验，尽快制定老年教育事业发展规划和远景目标，进一步加强领导，科学指导，逐步规划老年教育事业的发展。

各级文化部门要抓紧时间对现有的老年大学、老年学校进行摸底调查，做好老年大学、老年学校的登记备案工作。各单位要给予大力支持和协助。

各级文化部门和文化事业单位要充分发挥现有文化设施的作用，依托省、市、县群艺馆、文化馆和乡镇文化站等群众文化设施，多渠道、多层次地发展老年教育事业，积极兴办新的老年大学。争取在较短的时间内实现“县县有老年大学”的目标，并逐步向社区、村镇延伸。培育和树立一批条件较好、质量较高、制度较全、颇具规模的规范化老年大学示范校。

三、各单位兴办的老年大学、老年学校，不改变现有的行政隶属关系，不改变现有的经费来源渠道，不改变现有的正确的办学方向，不改变现有的科学的办学模式。各办学单位要有计划、有步骤地提高现有老年大学、老年学校的数量和质量，扩大办学规模。加强与各级文化部门的沟通，加强研究，在办学形式和内容上大胆探索，使现有的老年大学、老年学校向更高水平发展。

四、鼓励社会各界积极兴办老年教育事业，逐步拓宽社会化办学的路子。继续倡导联合办学，民主办学。争取使全国现有的和新办的各级各类老年大学、老年学校基本做到有领导、有经费、有阵地、有队伍、有效益，在本世纪前10年建立健全具有中国特色的老年教育事业体系。

老年教育工作关系到社会的各个方面，需要全社会的共同关心和支持。各级文化部门要在当地党委和政府的统一领导下，与组织、教育、民政、老龄部门密切配合，动员全社会积极行动起来，同心协力，齐抓共建，积极开创老年教育事业的新局面，促进老年教育事业的全面繁荣。

中组部、文化部、教育部、民政部、全国老龄工作委员会办公室

                                                                                                         二OO一年六月二十二日

**中国老龄事业发展“十五”计划纲要**

(2001—2005年)

从新世纪开始，我国将进入全面建设小康社会，加快推进社会主义现代化的新阶段。为促进我国老龄事业的发展，根据《中华人民共和国老年人权益保障法》、《中共中央、国务院关于加强老龄工作的决定》(中发〔2000〕13号)和《中华人民共和国国民经济和社会发展第十个五年计划纲要》，结合我国人口老龄化的实际，制定《中国老龄事业发展“十五”计划纲要(2001—2005年)》(以下简称《纲要》)。

**一、背景**

改革开放以来，在党中央、国务院的领导下，我国老龄事业有了较快发展。1994年12月，国家计委、民政部等部门联合制定了《中国老龄工作七年发展纲要(1994—2000年)》，1999年10月，党中央、国务院决定成立全国老龄工作委员会，2000年8月，党中央、国务院又下发了《关于加强老龄工作的决定》，有力地推动了我国老龄事业的发展。老年人的经济供养与医疗保障得到改善，老年福利、文化、教育、体育事业有了较快发展，老年人合法权益受到重视，敬老、养老、助老的社会氛围逐步形成，老年人生活质量明显提高，老龄组织健康发展，各级老龄工作委员会及其办事机构正在健全和加强。但是，我国老龄事业总体上仍滞后于人口老龄化的要求和社会经济的发展。社会保障制度不够完善，老年设施、产品与服务短缺，老年精神文化生活不够丰富，侵犯老年人合法权益的现象时有发生，适应社会主义市场经济体制的老龄事业发展机制尚待建立和完善。

世纪之交，我国60岁以上老年人口超过总人口的10%，人口年龄结构开始进入老龄化阶段。今后一个时期，我国老年人口还将以较快速度增长，到2015年60岁以上人口将超过2亿，约占总人口的14%。我国人口老龄化与先期进入人口老年型的国家相比，具有老龄化发展快、老年人口数量大、地区之间不平衡、超前于社会经济发展等特点。老年人对经济供养、医疗保健、生活照料和精神文化等方面需求的日益增长，必将给国民经济和社会发展带来巨大的挑战。解决老龄问题，满足老年人不断增长的物质和文化需求，实现老龄事业与经济社会协调发展，促进社会公平和稳定，是社会主义现代化建设一项重要而紧迫的战略任务。

从新世纪开始，我国将全面实施社会主义现代化建设第三步发展战略，经济发展和综合国力的增强将为解决老龄问题创造良好的物质基础。同时，未来十几年，虽然老年赡养比开始上升，但少儿抚养比明显降低，社会总抚养比持续下降，也为做好21世纪人口老龄化高峰前的准备提供了有利条件。我们要抓住机遇，制定对策，有计划、有步骤地推动老龄事业持续健康发展。

**二、总目标和指导原则**

(一)总目标。

加快老龄事业发展步伐，重点解决老龄事业发展中的突出问题，落实“老有所养、老有所医、老有所教、老有所学、老有所为、老有所乐”，把老龄事业推向全面发展的新阶段。

——初步建立适应社会主义市场经济要求、体现城乡不同特点的城市和农村养老保障体系。

——建立以城市社区为基础的老年人管理与服务体系。

——进一步丰富老年人的精神文化生活，加强思想政治工作。

——切实维护老年人的合法权益。

——建立老龄事业的正常投入机制。

——健全老龄工作体系。

(二)指导原则。

以邓小平理论和江泽民同志“三个代表”的重要思想为指导，以提高老年人口的生活质量，实现经济和社会的可持续发展为宗旨发展老龄事业，并遵循以下原则：

1.坚持老龄事业与国民经济和社会发展相适应。把老龄事业纳入可持续发展战略，增加老龄事业投入，使其与经济社会发展水平和老年人口增长相适应。把解决现实老龄问题和应对人口老龄化高峰的长远准备结合起来，统筹兼顾，协调发展。

2.坚持老龄事业与社会主义市场经济体制相适应。发展老龄事业要着眼于体制创新，发挥政府的主导作用，运用市场机制，动员社会各方面力量，推动老年服务业走社会化、产业化道路。

3.坚持家庭养老与社会养老相结合。完善社会养老保障制度，加快发展老年服务事业，同时继续鼓励和支持家庭养老，倡导个人的自我养老准备，走政府、社会、家庭和个人相结合的养老保障道路。

4.坚持用法制和道德的力量营造发展老龄事业的社会环境。加强法制建设，建立健全法律约束机制；完善道德规范，重视舆论引导，增进代际和谐。

5.坚持从物质和精神两方面提高老年人的生活质量。在保障老年人生活的同时，注意丰富老年人的精神文化生活，重视老年人的价值，发挥老年人的作用，引导老年人自立自强，积极向上。

6.坚持因地制宜，分类指导，突出重点。重视农村老龄问题和农民的养老问题；解决好老少边穷地区老年人的生活困难问题；保障高龄老人、残疾老人、老年妇女、独居老人等特殊群体的基本生活和合法权益；把老龄工作的重心放在社区、基层。

**三、任务和措施**

(一)经济供养。

1.任务。

——初步建立政府、社会、家庭和个人相结合的经济供养体系，保障老年人基本生活。

——确保老年人生活水平随社会经济发展逐步提高。

2.措施。

——在城镇，要加快建立统一、规范、完善的养老保险体系，确保企业离退休人员基本养老金的按时足额发放，全面实行基本养老金的社会化发放。依法扩大基本养老保险覆盖面，鼓励发展个人储蓄性养老保险。进一步完善基本养老金的正常调整机制，随着经济发展和职工工资水平的提高，合理增加基本养老金，使离退休人员共享经济和社会发展成果。多渠道筹集社会保障基金，为应对人口老龄化高峰作好准备。

——在农村，要逐步建立和完善土地保障、家庭赡养和社会扶持相结合的农民养老保障体系。农民养老以家庭赡养为主，倡导赡养人之间签订“家庭赡养协议”；鼓励低龄健康老人提高自养能力；对无劳动能力、无生活来源、无赡养人和扶养人，或者赡养人和扶养人确无赡养能力或者扶养能力的老人继续完善以保吃、保穿、保住、保医、保葬为内容的“五保”供养制度，逐步提高供养水平；有条件的地方可实行对老年人的集体福利制度；根据情况逐步建立独生子女户和两女户的计划生育养老保障制度；注意探索和解决城镇化过程中老年人的养老保障问题。

——按照政府救济和社会互助相结合的原则，构建多层次、多元化、多项目的贫困老人救助体系。确保城市居民最低生活保障制度在城市老年人中的贯彻落实，并随社会经济发展相应提高最低生活保障标准。通过政府救济和社会互助，多渠道筹集资金，对特殊困难的老人实行临时性救助，大力倡导多种形式的扶老助困送温暖活动。

(二)医疗保健。

1.任务。

——努力满足老年人的基本医疗需求。

——初步建立以社区卫生服务为基础的老年医疗保健服务体系。

——做好健康教育和预防保健工作，提高老年人口健康水平。健康教育普及率城市达到80%，农村达到50%。老年人体育健身参与率达到40—50%。

2.措施。

——要完善和推进城镇职工基本医疗保险制度，按照规定，将符合条件的社区卫生服务机构纳入城镇职工基本医疗保险定点机构，服务项目纳入基本医疗保险支付范围，积极发展多种形式的补充医疗保险，逐步建立多层次的医疗保障体系。要探索多种形式的农村健康保障办法。逐步探索和建立城乡医疗救助制度，改善特困老年人的医疗条件。

——加强社区老年卫生工作。要充分利用城市现有卫生资源，大力发展社区老年卫生服务，以优质、便捷、经济为原则，为老年人提供预防、医疗、护理和康复等多种服务，逐步把老年人的基本健康问题大部分解决在社区。要完善县、乡、村三级医疗预防保健网，加快乡、村医疗卫生组织建设，努力改善老年人的医疗卫生条件。加强“老少边穷”地区老年人的医疗保健工作，逐步改善贫困老年人缺医少药的状况。

——重视健康教育和预防保健。在青少年中倡导科学、文明、健康的生活方式，降低老年期常见疾病的发病率和致残率；在老年人中普及卫生保健知识，增强自我保健能力；注意针对老年妇女的身心特点做好预防保健工作，努力提高健康预期寿命。逐步建立社区老年人口健康档案，加强健康教育和健康监测。重视老年期常见病、多发病的防治和康复研究，建立老年人口健康评价指标体系。

——为老年人参与体育健身创造条件。县(市、区)要普遍设立老年体育指导站(点)，加强对老年人体育健身活动的科学指导；城市社区和农村乡镇要因地制宜地开辟老年人健身场所；逐步建立老年人体质监测站，开展老年人体质监测指导工作。

——高龄老人和残疾老人就医，享受优先服务。

(三)照料服务。

1.任务。

——初步建成养老设施网络。城市养老机构床位数达到每千名老人10张，农村乡镇敬老院覆盖率达到90%。

——初步形成以社区为依托的老年照料服务体系，提供全方位、多层次的服务。

——建立社区为老服务的有效管理体制和服务队伍。

2.措施。

——国家在充分利用现有设施的基础上，增加对养老设施建设的投入，通过新建和改、扩建的办法，办好示范性的养老设施，同时制定优惠政策，吸引社会力量投资兴办以社区为基础、不同档次的养老服务设施，注意以需求为导向发展护理型养老设施。城市要有老年公寓、社会福利院、老年护理院，街道要有与老年人需求相适应的养老院或托老所，充分利用城市基层医疗机构的现有资源开展养老服务，逐步形成养老设施网络。乡(镇)敬老院要进一步加强设施建设，完善服务功能，服务范围扩大到周边地区的老年人。

——制订和完善各类老年设施的建设标准和设计规范。住宅、道路交通和公共设施建设应逐步满足老年人的特殊需求。

——大力发展社区老年照料服务。在社区建立综合性、多功能的服务站，依托社区老年服务设施，采取上门服务、定点服务等形式，开展看护照料、精神慰籍、家务帮助等服务项目。有条件的地区应逐步建立老年人紧急呼叫系统。充分利用家庭照料资源，积极探索支持家庭成员照料老年人的有效办法，逐步优化支持老年人居家养老的社会和社区环境。

——加强社区老年管理与服务人员的培训，提高职业道德和业务素质。建立管理人员定期培训制度；服务人员培训率达到60—80%，经过考核，持证上岗。重视整合和充分利用现有社区人力资源，大力发展社区志愿服务组织，积极鼓励在校学生以各种形式参加为老服务，形成专、兼职和志愿者相结合的为老服务队伍。

——适应退休人员逐步由社区管理的新形势，积极探索退休人员社区管理服务的新模式。逐步完善老年管理服务网络。充分发挥群众组织在社区老龄工作中的作用。

(四)精神文化生活。

1．任务。

——营造全社会尊重、理解、关心和帮助老年人的社会环境与舆论氛围。

——丰富老年人闲暇生活，提高老年人精神文化生活质量。

——大力发展老年教育，在校老年学员人数在现有基础上增加1倍。

——充分发挥老年人在社会生活中的积极作用。

2．措施。

——充分发挥广播、电视、报刊等大众传播媒体的宣传教育和引导作用，采取多种形式，加强老龄宣传工作，增强全社会的老龄意识；要把弘扬敬老、养老、助老美德作为社会主义精神文明建设的重要内容，将敬老教育内容列入中小学教材；地方各级人民政府和有关部门要对老龄事业中涌现出的先进单位、家庭和个人给予表彰和奖励。

——加强老年活动设施建设。大中城市要逐步建立设施完备、功能齐全、综合性的老年活动中心，县(市、区)要建立老年文化活动中心，乡(镇)、街道要设立老年活动站，有条件的村委会、居委会要开设老年活动室。各地要在现有或新建的公益性文化设施中开辟老年人活动场所，同时鼓励部门和单位管辖的文化活动场所向老年人开放。公园、图书馆、文化馆(站)、体育馆、博物馆等公共文化活动设施要优惠向老年人开放。

——加强老年文化建设。广播电台、电视台要办好老年文化专题节目；文学、影视、戏剧界要积极创作老年人喜闻乐见的优秀作品；新闻出版部门要重视办好老年报刊，出版面向老年人的图书、音像、电子出版物。大力开展适合老年人特点的社区文体活动，对各类专业性老年群众文体组织给予支持和引导，有计划地组织老年文艺汇演、书画展览等活动。

——建立老年教育网络。各级政府要合理安排对老年教育的投入，同时动员社会力量，因地制宜办好老年教育。省(自治区、直辖市)要建立1～2所示范性老年大学，地级市和有条件的县(市、区)要设立老年大学，乡(镇)、街道以及有条件的村委会、居委会要有老年学校、图书阅览室等学习场所；各种成人教育院校应积极开设老年班；发展老年电视大学、老年网上学校等多种形式的老年教育。重视做好老年教育教材的编写工作。制定并公布全国老年教育条例，加强老年教育的规范化管理。

——开展适合老年人特点的教育活动，帮助老年人增长知识，陶冶情操，倡导科学、文明、健康的生活方式。要特别重视加强老年思想政治工作，在城市，要通过举办各种讲座、学习班、报告会，就近就地开展多种形式、生动活泼的老年思想教育活动，帮助老年人树立科学的世界观和积极健康的人生观、价值观；在农村，要积极组织引导老年人学习科学文化知识和农业技术，崇尚科学，破除迷信，移风易俗。重视发挥基层党支部在老年思想教育工作中的作用和老党员的先锋模范作用。

——鼓励老年人继续参与社会发展。根据社会需要和自愿量力的原则，创造条件，积极发挥老年人在两个文明建设中的作用。在城镇，要重视老年人才资源的开发和利用，引导老年人从事教育、科研、咨询以及维护社会治安、社区服务等社会公益活动；在农村，鼓励健康老人从事种植、养殖和加工业。支持老年人自助互助。注意充分发挥老年人在基层民主政治建设中的作用。

(五)权益保障。

1．任务。

——加强立法、执法工作，逐步形成维护老年人合法权益的法律保障体系。

——加强普法教育工作。城市普法教育普及率不低于80%，农村不低于60%。

2．措施。

——抓紧制订中华人民共和国老年人权益保障法实施细则以及有关老年人社会福利等方面的法律法规，根据实际情况，制订地方行政法规和部门规章。

——加大执法力度，依法处理和打击侵犯老年人合法权益的不法行为。逐步建立较为完善的老年法律服务、法律援助组织网络，基层法律服务和法律援助机构要设立老年人合法权益维护岗，社区要设立法律服务网点，保证老年人能够就地、就近、及时得到有效的法律服务。对无力支付法律服务费用的老年人，按有关规定提供法律援助，给予缓交、减交或免交的优待。

——加大《中华人民共和国老年人权益保障法》的宣传力度，强化全社会维护老年人权益的法制观念。帮助老年人学法、懂法、守法，提高法律意识，依法维护自身的合法权益。

——加强法律监督。各级老龄工作机构要切实履行职责，督促和协调各部门依法行政，积极配合和协助司法行政部门，做好维护老年人合法权益的工作。有关部门要对《中华人民共和国老年人权益保障法》的贯彻执行情况定期进行检查。

**四、实施《纲要》的保证**

(一)加强组织建设。

地方各级人民政府要加强对老龄工作的领导，把老龄工作列入日常工作议程，建立和完善各级老龄工作委员会及其办事机构，明确任务和职责，核定编制，配备干部，保证必要的工作经费和工作条件。加强老龄工作队伍建设，采取多种形式，对老龄工作干部进行有计划的培训，提高政治和业务素质。

(二)加大经费投入。

将老龄事业纳入国民经济和社会发展总体规划，根据国民经济发展水平和老年人口规模，从实际需要和可能出发，增加对老龄事业经费的投入，用于补充基本养老保险基金、城市居民最低生活保障资金、老年服务设施建设、科学研究和老年教育等方面，逐步形成制度化的财力投入机制。在国家发行的彩票收益中要明确一定比例用于老龄事业的发展。同时，要调动社会各方面的积极性，建立多元化的老龄事业投入机制。

(三)加快体制创新。

大力推进老年福利事业社会化、产业化和法制化进程。在坚持政府主导，加大对老年福利事业投入的同时，要充分运用市场机制，动员社会各方面力量广泛参与。计划、财政、工商、税务、物价、国土、建设和民政等部门要制定优惠扶持政策，鼓励社会团体、民办非企业单位、私营企业和国内外人士投资老龄事业，发展老年产业，满足不断增长的老年群体对设施、产品与服务的需求。

政府办的老年福利事业也要引入市场机制，并积极探索公办民营等委托运作形式。同时，要改革内部管理体制，提高工作效率和服务质量。要对社会办的老年福利事业进行必要的调控、规范、指导和监督，逐步形成政府引导和宏观管理、社会中介组织经办运作、福利机构自主经营的管理体制。

(四)重视科学研究和人才培养。

加强老龄问题基础理论和应用研究，密切注视人口老龄化发展趋势及其对社会经济的影响，关注老年人口状况，建立老龄事业统计指标体系，形成统计工作制度，为科学规划和科学决策服务。加强对外交流与合作，借鉴国际经验，不断提高老龄科研水平。

加强人才培养，为老龄事业培养各类专门人才。在有条件的综合性大学开设社会老年学专业或课程，在医学和护理院校增加老年医学、老年护理学教学内容。有条件的院校可设立硕士、博士学位，培养老年学高级专门人才。大力开展老年教育和老年医学研究。

(五)建立督查和评估机制。

《纲要》由全国老龄工作委员会负责协调并督促实施。各地区、各部门要按照《纲要》的要求，根据各地实际和职责范围，制定本地区、本部门的具体实施方案。要对做出突出成绩的地方和部门给予表彰，形成老龄事业发展的激励机制。全国老龄工作委员会办公室要会同有关部门，组织力量定期对《纲要》的实施情况进行检查和评估，并在2005年对《纲要》的执行情况进行全面评审。

**关于积极推进企业退休人员社会化**

**管理服务工作的意见**

（2003年6月6日）

近年来，各地按照党中央、国务院的部署，在基本实现企业退休人员养老金由社会服务机构发放的基础上，加大推进社会化管理服务工作的力度，取得初步成效。这对深化企业改革和经济结构调整，保障企业退休人员基本生活，维护社会稳定发挥了积极作用。但由于这项工作目前尚处于起步阶段，一些部门和地区还存在思想认识不到位、工作条件不配套、职责分工不明确、管理服务不规范等问题。为积极推进企业退休人员社会化管理服务工作，现提出如下意见。

**一、提高思想认识，切实加强领导**

企业退休人员实行社会化管理服务，是建立独立于企业事业单位之外的社会保障体系的重要内容，是贯彻落实“三个代表”重要思想和十六大精神的具体体现，也是党中央、国务院在社会保障方面提出的一项重要任务。按照党的十五届四中全会通过的《中共中央关于国有企业改革和发展若干重大问题的决定》精神，国务院《关于完善城镇社会保障体系的试点方案》(国发〔2000〕42号)和《中共中央、国务院关于进一步做好下岗失业人员再就业工作的通知》(中发〔2002〕12号)对做好企业退休人员社会化管理服务工作提出了明确要求。广大企业退休人员长期以来为我国社会主义建设和国家发展作出了重要贡献，他们的晚年生活应当通过实行社会化管理服务得到更加充分的保障。同时，积极推进这项工作，对于深化国有企业改革、建立现代企业制度，对于更好地维护社会稳定，都具有十分重要的意义。各级党委和政府要充分认识做好这项工作的重要性和紧迫性，将其摆上重要议事日程，切实加强领导，精心组织，统筹规划，保证工作的顺利推进。

各地区要按照党中央、国务院提出的完善社会保障体系的有关目标和要求，以深化企业改革、维护社会稳定和不断提高企业退休人员生活质量为宗旨，因地制宜，分类指导，积极探索推进社会化管理服务工作的有效途径和方法，加大工作力度，逐步完善和规范管理服务办法。要充分利用城市社区资源，努力实现企业退休人员老有所养、老有所医、老有所教、老有所学、老有所为、老有所乐，使他们共享经济和社会发展的成果。

**二、企业退休人员社会化管理服务的主要内容**

企业退休人员社会化管理服务是指职工办理退休手续后，其管理服务工作与原企业分离，养老金实行社会化发放，人员移交城市街道和社区实行属地管理，由社区服务组织提供相应的管理服务。街道和社区的社会化管理服务工作主要包括：配合社会保险经办机构做好确保养老金按时足额发放工作，保障企业退休人员的基本生活；为企业退休人员提供社会保险政策咨询和各项查询服务；跟踪了解企业退休人员生存状况，协助社会保险经办机构进行领取养老金资格认证；帮助死亡企业退休人员的家属申请丧葬补助金和遗属津贴；集中管理企业退休人员的人事档案；组织企业退休人员中的党员经常开展组织活动，加强企业退休人员的思想政治工作；建立企业退休人员健康档案，有计划地开展健康教育、疾病预防控制和保健工作，提供方便的医疗、护理和康复服务；组织企业退休人员开展文化体育健身活动，指导和帮助他们通过各种形式的社会公益活动发挥余热，开展自我管理和互助服务。

企业退休人员中由中央管理的领导干部的移交、管理问题，另行规定。由县(市)以上各级党委管理的企业退休领导干部，在纳入街道和社区管理时，人事档案暂不移交，街道和社区可先建立这些退休人员基本情况的信息库。

**三、企业退休人员社会化管理服务的形式**

社会化管理服务的基本形式是将企业退休人员直接纳入街道和社区进行管理与服务。大中城市和其他经济比较发达、社区建设比较规范的地区，应主要采取这种形式。中央下放到地方管理的企业的退休人员，原则上应纳入其常年居住地或户口所在地的街道、社区实行属地管理，其养老保险关系和基本养老金的社会化发放工作，仍由省级社会保险经办机构负责管理。

由于各地城市社区建设发展不平衡，部分地区在一定时期内还可以结合实际情况，采取一些现实可行的管理服务形式。在远离城市的独立工矿区和企业退休人员居住比较集中的企业生活区，可以委托企业主管单位或企业，确定或设立企业退休人员管理服务机构，对退休人员实行管理服务。劳动保障部门要通过统一机构名称、统一规章制度和工作职责、对工作人员进行培训考核等方式，加强对这类管理服务机构的指导和规范。有条件的地区可以将企业原有的退休人员管理服务机构、人员和设施一并移交当地政府，对企业退休人员实行集中统一管理。在社区组织不够健全、企业退休人员居住比较分散的县，可以由社会保险经办机构直接对企业退休人员进行管理服务。部分地区经当地政府确定，由有关部门、单位建立的企业退休人员管理服务机构应充分利用现有的组织、人员及活动场所，继续开展社会化管理服务工作。随着企业办社会职能的逐步移交和当地社区建设的发展，这几种管理服务形式应逐步过渡到由街道和社区进行管理服务的基本形式。

**四、加强党组织建设和思想政治工作**

要加强街道、社区和企业党组织建设，做好企业退休人员党组织关系的接转工作，开展党的组织活动。尚未建立党组织的社区，要加快党组织建设的步伐，努力实现“一社区一支部(总支、党委)”的目标。企业退休人员中党员的组织关系要转入居住地党组织。企业退休人员中的党员，其居住地与户口所在地分离的，应将组织关系转入居住地党组织；又被原单位返聘的，组织关系继续留在原单位；受聘于其他单位或外出务工经商半年以上的，组织关系转入所去地区或单位的党组织。街道和社区党组织要认真抓好这项工作，及时将企业退休人员中的党员编入基层党支部，切实加强对他们的教育管理。要及时组织企业退休人员中的党员学习党的路线方针政策和国家的法律法规，履行党员义务，发挥先锋模范作用。对于转到街道和社区管理的企业退休领导干部，街道党(工)委和上级党组织应积极创造条件，保证这些退休人员能够按原来的职务级别阅读文件，参加相关的会议和活动，确保他们的政治待遇不受影响。

在推进社会化管理服务的过程中，要认真做好企业退休人员的思想政治工作。有关部门和街道、社区要通过多种形式广泛宣传企业退休人员实行社会化管理服务的有关政策、措施，创造良好的社会舆论环境。要把解决思想问题与解决实际困难结合起来，增强思想政治工作的实效，不断提高管理服务水平，消除企业和退休人员的顾虑，争取社会各方面的充分理解和广泛支持。

**五、尽快落实社会化管理服务的工作条件**

各地区要认真贯彻落实《中共中央、国务院关于进一步做好下岗失业人员再就业工作的通知》(中发〔2002〕12号)精神，加强街道、社区的劳动和社会保障工作。街道和工作任务重的乡镇可设立或确定负责劳动保障事务的机构，具体由各地党委、政府根据实际情况决定。街道劳动保障机构的人员编制由各地劳动保障部门提出方案，商同级编制部门确定。街道劳动保障机构的人员和工作经费，以及社区劳动保障工作经费由财政安排。要采取措施统筹解决开展社会化管理服务的场所和设施等方面的问题，满足企业退休人员档案管理、政策查询、工作人员办公和组织开展文体活动等方面的需要。

**六、继续落实企业在一定时期应承担的责任**

企业应加强与退休人员所在街道劳动保障机构联系，密切配合，共同做好企业退休人员的移交和社会化管理服务工作，及时解决他们的生活和思想问题。移交的人事档案要做到材料齐全、完好。企业退休人员的统筹项目外养老金，由企业继续按有关政策发放。尚未参加基本养老保险和基本医疗保险的企业退休人员的养老金和医疗费，继续由原渠道支付；企业退休人员居住的企业住房，尚未实行房改的，管理和维修工作仍由企业负责。企业不得以社会化管理为由随意减少退休人员的福利待遇。企业现有的用于退休人员活动的场所、设施，要继续发挥作用，并向社会开放。

**七、加强协调配合，共同做好企业退休人员社会化管理服务工作**

企业退休人员社会化管理服务工作内容多，涉及面广，各地要在党委、政府的统一领导下，动员社会各有关方面的力量，共同把这项工作做好。各级劳动保障部门及其社会保险经办机构要加强对街道(乡镇)、社区劳动保障工作的指导，积极推进医疗保险制度改革，及时将符合条件的社区卫生服务机构纳入城镇职工基本医疗保险定点医疗机构的范围，在医药费结算方式上对社区内的企业退休人员予以适当照顾并提供方便。组织部门要加强街道和社区党建工作，指导街道和社区党组织开展经常性的组织活动，加强对企业退休人员中党员的教育管理。发展改革部门要把社区公共服务体系建设纳入当地国民经济和社会发展计划，加快发展社区服务业，加强社区公共服务设施建设。民政部门要加强社区建设，指导社区服务坚持产业化、社会化发展方向，加快社区老年服务设施和服务网络建设，将有特殊生活困难的企业退休人员纳入社会扶助范围，及时提供公益性养老服务，及时向符合享受低保条件的企业退休人员家庭提供最低生活保障。财政部门在编制预算时，要统筹考虑企业退休人员社会化管理服务工作所需经费。卫生部门要加快社区卫生服务网络建设，为企业退休人员就近医疗提供方便。文化、体育部门要加快社区文体设施建设，组织企业退休人员开展丰富多彩的文化体育健身活动。工会、共青团、妇联、老龄等组织和机构要充分利用各自的管理服务网络，发挥自身优势，积极组织和指导社会志愿者队伍和其他社会公益组织，为企业退休人员特别是为高龄、孤寡、病残等生活困难的退休人员提供义务服务，并在维护企业退休人员合法权益方面发挥积极作用。

各省、自治区、直辖市要根据企业退休人员社会化管理服务目标和任务，抓紧制定切实可行的工作方案，于2003年7月底前报劳动和社会保障部备案，并尽快组织实施。

**《中央组织部、中央宣传部、中央统战部、人事部、科技部、劳动保障部、解放军总政治部、中国科协<关于进一步发挥离退休专业技术人员作用的意见>》的通知
（中办发[2005]9号）**

  我国广大离退休专业技术人员，长期奋斗在教育、科研、文化、卫生和工农业生产等各个领域，积累了丰富的实践经验，具有较高的专业技术水平，为国家的科技进步、经济社会发展作出了重要贡献，是党和国家的宝贵财富。在全面建设小康社会、加快推进社会主义现代化的新时期，继续发挥好离退休专业技术人员特别是老专家的作用，对于实施人才强国战略，促进人才队伍建设和构建和谐社会，具有重要意义。为贯彻落实〈中共中央、国务院关于进一步加强人才工作的决定〉(中发[2003]16号)精神，进一步发挥离退休专业技术人员的作用，现就做好有关工作提出如下意见。

  一、发挥离退休专业技术人员作用工作的总体要求。要以邓小平理论和“三个代表”重要思想为指导，贯彻尊重劳动、尊重知识、尊重人才、尊重创造的方针，按照政府引导支持、市场主导配置、单位按需聘请、个人自愿量力的原则，坚持社会需求和本人志趣、专业特长相结合，进一步完善政策措施，提高服务水平，保障合法权益，营造良好环境，使离退休专业技术人员特别是老专家在保持身心健康、安度晚年的同时，继续为全面建设小康社会贡献经验、才智和力量。

  二、积极支持离退休专业技术人员发挥作用。各级党委、政府和有关部门要通过多种形式，支持离退休专业技术人员特别是老专家进一步发挥在经济建设和科技进步中的服务和推动作用，发挥在培养教育下一代中的示范和教育作用。在重大工程立项、重要政策制定等方面组织专家咨询时，可聘请具有较高专业水平和社会声望的离退休专家参加决策咨询，充分听取他们的意见和建议。根据科技、经济和社会发展需要，可组织离退休专业技术人员参与教育培训、技术咨询、科技扶贫等活动，根据工作需要，可采取专项活动聘请、项目聘请、短期聘请等多种方式，聘请离退休专业技术人员特别是老专家从事青少年教育、传播科学文化知识、咨询服务、医疗卫生、科技开发应用等符合离退休专业技术人员特点的工作。支持离退休专业技术人员对青少年开展爱国主义、集体主义、社会主义和中华民族精神教育，进行科学知识普及。支持他们从事讲学、翻译、指导研究、专家门诊、咨询服务等专业技术活动。支持他们总结自己的实践经验，通过著书立说、培训指导等多种形式，培养青年人才。
  要根据市场需求和离退休专业技术人员的志愿，积极搭建服务平台，开拓离退休专业技术人员发挥作用的渠道。5-2 各类人才市场、人才中介机构应积极把离退休专业技术人员纳入服务范围。5-3

政府所属的人才交流中心、专家服务机构要通过设立专门的离退休专业技术人员服务窗口，举办专项的离退休专业技术人才和项目交流活动，开设老专家电话咨询服务热线等多种方式，主动为离退休专业技术人员发挥作用做好服务。

建立离退休专家信息数据库和离退休专业技术人员信息网络，定期举办网上离退休专业技术人才交流活动，为他们发挥作用提供信息平台。
  三、努力为离退休专业技术人员发挥作用提供必要的条件。凡符合条件的离退休专业技术人员可以参加专业技术人员职业资格考试，考试合格取得证书者按照规定登记注册。符合条件的离退休专业技术人员可以受聘作为项目组成员，参与申请和承担国家科技计划项目。离退休专业技术人员在科研成果评审、著作出版等方面，与在职人员一视同仁。科技成果符合国家科技奖励标准的，可按规定程序申报。离退休专业技术人员在从事科研、著书等活动时，原单位应允许他们借阅图书资料。离退休专业技术人员为聘用单位服务，需要使用原单位设备、器材以及技术资料、图纸等时，可与原单位协商后使用。
    离退休专业技术人员一般不再出国执行公务。如受聘后确需出国执行公务的，要按有关规定征得原单位同意并办理政审手续后，由派遣单位办理出国手续，有关费用由派遣单位负责。离退休专业技术人员应国(境)外机构和组织邀请，因私出国(境)讲学或参加学术交流，按照《中国人民共和国公民出境入境管理法》的有关规定办理，原单位应给予必要的协助。
  四、切实维护离退休专业技术人员的合法权益。各单位聘请离退休专业技术人员要按照平等协商、报酬合理的原则，通过合同方式明确双方的权利和义务，保障双方的合法权益。应聘期间，离退休专业技术人员继续享受原离退休费和生活[福利待遇](http://www.66law.cn/topic2010/福利待遇/)。离退休专业技术人员按照国家有关法律规定享有其科研成果转化的收益。离退休专业技术人员在应聘期间取得的报酬和科研成果转化收益应依法纳税。
  离退休专业技术人员受聘工作期间，因工作发生职业伤害的，应由聘用单位参照[工伤保险](http://www.66law.cn/topics/gsbx/default.aspx)的相关待遇标准妥善处理；因工作发生职业伤害与聘用单位发生争议的，可通过民事诉讼处理；与聘用单位之间因履行聘用合同发生争议的，可通过人事或劳动争议仲裁渠道解决。有条件的聘用单位在符合有关规定的情况下，可为聘请的离退休专业技术人员购买聘期内的[人身意外伤害保险](http://www.66law.cn/topic2010/rsywshbx/)。各聘用单位要关心离退休专业技术人员的身体健康，从工作需要和他们的实际情况出发，聘请他们从事力所能及的工作。
  五、高度重视发挥离退休专业技术人员社团组织的作用。要充分发挥中国老科技工作者协会、中国老教授协会等社团组织团结和凝聚离退休专业技术人员的桥梁纽带作用。鼓励和支持这些社团组织围绕经济社会发展的需要，发挥专业特长和优势，组织、推荐离退休专业技术人员特别是老专家继续发挥作用。政府开展的有关人才培养、项目开发、服务咨询等活动，可邀请离退休专业技术人员社团组织参加或承办。要加强与他们的联系，倾听他们的意见和建议。鼓励企事业单位和个人对这些社团组织开展发挥离退休专业技术人员作用的工作提供资金支持。
  广大离退休专业技术人员要模范地执行党的路线方针政策，遵守国家的[法律法规](http://laws.66law.cn)，大力宣传和弘扬科学精神，恪守职业道德，维护原单位和聘用单位的技术、经济权益，保守工作秘密。要大力发扬党的优良传统和作风，传承中华民族的优秀文化，在教育和引导青少年、中青年专业技术人员发扬优良传统、培育科学精神等方面，充分发挥示范、表率作用。
  六、大力加强对发挥离退休专业技术人员作用的领导。发挥离退休专业技术人员作用工作政策性强、涉及面广，各级党委、政府和有关部门要从实施人才强国战略的高度，重视发挥好离退休专业技术人员特别是老专家的作用，并努力在全社会营造重视、关心、支持离退休专业技术人员发挥作用的良好环境，使他们继续为全面建设小康社会作出贡献。要把离退休专业技术人才资源的开发纳入到人才队伍建设的整体规划之中，积极探索新形势下离退休专业技术人才资源开发的新思路、新机制，完善政策措施，创新服务方式，做好引导支持工作。由人事部牵头，中央组织部、中央宣传部、中央统战部、科技部、教育部、财政部、劳动保障部、解放军总政治部、中国科协、中国老科技工作者协会、中国老教授协会共同建立离退休专业技术人员发挥作用联席会议制度，负责沟通工作情况，研究政策建议，加强协调协作，在支持离退休专业技术人员发挥作用方面形成合力。

**中国老龄事业发展“十一五”规划**

　　为了加快发展老龄事业，根据《中华人民共和国国民经济和社会发展第十一个五年规划纲要》、《中华人民共和国老年人权益保障法》和《中共中央国务院关于加强老龄工作的决定》，结合我国人口老龄化快速发展的趋势，制定《中国老龄事业发展“十一五”规划》（以下简称《规划》）。

一、背景

　　（一）“十五”期间老龄事业的主要成就

　　《中国老龄事业发展十五计划纲要》实施以来，我国的老龄事业有了较快发展。养老保障体系逐步建立，养老保险覆盖面不断扩大，基本养老金按时足额发放，保障水平不断提高，企业离退休人员基本生活得到保障；基本医疗保险制度在城镇基本建立，新型农村合作医疗制度逐步推广，医疗救助制度开始试点；企业退休人员社会化管理服务工作进一步加强；老年福利、教育、文化、体育等事业有了较大发展，敬老、养老、助老的道德风尚进一步形成；老年人的合法权益得到有效维护，老年人精神文化生活日益丰富；地方老龄工作体制基本理顺，老龄工作有效开展；老龄领域的国际交流与合作不断扩大，为加快我国老龄事业的发展奠定了良好基础。

　　（二）“十一五”期间人口老龄化的形势

　　“十一五”期间，我国60岁及以上老年人口持续增长，到2010年将达到1.74亿，约占总人口的12.78％，其中，80岁以上高龄老年人将达到2132万，占老年人口总数的12.25％。和“十五”时期相比，老年人口增长速度明显加快，高龄化显著，农村老龄问题加剧，社会养老负担加重，养老保障问题突出，社区照料服务需求迅速增加，老龄问题的社会压力日益增大，对我国政治、经济、社会都将产生深刻影响。

　　（三）“十一五”期间是全面应对人口老龄化的关键时期

　　根据预测，我国人口老龄化高峰期将在2030年到来，从现在开始，我国应对人口老龄化的战略准备期只有25年。“十一五”期间，既是我国经济社会发展重要的战略机遇期，也是全面应对人口老龄化的关键时期。要未雨绸缪，抓住机遇，按照“五个统筹”的思想，协调处理好经济与社会、全局与局部、当前与长远等各个方面的关系，在大力促进经济发展的同时，加快发展老龄事业，认真解决人口老龄化带来的各种矛盾和问题，为应对人口老龄化挑战打下坚实基础。

　　二、指导思想、总体目标和基本原则

　　（一）指导思想

　　以邓小平理论和“三个代表”重要思想为指导，坚持以人为本，落实科学发展观，深入贯彻“党政主导，社会参与，全民关怀”的老龄工作方针，从我国的基本国情出发，加快体制创新，制定应对人口老龄化的政策措施，努力完善社会保障制度，维护老年人的合法权益，充分发挥政府、社会、家庭和个人的作用，提高老年人的生活质量，按照“五个统筹”的要求，促进老龄事业和经济社会的协调发展。

　　（二）总体目标

　　“十一五”期间，继续增加财政对社会保障的投入，多渠道筹措老年社会保障基金，合理确定保障标准和方式，逐步建立广泛覆盖、持续发展，与经济社会相适应，与其他保障制度相衔接的老年社会保障体系。基本建立相对完善的老龄政策法规体系。健全与人口老龄化相适应、协调高效的老龄工作体制。积极推进方便老年人生活的基础设施建设，建立健全适应家庭养老和社会养老相结合的为老服务网络和满足老年人特殊需求的老年用品市场，进一步营造敬老、养老、助老和代际和谐的良好社会氛围，为实现“老有所养、老有所医、老有所教、老有所为、老有所学、老有所乐”的目标创造更为有利的社会条件。

　　（三）基本原则

　　1、坚持科学发展观，按照“五个统筹”的要求，兼顾当前与长远，把解决当前老龄问题和应对人口老龄化挑战结合起来，促进老龄事业与经济社会协调发展。

　　2、坚持以满足老年人的物质文化生活需求为出发点，完善养老、医疗等社会保障制度，提高老年人生活质量。

　　3、坚持国家、社会、家庭和个人相结合，大力发展为老服务体系，走中国特色的为老服务之路。

　　4、坚持统筹规划，充分考虑不同地区经济社会发展的差异，统筹兼顾，制定相应的政策措施，分类指导，发挥地区优势，稳步推进城市老龄工作，切实加强农村老龄工作，推动老龄事业协调发展。

　　三、老年社会保障

　　（一）养老保障

　　在城市，继续完善企业职工基本养老保险制度，以非公有制经济组织和城镇个体工商户、灵活就业人员为重点，扩大养老保险覆盖面，加强养老保险基金征缴工作，继续调整财政支出机构，增加各级财政对养老保险基金的投入，建立可靠、稳定的资金筹措机制，逐步提高统筹层次，增强统筹调剂的能力。进一步加强企业退休人员社会化管理服务工作，建立基本养老金正常调整机制，确保企业离退休人员基本养老金按时足额发放，保障他们的基本生活。积极推进机关事业单位养老保险制度改革。

　　在农村，探索建立与农村经济社会发展水平相适应、与其他保障措施相配套的农村社会养老保险制度。加快完善农村部分计划生育家庭奖励扶助制度。研究解决好社会主义新农村建设过程中老年人的养老保障问题，发挥土地养老的保障作用。推行签订家庭赡养协议书，明确赡养责任，巩固家庭养老保障功能。

　　（二）医疗保障

　　扩大老年人医疗保险覆盖范围，健全多层次的医疗保障体系。在城市，进一步完善城镇医疗保障制度，合理确定定点医疗机构范围，完善医疗服务管理，方便老年人就医。鼓励、支持医疗机构实行老年人挂号、就诊、取药、住院等优先服务。建立健全以社区卫生服务为基础的老年医疗保健服务体系，加强社区老年卫生工作，增加服务项目，改进服务措施，为老年人提供预防、医疗、护理和康复等多种服务。利用学校教育、岗位培训、继续教育等形式，加强社区全科医生和社区护士队伍建设。重视老年常见病、多发病的防治和康复研究，加强老年人疾病预防、保健知识教育，使老年人的健康教育普及率达到85%。

　　在农村，加强以乡镇卫生院为重点的农村卫生基础设施建设，健全农村卫生服务体系。继续推进新型农村合作医疗制度建设，扩大合作医疗试点范围。通过多种渠道努力改善老年人医疗卫生条件，使老年人的健康教育普及率达到55%。

　　建立和完善城乡医疗救助制度。帮助贫困老年人参加新型农村合作医疗，着力解决好困难企业退休人员医疗保障问题，为无支付能力的老年人提供帮助。

　　（三）社会救助

　　加强对贫困老年人的生活救助。在城市，要将城市贫困老年人全部纳入最低生活保障范围，切实保障其基本生活。在农村，完善农村特困户生活救助制度，将不具备劳动能力的贫困老年人纳入救助范围，给予其定期定量生活救济，并逐步向最低生活保障制度过渡。落实《农村五保供养工作条例》，把符合五保供养条件的老年人全部纳入供养范围。继续开展“计划生育困难家庭救助活动”，探索建立独生子女家庭老年人扶助制度。

　　（四）社会福利

　　立足发展补缺型的老年社会福利事业，兼顾发展面向全体社会公众的普惠型的老年福利事业，以满足“三无”老人、低保老人、残疾老人、高龄老人和空巢老人的生活照料需求为出发点，在鼓励家庭成员为老年人提供服务的同时，加快建立以居家养老为基础、社区服务为依托、机构养老为补充的老年人社会福利服务体系。

　　制定鼓励和引导社会力量兴办各类老年福利服务设施的政策；建设一批以老年服务为主体的综合示范性社区福利服务中心；开展多层次、多种类、多方式的居家养老和集中养老服务，建立健全老年福利服务网络。

　　四、老龄事业基础设施建设

　　（一）公共服务建设

　　努力增加资金投入，建设好与老年人日常生活密切相关的文化、卫生、社区服务等公共设施（场所），并充分利用好现有设施（场所）为老年人服务，逐步实现为老服务社区化。公园、展览馆、博物馆及图书馆、文化馆、图书室等文化娱乐场所，要增加面向老年人的服务项目，并免费或者优惠对老年人开放。

　　（二）养老服务设施建设

　　加大老年福利服务设施建设的财政投入，鼓励社会资本进入老年福利服务设施建设领域，积极发展老年人社会福利事业。

　　在养老机构建设方面，“十一五”期间，各省、市、县（区）要根据当地实际情况，建设一批设施齐全、功能完善的养老服务机构。农村五保供养服务机构要实现集中供养率50%的目标，新增供养床位220万张，使生活不能自理的农村五保供养对象的生活得到有效照料；要新增城镇孤老集中供养床位80万张，有效缓解城镇孤老安置床位紧张局面；开展以生活不能自理和半自理老年人为对象的“爱心护理工程”试点和示范工作，在大中城市建设一批“爱心护理院”。

　　在社区为老服务设施建设方面，要考虑老年人的需求差异，建设一批不同类型、不同层次的福利服务设施，缓解城市街居和农村乡镇老年人福利服务设施严重匮乏的矛盾，为居家养老提供支持，为老年人活动提供场所。在城市，把老年服务设施纳入街道、社区服务体系，统筹规划、合理建设；在农村，充分利用现有闲置资源，加大农村养老设施的资金投入，以农村五保供养服务机构建设为依托，加强农村乡镇敬老院、老年活动中心和综合性老年福利服务中心建设，争取使其覆盖75%以上的乡镇。西部地区的县、中东部地区的乡镇争取至少建一所设施齐备、功能完备、服务到位的老年活动中心。

　　（三）住房和生活环境建设

　　制定和完善各类老年设施的建设标准和技术标准，完善老年住宅、老年公寓、养老院、护理院、托老所等养老设施的标准规范。制定城市和村镇老龄设施规划规范和养老设施建筑设计规范，逐步形成老年服务设施建设标准体系。

　　新建城市道路、公共建筑和养老场所要严格执行《城市道路和建筑物无障碍设计规范》，在规划、设计、施工、监理、验收等环节严格把关，新建城市道路和养老场所无障碍率达到100%。对已建成并投入使用的与老年人生活、工作密切相关的居住区、城市道路、公共建筑和养老场所，要制定改造计划，增补无障碍设施，到2010年养老场所无障碍改造率达到60%。省会以上大中型城市积极实施城市交通无障碍工程，加强道路改造，加快无障碍公共交通工具的研制开发工作，逐步提高公交设施无障碍的比例。

　　五、老龄产业

　　（一）政策扶持

　　发挥政府的指导和监督作用，把老龄产业纳入国民经济发展总体规划，列入国家扶持行业目录。制定引导老龄产业发展的税收、信贷、投资等政策，采取税收优惠、减免费用、信贷支持等措施，大力扶植尚在起步阶段的老龄产业，积极鼓励、引导和规范个体私营和外资等非公有资本参与老龄产业的发展。

　　（二）养老服务业

　　鼓励吸引社会力量投资兴办不同档次的养老服务机构。支持信息服务、管理咨询、人才培训等社会中介机构的发展，鼓励社会力量开展以社区为基础的养老服务，逐步形成为老年人提供生活照料、医疗保健、康复护理、家政服务、心理咨询、文化学习、体育健身、娱乐休闲等综合性的服务网络，为居家老人提供优质、便捷的服务。

　　（三）老年用品和老年服务产品

　　鼓励和扶持开发老年用品，引导企业生产满足老年人各种需求的门类齐全、品种多样、经济适用的老年用品；充分利用各地旅游资源，推出适合老年人特点的旅游线路和服务产品，方便老年人出游；拓展多种经济成分并存的老年卫生健康服务领域，为老年人提供预防、医疗、护理和康复保健等服务；积极开发适合老年人的金融、理财、保险等其他服务项目。

　　（四）老年消费

　　积极开发老年用品市场，鼓励支持老年用品专卖店和专卖柜台的发展，可采用老年用品博览等形式，促进流通，扩大销售。正确引导老年人的消费观念和消费行为，促进老年消费市场的繁荣与发展。

　　六、老年精神文化生活

　　（一）老年教育

　　各级政府要继续加大对老年教育的资金投入，同时动员社会力量，因地制宜地办好老年电视大学、老年网上学校，倡导社区办学。到2010年，老年大学和老年学校在现有基础上增加1万所。重视对老年农民的培训，把老年教育与老年人脱贫致富、维护权益、破除迷信和移风易俗结合起来，促进社会主义新农村建设。

　　（二）老年文化和体育

　　老年文化和体育要纳入全民文化和体育健身的发展规划。制定相关政策法规，鼓励和引导社会力量投入老年文化体育事业。广播电台、电视台要办好老年文化专题节目。文学、影视、戏剧界积极创作老年题材的优秀作品。新闻出版部门要办好老年报刊，出版面向老年人的图书、音像、电子出版物。文化部门要积极开展老年文艺活动，送文艺到基层，丰富社区、农村老年人的精神文化生活。各地要开展经常性的基层老年文体活动，有计划地组织老年文艺汇演、老年才艺展演和老年体育健身运动会。对在老年教育和老年文体活动中表现突出的先进单位和个人进行表彰。

　　七、老年人权益保障

　　（一）老年人权益法制建设

　　提出《中华人民共和国老年人权益保障法》的修订意见，制定《中华人民共和国老年人权益保障法》实施办法，加快老年社会保障的法制化进程。

　　（二）老年人法律服务与法律援助工作

　　“十一五”期间，政府要安排资金用于老年人法律援助和法律服务组织建设，鼓励社会各界关心、支持老年人法律援助工作。法律服务机构要优先为老年人提供及时、便利、高效的法律服务。继续做好农村家庭赡养协议书的签订工作。

　　（三）老年人权益宣传教育

　　加强《中华人民共和国老年人权益保障法》的宣传教育。推动《中华人民共和国老年人权益保障法》的宣传进社区、进农村，大力开展“敬老模范乡镇（村居）”的评选表彰活动，形成法制教育和道德教育相结合的机制，倡导敬老、养老、助老的社会文明之风。

　　（四）老年人权益维护

　　各级司法部门对侵害老年人合法权益的案件要做到及时受理、及时立案、及时审理、及时执行，比较突出的涉老案件要及时回访。酌情减免经济困难老年人的诉讼费用。各级公安部门特别是基层派出所对严重侵害老年人人身权和财产权的不法行为，要及时采取措施予以制止，妥善处理，构成犯罪的要依法惩处。

　　八、老年人社会参与

　　（一）老年人参与社会

　　研究制定相关政策，充分发挥老年人在构建社会主义和谐社会中的优势和特长，鼓励和支持老年人继续参与经济社会发展。发挥老年人在教育下一代中的示范和教育作用，鼓励老年人积极参与维护社会治安、社区建设等社会公益活动。在农村，鼓励低龄、健康老年人从事种植、养殖和加工业等经济活动。积极倡导和支持老年人广泛开展自助互助，努力探索实现“老有所为”的新形式。

　　（二）老年人才开发

　　积极开发老年人才市场，建立国家老年人才信息数据库和老年人才信息中心。凡符合条件的老年人，均可以参加专业技术人员职业资格考试，考试合格取得证书者按规定登记注册。符合条件的老年技能人才，可以参加职业技能鉴定，取得相应的职业资格证书。各地要把老年人才的开发和利用纳入人才市场建设的总体规划，根据市场需求和老年人的志愿，积极搭建老年人才服务平台，开拓老年人才参与社会的渠道。各类人才中介服务机构要根据需要举办多种形式的老年人才交流活动，积极为老年人提供服务。

　　九、实施《规划》的保证措施

　　（一）发挥政府的主导作用

　　各级政府要切实加强对老龄工作的领导，真正把老龄工作纳入议事日程。各级老龄工作委员会成员单位要充分发挥作用，按照职能，把老龄事业发展纳入本部门的工作规划。

　　（二）加大老龄事业资金投入

　　逐步加大老龄事业投入。各级财政要根据老龄事业发展需要，逐步增加对老年服务设施建设、老年文化教育、老龄科学研究和老年活动等方面的投入。在彩票公益金中要有一定比例用于老龄事业。充分发挥各级老年基金会和其他组织的作用，积极引导民营资本和国外资金投入老龄事业，逐步形成多元化的老龄事业投入机制。

　　（三）加强老龄工作机构和老年群众组织

　　机构编制要适应“十一五”期间老龄事业发展需要，加强各级老龄工作，充分发挥综合协调、督促检查、参谋助手作用。要把老龄工作干部培训纳入组织部门的培训计划，不断提高政治和业务素质。进一步加强基层老年群众组织建设，制定出台老年群众组织管理办法，规范老年群众组织的活动，充分发挥其在基层民主自治、社区建设和老龄工作中的作用。

　　（四）扩大为老服务队伍

　　加快老龄产业人才培养，特别是老龄产业管理人员、服务人员的培养。建立职级评聘体系，编制养老护理员国家职业标准和培训教材。根据国家职业标准，组织开展养老护理人员职业培训和鉴定工作。在有条件的普通高等学校和中等职业学校，在相关专业开设老年学、老年心理学和护理服务等课程。养老机构和社会培训机构要适应市场需求，培训养老护理员和服务员，落实持证上岗制度，逐步建立覆盖全国城乡的基层为老服务队伍。积极培育为老年人服务的非盈利性民间组织，大力发展志愿者队伍和社工队伍。

　　（五）加强老龄科研与对外交流合作

　　加大老龄科研经费投入，改革科研体制，培养高水平的老龄科研队伍。加强人口发展战略研究，制定人口发展中长期规划，统筹考虑解决人口年龄结构问题。在人员培训、学术研究和项目合作等领域拓宽对外交流与合作，借鉴国际应对人口老龄化的经验和做法，不断提高老龄工作水平。

　　（六）开展督查和评估

　　各地、各有关部门要按照本《规划》的要求，制定本地、本部门实施方案。全国老龄工作委员会办公室将会同有关部门对《规划》的实施情况进行督促检查，2010年对《规划》的执行情况进行全面评估。

**中华人民共和国国民经济和社会发展第十一个五年规划纲要**

**第三十八章　全面做好人口工作**

**第一节　稳定人口低生育水平**
　　坚持计划生育基本国策，稳定和完善现行生育政策，落实人口和计划生育工作目标责任制。建立独生子女死亡、伤残家庭扶助制度，完善基本服务项目免费制度。加强计划生育服务管理能力建设。完善以现居住地管理为主的流动人口计划生育服务管理体系。
　　**第二节　改善出生人口素质和结构**
　　普及优生优育知识，实施计划生育生殖健康促进计划，加大出生缺陷干预力度，鼓励婚前和孕前医学检查，预防和控制先天性感染、遗传性因素对出生人口健康的影响。采取综合措施有效治理出生人口性别比升高的问题。
　　**第三节　积极应对人口老龄化**
　　弘扬敬老风尚，营造老有所养、老有所乐、老有所为的社会氛围。积极发展老龄产业，增强全社会的养老服务功能，提高老年人生活质量，保障老年人权益。
　　实施爱心护理工程，加强养老服务、医疗救助、家庭病床等面向老年人的服务设施建设。
　　**第四节　保障妇女儿童权益**
　　落实男女平等基本国策，实施妇女发展纲要，保障妇女平等获得就学、就业、社会保障、婚姻财产和参与社会事务的权利，加强妇女卫生保健、扶贫减贫、劳动保护、法律援助等工作。坚持儿童优先原则，实施儿童发展纲要，依法保障儿童生存权、发展权、受保护权和参与权。改善儿童成长环境，促进儿童身心健康发展。完善孤残儿童手术康复、家庭寄养经费投入和艾滋孤儿救助机制。
　　**第五节　保障残疾人权益**
　　倡导和鼓励社会各界关心、支持和参与残疾人事业。推进无障碍设施建设，加强残疾人康复、贫困残疾人脱贫、残疾少年儿童义务教育、残疾人就业服务和社会保障等工作，创造残疾人平等参与社会生活的条件。

**关于加快发展养老服务业的意见**
全国老龄委办公室　发展改革委　教育部
民政部　劳动保障部　财政部　建设部
卫生部　人口计生委　税务总局

　　我国是人口大国，也是老年人口最多的国家。目前，我国60岁以上的老年人口已达1.43亿，占总人口的11%；预计2020年将达到2.4亿，占当时总人口的16%左右。养老服务业是为老年人提供生活照顾和护理服务，满足老年人特殊生活需求的服务行业。为促进养老服务业加快发展，现提出以下意见：
　　**一、充分认识加快发展养老服务业的重要意义**
　　老年人最值得尊敬和爱戴，也最需要关心和帮助。中华民族素有敬老、尊老的传统，随着经济社会的发展，人民生活水平的提高，社会生活方式的转变，老年群体在日常生活照顾、精神慰藉、心理支持、康复、护理、临终关怀、紧急救助等方面呈现出日益增长的需求。妥善处理人口老龄化问题，关心老年人的需求，加快发展养老服务业，是贯彻落实科学发展观、坚持以人为本的具体体现。认真解决老年人生活中的实际问题，有利于保持家庭关系稳定和睦，促进老年群体与其他群体和谐相处，这是构建社会主义和谐社会的重要内容，是社会文明进步的重要标志。同时，加快发展养老服务业，有利于促进相关行业发展，推动经济增长，提高全体人民生活质量和水平。各地区、各部门要充分认识发展养老服务业的重要意义，采取有效措施，推动养老服务业加快发展。
　　**二、突出工作重点，明确政策措施**
　　发展养老服务业要按照政策引导、政府扶持、社会兴办、市场推动的原则，逐步建立和完善以居家养老为基础、社区服务为依托、机构养老为补充的服务体系。要建立公开、平等、规范的养老服务业准入制度，积极支持以公建民营、民办公助、政府补贴、购买服务等多种方式兴办养老服务业，鼓励社会资金以独资、合资、合作、联营、参股等方式兴办养老服务业。
　　（一）进一步发展老年社会福利事业。地方各级人民政府要不断加大投入，建立健全老年福利服务体系，为城乡无劳动能力、无生活来源、无赡养人的老年人和生活困难的老年人提供无偿或低收费服务，保障他们的基本生活。要采取多种形式，鼓励和支持社会力量多形式、多渠道参与老年社会福利事业，增加老年福利服务设施数量，提高服务质量。
　　（二）大力发展社会养老服务机构。地方各级人民政府和有关部门要采取积极措施，大力支持发展各类社会养老服务机构。引导和支持社会力量兴建适宜老年人集中居住、生活、学习、娱乐、健身的老年公寓、养老院、敬老院，鼓励下岗、失业等人员创办家庭养老院、托老所，开展老年护理服务，为老年人创造良好的养老环境和条件。
　　（三）鼓励发展居家老人服务业务。要通过政策引导，鼓励社会资本投资兴办以老年人为对象的老年生活照顾、家政服务、心理咨询、康复服务、紧急救援等业务，向居住在社区（村镇）家庭的老年人提供养老服务，为他们营造良好的生活环境。
　　（四）支持发展老年护理、临终关怀服务业务。支持兴办老年护理、临终关怀性质的医疗机构，鼓励医疗机构开展老年护理、临终关怀服务。根据实际情况，对开展老年护理、临终关怀服务的机构按规定给予政策扶持。
　　（五）促进老年用品市场开发。制定鼓励措施，引导企业开发、生产老年人特殊用品，促进老年用品市场发展，满足老年人的多方面需求。
　　（六）加强教育培训，提高养老服务人员素质。加快培养老年医学、管理学、护理学、营养学以及心理学等方面的专业人才，提高社区及农村基层卫生技术人员的专业素质。有计划地在高等院校和中等职业学校增设养老服务相关专业和课程，改革教学内容和教学方法。加强岗位培训，提高养老服务从业人员职业道德、服务意识和业务技术水平。
　　**三、加强组织领导，认真落实责任**
　　各地区、各有关部门要加强领导，把加快发展养老服务业列入议事日程，纳入经济社会发展规划，明确工作目标，认真落实责任。要进一步强化政府公共服务职能，强化服务意识，改进服务方式，提高工作效率。要组织或促进制定建筑设施、卫生条件、质量标准、服务规范等养老服务行业标准，开展服务质量评估和服务行为监督，促进养老服务业向规范化、标准化发展。要鼓励建立养老服务行业中介组织，发挥其在行业自律、沟通企业与政府联系等方面的积极作用。各有关部门要加强协调，密切配合，认真解决养老服务业发展中的问题，促进养老服务业健康发展。
　　各地区、各有关部门要根据本意见精神，抓紧制定和完善促进养老服务业发展的具体措施。

**国务院办公厅关于印发人口发展
“十一五”和2020年规划的通知**

**人口发展“十一五”和2020年规划**

　老年人口规模日益增大，对经济社会发展将产生重大影响。受人口流动迁移影响，农村老龄问题更为严重。
　　出生人口性别比居高不下。据统计，全国出生人口性别比2000年为116.86，2005年为118.58，严重偏离正常范围。到2020年，预计20岁—45岁男性将比女性多3000万人左右。出生人口性别比过高、持续时间过长，将影响社会的稳定与和谐。
　　劳动年龄人口数量庞大且保持增长态势。据预测，到2016年，15岁—64岁劳动年龄人口将达到峰值10.1亿人，到2020年达10亿人左右；15岁—59岁劳动年龄人口将于2013年达到峰值9.32亿人，到2020年达9.30亿人左右。庞大的劳动年龄人口，使扩大就业和减少失业成为当前和今后长时期重大而艰巨的任务。
　　区域间人口发展差异明显。农村、中西部和贫困地区面临生育水平较高、人口自然增长率较高、文盲率高、孕产妇死亡率较高、平均受教育年限较短等问题，留守老人、儿童的照料和教育问题突出；城镇与农村人口社会保障水平不平衡。城乡间、地区间收入差距拉大的趋势没有明显改善，由此引发的各种深层次社会矛盾严重影响社会主义和谐社会的构建。
　 加大综合治理力度，有效遏制出生人口性别比升高势头。建立党政负责、部门配合、群众参与的标本兼治工作机制。加强综合治理的过程评估和责任考核。深入开展“关爱女孩行动”、“婚育新风进万家”活动。以消除性别歧视为重点，广泛宣传男女平等、少生优生等文明婚育观念，普及保护妇女儿童权益的法律法规知识。制定有利于女孩健康成长和妇女发展的社会经济政策，促进男女平等就业和共同参与社会经济活动。对农村计划生育女儿户给予奖励，在扶贫济困、慈善救助、贴息贷款、就业安排、项目扶持中对计划生育女儿户予以倾斜，推动“幸福工程”、“春蕾计划”等社会公益活动。鼓励男到女家落户，依法保护妇女的宅基地、房屋等继承权和土地承包权等权益。严禁非医学需要的胎儿性别鉴定和选择性别的人工终止妊娠。建立B超检查和人工终止妊娠登记、孕情检测、孕产过程管理等制度。完善执业资格认证和B超使用准入制度。对终止妊娠药品和促排卵药品实行严格的处方管理。运用法律手段，严厉打击非法实施胎儿性别鉴定和选择性别人工终止妊娠的行为，依法严惩溺、弃、残害女婴和拐卖、绑架妇女儿童的犯罪活动和歧视、虐待生育女婴的妇女等违法行为，保护妇女儿童合法权益。探索建立跨地区、跨部门的出生性别监控系统和选择性别终止妊娠综合监管制度。实施举报制度，加强社会监督。
　　建立健全养老保障体系和老年社会服务体系，积极应对人口老龄化。制定和落实老龄事业发展战略规划和政策，把逐步建立覆盖城乡居民的养老保障制度作为社会保障体系建设的重点，加强社区老年服务机构和基础设施建设，到2010年，初步构建以居家养老为基础、社区服务为依托、机构照料为补充的养老服务体系。加强农村乡镇敬老院、老年活动中心和综合性老年福利服务中心建设，争取使其覆盖75%以上的乡镇。在农村探索建立多种形式的计划生育家庭养老保险制度。有条件的地方，可建立政府、集体和社会共同参与的养老服务机构。对生活不能自理的农村计划生育家庭老年父母，按规定提供适当补助。对军烈属、鳏寡及其他有特殊困难的老年人，按规定给予养老救助。在城市逐步完善社会统筹与个人账户相结合的基本养老保险制度，构建多层次的城镇养老保障体系。积极发展适合老年人特点的知识和经验密集型服务业，为老年人提供力所能及参与社会的机会。提高养老服务机构在城市规划中的比重，发展社区老年活动场所和服务设施，制定优惠政策，鼓励社会开办各种类型的养老服务机构。发扬敬老、养老、助老的良好社会风尚，积极探索和实施“爱心护理”等工程。从老年预防保健入手，倡导健康生活方式，营造出行安全和起居方便的环境。探索建立老年服务志愿者、照顾储蓄、长期护理保险等社会化服务制度。大力弘扬子女赡养、家庭养老和邻里互助的传统美德。加强舆论监督，对拒绝赡养或虐待父母的行为，追究法律责任。大力发展老龄产业，建立满足特殊需求的老年用品和服务市场。倡导终生学习、终生保健，促进老年人身心健康。
　　（三）坚持教育优先发展，充分开发人力资源。
　　全面实施素质教育。坚持德育为先，把社会主义核心价值体系纳入国民教育全过程，切实加强和改进中小学思想道德建设和大学生思想政治教育。进一步深化教育教学改革，倡导和组织学生参加生产劳动和社会实践活动，加强对学生创新精神和实践能力的培养。提高学生的健康素质和审美素养。改革和完善考试评价制度，探索建立教育质量监测和督导评估体系。加强学校教育、家庭教育和社会教育的融合，形成全社会推进素质教育的合力。
　　　**五、保障措施**
　　（一）进一步加强对人口工作的领导。
　　各级政府要从落实科学发展观和构建社会主义和谐社会重大战略思想的高度，把人口发展摆到重要位置。充分认识统筹解决人口问题的长期性、艰巨性、复杂性和紧迫性，把人口发展作为国民经济和社会发展的出发点、着力点和落脚点，切实做到认识、责任、措施、投入“四个到位”。统筹人口规划、人口政策及相关经济社会发展政策等的制定和实施，加强对出生、婚姻、死亡、流动、户籍、就业、教育、医疗、税收、社会保障等人口管理制度的统一协调，建立健全人口发展的宏观调控体系和人口工作的综合治理机制。把人口和计划生育工作作为社会主义新农村建设的重要组成部分。
　　进一步完善党政第一把手亲自抓、负总责的责任制度。把领导干部落实人口发展目标的情况作为衡量政绩和选拔奖惩的重要内容，对工作失职的要追究责任。按照客观公正、突出重点、注重实效、鼓励创新、简便易行的原则，不断完善考核评估方法。
　　（二）构建人口与发展综合决策支持系统。
　　深入开展人口发展战略研究。人口问题是对国民经济和社会发展具有根本性、长期性影响的重大问题。“十一五”时期，在国家人口发展战略研究取得重大成果的基础上，进一步开展对和谐社会、功能区划、老龄化、人口流动迁移等重大课题的研究，不断揭示人口数量、素质、结构、分布与经济、社会、资源、环境之间的变动规律，研究制定与落实科学发展观和构建社会主义和谐社会要求相适应的人口发展指标体系和宏观调控政策。创新人口理论与研究方法，建立健全人口发展战略研究的长效机制。
　　构建人口与发展综合决策支持系统和监测评估体系。创新人口信息采集的手段和方法，建立快速、科学的监测机制，提高信息的真实性和权威性。加强流动人口的信息化建设，实现流动人口信息适时变动、异地查询和跟踪管理。建立部门间人口信息共享机制，实现互联互通和动态更新。加强人口信息的有效整合和系统分析。加快人口宏观管理与决策信息系统建设。建立和完善面向公众的人口信息公共服务网。
　　（三）改革和加强人口发展的社会管理和公共服务体系。
　　完善人口发展的法律法规体系，全面推行依法行政。继续贯彻依法治国基本方略，加强教育、就业、社会保障、卫生、人口和计划生育、环境保护等领域的立法，为实现人口发展目标提供可靠的法律保障。加强法制宣传，加大人口发展领域的执法力度，有法必依、执法必严，维护人民群众的合法权益，尊重和保护人权，使各项工作步入法制化管理的轨道。实行政务公开，加强社会监督。
　　加快转变政府职能。加强政府在人口发展中的社会管理和公共服务职能。在发挥政府主导作用的同时，利用市场机制，打破行政性垄断，充分发挥社区、企业和非政府组织的作用，逐步建立政府监管、社会主办的管理体制。积极探索政府购买服务等方式，调动社会力量兴办社会事业。
　　（四）建立优先投资于人的全面发展的公共财政投入体制。
　　建立稳定的促进人的全面发展的财政投入保障机制。调整财政支出结构，进一步加大对基础教育、就业培训、社会保障、公共卫生、人口和计划生育的投入。人口和计划生育财政投入增长幅度要高于经常性财政收入增长幅度。各级财政要逐年增加投入，到2010年，全国人均人口和计划生育事业费在“十五”期末人均10元的基础上增加到22元，继续安排“十五”期间已将社会抚养费、乡（镇）统筹费纳入财政预算的人口和计划生育事业费支出人均8元，届时，各级财政投入人口和计划生育事业费达到人均30元。进一步加大对农村、中西部和贫困地区人口发展的政策支持和资金扶持力度。
　　利用经济手段，形成良性循环的社会投入机制。建立多渠道的筹资体制，鼓励民间捐资、社会募捐和国际捐赠，引导企业、家庭、个人等加大对人力资本的投入力度。
　　（五）建立人口发展规划监测与评估机制。
　　抓好规划的实施。地方各级人民政府和有关部门共同实施人口发展规划。各地区、各部门要面向社会、面向群众，广泛开展对人口战略思想、发展目标、主要任务、重大政策与重大工程的宣传，在全社会形成关心和参与规划实施的氛围。要以本规划为基本依据，制定和实施相关年度计划。要根据实际情况和职责范围，制定本地区、本部门的具体实施方案。要通过规划引领项目，依靠项目促进规划的实施。
　　加强规划监督评估。在国务院的统一领导下，人口计生委和有关部门共同对人口发展规划的实施进行监督和评估。加强规划实施的动态监测与跟踪分析，及时发现和解决规划执行过程中存在的问题。在“十一五”中期和期末对规划执行情况进行考核评估。地方各级人民政府要定期组织对规划实施情况的检查，促进规划的顺利实施。在规划监督评估中，要发挥群众和团体的积极作用。

**中共中央　国务院
关于全面加强人口和计划生育工作
统筹解决人口问题的决定**
（2006年12月17日）

中发〔2006〕22号

为贯彻党的十六大和十六届三中、四中、五中、六中全会精神，坚持以邓小平理论和“三个代表”重要思想为指导，落实科学发展观和构建社会主义和谐社会的重大战略思想，全面加强新时期人口和计划生育工作，统筹解决我国人口问题，现作出如下决定。

**四、大力提高出生人口素质**
　　提高出生人口素质，事关千家万户的幸福，事关国家和民族的未来。要科学制定提高出生人口素质的规划及行动计划，加强出生缺陷干预能力建设，全面实施出生缺陷干预工程，实行定期评估、通报制度。人口和计划生育技术服务机构与医疗保健机构要在各自职责范围内密切配合，大力宣传和普及预防出生缺陷科学知识，实施计划生育生殖健康促进计划，加强婚育咨询和指导，积极开展婚前和孕前保健、孕产期保健、产前筛查和诊断、产后访视、新生儿疾病筛查和康复等工作；促进住院分娩和母乳喂养，为贫困妇女提供必要的生育救助和安全接生。
　　倡导科学婚检。加强性病和艾滋病防治工作，预防艾滋病母婴传播。对影响出生缺陷的生物遗传、社会环境、不良生活方式等重大危险因素进行研究、评估和干预。大力普及婴幼儿抚养和家庭教育的科学知识，开展婴幼儿早期教育。强化独生子女社会行为教育和培养。
　　**七、积极应对人口老龄化**
　　目前，我国已进入老龄社会，60岁及以上老年人口达1.44亿人，占总人口的11.03%。要制定和落实老龄事业发展战略规划和政策，把逐步建立覆盖城乡居民的养老保障制度作为社会保障体系建设的重点，构建以居家养老为基础、社区服务为依托、机构照料为补充的养老服务体系。
　　农村要探索建立多种形式的计划生育家庭养老保险制度。有条件的地方，可建立政府、集体和社会共同参与的养老服务机构。对生活不能自理的农村计划生育家庭老年父母，按规定提供适当补助。对军烈属、鳏寡及其他有特殊困难的老年人，按规定给予养老救助。
　　城市要逐步完善社会统筹与个人账户相结合的基本养老保险制度，构建多层次的城镇养老保障体系。积极发展适合老年人特点的知识和经验密集型服务业，为老年人提供力所能及参与社会的机会。提高养老服务机构在城市规划中的比重，发展社区老年活动场所和服务设施，制定优惠政策，鼓励社会开办各种类型的养老服务机构。
　　发扬敬老、养老、助老的良好社会风尚，积极探索和实施“爱心护理”等工程。从老年预防保健入手，倡导健康生活方式，营造出行安全和起居方便的环境。探索建立老年服务志愿者、照料储蓄、长期护理保险等社会化服务制度。大力弘扬子女赡养、家庭养老和邻里互助的传统美德。要加强舆论监督，对拒绝赡养或虐待父母的行为，追究法律责任。大力发展老龄产业，建立满足特殊需求的老年用品和服务市场。

**国务院关于加强和改进**

**社区服务工作的意见**

国发〔2006〕14号

各省、自治区、直辖市人民政府，国务院各部委、各直属机构：

随着社会主义市场经济的发展和城镇化进程的加快，城市社区在经济社会发展中的地位越来越重要，社区居民对社区服务的需求越来越多，要求越来越高。做好社区服务工作对于提高居民生活质量、扩大就业、化解社会矛盾、促进和谐社会建设都具有重要意义。现就加强和改进社区服务工作提出以下意见：

**二、大力推进公共服务体系建设，使政府公共服务覆盖到社区**

（四）推进社区就业服务。加强街道、社区劳动保障工作平台建设，通过提供就业再就业咨询、再就业培训、就业岗位信息服务和社区公益性岗位开发等，对就业困难人员提供针对性的服务和援助。结合居民物质文化生活需要开发就业岗位，挖掘社区就业潜力，创建充分就业社区，提高就业稳定性。探索建立信用社区、创业培训与小额担保贷款联动机制，为下岗失业人员自谋职业和自主创业创造条件。建立就业与失业保险、城市居民最低生活保障工作联动机制，促进和帮助享受失业保险、城市居民最低生活保障待遇的相关人员尽快实现就业。

（五）推进社区社会保障服务。加强企业离退休人员社会化管理服务工作，加快老年公共服务设施和服务网络建设。具备条件的地方，可开展老年护理服务，兴建退休人员公寓。充分发挥劳动保障工作平台的作用，促进和帮助城镇居民按规定参加各项社会保险。

（六）推进社区救助服务。加强对失业人员和城市居民最低生活保障对象的动态管理，及时掌握他们的就业及收入状况，切实做到“应保尽保”。积极开展基层社会救助服务，帮助群众解决生产生活中的实际困难。进一步推进社会福利社会化，加快发展社区居家养老服务业。大力发展社区慈善事业，加强对社区捐助接收站点、“慈善超市”的建设和管理。

（七）推进社区卫生和计划生育服务。坚持政府主导、社会力量参与，建立健全以社区卫生服务中心（站）为主体的社区卫生和计划生育服务网络，以妇女、儿童、老年人、慢性病人、残疾人、贫困居民等为重点，为社区居民提供预防保健、健康教育、康复、计划生育技术服务和一般常见病、多发病、慢性病的诊疗服务。大力培养社区卫生服务技术和管理人员，加强对社区卫生服务的监督管理，保证服务质量。实施国家政策规定的计划生育基本项目免费服务。建立民主监督制度，把社区居民满意程度作为考核社区卫生服务工作人员业绩的重要标准。完善社区卫生服务运行机制，发挥社区卫生服务的健康保障功能，努力实现人人享有初级卫生保健的目标。

（八）推进社区文化、教育、体育服务。发展面向基层的公益性文化事业，逐步建设方便社区居民读书、阅报、健身、开展文艺活动的场所，加强对社区休闲广场、演艺厅、棋苑、网吧等文化场所的监督管理，促进社会主义精神文明建设。调动社区资源和力量支持和保障社区内中小学校开展素质教育和社会实践活动，为青少年健康成长创造良好的社区环境。落实《全民科学素质行动计划纲要》，不断提高居民科学素质。统筹各类教育资源，充分发挥社区学院、市民学校的作用，积极创建各种类型的学习型组织，面向社区居民开展多种形式的教育培训和科普活动，建立覆盖各类人群的多渠道、全方位的社区学习服务体系。培育群众性体育组织，落实《全民健身计划纲要》，配置相应的健身器材，不断增强居民体质。

（九）推进社区流动人口管理和服务。按照“公平对待、合理引导、完善管理、搞好服务”和“以现居住地为主，现居住地和户籍所在地互相配合”的原则，实行与户籍人口同宣传、同服务、同管理，为流动人口的生活与就业创造好的环境和条件。简化办事程序，减少相关手续，取消不合理收费，为流动人口提供优质服务。

（十）推进社区安全服务。深入开展基层安全创建活动，加强社区警务室（站）建设，大力实施社区警务战略，建立人防、物防、技防相结合的社区防范机制和防控网络。依托社区居委会等基层组织，挖掘和利用社区资源，加强群防群治队伍建设。深入开展法制宣传教育和咨询服务活动，建立完善收集、反馈社情民意的工作机制，组织开展以社区保安、联防队员为主体，专职和义务相结合的巡逻守望、看楼护院等活动。建立及时有效的矛盾纠纷排查、调处工作机制，加强对刑释解教人员、监外执行人员和有不良行为青少年的帮助、教育和转化工作。做好社区消防工作，提升社区消防安全水平。深入开展打击“黄赌毒”和禁止传销等工作。健全社区环境保护管理制度，建设资源节约型、环境友好型社区。建立传染病、食品安全、灾害事故的应急反应机制，不断提高社区应对突发事件的能力。

（十一）不断改进政府公共服务方式。整合政府各部门在城市基层的办事机构，积极推进“一站式”服务，提高为社区及其居民提供公共服务的水平。政府有关部门不得将应由自身承担的行政性工作摊派给社区组织。对有些社区组织做起来有优势的行政性工作，可依法采取“权随责走、费随事转”的原则，委托社区组织承担。积极探索通过政府“购买服务”、项目管理等多种形式，调动社会组织参与社区服务的积极性，促进公共服务社会化。梳理、整合各类服务热线、呼叫热线，形成社区公共资源共享机制。建设社区信息化平台，提高社区公共服务的自动化、现代化水平。

**三、充分发挥社区居委会在社区服务中的作用**

（十二）支持社区居委会协助城市基层政府提供社区公共服务。充分发挥社区居委会在了解社区居民需求、提供便民服务方面的独特优势和重要作用。城市基层政府及有关单位要妥善解决社区居委会开展有关服务所必需的房屋、设施和工作经费。要积极指导社区居委会定期听取居民对社区公共服务的意见，并积极向政府反映，促进社区公共服务质量的不断提高。

（十三）支持社区居委会组织社区成员开展自助和互助服务。鼓励并支持社区居委会组织动员驻社区单位和社区居民开展邻里互助等群众性自我服务活动，为居家的孤老、体弱多病和身边无子女老人提供各种应急服务，为优抚对象、残疾人及特困群体缓解生活困难提供服务；倡导社区居民和驻社区单位开展社会捐赠、互帮互助，对社区困难群体实行辅助性生活救助；管理、利用好社区公益性服务设施，方便社区成员生活。有条件的地方，社区居委会可以根据居民需要，建立热线电话救助网络、社区智能服务网络、社区服务站、社区公共服务社等服务载体，开展非营利服务。

（十四）指导社区居委会为发展社区服务提供便利条件。鼓励并指导社区居委会组织居民参与文化、教育、科技、体育、卫生、法律、安全等进社区活动；支持社会各方面力量利用闲置设施、房屋等资源兴办购物、餐饮、就业、医疗、废旧物资回收等与居民生活密切相关的服务网点，并维护其合法权益；引导和管理各类组织和个人依法有序开展社区服务；正确处理好社区居委会与社区物业管理企业的关系，支持和指导物业管理企业依法经营。

**四、培育社区服务民间组织，组织开展社区志愿服务活动**

（十五）大力培育社区生活服务类民间组织。支持和鼓励社区居民成立形式多样的慈善组织、群众性文体组织、科普组织和为老年人、残疾人、困难群众提供生活服务的组织，使社区居民在参与各种活动中，实现自我服务、自我完善和自我提高。积极支持民间组织开展社区服务活动，加强引导和管理，使其在政府和社区居委会的指导、监督下有序开展服务。

（十六）积极组织开展社区志愿服务活动。培育社区志愿服务意识，弘扬社区志愿服务精神，推行志愿者注册制度。积极动员共产党员、共青团员、公务员、专业技术人员、教师、青少年学生以及身体健康的离退休人员等加入志愿服务队伍，优化志愿人员结构，壮大志愿人员力量。指导建立志愿服务激励机制，使志愿者本人需要帮助时，能够及时得到志愿者组织和其他志愿者的服务。指导志愿组织和志愿人员开展社会救助、优抚、助残、老年服务、再就业服务、维护社区安全、科普和精神文明建设活动，不断创新服务形式，提高服务水平。

**六、加强领导和政策指导，强化社区服务监管**

（十九）加强组织领导。地方各级人民政府和有关部门要充分认识新形势下搞好社区服务的重要性，把这项工作与提高居民生活质量、实施社会救助和再就业工程、发展第三产业、促进精神文明建设等各项工作紧密结合起来。要建立健全政府统一领导、民政部门牵头、有关部门配合、社会广泛参与的社区服务管理体制和工作机制。依托社区提供公共服务的教育、科技、公安、司法行政、劳动保障、建设、文化、卫生、人口计生、环保、体育等部门，要按照社区服务发展要求加强业务指导，提高服务水平。各级发展改革、财政、商务、银行、税务、工商等部门要按照各自职能，进一步制定促进社区服务发展的政策措施。积极鼓励工会、共青团、妇联及残联、老龄、慈善等组织参与社区服务，大力倡导团结互助、扶贫济困的良好风尚，形成推动社区服务发展的合力。

（二十）加强社区服务工作队伍建设。切实解决社区居委会成员及其聘用的服务人员的生活补贴、工资、保险等福利待遇问题，并使待遇水平随经济发展而适当增长。经常开展对社区服务人员的思想教育和业务培训，不断提高他们服务居民、管理社区的能力。加强对社区服务的理论研究，鼓励有条件的大专院校和培训机构开设社会工作专业、社区服务课程，培养专业人才。

（二十一）加强社区服务的统筹规划和政策指导。地方各级人民政府要从实际出发，因地制宜，分级制订社区服务发展规划，确定发展目标和重点，完善相关政策措施，促进社区服务各项工作的落实。要将社区服务设施建设纳入城市规划和土地利用规划，统筹安排，通过新建和改造，完善社区各类服务设施，健全服务体系，增强服务功能。有条件的地方，可以开展农村社区服务的试点，逐步实现城乡社区服务统一规划，统筹发展。

地方各级人民政府和有关部门要帮助社区落实开展公共服务的资金、场所和人员，对社区组织开展的互助性服务、志愿服务和社会力量兴办的微利性商业服务给予政策和资金扶持；对社区营利性商业服务要积极引导向产业化、市场化发展，充分发挥行政机制、互助机制、志愿机制、市场机制在社区服务中的作用。积极推进适宜产业化经营的社区服务实体的股份制改造；鼓励大型服务企业兼并、控股国有或集体所有的社区服务单位，支持个体私营经济参股或兴办社区服务企业。

（二十二）加强对社区服务活动的监督管理。综合运用行政、法律手段监督、管理社区服务。推动制定各类社区服务行业标准并监督执行。建立健全反映社区服务设施、服务管理、居民需求及满意程度等有关信息的采集及工作评估体系。严格财务和审计制度，严禁将救助、福利、公益款物等挪作他用。认真解决社区服务发展中的各种问题，及时查处违法违纪和损害群众利益的行为，保证社区服务健康发展。

**国家发展改革委、科技部、财政部、中国科协关于印发科普基础设施发展规划(2008－2010－2015)的通知**

  推动国内外交流与合作。引进、借鉴国际先进理念和方法，提高国内自主开发科普展教品的能力。营造交流与合作环境，推动国内外科普展教品研发的有效合作。适度引进国外优秀展教资源，推动科普展教品国内外交流与合作。
  2．创新科普展览和教育活动
  强化科普展览和教育活动的策划与组织。围绕未成年人、老年人、农民、城镇劳动人口、领导干部和公务员等人群的心理特点和个性化需求，结合青少年活动中心、社区活动中心、农业教育培训机构、企业职业技能培训机构和党校、行政院校、干部学院等开展的培训教育活动，设计开发各类互动式、体验式科普展览和教育活动。将开发科教影视节目、科普图书、挂图等展教资源与各类科普基础设施的展教活动有机结合起来，提高资源利用率和活动效果。推动旅游景区、农业观光园、绿色生态园等结合自身优势，开展特色科普展教活动。策划针对中西部地区的科普展览和科普活动。
  推动主题展览和常设展品在中小科技场馆之间进行交流与共享。搭建互动平台，形成工作机制，丰富中小科技场馆的展示和活动内容，逐步提高中小科技场馆开展科普活动的能力与水平。依托图书馆、文化馆、活动中心等场所，推出简便易行、具有特色的科普展览和科普活动。增加科技实验趣味性演示和科技实用技术性培训的内容，加大对学校科学实验室、实验器材、教具等科普设施设备的配备。
  促进科普展教活动与学校科学课程教学、综合实践和研究性学习相衔接。适应学校科学教育的要求，集成现有展教资源并适当研发新的展教资源，将学校的科学课程安排到科技类博物馆和科普基地等科普设施中。集成优化科技类博物馆和科普基地等各项科普设施的科普展览和教育资源，送到广大农村地区和中小城市的中小学。
  3．培育科普展教资源产业
  培育科普展教资源市场。制定和完善有关优惠政策，引入市场机制，加强与文化创意产业的结合，推动设计制作社会化。鼓励科研机构、大学、企事业单位、社会团体等加强合作，参与科普产品研发中心的建设和展教资源的开发活动。支持企业经营展教资源的研发、生产、销售和服务，为各类科普设施提供市场化的展览开发服务。建立区域合作与互助机制，促进东西部、发达地区和欠发达地区之间的展教资源交流。开展科普展教资源研发理论研究，完善展教资源技术规范和设计制作机构资质认定办法等，培育科普展览策划、研制、使用、推广的一体化产业。
  加强知识产权保护。开展科普产品知识产权战略和管理的研究工作，完善科普产品知识产权保护相关政策及制度。开展知识产权宣传工作，加强法制教育，营造尊重和保护科普产品知识产权的良好环境。加强科普产品知识产权法律实施的监督、检查工作，保障知识产权保护制度的有效实施。
  （二）科普基础设施拓展工程
  科普基础设施拓展工程是为全体公民提供更多参与科普教育活动机会的公共服务体系保障。基本思路是：充实和完善现有各类科普基础设施的科普教育功能，统筹利用、挖掘潜力，拓展和提升社会设施资源的科普服务能力，改建、扩建和新建相结合，形成各类科普基础设施优势互补、协同发展的良好格局。
  4．拓展完善科技类博物馆
  更新改造现有科技类博物馆。按照《科学技术馆建设标准》，对不具备展教功能或不能充分发挥科普作用的科技馆进行必要的更新改造，激发活力，满足公众参与科普活动的需求。有计划地对现有自然博物馆进行更新改造，引入新理念，从简单展出标本向揭示自然发展规律等主题展示转变。配合国家重大安排和重点区域布局，突出生态环境保护、防震减灾等区域重点和特色，在科技馆、自然博物馆、天文馆等适当新增部分内容。
  挖掘潜在社会科普设施资源。充分利用国家有关重大工程项目或企业闲置、淘汰的生产设施，建设工业科技类博物馆。在有条件的研究机构、大学和具有重要资源的城市，利用现有设施和资源建设专业或产业科技类博物馆。积极推动农业科学技术博物馆、健康科学博物馆等具有专业特色的科技类博物馆建设。结合国家文物类博物馆、工程技术展览馆等建设，充实科学技术相关内容。与建立民族、民俗博物馆相结合，在少数民族地区建立具有民族特色的科技类博物馆。
  适当新建科技类博物馆。鼓励社会力量参与科技类博物馆建设，结合区域、产业发展重点，积极推动科技馆（科学中心）、自然科学博物馆、天文馆以及专业科技馆、产业科技馆等在全国各区域、各层级的合理布局，避免科技类博物馆建设的功能重复、形式单一、内容雷同。鼓励、推动有条件的企事业单位根据自身特点，因地制宜地建设一批工业科技类博物馆或产业科技类博物馆。注重内容建设，将展览展品设计纳入新建科技类博物馆建设工程的整体规划中，鼓励设计理念、主题内容和展示框架的创新，提高展教品制作工艺水平，增强展览展品的互动性、生动性、趣味性。
  5．开发开放科普基地
  推进各类科普基地建设。适度发展国家级和省部级科普基地。充分发挥各行业部门和地方优势，根据自身特点和资源，把农业、林业、国土资源、医疗卫生、计划生育、生态环境保护、安全生产、气象、地震、体育、文物、旅游、妇女儿童、民族、国防教育等工作与科普工作有机结合，按照开展科学技术教育、传播与普及等需要，建设不同功能的行业科普基地。
  挖掘和综合利用社会科普教育资源。推动科研机构和大学面向公众开放实验室、研究中心等科研设施，支持和鼓励科研机构和大学创造条件设立面向公众的专门科普场所。推动青少年宫和青少年实践基地等未成年人校外活动场所、妇女儿童活动中心、家长学校、文化宫、职工学校、技工院校、职业技能培训机构和农村致富技术函授大学等增加科普内容，实现科普教育的功能。完善相关政策措施，鼓励高新技术园区开展科普活动，有条件的企业面向公众开放研发机构、生产设施（流程）或展览馆，并根据自身特点建设专门科普场所；引导海洋馆、野生动物园、主题公园、自然保护区、森林公园、地质公园、动植物园等经营性旅游场馆强化科普教育功能。
  6．大力发展基层科普设施
  完善基层科普服务设施体系。推动在全国所有的县（市、区）建设具备科普教育、培训、展示等功能的县级综合性科普活动场所。在县（市、区）、街道（乡镇）、城乡社区（村）的公共活动场所建立科普活动站（室）、科普画廊（宣传栏），定期更新科普内容。
  依托现有社会设施共建共享基层科普设施。依托县级文化馆、图书馆、青少年活动中心、妇女儿童活动中心、少年宫等，拓展科普教育功能，建设县级综合性科普活动场所。依托遍布在乡镇（街道）、村（社区）的文化站、广播站、中小学校、成人文化技术学校、职业培训学校、党校（党员活动室）以及有条件的乡镇企业、农村专业合作经济组织等公共设施，结合农村党员干部现代远程教育、全国文化信息资源共享工程、“农家书屋”和“农民科技书屋”工程、科技大院等国家重点项目，增加科普图书、挂图、声像资料以及有关展示设备的数量和比例，丰富科普教育内容，建设“科普活动站（室）”、“科普图书室”、“社区科普学校（大学）”等基层科普阵地。有条件的中小学根据科学课程的需要，利用现有的教育培训场所、基地，充实实验仪器、教具、音像设备、计算机等教学器材，建立青少年科学工作室。发展具有地方特色的农业观光园、绿色生态园和科技示范园，增强其农业科技教育服务功能。拓展各类职业培训中心、再就业培训中心（基地）等基础设施的科普功能。
  7．加大科普大篷车建设力度
  完善科普大篷车等流动科普设施的布局。增加科普大篷车配发数量，重点向地（市、州）和有条件的县（市、区）倾斜。鼓励有条件的地方发展符合当地需求的流动科普设施。探索与社会各界共建“科普大篷车”的新形式，拓展“西部乡村流动图书车”、“农业科技入户直通车”等流动设施的科普展教功能。鼓励、引导社会各方面力量参与各类流动科普设施的研制、配发和运行，扩大配发覆盖面，搭建省、地、县三级服务梯形结构，使其活动覆盖全国城乡社区。
  扩展科普大篷车功效。根据服务对象的不同需求，开发研制专题科普大篷车系列车型。充实和完善已有各类流动设施的科普功能，不断创新车载设备和展品的形式与内容，丰富活动形式，提高活动效果。研究适合科普大篷车运行的活动模式和教育项目。完善科普大篷车相关技术标准、产品生产规范。加大公共投入，制定优惠政策，广泛吸纳社会资金，为科普大篷车的配发及运行服务提供支撑和保障。
  （三）数字科技馆建设工程
  数字科技馆建设工程是利用网络信息技术开展科普活动的重要手段，是对实体科普基础设施的重要补充。基本思路是：健全数字科技馆共建共享机制，集成社会现有科普资源并进行数字化开发和转化，加快支撑服务体系建设，重点建设科普基础设施资源门户系统和面向社会的展示服务系统，搭建功能完备、运行高效的科普传播平台。
  8．集成和开发数字科普资源
  建设数字化科普资源库。建立有效机制，集成全社会优质科普资源，对各类科技、教育等资源进行开发和转化，实现资源的科普化、数字化、信息化、网络化和集成化。做好整体规划和标准制订，定期发布科普资源建设指南，不断完善科普资源内容的覆盖面和规范化建设。及时更新科普内容，重点建立科普图库、科普动漫作品库、科普音像库、科普书库、科普报告库、科普基地资源库、科技馆展品库和博物馆藏品库等若干数字化科普资源数据库。
  9．完善科普资源信息服务功能
  强化中国数字科技馆的平台功能。拓展科普信息发布、虚拟科普社区、资源集成与组合、动态跟踪与监管等功能，构建支持视频、音频和图像等多媒体形式的交互式科普信息内容的集成平台、发布平台和管理平台。培育和扶持一批对公众有较强吸引力的优秀科普网站。
  建立虚拟科普场馆。利用多媒体、虚拟现实和人机交互等现代信息技术，配置数字化藏品和场景，建立主题虚拟博物馆或各类兼具知识传播和科学实践功能的专题虚拟科学体验区，构建包括观察认知、探索体验和实验制作等众多主题的虚拟科学乐园，使公众通过人机交互等方式体验科学的过程。
  10．健全科普信息资源共建共享机制
  完善数字科技馆标准规范体系。加强规范化建设，遵循“实用、简明、可操作”原则，制定和完善科学合理的技术、质量和可用性标准与规范。加大标准规范的执行力度，不断提升建设质量。建立全面高效的评估系统，促进实现数字科技馆的长效服务功能。
  建立数字科技馆共建共享机制。健全保障数字科技馆建设与运行的绩效考核机制、共享监管机制和人才评价机制，探索数字科技馆的市场化运行机制。完善资源整合与共享过程中的部门协调机制与措施，加强应用推广。坚持政府引导与社会参与、公益性与市场机制相结合原则，调动拥有数字科普资源的各方面力量，积极参与数字科技馆建设。
  （四）科普人才队伍培养工程
  科普人才队伍培养工程是促进科普基础设施长效发展的人力资源保障。基本思路是：完善正规教育体系中科普基础设施适用人才培养体系和科普基础设施人员在职培训体系；加强与社会兼职科普专家的密切联系，发展和壮大兼职、志愿者队伍。
  11．重点建设专职科普人才队伍
  完善正规教育体系中科普基础设施适用人才培养工作。加强科技传播学和科技博物馆学等学科建设，利用现有科技传播学、科学教育、科学技术史、科学技术哲学、科学社会学、工业设计、动漫制作等学科点和相关专业博士后流动站，培养科技传播和科技博物馆领域的研究、开发、设计和制作专业人才，以及各类科普设施运行管理服务人才。支持大学开设科技传播、博物馆学、科学技术史、科学技术与社会、科学方法论、科普创作等课程，鼓励设立科技传播及科技博物馆领域和方向的博士后工作站和研究项目，激发大学生对科普事业的兴趣。
  建立科普基础设施人员在职培训体系。围绕科普基础设施建设与运行管理，充分利用现有科技场馆、研究机构、高等院校资源，通过设立专题进修班、研究生课程班等，积极开展学术探讨和经验交流，加大在职人员培训力度，提升从业人员的创新服务能力和综合素质。不断拓宽国际合作渠道，加强科普设施人才的国际交流与培养。
  加强科普岗位人员配置。完善考核激励机制，强化岗位设置和人员配备，建立科技类博物馆馆长的职业化管理模式，增加在编专职科学教育或展教人员的比例。在特大、大型科技馆等科普场馆设立展教资源研发岗位。采取相应措施，吸引工业、艺术等展览专业人才从事科普教育工作。逐步改进和完善各类科普设施的用人机制和分配政策，制定与科普工作岗位特点相适应的工作业绩考核和评价办法，逐步形成激发从业人员不断进取、创新服务的激励机制。
  12．积极发展兼职、志愿者科普队伍
  建设稳定、高素质的兼职科普队伍。科技工作者有义务参与科学技术教育、传播与普及工作。通过设立荣誉或客座职位，鼓励大学、研究机构和传媒等领域的专家、学者到科技类博物馆、科普基地等兼职。充分利用基层单位的人才资源，建立专兼结合、一专多能的社区、乡村科普宣传员队伍，积极推动大学生村官兼任科普宣传员。
  发展壮大志愿者科普队伍。充分发挥在职科技工作者、高校学生和离退休科技、教育和传媒工作者等各界人士的专业和技术特长，鼓励他们积极参与科学教育、传播与普及工作，及时将科学前沿的研究成果转化为科普资源。鼓励在校大学生、研究生利用假期社会实践和支农支边支教等活动开展科普宣传。充分利用少数民族科普工作队、县级科普工作队等形式，动员和组织广大科技工作者深入基层开展科技教育、传播与普及活动。探索有效激励机制，推动建设能够定期、长期深入城乡社区和边远、贫困和少数民族地区开展科普宣传活动的志愿者队伍。
  提高兼职、志愿者科普队伍服务能力。加强兼职、志愿者队伍的培训，提高科普教育服务能力。研究制定对科普基地工作人员进行业绩考核的办法，将其开展的科普教育工作纳入业绩考核范围，调动他们的积极性。
  未来三年的重点任务：
  未来三年是推动我国科普基础设施发展的重要阶段。国家将逐步加大对科普基础设施的支持力度，采取相应的行动，落实相关任务，集中优势资源，着力解决关键环节，努力实现一下具体任务：
  1．加强科普展教资源的创新和开发。研究制定《科普资源共建共享工作方案》，重点围绕“节约能源资源、保护生态环境、保障安全健康”等主题，开发一批展品、图书、挂图、音像制品和设备等。推进国家科技计划项目科普创作试点，推出一批科普创作精品。开发适合科普大篷车活动的互动表演剧、科普活动资源包和主题展览。推进“科技馆活动进校园”工作，设计和开发一批与学校科学课程有机结合的活动项目。制作一批在青少年中有广泛影响且具有知识性、趣味性的科普作品和科普网络游戏。发展适合乡村党员活动室、文化站、科技大院、农家书屋等设施的科普展教品，促进基层科普设施的内容建设。
  2．加强科普展教资源的共享与服务。向社会推介优秀科普作品和选题。继续实施中小科技馆支援计划，重点推动主题展览的巡展和交换，支持中小科技场馆充实和丰富展教内容，为中小科技场馆提供技术支持和人员培训服务，提高其业务水平。
  3．加强科普产品研发中心建设。扶持一批不以盈利为目的、专业化的展教资源设计和开发机构，使其初步具备科普展教资源的基础性、原创性研发能力，助其成为科普产品研发中心。
  4．推动现有特大、大型和中型科技馆达标。按照《科学技术馆建设标准》，对各地科技馆进行考核评估，推动不能充分发挥科普作用的科技馆进行必要的更新完善和机制改革，激发活力，改进服务，满足公众需求。
  5．加强对各地建设科技类博物馆的指导。指导各地在未建立科技类博物馆的省会城市和自治区首府以及常住人口100万以上的城市新建20-30座科技类博物馆，其中特大、大型和中型科技馆10-15座。在我国具有天文观测站的重点城市新建、扩建5座天文馆，展示天文学的最新成就，激发公众尤其是青少年的兴趣。推动在西藏、青海、新疆等地围绕生态环境保护等内容建设主题自然科学博物馆。推动面向妇女、儿童等特定人群的博物馆建设。
     四、保障措施
  政策法规、经费投入以及组织实施等，是推动科普工作体制机制创新，动员全社会力量参与，推动科普基础设施发展的重要保障。
  （一）政策法规
    加快相关政策法规的制定，完善国家公共科普基础设施管理体制和运行机制。一是研究制定促进科普展教资源建设的政策。推动将国家科技计划项目成果转化为科普资源，探索建立科技成果及时转化为展教资源的工作机制。创造公共科普展教资源公平使用的政策环境，推动科普文化产业健康发展。二是研究制定加强科普基础设施公共服务的政策。推动公益类科技馆、科普基地等科普基础设施优惠开放，进一步推进科研机构和大学面向社会开展科普活动。三是研究制定加快人才队伍建设的相关政策。将相关科普人才的培养列入国家人才工作规划。依托重点科普设施建设，吸引和凝聚高水平人才。探索建立有效机制和激励措施，充分调动在职和离退休科技、教育、传媒工作者、大学生、研究生等各界人士从事科技传播和普及工作的积极性。
  （二）经费投入
  将科普基础设施建设纳入国民经济和社会事业发展总体规划。加大对公益性科普基础设施建设和运行经费的公共投入。加强国家对社会资金的引导，多渠道、多层次筹措资金，鼓励社会力量参与科普基础设施建设和运行服务。
  （三）组织实施
  《科普设施规划》是贯彻落实《科学素质纲要》的重要任务。国家发展改革委、科技部、财政部和中国科协负责协调和推动落实，相关部门结合本部门的职能制定工作规划和计划并加以落实。各级地方政府要将科普基础设施建设纳入国民经济和社会事业发展总体规划，落实建设和运行经费。充分发挥各有关学会、协会和研究会等社会团体的作用，加强对各类科普基础设施建设与运行的咨询和指导。
  按照《科学素质纲要》实施的要求，研究制定科普基础设施建设与运行的监测评估指标体系，定期开展监测评估工作，促进科普基础设施全面、协调、可持续发展。对作出突出成绩的单位和个人进行表彰和奖励。

**国务院办公厅关于印发社会养老
服务体系建设规划（2011-2015年）的通知**

**社会养老服务体系建设规划（2011－2015年）**

　为积极应对人口老龄化，建立起与人口老龄化进程相适应、与经济社会发展水平相协调的社会养老服务体系，实现党的十七大确立的“老有所养”的战略目标和十七届五中全会提出的“优先发展社会养老服务”的要求，根据《中华人民共和国国民经济和社会发展第十二个五年规划纲要》和《中国老龄事业发展“十二五”规划》，制定本规划。
　　**一、规划背景**
　　（一）现状和问题。
　　自1999年我国步入老龄化社会以来，人口老龄化加速发展，老年人口基数大、增长快并日益呈现高龄化、空巢化趋势，需要照料的失能、半失能老人数量剧增。第六次全国人口普查显示，我国60岁及以上老年人口已达1.78亿，占总人口的13.26%，加强社会养老服务体系建设的任务十分繁重。
　　近年来，在党和政府的高度重视下，各地出台政策措施，加大资金支持力度，使我国的社会养老服务体系建设取得了长足发展。养老机构数量不断增加，服务规模不断扩大，老年人的精神文化生活日益丰富。截至2010年底，全国各类收养性养老机构已达4万个，养老床位达314.9万张。社区养老服务设施进一步改善，社区日间照料服务逐步拓展，已建成含日间照料功能的综合性社区服务中心1.2万个，留宿照料床位1.2万张，日间照料床位4.7万张。以保障三无、五保、高龄、独居、空巢、失能和低收入老人为重点，借助专业化养老服务组织，提供生活照料、家政服务、康复护理、医疗保健等服务的居家养老服务网络初步形成。养老服务的运作模式、服务内容、操作规范等也不断探索创新，积累了有益的经验。
　　但是，我国社会养老服务体系建设仍然处于起步阶段，还存在着与新形势、新任务、新需求不相适应的问题，主要表现在：缺乏统筹规划，体系建设缺乏整体性和连续性；社区养老服务和养老机构床位严重不足，供需矛盾突出；设施简陋、功能单一，难以提供照料护理、医疗康复、精神慰藉等多方面服务；布局不合理，区域之间、城乡之间发展不平衡；政府投入不足，民间投资规模有限；服务队伍专业化程度不高，行业发展缺乏后劲；国家出台的优惠政策落实不到位；服务规范、行业自律和市场监管有待加强等。
　　（二）必要性和可行性。
　　我国的人口老龄化是在“未富先老”、社会保障制度不完善、历史欠账较多、城乡和区域发展不平衡、家庭养老功能弱化的形势下发生的，加强社会养老服务体系建设的任务十分繁重。
　　加强社会养老服务体系建设，是应对人口老龄化、保障和改善民生的必然要求。目前，我国是世界上唯一一个老年人口超过1亿的国家，且正在以每年3%以上的速度快速增长，是同期人口增速的五倍多。预计到2015年，老年人口将达到2.21亿，约占总人口的16%；2020年达到2.43亿，约占总人口的18%。随着人口老龄化、高龄化的加剧，失能、半失能老年人的数量还将持续增长，照料和护理问题日益突出，人民群众的养老服务需求日益增长，加快社会养老服务体系建设已刻不容缓。
　　加强社会养老服务体系建设，是适应传统养老模式转变、满足人民群众养老服务需求的必由之路。长期以来，我国实行以家庭养老为主的养老模式，但随着计划生育基本国策的实施，以及经济社会的转型，家庭规模日趋小型化，“4-2-1”家庭结构日益普遍，空巢家庭不断增多。家庭规模的缩小和结构变化使其养老功能不断弱化，对专业化养老机构和社区服务的需求与日俱增。
　　加强社会养老服务体系建设，是解决失能、半失能老年群体养老问题、促进社会和谐稳定的当务之急。目前，我国城乡失能和半失能老年人约3300万，占老年人口总数的19%。由于现代社会竞争激烈和生活节奏加快，中青年一代正面临着工作和生活的双重压力，照护失能、半失能老年人力不从心，迫切需要通过发展社会养老服务来解决。
　　加强社会养老服务体系建设，是扩大消费和促进就业的有效途径。庞大的老年人群体对照料和护理的需求，有利于养老服务消费市场的形成。据推算，2015年我国老年人护理服务和生活照料的潜在市场规模将超过4500亿元，养老服务就业岗位潜在需求将超过500万个。
　　在面对挑战的同时，我国社会养老服务体系建设也面临着前所未有的发展机遇。加强社会养老服务体系建设，已越来越成为各级党委政府关心、社会广泛关注、群众迫切期待解决的重大民生问题。同时，随着我国综合国力的不断增强，城乡居民收入的持续增多，公共财政更多地投向民生领域，以及人民群众自我保障能力的提高，社会养老服务体系建设已具备了坚实的社会基础。
　　**二、内涵和定位**
　　（一）内涵。
　　社会养老服务体系是与经济社会发展水平相适应，以满足老年人养老服务需求、提升老年人生活质量为目标，面向所有老年人，提供生活照料、康复护理、精神慰藉、紧急救援和社会参与等设施、组织、人才和技术要素形成的网络，以及配套的服务标准、运行机制和监管制度。
　　社会养老服务体系建设应以居家为基础、社区为依托、机构为支撑，着眼于老年人的实际需求，优先保障孤老优抚对象及低收入的高龄、独居、失能等困难老年人的服务需求，兼顾全体老年人改善和提高养老服务条件的要求。
　　社会养老服务体系建设是应对人口老龄化的一项长期战略任务，是坚持政府主导，鼓励社会参与，不断完善管理制度，丰富服务内容，健全服务标准，满足人民群众日益增长的养老服务需求的持续发展过程。本建设规划仅着眼于构建体系建设的基本框架。
　　（二）功能定位。
　　我国的社会养老服务体系主要由居家养老、社区养老和机构养老等三个有机部分组成。
　　居家养老服务涵盖生活照料、家政服务、康复护理、医疗保健、精神慰藉等，以上门服务为主要形式。对身体状况较好、生活基本能自理的老年人，提供家庭服务、老年食堂、法律服务等服务；对生活不能自理的高龄、独居、失能等老年人提供家务劳动、家庭保健、辅具配置、送饭上门、无障碍改造、紧急呼叫和安全援助等服务。有条件的地方可以探索对居家养老的失能老年人给予专项补贴，鼓励他们配置必要的康复辅具，提高生活自理能力和生活质量。
　　社区养老服务是居家养老服务的重要支撑，具有社区日间照料和居家养老支持两类功能，主要面向家庭日间暂时无人或者无力照护的社区老年人提供服务。在城市，结合社区服务设施建设，增加养老设施网点，增强社区养老服务能力，打造居家养老服务平台。倡议、引导多种形式的志愿活动及老年人互助服务，动员各类人群参与社区养老服务。在农村，结合城镇化发展和新农村建设，以乡镇敬老院为基础，建设日间照料和短期托养的养老床位，逐步向区域性养老服务中心转变，向留守老年人及其他有需要的老年人提供日间照料、短期托养、配餐等服务；以建制村和较大自然村为基点，依托村民自治和集体经济，积极探索农村互助养老新模式。
　　机构养老服务以设施建设为重点，通过设施建设，实现其基本养老服务功能。养老服务设施建设重点包括老年养护机构和其他类型的养老机构。老年养护机构主要为失能、半失能的老年人提供专门服务，重点实现以下功能：1.生活照料。设施应符合无障碍建设要求，配置必要的附属功能用房，满足老年人的穿衣、吃饭、如厕、洗澡、室内外活动等日常生活需求。2.康复护理。具备开展康复、护理和应急处置工作的设施条件，并配备相应的康复器材，帮助老年人在一定程度上恢复生理功能或减缓部分生理功能的衰退。3.紧急救援。具备为老年人提供突发性疾病和其他紧急情况的应急处置救援服务能力，使老年人能够得到及时有效的救援。鼓励在老年养护机构中内设医疗机构。符合条件的老年养护机构还应利用自身的资源优势，培训和指导社区养老服务组织和人员，提供居家养老服务，实现示范、辐射、带动作用。其他类型的养老机构根据自身特点，为不同类型的老年人提供集中照料等服务。
　　**三、指导思想和基本原则**
　　（一）指导思想。
　　以邓小平理论和“三个代表”重要思想为指导，深入贯彻落实科学发展观，以满足老年人的养老服务需求为目标，从我国基本国情出发，坚持政府主导、政策扶持、多方参与、统筹规划，在“十二五”期间，初步建立起与人口老龄化进程相适应、与经济社会发展水平相协调，以居家为基础、社区为依托、机构为支撑的社会养老服务体系，让老年人安享晚年，共享经济社会发展成果。
　　（二）基本原则。
　　1.统筹规划、分级负责。加强社会养老服务体系建设是一项长期的战略任务，各级政府对养老机构和社区养老服务设施的建设和发展统筹考虑、整体规划。中央制定全国总体规划，确定建设目标和主要任务，制定优惠政策，支持重点领域建设；地方制定本地规划，承担主要建设任务，落实优惠政策，推动形成基层网络，保障其可持续发展。
　　2.政府主导、多方参与。加强政府在制度、规划、筹资、服务、监管等方面的职责，加快社会养老服务设施建设。发挥市场在资源配置中的基础性作用，打破行业界限，开放社会养老服务市场，采取公建民营、民办公助、政府购买服务、补助贴息等多种模式，引导和支持社会力量兴办各类养老服务设施。鼓励城乡自治组织参与社会养老服务。充分发挥专业化社会组织的力量，不断提高社会养老服务水平和效率，促进有序竞争机制的形成，实现合作共赢。
　　3.因地制宜、突出重点。根据区域内老年人口数量和养老服务发展水平，充分依托现有资源，合理安排社会养老服务体系建设项目。以居家养老服务为导向，以长期照料、护理康复和社区日间照料为重点，分类完善不同养老服务机构和设施的功能，优先解决好需求最迫切的老年群体的养老问题。
　　4.深化改革、持续发展。按照管办分离、政事政企分开的原则，统筹推进公办养老服务机构改革。区分营利性与非营利性，加强对社会养老服务机构的登记和监管。盘活存量，改进管理。完善养老服务的投入机制、服务规范、建设标准、评价体系，促进信息化建设，加快养老服务专业队伍建设，确保养老机构良性运行和可持续发展。
　　**四、目标和任务**
　　（一）建设目标。
　　到2015年，基本形成制度完善、组织健全、规模适度、运营良好、服务优良、监管到位、可持续发展的社会养老服务体系。每千名老年人拥有养老床位数达到30张。居家养老和社区养老服务网络基本健全。
　　（二）建设任务。
　　改善居家养老环境，健全居家养老服务支持体系。以社区日间照料中心和专业化养老机构为重点，通过新建、改扩建和购置，提升社会养老服务设施水平。充分考虑经济社会发展水平和人口老龄化发展程度，“十二五”期间，增加日间照料床位和机构养老床位340余万张，实现养老床位总数翻一番；改造30%现有床位，使之达到建设标准。
　　在居家养老层面，支持有需求的老年人实施家庭无障碍设施改造。扶持居家服务机构发展，进一步开发和完善服务内容和项目，为老年人居家养老提供便利服务。
　　在城乡社区养老层面，重点建设老年人日间照料中心、托老所、老年人活动中心、互助式养老服务中心等社区养老设施，推进社区综合服务设施增强养老服务功能，使日间照料服务基本覆盖城市社区和半数以上的农村社区。
　　在机构养老层面，重点推进供养型、养护型、医护型养老设施建设。县级以上城市，至少建有一处以收养失能、半失能老年人为主的老年养护设施。在国家和省级层面，建设若干具有实训功能的养老服务设施。
　　提高社会养老服务装备水平，鼓励研发养老护理专业设备、辅具，积极推动养老服务专用车配备。
　　加强养老服务信息化建设，依托现代技术手段，为老年人提供高效便捷的服务，规范行业管理，不断提高养老服务水平。
　　（三）建设方式。
　　通过新建、扩建、改建、购置等方式，因地制宜建设养老服务设施。新建小区要统筹规划，将养老服务设施建设纳入公建配套实施方案。鼓励通过整合、置换或转变用途等方式，将闲置的医院、企业、农村集体闲置房屋以及各类公办培训中心、活动中心、疗养院、小旅馆、小招待所等设施资源改造用于养老服务。通过设备和康复辅具产品研发、养老服务专用车配备和信息化建设，全面提升社会养老服务能力。
　　（四）运行机制。
　　充分发挥市场在资源配置中的基础性作用，为各类服务主体营造平等参与、公平竞争的环境，实现社会养老服务可持续发展。
　　公办养老机构应充分发挥其基础性、保障性作用。按照国家分类推进事业单位改革的总体思路，理顺公办养老机构的运行机制，建立责任制和绩效评价制度，提高服务质量和效率。
　　鼓励有条件或新建的公办养老机构实行公建民营，通过公开招投标选定各类专业化的机构负责运营。负责运营的机构应坚持公益性质，通过服务收费、慈善捐赠、政府补贴等多种渠道筹集运营费用，确保自身的可持续发展。
　　加强对非营利性社会办养老机构的培育扶持，采取民办公助等形式，给予相应的建设补贴或运营补贴，支持其发展。鼓励民间资本投资建设专业化的服务设施，开展社会养老服务。
　　推动社会专业机构以输出管理团队、开展服务指导等方式参与养老服务设施运营，引导养老机构向规模化、专业化、连锁化方向发展。鼓励社会办养老机构收养政府供养对象，共享资源，共担责任。
　　（五）资金筹措。
　　社会养老服务体系建设资金需多方筹措，多渠道解决。
　　要充分发挥市场机制的基础性作用，通过用地保障、信贷支持、补助贴息和政府采购等多种形式，积极引导和鼓励企业、公益慈善组织及其他社会力量加大投入，参与养老服务设施的建设、运行和管理。
　　地方各级政府要切实履行基本公共服务职能，强化在社会养老服务体系建设中的支出责任，安排财政性专项资金，支持公益性养老服务设施建设。
　　民政部本级福利彩票公益金及地方各级彩票公益金要增加资金投入，优先保障社会养老服务体系建设。
　　中央设立专项补助投资，依据各地经济社会发展水平、老龄人口规模等，积极支持地方社会养老服务体系发展，重点用于社区日间照料中心和老年养护机构设施建设。
　　**五、保障措施**
　　（一）强化统筹规划，加强组织领导。从构建社会主义和谐社会的战略高度，充分认识加强社会养老服务体系建设的重要意义，增强使命感、责任感和紧迫感，将社会养老服务体系建设摆上各级政府的重要议事日程和目标责任考核范围，纳入经济社会发展规划，切实抓实抓好。各地要建立由民政、发展改革、老龄部门牵头，相关部门参与的工作机制，加强组织领导，加强协调沟通，加强对规划实施的督促检查，确保规划目标的如期实现。鼓励社会各界对规划实施进行监督。
　　（二）加大资金投入，建立长效机制。对公办养老机构保障所需经费，应列入财政预算并建立动态保障机制。采取公建民营、委托管理、购买服务等多种方式，支持社会组织兴办或者运营的公益性养老机构。鼓励和引导金融机构在风险可控和商业可持续的前提下，创新金融产品和服务方式，改进和完善对社会养老服务产业的金融服务，增加对养老服务企业及其建设项目的信贷投入。积极探索拓展社会养老服务产业市场化融资渠道。积极探索采取直接补助或贴息的方式，支持民间资本投资建设专业化的养老服务设施。
　　（三）加强制度建设，确保规范运营。建立、健全相关法律法规，建立养老服务准入、退出、监管制度，加大执法力度，规范养老服务市场行为。制定和完善居家养老、社区养老服务和机构养老服务的相关标准，建立相应的认证体系，大力推动养老服务标准化，促进养老服务示范活动深入开展。建立养老机构等级评定制度。建立老年人入院评估、养老服务需求评估等评估制度。
　　（四）完善扶持政策，推动健康发展。各级政府应将社会养老服务设施建设纳入城乡建设规划和土地利用规划，合理安排，科学布局，保障土地供应。符合条件的，按照土地划拨目录依法划拨。研究制定财政补助、社会保险、医疗等相关扶持政策，贯彻落实好有关税收以及用水、用电、用气等优惠政策。有条件的地方，可以探索实施老年护理补贴、护理保险，增强老年人对护理照料的支付能力。支持建立老年人意外伤害保险制度，构建养老服务行业风险合理分担机制。建立科学合理的价格形成机制，规范服务收费项目和标准。
　　（五）加快人才培养，提升服务质量。加强养老服务职业教育培训，有计划地在高等院校和中等职业学校增设养老服务相关专业和课程，开辟养老服务培训基地，加快培养老年医学、护理、营养和心理等方面的专业人才，提高养老服务从业人员的职业道德、业务技能和服务水平。如养老机构具有医疗资质，可以纳入护理类专业实习基地范围，鼓励大专院校学生到各类养老机构实习。加强养老服务专业培训教材开发，强化师资队伍建设。推行养老护理员职业资格考试认证制度，五年内全面实现持证上岗。完善培训政策和方法，加强养老护理员职业技能培训。探索建立在养老服务中引入专业社会工作人才的机制，推动养老机构开发社工岗位。开展社会工作的学历教育和资格认证。支持养老机构吸纳就业困难群体就业。加快培育从事养老服务的志愿者队伍，实行志愿者注册制度，形成专业人员引领志愿者的联动工作机制。
　　（六）运用现代科技成果，提高服务管理水平。以社区居家老年人服务需求为导向，以社区日间照料中心为依托，按照统筹规划、实用高效的原则，采取便民信息网、热线电话、爱心门铃、健康档案、服务手册、社区呼叫系统、有线电视网络等多种形式，构建社区养老服务信息网络和服务平台，发挥社区综合性信息网络平台的作用，为社区居家老年人提供便捷高效的服务。在养老机构中，推广建立老年人基本信息电子档案，通过网上办公实现对养老机构的日常管理，建成以网络为支撑的机构信息平台，实现居家、社区与机构养老服务的有效衔接，提高服务效率和管理水平。加强老年康复辅具产品研发。
　　各地可根据本规划，结合实际，制定本地区的社会养老服务体系建设规划。

**中共教育部党组关于加强和改进新形势下离退休干部工作的意见**

教党〔2011〕19号

　一、深刻认识新形势下离退休干部工作的重要意义

　　离退休干部是党和国家的宝贵财富，是促进教育事业科学发展的宝贵资源。离退休干部工作是党的组织工作、干部工作的重要组成部分，是社会主义和谐社会建设的一个重要方面。全面做好离退休干部工作，是党的十七大提出的目标要求和政治任务，是部机关各级党组织和各级领导干部应尽的政治责任，也是全面贯彻落实教育规划纲要的重要保障。各司局要进一步提高认识，自觉把离退休干部工作放到推进教育事业科学发展的全局中进行谋划，纳入教育改革发展的总体部署组织安排，作为党建工作的重要任务推进实施，努力把我部离退休干部工作提高到一个新的水平。

　　近年来，我部离退休干部工作取得了长足发展和显著成绩，形成了“围绕中心、服务大局”的良好风气和局面。离退休干部队伍思想稳定、精神状态良好，为办好人民满意的教育和建设社会主义和谐社会发挥了重要作用。随着经济社会发展和人口老龄化进程加快，我部离退休干部数量不断增多，人员结构发生较大变化，离休干部越来越少，退休干部越来越多，整个队伍呈现出基数大、增速快、高龄空巢病号多等特点。这些新的情况和特点对我部离退休干部工作提出了新的更高要求。

　　二、进一步加强和改进离退休干部工作的总体要求

　　加强和改进新形势下的离退休干部工作，要坚持以马克思列宁主义、毛泽东思想、邓小平理论和“三个代表”重要思想为指导，深入贯彻落实科学发展观。要坚持以人为本、重在服务、各方参与、务实创新的工作方针，围绕中心、服务大局，努力推动教育事业科学发展，促进社会和谐。要坚持从离退休干部队伍的实际出发，关心照顾好离退休干部的生活，不断加强思想政治建设，用马克思主义中国化的最新成果武装广大离退休干部。要坚持把老有所养和老有所为结合起来，引导离退休干部在贯彻实施教育规划纲要、关心教育下一代、建设人力资源强国等方面发挥积极作用。要坚持以让党放心、广大离退休干部满意为标准，不断提高服务管理水平，努力形成部党组统一领导，离退休干部局具体负责，各业务司局积极参与，全体党员干部共同关心，互相配合、齐抓共管的工作格局和机制。

　　三、建立健全离退休干部工作制度和工作机制

　　1.建立分工联系和走访慰问制度。部党组成员和离退休干部局、老同志原工作司局负责同志要分工联系并定期走访慰问离退休干部，主动沟通情况，听取意见建议。在重要节日、老同志大寿、病危住院、去世送别时，分工联系的部领导及相关部门负责人要亲自到场，把党和政府的关怀送到老同志身边。各司局党支部应负起责任，将此项工作作为日常党务工作的一项重要任务，列入年度基层党组织工作计划，指定专门联系人，定期走访、定期座谈，并将工作情况列入年度考核内容。

　　2.坚持完善政治理论学习和情况通报制度。认真落实离退休干部阅读文件、听报告、参加重要会议和重大活动、以及就近参观学习等制度，切实保障离退休干部相关政治待遇。通过举办“离退休干部大讲堂”等形式，定期邀请有关领导和专家向离退休干部通报情况，并针对他们共同关心的热点问题，做好释疑解惑工作，使广大老同志及时了解党的方针政策、国际国内形势以及经济社会发展和教育改革发展的新形势、新情况、新变化，进而在思想上、政治上与党中央保持一致，做到政治坚定、思想常新、理想永存、豪情满怀。

　　3.坚持离退休干部党建会和评优表彰制度。要开好一年一次的老同志党建工作会议，认真总结研究加强离退休干部党建和思想政治工作的经验和做法。要以创先争优活动为载体，积极推进学习型党支部建设和“五好支部”、“四好党员”争创工作。要建立和完善评优表彰制度，每两年评选表彰一次离退休干部先进党支部和优秀共产党员、优秀党务工作者。

　　4.健全完善离退休经费保障机制。认真落实离退休干部各项生活待遇，确保离退休费按时足额发放和医药费按规定报销。针对离休干部整体进入“双高期”和退休干部不断增多的实际，要进一步加强医疗保健和生活服务工作，强化健康体检和保健教育，充分发挥教育系统医疗卫生资源优势，为离退休干部看病就医创造条件。要根据离退休干部的实际需要，努力拓宽服务内涵，提高针对性和实效性。

　　5.探索建立成果共享机制。在研究制定涉及老同志切身利益的改革方案，特别是调整住房、提高福利待遇等，要充分考虑离退休干部的实际情况，使离退休干部的物质和精神文化生活水平得到相应改善和提高，共享改革发展稳定带来的实际利益。要进一步加大投入，加强离退休干部活动中心建设，更好地满足老同志的精神文化需求。

　　6.建立完善困难帮扶机制。要设立专项经费，解决离退休干部医疗生活特殊困难。要动员广大共产党员、机关干部开展爱心活动，积极筹措资金，对家庭收入低、身患重疾、孤寡空巢失能，以及因病致贫等困难人员给予特殊关照和适当补助，使他们感受到党组织的温暖和社会主义制度的优越。要支持工会、共青团等组织开展助老志愿者活动，鼓励低龄健朗离退休干部关心帮助高龄孤寡空巢人员，形成邻里之间互相关心、互相帮助的良好氛围。要加强与老同志所在社区联系，整合资源，为离退休干部居家养老提供帮助。

　　7.充分发挥离退休干部的作用。要根据离退休干部的身体状况、志趣爱好和专业特长，本着自觉自愿、量力而行的原则，创造条件、搭建平台、提供服务，引导离退休干部关心支持国家建设和教育改革发展，为实施教育规划纲要做贡献。要积极探索“老有所养”与“老有所为”有机结合的途径和方法，使离退休干部在服务社会、服务他人、有所作为中感受快乐，愉悦身心，益寿延年。

　　四、切实加强对离退休干部工作的组织领导

　　1.成立教育部离退休干部工作领导小组。由部党组书记、部长任组长，分管离退休干部工作的副部长任副组长，成员包括办公厅、人事司、财务司、机关党委、离退休干部局、机关服务中心等部门的主要负责人以及离退休干部代表。领导小组要及时研究离退休干部工作，协调解决有关困难，督促检查工作的落实情况。各司局要确定一名办公室负责人作为联系人，加强与离退休干部局的联系沟通，协助做好本司局离退休干部的相关工作。

　　2.进一步加强离退休干部工作队伍建设。认真落实中央机构编制委员会关于我部离退休干部局机构、人员编制等方面的规定，保证必要的经费和条件，使之与担负的任务相适应。要按照政治素质好、工作能力强、作风过得硬、对离退休干部有感情的要求，选好配强领导班子。要关心离退休干部局工作人员的工作、学习和生活，努力为其成长进步创造条件。要采取倾斜措施推进干部轮岗和交流工作，建立形成进得来、出得去的良好机制。要把离退休干部部门作为培养、锻炼干部的基地。

　　3.建立和完善领导责任制，加强责任考核。要建立和完善激励机制，对表现突出的离退休干部工作先进单位和个人，以及敬老爱老助老先进典型，及时进行表彰和宣传，促进形成全机关尊重关爱老同志、关心支持离退休干部工作的良好风气和氛围。

**国务院关于印发国家人口发展“十二五”规划的通知**

| 一、“十二五”时期人口发展形势  （一）“十一五”时期人口发展的成就。  生育水平继续保持稳定。人口计生工作思路方法不断创新，更加注重利益导向，更加注重服务关怀，更加注重宣传倡导，稳定低生育水平长效机制基本建立。以计划生育家庭奖励扶助制度、“少生快富”工程和特别扶助制度为主体的利益导向政策体系初步形成。计划生育优质服务和生育关怀行动普遍开展。婚育新风进万家活动、关爱女孩行动和新农村新家庭计划深入推进。妇女总和生育率稳定在1.8以下，人口自然增长率保持在6‰以内，2010年末总人口为13.41亿人。  人口素质稳步提高。科技、教育、人力资源、文化、卫生、人口计生事业不断发展。各级教育普及水平进一步提高，城乡免费九年义务教育全面实现，2010年高等教育毛入学率达到26.5%，15岁以上国民平均受教育年限达到9年，新增劳动力平均受教育年限达到12.4年。人才队伍建设成效显著，人才资源总量不断扩大。孕产妇和婴儿死亡率继续下降，人均预期寿命达到73.5岁。  人口城镇化水平继续提高。积极稳妥推进城镇化，促进农业转移人口转为城镇居民。2010年城镇人口6.7亿人，城镇化率达到50%。流动人口服务管理体制建设取得积极进展。  城乡就业规模持续扩大。积极就业政策取得成效，城乡就业形势总体稳定。城镇新增就业5771万人，转移农业劳动力4500万人，城镇登记失业率保持在4.3%以下。  民生建设取得新进展。城乡居民收入快速增长，生产生活条件得到改善。城镇居民基本医疗保险制度全面建立，新型农村合作医疗制度全面实施；新型农村社会养老保险试点启动并逐步扩大范围，全国统一的城镇企业职工基本养老保险关系转移接续办法出台，企业退休人员基本养老金不断提高；生育保险制度进一步完善。城乡居民最低生活保障全面加强，重点优抚对象抚恤优待标准大幅度提高。妇女和未成年人权益保护得到加强，残疾人参与社会生产生活的环境进一步改善，老年人社会保障和服务水平不断提高。减灾救灾和应急管理体制机制进一步健全。  （二）“十二五”时期人口发展的阶段性特征。  人口增长势头减弱。生育旺盛期妇女数量开始呈现下降态势，同时随着人口老龄化程度提高，人口死亡率有所上升，在生育政策不变的条件下，人口增长的势头进一步减弱。  主要劳动年龄人口达到峰值。“十二五”是我国人力资源最为丰富的时期，劳动年龄人口总量达到峰值，此后缓慢下行，人口抚养比在经历40多年下降后开始上升。  老年人口出现第一次增长高峰。20世纪50年代第一次生育高峰出生人口相继进入老年，“十二五”期间我国60岁以上老年人口年均增长800万以上，总量将突破2亿。  城镇人口历史性超过农村人口。城镇化率超过50%，城乡人口格局正在发生根本性变化。随着产业转移的加快、中西部城市群的发展，人口流动迁移呈现出新的特点，人口流向趋于多元化。  （三）面临的主要问题与矛盾。  人口数量对可持续发展的压力仍然较大。我国人口多、底子薄，资源相对不足、环境容量有限，发展不平衡。随着人口继续增长，人口与资源、环境以及经济社会发展的矛盾将进一步显现。劳动年龄人口规模庞大，解决就业问题仍将是长期而艰巨的任务。  人口素质正成为提升国家竞争力的瓶颈。国际产业分工的调整、经济发展方式的转变，对人口素质提出了更高的要求，但我国人口素质总体不高，人力资本对经济增长的贡献率远低于发达国家平均水平，正在成为影响我国国际竞争力的重要因素。  人口结构性矛盾成为影响经济社会发展的重大问题。我国出生人口性别比长期居高不下，老年人口比重不断提高，人口抚养比开始上升，区域间、城乡间人口发展不平衡，人口结构性矛盾对经济社会发展的影响日益深刻。  人口分布不合理影响城乡区域协调发展。人口空间分布与经济布局不协调，与资源环境承载力不适应。城镇化率偏低，流动人口规模庞大，对社会管理和公共服务等带来一系列挑战。  家庭结构变化带来新挑战。家庭规模小型化、结构多样化、成员居住分散化趋势明显，导致传统家庭功能弱化，在婚姻、生育、养老等方面出现诸多新问题，人口服务管理相对滞后。  总体上看，“十二五”时期是我国人口发展的重大转折期，人口发展的机遇与挑战并存。一方面，经济社会发展仍然面临着人口总量持续增加的压力，人口对经济社会、资源环境的影响更加突出；另一方面，人口各要素关系更趋复杂，素质、结构、分布正在成为影响发展的主要因素。必须从战略上重视人口问题，遵循人口发展规律，充分利用人力资源丰富、社会抚养比低、人口流动活跃的有利时机，全面做好人口工作，为经济社会发展营造良好的人口环境。  二、总体思路、基本原则和主要目标   （一）总体思路。  高举中国特色社会主义伟大旗帜，以邓小平理论和“三个代表”重要思想为指导，深入贯彻落实科学发展观，坚定不移走中国特色统筹解决人口问题的道路。按照全面做好人口工作的总体要求，以建设人口均衡型社会为主线，坚持计划生育基本国策，逐步完善政策，稳定低生育水平，提高人口素质，优化人口结构与分布，促进人口长期均衡发展，促进人口与经济社会、资源环境相协调。  （二）基本原则。  坚持以人为本。把促进人的全面发展作为处理好人口与发展关系的出发点和落脚点。在解决人口问题的过程中，注重保障和改善民生，提高家庭发展能力。  坚持统筹协调。强化人口的基础地位，注意把握人口各要素之间、人口与经济社会及资源环境之间的互动关系，构建有利于促进人口长期均衡发展的政策体系。  坚持科学指导。把握好人口与经济社会发展的阶段性特点，适时完善人口发展政策，指导地方积极探索统筹解决人口问题的有效方法和途径。  坚持创新发展。不断推进人口工作的理论创新、管理创新、服务创新、科技创新，通过体制机制创新，着力解决人口发展中出现的新情况、新问题。  （三）主要目标。  人口总量目标。低生育水平保持稳定，“十二五”期间，人口年均自然增长率控制在7.2‰以内，全国总人口控制在13.9亿人以内。  人口素质目标。教育普及水平进一步提高，15岁以上国民平均受教育年限达到9.3年，新增劳动力平均受教育年限达到13.3年；在职人员培训覆盖面进一步扩大，劳动力素质明显提高，人才队伍进一步壮大。婴儿死亡率降到12‰，孕产妇死亡率降到22/10万，严重多发致残的出生缺陷发生率降低，人均预期寿命达到74.5岁。  人口结构目标。出生人口性别结构得到有效改善，全国出生人口性别比下降至115以下。  人口分布目标。城镇化率提高约4个百分点，限制开发区域内人口更多地集聚在县城和中心镇，禁止开发区域内人口总量有所减少，人口空间分布趋于合理。流动人口基本公共服务均等化初步实现。  民生保障目标。社会就业更加充分，城镇登记失业率控制在5%以内，城乡居民收入大幅提高，家庭发展能力得到增强。覆盖城乡居民的社会保障体系建设加快推进，社会养老服务体系基本建立，养老服务能力明显提升。贫困人口显著减少，残疾人社会保障体系和服务体系框架基本建立，生活状况得到进一步改善。  三、主要任务   （一）坚持计划生育基本国策，稳定低生育水平。  全面做好人口和计划生育工作。保持生育政策的连续性、稳定性，更加注重利益引导，更加注重服务关怀，更加注重宣传倡导，全面加强基层基础工作，突出做好重点地区重点人群的人口计生工作。深化人口计生综合改革，着力创新稳定低生育水平、统筹解决人口问题的工作机制和方法，解决影响和制约人口计生工作科学发展的突出问题。坚持依法行政，完善相关法律法规。  完善稳定低生育水平相关经济社会政策。将稳定低生育水平的利益导向政策作为保障和改善民生的重要组成部分，纳入政府改善民生行动计划。全面落实法律法规规定的计划生育家庭奖励优惠政策。促进经济社会相关政策与人口计生政策的有机衔接，在就业、社会保障、扶贫开发、征地补偿、集体收益分配等方面，制定对计划生育家庭的倾斜政策。进一步完善以计划生育家庭奖励扶助制度、“少生快富”工程和特别扶助制度为主的优先优惠政策体系，扩大范围、提高扶助标准并建立动态调整机制。  完善人口和计划生育公共服务体系。加快人口计生服务体系建设，拓展服务范围。加强人口计生服务基础设施和信息服务配套设施建设。实施人口和计划生育职业体系建设工程，建立职业标准、教育培训、评估认证和职业拓展体系。加强计划生育和生殖健康科技创新基地建设，扶持一批部委级重点实验室。加强新型避孕药具的研究与开发，完善计划生育药具不良反应监测网络。全面提升计划生育优质服务能力，加强生殖健康保健，使人人享有生殖健康服务。  （二）完善人口政策，促进人口长期均衡发展。  深化人口长期均衡发展战略研究。按照科学发展观以及加快经济发展方式转变的要求，充分考虑人口变化对经济社会发展的长期性、基础性影响，准确把握人口变动趋势，深入研究不同阶段的人口政策取向及主要任务，为人口决策提供支撑。  坚持和完善现行生育政策。进一步完善宣传教育、依法管理、村（居）民自治、优质服务、政策推动、综合治理的长效工作机制，确保低生育水平的稳定。着眼长远发展，逐步完善政策，确保人口中长期战略目标的实现。  形成人口政策合力。进一步完善人口计生、人力资源开发、男女平等、人口老龄化以及人口迁移流动等方面政策，制定促进家庭发展、主体功能区建设等方面的人口配套政策，加快形成统筹解决人口问题的政策体系。  （三）着力提高人口素质，加快人口大国向人力资源强国转变。  提高出生人口素质。有关部门按照职责分工，加强协作，积极落实出生缺陷三级预防措施，加大出生缺陷干预力度。开展易为广大群众接受的宣传和引导活动，加强婚前、孕前咨询指导。组织实施计划生育生殖健康促进工程，做好健康教育、优生咨询、高危人群指导、孕前筛查、营养素补充等优生服务工作。逐步扩大国家免费孕前优生健康检查项目试点范围，探索建立国家免费孕前优生健康检查制度。建设全国产前诊断网络，推进新生儿疾病筛查、诊断和治疗工作。开展出生缺陷发生机理和防治技术研究，加强出生缺陷防治重点实验室建设。提高助产服务能力，进一步降低孕产妇和婴儿死亡率。  提高人口健康素质。普及健康教育，积极倡导健康文明的生活方式，大力推进全民健身运动。全面加强公共卫生服务体系建设，健全医疗保障和服务体系，完善基本医疗制度。充分利用基层医疗卫生和计划生育服务网络，形成以预防为主、防治结合的公共卫生服务体系，逐步缩小城乡居民基本公共卫生服务差距。继续加强性病、艾滋病的防治工作，全面实施慢性病综合防控，最大限度地控制和减少传染病、地方病的发生和传播。加强心理卫生和精神健康工作。  提升国民教育水平。贯彻落实《国家中长期教育改革和发展规划纲要（2010—2020年）》，深化教育体制改革，全面实施素质教育，大力促进教育公平，加快构建覆盖城乡的基本公共教育服务体系。巩固提高义务教育普及成果，全面提高质量和水平，促进义务教育均衡发展。加强婴幼儿早期教育。积极发展学前教育，构建“广覆盖、保基本、多形式”的学前教育体系。大力发展职业教育，巩固规模，提高质量，建立支撑现代产业发展的职业教育体系。全面提高高等教育质量，实现从以规模扩张为基本特征的外延式发展向以提高质量为核心的内涵式发展转变，办出具有中国特色、世界水平的现代高等教育。加快发展多样化的继续教育，重视和支持特殊教育，继续发展老年教育，推动民族教育加快发展。健全国家资助政策体系，扶助家庭经济困难学生完成学业。加强青少年健康人格教育、独生子女社会行为教育。加强道德素质和诚信教育，提高国民素养。  加强人才队伍建设。加大专业技术人才和高技能人才培养力度。优化人才培养结构，突出培养造就创新型科技人才，重视开发领军人才，大力开发经济社会发展急需紧缺专门人才，提高应用型、复合型、技能型人才培养规模和水平。建立和完善职业资格证书制度，对城乡有就业要求和培训愿望的劳动者参加职业技能培训给予培训费补贴，推进中等职业教育免费进程。加强农村实用人才的培养，提高农民工的就业能力。营造人才脱颖而出的环境，实施更加积极的就业政策，充分发挥劳动力资源丰富的优势，加快人口大国向人力资源强国转变，为经济社会发展提供持久动力。  （四）引导人口有序流动，促进人口合理布局。  优化人口布局。实施与主体功能区相配套的人口政策，促进形成合理的人口分布格局。积极推进资源环境承载能力较强、经济发达的城市化地区吸纳和集聚人口，引导人口超载的重要生态地区人口自愿、平稳、有序转移。深入开展人口空间分布研究，做好人口发展规划和主体功能区规划的衔接协调。研究促进人口有序流动、合理分布的政策，探索优化人口分布的有效途径。  积极稳妥推进城镇化。将符合条件的农业转移人口逐步转为城镇居民作为推进城镇化的重要任务。积极稳妥推进户籍管理制度改革，特大城市要合理控制人口规模，大中城市要加强和改进人口管理，继续发挥吸纳人口的重要作用，中小城市和小城镇要根据实际放宽落户条件。坚持因地制宜、分步推进，按规定把有合法稳定职业并有合法稳定住所（含租赁）的农村人口逐步转为城镇居民。构建城镇化战略格局，完善城镇基础设施，增强城镇承载能力，改善人居环境。统筹考虑人口变化和城镇化趋势，优化城乡公共服务设施布局。加快推进社会主义新农村建设，促进城乡经济社会发展一体化。  创新流动人口服务管理体制机制。建立全国流动人口生存发展状况、分布的动态监测体系。在国家综合配套改革试验区进行改革试点，积极探索符合时代要求的人口服务管理体制。逐步实行暂住人口居住证制度。在农民工流动较为集中的地区建立农民工综合服务中心，为流动人口提供“一站式”服务。建立农民工基本培训补贴制度，增加对农民工的技能培训和就业服务。将与企业建立稳定劳动关系的农民工纳入城镇职工基本养老和医疗保险。多渠道多形式改善农民工居住条件，鼓励采取多种方式将符合条件的农民工纳入城镇住房保障体系。坚持以输入地政府管理为主，以全日制公办中小学为主，确保进城务工人员随迁子女平等接受义务教育。研究制定进城务工人员随迁子女接受义务教育后在当地参加升学考试的办法。巩固完善流动人口计划生育“一盘棋”工作机制，建立流动人口计划生育基本公共服务均等化的保障机制。  （五）健全养老保障和服务体系，积极应对人口老龄化。  探索具有中国特色的应对人口老龄化新路子。深化应对人口老龄化战略研究，实施积极应对人口老龄化战略，制定与其他经济社会政策互相衔接、相互支撑的应对人口老龄化政策体系，推进老年人权益保障法制化进程。提高老年人口的素质和技能，充分开发老年人力资源，鼓励老年人参与经济社会活动。加强组织领导，健全党政主导、老龄委协调、部门尽责、社会参与、全民关怀的大老龄工作格局。  完善养老保障制度。以基本养老保险为重点，加快完善城乡居民社会养老保障制度。实现新型农村社会养老保险制度和城镇居民社会养老保险制度全覆盖，完善城镇职工基本养老保险制度，全面落实城镇职工基本养老保险省级统筹，实现基础养老金全国统筹，切实做好城镇职工基本养老保险关系接续工作。城镇参加基本养老保险人数达到3.57亿人，60岁以上非就业城镇居民全部享受基础养老金待遇。鼓励有条件的地区在养老保险基础上，进一步加强养老保障工作，积极探索为独生子女父母、无子女和失能老人提供必要的养老服务补贴和老年护理补贴。  加快养老服务体系建设。注重发挥家庭和社区功能，建立以居家为基础、社区为依托、机构为支撑的社会养老服务体系，每千名老人拥有养老床位达到30张。加强养老服务标准、行业规范和管理制度建设，建立养老机构准入、退出与监管机制。优先发展社会养老服务，加强老年人宜居环境建设，增加社区老年活动场所和便利设施。  （六）综合治理出生人口性别比偏高问题，促进社会性别平等。  坚持男女平等基本国策。提高社会性别平等意识，清理涉及社会性别歧视的法规政策。指导村（居）民自治组织修订完善自治章程或村规民约，在扶贫济困、慈善救助、贴息贷款、就业服务、项目扶持、村集体收益分配等方面对计划生育家庭女儿户予以倾斜。深入推进关爱女孩行动，进一步开展“幸福工程”和“春蕾计划”等社会公益性活动，充分发挥社会组织在贫困母亲救助和女童健康成长帮助中的重要作用。  推动妇女儿童事业全面发展。贯彻《国务院关于印发中国妇女发展纲要和中国儿童发展纲要的通知》（国发〔2011〕24号），切实保障妇女合法权益，加强未成年人保护。促进妇女就业创业，提高妇女参与经济发展和社会管理能力。严厉打击暴力侵害妇女、拐卖妇女儿童、弃婴等违法犯罪行为。切实解决流动儿童、留守儿童、孤残儿童和流浪未成年人救助等问题。  加强出生人口性别比综合治理。建立健全国家和省级部门间协调机制，把促进社会性别平等、综合治理出生人口性别比偏高问题列入地方各级人口和计划生育领导小组成员单位的职责范围。规范人工终止妊娠药品和计划生育手术器械经营管理，完善医学需要的胎儿性别鉴定和人工终止妊娠登记、孕产期全程服务管理制度。严厉打击非医学需要的胎儿性别鉴定和选择性别人工终止妊娠行为。完善出生统计监测体系，全面实施出生实名登记制度，建立部门间出生人口信息收集和共享机制。强化区域协作和重点区域的治理，在出生人口性别比严重偏高的连片地区，建立区域协查制度。在重点区域探索建立跨省区出生人口信息共享制度和出生性别监测预警机制。  （七）提高家庭发展能力，促进家庭和谐幸福。  建立健全家庭发展政策。稳定家庭功能，在优生优育、家庭教育、子女成才、抵御风险、生殖健康、家庭致富以及养老保障等方面，加快建立和完善提高家庭发展能力的政策体系。加大对孤儿监护人家庭、老年人家庭、残疾人家庭、留守家庭、流动家庭、受灾家庭和其他特殊困难家庭的扶持力度。进一步完善工伤、生育等保险制度，合理提高低保标准和补助水平，加强城乡社会救助体系建设，积极发展社会福利和慈善事业。提高住房保障水平。完善生育关怀制度，将计划生育节育手术并发症三级以上人员纳入计划生育家庭特别扶助制度范围。免费为符合条件的育龄妇女提供再生育技术服务。研究出台有利于促进计划生育家庭成员就业、创业、勤劳致富的扶持政策。  着力提高家庭服务能力。大力发展家庭服务业，逐步建立比较健全的惠及城乡居民的家庭服务体系。在全员人口信息系统中，加强家庭信息采集和管理，为家庭发展政策的制定和实施提供依据。探索建立以家庭为中心的人口计生公共服务体系，开展婚育指导、家庭初级保健、儿童早期发展、家庭教育指导，以及对计划生育家庭、空巢家庭、流动家庭、留守家庭的关怀服务等。  大力推进新型家庭人口文化建设。以婚育新风进万家活动、关爱女孩行动、新农村新家庭计划、幸福工程为载体，广泛宣传人口计生政策。开展幸福家庭创建活动。倡导婚姻自由平等、生殖健康、优生优育、社会性别平等的观念，倡导积极健康、负责任的婚育行为，倡导低消耗、低污染的家庭生活方式。大力弘扬尊老爱幼、邻里互助的社会风尚。加强流动人口聚集区多元文化交流，促进流动人口融入当地社会。加强农村人口文化设施建设，纳入新农村建设整体规划。加强少数民族人口文化建设。  （八）健全残疾人保障和服务体系，支持残疾人事业发展。  健全残疾人社会保障体系。鼓励残疾人普遍加入基本医疗保险和基本养老保险，逐步将残疾人基本康复医疗按规定纳入基本医疗保障范围。将符合条件的残疾人家庭优先纳入基本住房保障制度，继续实施“阳光安居”农村贫困残疾人家庭危房改造工程。逐步建立贫困残疾人生活补助和重度残疾人护理补贴制度。支持残疾人辅助器具的研发、生产和适配服务，制定政府补贴的残疾人基本型辅助器具目录。  完善残疾人服务体系。实施重点康复工程，为贫困残疾人提供康复服务。全面开展社区康复工作，推进残疾人“人人享有康复服务”。制定实施国家残疾预防行动计划。继续实施“阳光家园计划”。健全残疾人托养服务体系，大力发展居家助残服务。积极开展无障碍建设。实施残疾人文化建设和自强健身工程，丰富残疾人精神文化生活。  促进残疾人就学就业。改善特殊教育办学条件，逐步实行残疾学生高中阶段免费教育，加大对家庭困难残疾学生的资助力度。为残疾人免费提供公共就业服务，对残疾人参加职业技能培训和职业技能鉴定的，按规定给予补贴。完善残疾人就业促进和保护政策措施，加强残疾人就业援助，积极开发适合残疾人的公共就业岗位。加强对农村残疾人的生活救助和生产扶助。  四、主要保障措施   （一）加强组织领导和统筹协调。各地区、各有关部门要把统筹解决人口问题、建设人口均衡型社会列入重要议事日程，把人口计生工作列入经济社会发展总体规划，纳入改善民生的总体部署。构建党委领导、政府负责、社会协同、公众参与的人口计生工作格局，健全领导机制、协调机制、监督机制和问责机制，坚持人口计生工作党政一把手亲自抓、负总责，完善目标管理责任制和“一票否决”制度。坚持人口发展规划先行，其他专项规划制定应充分考虑人口因素影响；重大经济社会政策出台前，应开展对人口发展影响的评估。  （二）健全人口监测体系。建设国家人口基础信息库，实施“全员人口统筹管理信息系统”工程，建立人口信息动态采集和更新机制，科学监测和评估人口发展状况。构建科学的人口发展指标体系，规范人口统计口径。建立完善部门间人口信息共享制度和人口统计信息沟通机制，及时发布人口总量、结构、分布预测、预警信息，为科学决策、人口综合服务管理提供信息支撑。  （三）完善人口服务管理体系。完善人口计生服务管理体系，优化公共资源配置，以全员人口信息为基础，构建人口管理和公共服务平台。充分发挥人口和计划生育公共服务网络的作用，探索建立社区人口和家庭服务中心。加强人口计生队伍建设，完善职业体系，造就一支高素质的人口管理和服务人才队伍。  （四）建立健全投入保障机制。建立“财政为主、稳定增长、分类保障、分级负担、城乡统筹”的人口和计划生育投入保障机制，确保人口和计划生育财政投入增长幅度高于经常性财政收入增长幅度；区别不同重大项目，分别确定中央和地方资金的分担办法；按照“费随人走”的原则，以常住人口为依据进行中央财政对地方的转移支付；完善绩效挂钩、以奖代投（补）激励机制。鼓励民间资本投入人口发展领域。进一步加大对革命老区、民族地区、边疆地区、贫困地区人口发展的政策支持和资金扶持力度。  （五）加大宣传与国际交流力度。深入开展人口国情、人口政策和人口发展规划的宣传，充分发挥新闻媒体作用，正确引导舆论，在全社会营造有利于人口计生事业发展的氛围。发挥中国计划生育协会等社会团体作用，动员社会力量共同做好人口计生工作。建立和完善国际交流与合作机制，积极参与、引导有关国际规则的制定。  （六）加强规划实施的监测评估。地方各级人民政府、各有关部门共同做好人口发展规划的实施工作，制定本地区、本部门的具体实施方案和年度计划。加强对规划实施情况的动态监测与跟踪分析，及时发现和解决规划执行过程中存在的问题，定期组织对规划实施情况的检查，开展规划执行情况期中和期末评估，确保规划各项任务落到实处。 |
| --- |

**国务院关于印发中国老龄事业发展
“十二五”规划的通知**

　　为积极应对人口老龄化，加快发展老龄事业，根据《中华人民共和国国民经济和社会发展第十二个五年规划纲要》、《中华人民共和国老年人权益保障法》和《中共中央　国务院关于加强老龄工作的决定》（中发〔2000〕13号），制定本规划。

　　一、背景

　　（一）“十一五”期间取得的主要成就。

　　“十一五”时期是老龄事业快速发展的五年。养老保障体系逐步完善，覆盖范围进一步扩大，企业职工基本养老保险制度实现全覆盖，企业退休人员养老金水平连续五年提高，基本养老保险实现了省级统筹，新型农村社会养老保险开始试点并逐步扩大范围。职工和城镇居民基本医疗保险制度实现全覆盖，新型农村合作医疗参合率稳步提高。老年社会福利和社会救助制度逐步建立，城乡计划生育家庭养老保障支持政策逐步形成。老龄服务体系建设扎实推进,在城市深入开展并逐步向农村延伸，养老服务机构和老年活动设施建设取得较大进步。老年教育、文化、体育事业较快发展，老年精神文化生活更加丰富。全社会老龄意识明显增强，敬老爱老助老社会氛围日益浓厚，老年人权益得到较好保障。老龄领域的科学研究、国际交流与合作取得了新的进展。广大老年群众坚持老有所为，积极参与经济社会建设和公益活动，在构建社会主义和谐社会中发挥了重要作用。

　　（二）“十二五”时期老龄事业面临的形势。

　　“十二五”时期是我国全面建设小康社会的关键时期，也是老龄事业发展的重要机遇期。

　　长期以来，党和政府十分关心老年群众，不断采取积极措施，推动老龄事业发展进步，取得举世瞩目的成就，为老龄事业持续发展奠定了很好的基础。但是，在快速发展的老龄化进程中，老龄事业和老龄工作相对滞后的矛盾日益突出。主要表现在：社会养老保障制度尚不完善，公益性老龄服务设施、服务网络建设滞后，老龄服务市场发育不全、供给不足，老年社会管理工作相对薄弱，侵犯老年人权益的现象仍时有发生。对此，我们必须高度重视，认真解决。

　　“十二五”时期，随着第一个老年人口增长高峰到来，我国人口老龄化进程将进一步加快。从2011年到2015年，全国60岁以上老年人将由1.78亿增加到2.21亿，平均每年增加老年人860万；老年人口比重将由13.3%增加到16%，平均每年递增0.54个百分点。老龄化进程与家庭小型化、空巢化相伴随，与经济社会转型期的矛盾相交织，社会养老保障和养老服务的需求将急剧增加。未来20年，我国人口老龄化日益加重，到2030年全国老年人口规模将会翻一番，老龄事业发展任重道远。我们必须深刻认识发展老龄事业的重要性和紧迫性，充分利用当前经济社会平稳较快发展和社会抚养比较低的有利时机，着力解决老龄工作领域的突出矛盾和问题，从物质、精神、服务、政策、制度和体制机制等方面打好应对人口老龄化挑战的基础。

　　二、指导思想、发展目标和基本原则

　　（一）指导思想。

　　高举中国特色社会主义伟大旗帜，以邓小平理论和“三个代表”重要思想为指导，深入贯彻落实科学发展观，适应人口老龄化新形势，以科学发展为主题，以改革创新为动力，建立健全老龄战略规划体系、社会养老保障体系、老年健康支持体系、老龄服务体系、老年宜居环境体系和老年群众工作体系，服务经济社会改革发展大局，努力实现老有所养、老有所医、老有所教、老有所学、老有所为、老有所乐的工作目标，让广大老年人共享改革发展成果。

　　（二）主要发展目标。

　　——建立应对人口老龄化战略体系基本框架，制定实施老龄事业中长期发展规划。

　　——健全覆盖城乡居民的社会养老保障体系，初步实现全国老年人人人享有基本养老保障。

　　——健全老年人基本医疗保障体系，基层医疗卫生机构为辖区内65岁及以上老年人开展健康管理服务，普遍建立健康档案。

　　——建立以居家为基础、社区为依托、机构为支撑的养老服务体系，居家养老和社区养老服务网络基本健全，全国每千名老年人拥有养老床位数达到30张。

　　——全面推行城乡建设涉老工程技术标准规范、无障碍设施改造和新建小区老龄设施配套建设规划标准。

　　——增加老年文化、教育和体育健身活动设施，进一步扩大各级各类老年大学（学校）办学规模。

　　——加强老年社会管理工作。各地成立老龄工作委员会，80%以上退休人员纳入社区管理服务对象，基层老龄协会覆盖面达到80%以上，老年志愿者数量达到老年人口的10%以上。

　　（三）基本原则。

　　1.老龄事业与经济社会发展相适应。紧紧围绕全面建设小康社会和构建社会主义和谐社会宏伟目标，确立老龄事业在改革发展大局中的重要地位，促进老龄事业与经济社会协调发展。

　　2.立足当前与着眼长远相结合。从我国的基本国情出发，把着力解决当前的突出矛盾和应对人口老龄化长期挑战紧密联系，注重体制机制创新和法规制度建设，统筹兼顾，综合施策，实现全面、协调、可持续发展。

　　3.政府引导与社会参与相结合。按照社会主义市场经济的要求，积极发展老龄服务业。加强政策指导、资金支持、市场培育和监督管理，发挥市场机制在资源配置上的基础性作用，充分调动社会各方面力量积极参与老龄事业发展。

　　4.家庭养老与社会养老相结合。充分发挥家庭和社区功能，着力巩固家庭养老地位，优先发展社会养老服务，构建居家为基础、社区为依托、机构为支撑的社会养老服务体系，创建中国特色的新型养老模式。

　　5.统筹协调与分类指导相结合。注重城乡、区域协调发展，加大对农村和中西部地区的政策支持力度，资源配置向基层、特别是农村和中西部地区倾斜。充分发挥各地优势和群众的创造性，因地制宜地开展老龄工作，发展老龄事业。

　　6.道德规范与法律约束相结合。广泛开展孝亲敬老道德教育，加强老龄法制工作，为老龄工作和老龄事业的全面发展提供动力和保证。

　　三、主要任务

　　（一）老年社会保障。

　　1.加快推进养老保险制度建设。实现新型农村社会养老保险和城镇居民养老保险制度全覆盖。完善实施城镇职工基本养老保险制度，全面落实城镇职工基本养老保险省级统筹，实现基础养老金全国统筹，做好城镇职工基本养老保险关系转移接续工作。逐步推进城乡养老保障制度有效衔接，推动机关事业单位养老保险制度改革。建立随工资增长、物价上涨等因素调整退休人员基本养老金待遇的正常机制。发展企业年金和职业年金。发挥商业保险补充性作用。

　　2.完善基本医疗保险制度。进一步完善职工基本医疗保险、城镇居民基本医疗保险、新型农村合作医疗制度。逐步提高城镇居民医保和新农合人均筹资标准及保障水平，减轻老年人等参保人员的医疗费用负担。提高职工医保、城镇居民医保、新农合基金最高支付限额和政策范围内住院费用支付比例，全面推进门诊统筹。做好各项制度间的衔接，逐步提高统筹层次，加快实现医保关系转移接续和医疗费用异地就医结算。全面推进基本医疗费用即时结算，改革付费方式。积极发展商业健康保险，完善补充医疗保险制度。

　　3.加大老年社会救助力度。完善城乡最低生活保障制度，将符合条件的老年人全部纳入最低生活保障范围。根据经济社会发展水平，适时调整最低生活保障和农村五保供养标准。完善城乡医疗救助制度，着力解决贫困老年人的基本医疗保障问题。完善临时救助制度，保障因灾因病等支出性生活困难老年人的基本生活。

　　4.完善老年社会福利制度。积极探索中国特色社会福利的发展模式，发展适度普惠型的老年社会福利事业，研究制定政府为特殊困难老年人群购买服务的相关政策。进一步完善老年人优待办法，积极为老年人提供各种形式的照顾和优先、优待服务，逐步提高老年人的社会福利水平。有条件的地方可发放高龄老年人生活补贴和家庭经济困难的老年人养老服务补贴。

　　（二）老年医疗卫生保健。

　　1.推进老年医疗卫生服务网点和队伍建设。将老年医疗卫生服务纳入各地卫生事业发展规划，加强老年病医院、护理院、老年康复医院和综合医院老年病科建设，有条件的三级综合医院应当设立老年病科。基层医疗卫生机构积极开展老年人医疗、护理、卫生保健、健康监测等服务，为老年人提供居家康复护理服务。基层医疗卫生机构应加强人员队伍建设，切实提高开展老年人卫生服务的能力。

　　2.开展老年疾病预防工作。基层医疗卫生机构要为辖区内65岁及以上老年人开展健康管理服务，建立健康档案。组织老年人定期进行生活方式和健康状况评估，开展体格检查，及时发现健康风险因素，促进老年疾病早发现、早诊断和早治疗。开展老年疾病防控知识的宣传，做好老年人常见病、慢性病的健康指导和综合干预。

　　3.发展老年保健事业。广泛开展老年健康教育，普及保健知识，增强老年人运动健身和心理健康意识。注重老年精神关怀和心理慰藉，提供疾病预防、心理健康、自我保健及伤害预防、自救等健康指导和心理健康指导服务，重点关注高龄、空巢、患病等老年人的心理健康状况。鼓励为老年人家庭成员提供专项培训和支持，充分发挥家庭成员的精神关爱和心理支持作用。老年性痴呆、抑郁等精神疾病的早期识别率达到40%。

　　（三）老年家庭建设。

　　1.改善老年人居住条件。引导开发老年宜居住宅和代际亲情住宅，鼓励家庭成员与老年人共同生活或就近居住。推动和扶持老年人家庭无障碍改造。

　　2.完善家庭养老支持政策。完善老年人口户籍迁移管理政策，为老年人随赡养人迁徙提供条件。健全家庭养老保障和照料服务扶持政策，完善农村计划生育家庭奖励扶助制度和计划生育家庭特别扶助制度，落实城镇独生子女父母年老奖励政策，建立奖励扶助金动态调整机制。

　　3.弘扬孝亲敬老传统美德。强化尊老敬老道德建设，提倡亲情互助，营造温馨和谐的家庭氛围，发挥家庭养老的基础作用。努力建设老年温馨家庭，提高老年人居家养老的幸福指数。

　　（四）老龄服务。

　　1.重点发展居家养老服务。建立健全县（市、区）、乡镇（街道）和社区（村）三级服务网络，城市街道和社区基本实现居家养老服务网络全覆盖；80%以上的乡镇和50%以上的农村社区建立包括老龄服务在内的社区综合服务设施和站点。加快居家养老服务信息系统建设，做好居家养老服务信息平台试点工作，并逐步扩大试点范围。培育发展居家养老服务中介组织，引导和支持社会力量开展居家养老服务。鼓励社会服务企业发挥自身优势，开发居家养老服务项目，创新服务模式。大力发展家庭服务业，并将养老服务特别是居家老年护理服务作为重点发展任务。积极拓展居家养老服务领域，实现从基本生活照料向医疗健康、辅具配置、精神慰藉、法律服务、紧急救援等方面延伸。

　　2.大力发展社区照料服务。把日间照料中心、托老所、星光老年之家、互助式社区养老服务中心等社区养老设施，纳入小区配套建设规划。本着就近、就便和实用的原则，开展全托、日托、临托等多种形式的老年社区照料服务。

　　3.统筹发展机构养老服务。按照统筹规划、合理布局的原则，加大财政投入和社会筹资力度，推进供养型、养护型、医护型养老机构建设。积极推进养老机构运营机制改革与完善，探索多元化、社会化的投资建设和管理模式。进一步完善和落实优惠政策，鼓励社会力量参与公办养老机构建设和运行管理。“十二五”期间，新增各类养老床位342万张。

　　4.优先发展护理康复服务。在规划、完善医疗卫生服务体系和社会养老服务体系中，加强老年护理院和康复医疗机构建设。政府重点投资兴建和鼓励社会资本兴办具有长期医疗护理、康复促进、临终关怀等功能的养老机构。根据《护理院基本标准》加强规范管理。地（市）级以上城市至少要有一所专业性养老护理机构。研究探索老年人长期护理制度，鼓励、引导商业保险公司开展长期护理保险业务。

　　5.切实加强养老服务行业监管。进一步完善养老机构行政管理的法律法规，建立养老机构准入、退出与监管制度，做好养老机构登记注册和日常检查、监督管理工作。寄宿制养老机构等关系老年人安全和健康的重要场所，要列入消防安全和卫生许可制度重点管理范围。

　　（五）老年人生活环境。

　　1.加快老年活动场所和便利化设施建设。在城乡规划建设中，充分考虑老年人需求，加强街道、社区“老年人生活圈”配套设施建设，着力改善老年人的生活环境。通过新建和资源整合，缓解老年生活基础设施不足的矛盾。利用公园、绿地、广场等公共空间，开辟老年人运动健身场所。

　　2.完善涉老工程建设技术标准体系和实施监督制度。按照适应老龄化的要求，对现行老龄设施工程建设技术标准规范进行全面梳理、审定、修订和完善，在规划、设计、施工、监理、验收等各个环节加强技术标准的实施与监督，形成有效规范的约束机制。

　　3.加快推进无障碍设施建设。突出高龄和失能老年人居家养老服务设施、环境的无障碍改造，推行无障碍进社区、进家庭。加快对居住小区、园林绿地、道路、建筑物等与老年人日常生活密切相关的设施无障碍改造步伐，方便老年人出行和参与社会生活。研究制定《无障碍环境建设条例》，继续开展全国无障碍建设城市创建工作。

　　4.推动建设老年友好型城市和老年宜居社区。创新老年型社会新思维，树立老年友好环境建设和家庭发展的新理念。研究编制建设老年友好型城市、老年宜居社区指南，发挥典型示范作用。

　　（六）老龄产业。

　　1.完善老龄产业政策。把老龄产业纳入经济社会发展总体规划，列入国家扶持行业目录。研究制定、落实引导和扶持老龄产业发展的信贷、投资等支持政策。鼓励社会资本投入老龄产业。引导老年人合理消费，培育壮大老年用品消费市场。

　　2.促进老年用品、用具和服务产品开发。重视康复辅具、电子呼救等老年特需产品的研究开发。拓展适合老年人多样化需求的特色护理、家庭服务、健身休养、文化娱乐、金融理财等服务项目。培育一批生产老年用品、用具和提供老年服务的龙头企业，打造一批老龄产业知名品牌。

　　3.加强老年旅游服务工作。积极开发符合老年需求、适合老年人年龄特点的旅游产品。完善旅游景区、宾馆饭店、旅游道路的老年服务设施建设。完善针对老年人旅游的导游讲解、线路安排等特色服务。规范老年人旅游服务市场秩序。

　　4.引导老龄产业健康发展。研究制定老年产品用品质量标准，加强老龄产业市场监督管理。发挥老龄产业行业协会和中介组织的积极作用，加强信息服务和行业自律。疏通老龄产业发展融资渠道。

　　（七）老年人精神文化生活。

　　1.加强老年教育工作。创新老年教育体制机制，探索老年教育新模式，丰富教学内容。加大对老年大学（学校）建设的财政投入，积极支持社会力量参与发展老年教育，扩大各级各类老年大学办学规模。充分发挥党支部、基层自治组织和老年群众组织的作用，做好新形势下老年思想教育工作。

　　2.加强老年文化工作。加强农村文化设施建设，完善城市社区文化设施。鼓励创作老年题材的文艺作品，增加老年公共文化产品供给。鼓励和支持各级广播电台、电视台积极开设专栏，加大老年文化传播和老龄工作宣传力度。支持老年群众组织开展各种文化娱乐活动，丰富老年人的精神文化生活。

　　3.加强老年体育健身工作。在城乡建设、旧城改造和社区建设中，要安排老年体育健身活动场所。加强老年体育组织建设，积极组织老年人参加全民健身活动。经常参加体育健身的老年人达到50%以上。举办第二届全国老年人体育健身大会。

　　4.扩大老年人社会参与。注重开发老年人力资源，支持老年人以适当方式参与经济发展和社会公益活动。贯彻落实《中共中央办公厅　国务院办公厅转发〈中央组织部、中央宣传部、中央统战部、人事部、科技部、劳动保障部、解放军总政治部、中国科协关于进一步发挥离退休专业技术人员作用的意见〉的通知》（中办发〔2005〕9号），健全政策措施，搭建服务平台，支持广大离退休专业技术人员更好地发挥作用。重视发挥老年人在社区服务、关心教育下一代、调解邻里纠纷和家庭矛盾、维护社会治安等方面的积极作用。不断探索“老有所为”的新形式，积极做好“银龄行动”组织工作，广泛开展老年志愿服务活动，老年志愿者数量达到老年人口的10%以上。

　　（八）老年社会管理。

　　1.加强基层老龄工作机构和老年群众组织建设。各地要建立老龄工作委员会，城乡社区（村、居）要健全老龄工作机制。加强基层老年协会规范化建设，充分发挥老年人自我管理、自我教育、自我服务的积极作用。“十二五”期间，成立老年协会的城镇社区达到95%以上，农村社区（行政村）达到80%以上。

　　2.做好离退休人员管理服务工作。充分利用社区资源面向全体老年人开展服务，切实把为离退休老年人服务工作纳入社区服务范围。推进街道（乡镇）、社区劳动保障工作平台建设，为退休人员提供方便、快捷、高效、优质的服务。“十二五”期末，纳入社区管理服务的企业退休人员比例达到80%以上。

　　（九）老年人权益保障。

　　1.加强老龄法制建设。推进老年人权益保障法制化进程，做好修订《中华人民共和国老年人权益保障法》的相关工作，开展执法检查和普法教育，提高老年人权益保障法制化水平。

　　2.健全老年维权机制。弘扬孝亲敬老美德，促进家庭和睦、代际和顺。加强弱势老年人社会保护工作，把高龄、孤独、空巢、失能和行为能力不健全的老年人列为社会维权服务重点对象。加强对养老机构服务质量的检查、监督，维护老年人的生活质量与生命尊严，杜绝歧视、虐待老年人现象。

　　3.做好老年人法律服务工作。拓展老年人维权法律援助渠道，扩大法律援助覆盖面。重点在涉及老年人医疗、保险、救助、赡养、住房、婚姻等方面，为老年人提供及时、便利、高效、优质的法律服务。加大对侵害老年人权益案件的处理力度，切实保障老年人的合法权益。

　　4.加强青少年尊老敬老的传统美德教育。在义务教育中，增加孝亲敬老教育内容，开展形式多样的尊老敬老社会实践活动，营造良好的校园文化环境。

　　（十）老龄科研。

　　1.抓好重点科研项目。开展应对人口老龄化战略研究，制定国家老龄事业中长期发展规划。做好老年人生活状况追踪调查，开展区域性应对人口老龄化战略研究工作，为制定老龄政策提供决策依据。

　　2.加强老龄学科教育和专业人才培养。按照老龄事业发展规划和重点发展领域，统筹部署职业教育、高等教育学科专业设置，培养技能型、应用型、复合型人才，做好人力资源支撑，服务老龄事业发展。

　　3.推进信息化建设。建立老龄事业信息化协同推进机制，建立老龄信息采集、分析数据平台，健全城乡老年人生活状况跟踪监测系统。

　　（十一）老龄国际交流与合作。

　　广泛开展双边、多边国际交流，增进相互了解。积极发挥我国在国际老龄领域的重要影响，深化国际合作。密切跟踪联合国大会老龄问题工作组对建构老年人权利国际保护机制的动向，积极发挥作用，引导相关进程朝有利方向发展。积极研究借鉴国外应对人口老龄化理念和经验，做好联合国人口基金第七周期老龄项目。完成《国际老龄行动计划》在中国执行情况的检查评估。

　　四、保障措施

　　（一）加强组织领导。

　　各级政府要高度重视老龄问题，加强老龄工作。把发展老龄事业纳入重要议事日程，列入经济社会发展总体规划，及时解决老龄工作中的矛盾和问题。健全党政主导、老龄委协调、部门尽责、社会参与、全民关怀的大老龄工作格局。

　　（二）加大改革创新力度。

　　进一步解放思想，坚持改革，在体制机制、政策制度、工作思路和发展模式等方面加大创新力度，围绕涉老社会保障制度的配套衔接、老龄事业投入机制、政府购买服务方式、老龄服务市场准入与日常监管、民办养老机构扶持政策、社区养老服务资源的综合开发利用、老龄社会组织规范化建设等比较突出的矛盾和问题，深入开展调查研究，逐步完善政策法规制度，创新体制机制。

　　（三）建立多元长效投入机制。

　　各级政府要根据经济发展状况和老龄工作实际，多渠道筹资，不断加大老龄事业投入。进一步完善实施促进老龄事业发展的税收政策，政策引导与体制创新并重，调动社会资本投入老龄事业的积极性。大力发展老龄慈善事业。

　　（四）加强人才队伍建设。

　　加强老龄工作队伍的思想建设、组织建设、作风建设和业务能力建设。加快养老服务业人才培养，特别是养老护理员、老龄产业管理人员的培养。根据国家职业标准，组织开展养老护理人员职业培训和职业资格认证工作。有条件的普通高校和职业学校，在相关专业开设老年学、老年护理学、老年心理学等课程。大力发展为老服务志愿者队伍和社会工作者队伍。

　　（五）建立监督检查评估机制。

　　本规划由全国老龄工作委员会负责协调、督促、检查有关部门执行，2015年对规划的执行情况进行全面评估。

国务院关于印发国家基本公共服务体系
“十二五”规划的通知

国发〔2012〕29 号

各省、自治区、直辖市人民政府，国务院各部委、各直属机构：

　　现将《国家基本公共服务体系“十二五”规划》印发给你们，请认真贯彻执行。

　　　　　　　　　　　　　　　　　　　　　　　　　国务院

　　　　　　　　　　　　　　　　　　　　　　　　　2012年7月11日

第六章　基本社会服务

　　国家建立基本社会服务制度，为城乡居民尤其是困难群体的基本生活提供物质帮助，保障老年人、残疾人、孤儿等特殊群体有尊严地生活和平等参与社会发展。

| “十二五”时期，政府提供如下基本社会服务：  　　◆为城乡困难群体提供最低生活保障和专项救助；  　　◆为农村五保对象提供吃、穿、住、医、葬方面的生活照顾和物质帮助；  　　◆为自然灾害受灾人员提供救助；  　　◆为城市生活无着的流浪乞讨人员提供救助；  　　◆为残疾人、孤儿、精神病人等特殊群体提供福利服务；  　　◆为老年人提供基本养老服务；  　　◆为优抚安置对象提供优待抚恤和安置服务；  　　◆为城乡居民免费提供婚姻登记服务；  　　◆为身故者提供基本殡葬服务。 |
| --- |

第一节　重点任务

　　着力健全以城乡最低生活保障制度为核心，以农村五保供养、自然灾害救助、医疗救助、流浪乞讨人员救助制度为主要内容，以临时救助制度为补充的社会救助体系。以扶老、助残、救孤、济困为重点，逐步拓展社会福利的保障范围，推动社会福利由补缺型向适度普惠型转变，逐步提高国民福利水平。加强优抚安置工作。

　　——社会救助。完善城乡最低生活保障制度，健全低保标准动态调整机制。采取多种措施提高老年人、残疾人、未成年人和重病患者的保障水平。建立低收入家庭认定体系，健全收入核查制度。加强城乡低保与最低工资、失业保险和扶贫开发等政策的衔接。将专项救助逐步延伸至低保边缘家庭，重点解决其医疗、教育、住房等方面的困难。加强医疗救助与基本医疗保险制度的衔接，逐步实行诊疗费用即时救助，降低医疗救助起付线，有条件的地方可以取消医疗救助起付线。健全自然灾害监测预警、评估调查、信息发布、应急救援和应急物资储备体系，完善救助技术标准和补助项目。完善临时救助制度。加强城市生活无着的流浪乞讨人员救助管理，加大流浪未成年人保护力度。

　　——社会福利。建立健全孤儿保障体系，合理确定孤儿养育标准，建立自然增长机制。拓展孤儿安置渠道，鼓励家庭养育。扩大福利机构收养能力。加强贫困和重度精神疾病患者收养和治疗服务。推动婚姻登记标准化和全国信息联网，推行婚姻免费登记。有条件的地方可向城乡基本生活困难家庭发放基本殡葬服务补贴，提供遗体运送、火化和绿色安葬等服务。加快实施免费地名公共服务。依托社区综合服务平台，为社区居民提供公益便民利民社区服务。

　　——基本养老服务。适应人口老龄化趋势，有条件的地方可发放高龄老年人生活补贴和家庭经济困难的老年人养老服务补贴。将符合条件的农村老人全部纳入农村五保供养范围，实行分散供养与集中供养相结合，适度提高供养标准。建立健全养老服务体系，鼓励居家养老，拓展社区养老服务功能，增强公益性养老服务机构服务能力，鼓励通过公建民营、民办公助等方式引导社会资本参与养老服务机构建设和管理运行。

　　——优抚安置。全面落实优抚对象各项优待政策，确保军人的抚恤优待与经济和社会发展相适应。实施残疾军人辅具改造。改善优抚设施条件，健全孤老优抚对象和重残退役军人集中供养制度。落实退役士兵安置改革各项政策，组织引导符合条件的退役士兵免费参加职业教育和技能培训。

第二节　基本标准

　　加快建立健全基本社会服务国家标准体系。依据国家基本社会服务相关法律法规，为保障基本社会服务的规模和质量，明确工作任务的事权与支出责任，制定“十二五”时期基本社会服务国家基本标准。

　　各类基本社会服务机构资质认定、设施建设、设备配置、人员配备、服务规范以及服务对象资格认定等具体标准，由民政部依法会同有关部门及国家标准化行政管理部门制定实施。

各省（区、市）应遵循实施国家基本标准，并可结合本地区实际情况适当提高标准。

第十章　公共文化体育

　　国家建立公共文化体育服务制度，保障人民群众看电视、听广播、读书看报、进行公共文化鉴赏、参加大众文化活动和体育健身等权益。

| “十二五”时期，政府提供如下公共文化体育服务：  　　◆向全民免费开放基层公共文化体育设施，逐步扩大公共图书馆、文化馆（站）、博物馆、美术馆、纪念馆、科技馆、工人文化宫、青少年宫等免费开放范围；  　　◆为全民免费提供基本的广播电视服务和突发事件应急广播服务；  　　◆为农村居民免费提供文化信息资源共享、电影放映、送书送报送戏等公益性文化服务；  　　◆加强文化遗产保护和综合利用；  　　◆为城乡居民参加全民健身活动提供免费指导服务。 |
| --- |

第一节　重点任务

　　围绕建设社会主义核心价值体系和满足城乡居民精神文化需求的要求，坚持公益性、基本性、均等性、便利性，建立健全公共文化服务体系，扩大公共文化产品和服务的供给。推进全民健身公共服务体系建设。

　　——公益性文化。继续实施文化惠民工程，以农村基层和中西部地区为重点，加快公共文化基础设施建设。推进建立公共电子阅览室和未成年人公益性上网场所。促进城乡基层公共文化服务资源的共建共享。逐步实现公共文化场馆向全社会免费开放。推动文化科技卫生“三下乡”、“送欢乐下基层”等活动制度化，充分发挥流动文化服务车、流动电影放映车作用。广泛开展社区文化、村镇文化、校园文化、家庭文化等群众性文化活动，积极开展面向农民工和残疾人等群体的公益性文化服务。完善公益性演出补贴制度。加大对地方特色和民族特色文化的支持力度。加大文化和自然遗产、非物质文化遗产保护力度，逐步提高面向公众开放、展示的水平。

　　——广播影视。加强农村基层广播电视和无线发射台站建设，全面解决20户以下已通电自然村“盲村”广播电视覆盖。加强直播卫星平台建设，在有线网络未通达、无线网络不能覆盖的农村地区开展直播卫星公共服务。提高少数民族语言广播影视节目译制、制作、播出及传播覆盖能力。继续推进农村电影数字放映，将观看爱国主义教育影片纳入中小学教育教学计划。鼓励电影企业深入城乡社区、厂矿等开展公益放映活动。积极推进国家应急广播体系建设。加强地面数字电视建设，逐步完成地面模拟信号向数字信号的转换，不断提高无线广播电视公共服务的质量和水平。

　　——新闻出版。广泛开展全民阅读活动，逐步扩大基本免费或低收费阅读服务范围。继续加强农家书屋和城乡阅报栏（屏）建设，合理规划布局建设农村和中小城市出版发行网点。推进公益性数字出版产品免费下载、阅读和使用。大力扶持少数民族出版物的翻译和出版，积极开展少数民族文字书报刊赠送活动。

　　——群众体育。加强基层公共体育设施建设。大力推动公共体育设施向社会开放，健全学校等企事业单位体育设施向公众开放的管理制度。全面实施全民健身计划，健全基层全民健身组织服务体系，扶持社区体育俱乐部、青少年体育俱乐部和体育健身站（点）等建设，发展壮大社会体育指导员队伍，大力开展全民健身志愿服务活动。积极推广广播体操、工间操以及其他科学有效的全民健身方法，广泛开展形式多样、面向大众的群众性体育活动。建立国家、省、市三级体质测定与运动健身指导站，普及科学健身知识，指导群众科学健身。推动落实国家体育锻炼标准，加强学生体质监测，制定残疾人体质测定标准，定期开展国民体质监测。

第二节　基本标准

　　加快建立健全公共文化体育服务国家标准体系。依据国家文化体育相关法律法规，为保障服务的供给规模和质量，明确工作任务的事权与支出责任，促进城乡均衡发展，制定“十二五”时期公共文化体育服务国家基本标准。

　　各类公共文化体育设施布局、场馆建设、设备配置、人员配备、服务规范等具体标准，由文化部、广电总局、新闻出版总署、文物局和体育总局依法会同有关部门及国家标准化行政管理部门制定实施。

　　各省（区、市）应遵循实施国家基本标准，并可结合本地区实际情况适当提高标准。

“十二五”时期公共文化体育服务国家基本标准

| 服务项目 | 服务对象 | 保障标准 | 支出责任 | 覆盖水平 |  |
| --- | --- | --- | --- | --- | --- |
| 公益性文化服务 | | | | | |
| 公共文化场馆 开放 | 城乡居民 | 公共空间设施和基本服务项目免费，全年开放时间不少于10个月 | 中央和地方财政按比例共同负担 | 除文物建筑及遗址类博物馆外，各级文化文物部门归口管理的公共文化场馆全面向社会开放 |  |
| 公益性流动文化服务 | 城乡居民 | 免费享有影视放映、文艺演出、图片展览、图书销售和借阅、科技宣传为一体的流动文化服务；每个乡镇每年送4场地方戏曲；每学期中小学生观看两部爱国主义教育影片 | 地方政府负责，中央财政适当补助 | 基本建立灵活机动、方便群众的公益性流动文化服务网络，保障公益性演出场次 |  |
| 广播影视 | | | | | |
| 农村广播电视 | 农村居民为主 | 无偿提供中央第一套广播节目、中央第一套和第七套电视节目及本省第一套广播电视节目等4套以上广播和电视节目服务，逐步增加节目套数和提高播放质量 | 中央和地方政府共同负责 | 基本实现所有通电行政村和自然村村村和户户通广播电视 |  |
| 农村电影放映 | 农村居民 | 行政村一村一月放映一场电影，每场财政补贴200元 | 中央和地方财政按比例共同负担 | 每年放映780万场公益电影 |  |
| 少数民族语言 广播影视 | 主要少数民族地区居民 | 通过有线、无线或卫星等方式能够收听收看到本民族语言广播影视节目 | 中央和地方政府共同负责 | 覆盖藏、维、蒙、哈、朝、壮、傣等主要少数民族地区 |  |
| 应急广播 | 城乡居民 | 在突发公共事件发生前后及时获得政令、信息等服务 | 中央和地方政府共同负责 | 在全国范围内基本实现分层次、分类型、全方位立体覆盖 |  |
| 新闻出版 | | | | | |
| 公共阅读服务 | 城乡居民 | 农村行政村建立农家书屋，图书不少于1500册，报刊20—30种，电子音像制品不少于100种（张），并及时更新；城市和乡镇主要街道、大专院校、居民小区等人流密集地点设公共阅报栏（屏），及时提供各类新闻和服务信息 | 中央和地方财政按比例共同负担 | 基本实现行政村村村有农家书屋，新增城乡公共阅报栏（屏）10万个，国民综合阅读率达到80% |  |
| 民文出版译制 | 有文字的少数民族 | 可以获得本民族语言文字出版的、价格适宜的常用书刊、电子音像制品，政府给予出版物资助 | 中央和地方政府共同负责 | 每年选择不少于800种优秀国内外书刊、电子音像制品翻译成少数民族语言文字 |  |
| 盲文出版 | 盲人 | 可以获得价格适宜的盲文出版物，政府给予出版物资助 | 中央和地方政府共同负责 | 年生产盲文书刊1600种、70万册 |  |
| 文化遗产展示 | | | | | |
| 文化遗产展示 门票减免 | 未成年人、老年人、现役军人、残疾人和低收入人群 | 减免参观文物建筑及遗址类博物馆的门票 | 中央和地方财政分别负担 | 目标人群覆盖率100% |  |
| 群众体育 | | | | | |
| 体育场馆开放 | 城乡居民 | 有条件的公办体育设施（含学校体育设施）向公众开放，免费项目或有关收费标准由地方政府制定；开放时间与当地公众的工作时间、学习时间适当错开，不少于省（区、市）规定的最低时限，全民健身日免费开放，国家法定节假日和学校寒暑假期间，应当适当延长开放时间 | 地方政府负责，中央财政适当补助 | 可供使用的公共体育场地（含学校体育场地）占全国体育场地总数的比率达到53%左右 |  |
| 全民健身服务 | 城乡居民 | 免费享有健身技能指导、参加健身活动、获取科学健身知识等服务；免费提供公园、绿地等公共场所全民健身器材 | 地方政府负责，中央财政适当补助 | 经常参加体育锻炼人数比率达到32%以上 |  |

第三节　保障工程

　　实施公共文化体育服务保障工程，健全服务网络，着力改善基层文化体育设施条件，有效提升公共文化体育服务能力。

　　——公共文化服务体系建设工程。继续推进广播电视村村通、文化信息资源共享、国家数字图书馆推广工程、公共电子阅览室建设计划、农村数字电影放映、农家书屋、西藏新疆等边疆民族地区广播电视覆盖工程和边疆地区少数民族新闻出版工作，实施地面数字电视覆盖和直播卫星广播电视公共服务建设，新建、改扩建一批市（地）级公共图书馆、文化馆、博物馆。

　　——传播体系建设工程。重点加强媒体传播能力、民族文字出版和民族语言广播、文化传播渠道、国家应急广播体系建设。

　　——文化和自然遗产保护工程。重点支持国家重大文化和自然遗产地、全国重点文物保护单位、大遗址、中国历史文化名城名镇名村保护设施建设，推进非物质文化遗产保护利用设施建设试点。做好历史档案和文化典籍保护整理工作。

　　——体育基本公共服务建设工程。重点支持县级公共体育场建设，加快建设一批面向群众、贴近基层的中小型全民健身中心和灯光球场，充分利用城市绿地、广场、公园等公共场所和适宜的自然区域建设全民健身活动设施。继续实施农民体育健身工程，改善农村公共体育设施条件。

**文化部关于印发《文化部“十二五”时期
文化改革发展规划》的通知**

**文化部“十二五”时期文化改革发展规划**

　　“十二五”时期是全面建设小康社会的关键时期，是深化改革开放、加快转变经济发展方式的攻坚时期，也是促进文化又好又快发展的关键阶段。为深入贯彻落实党的十七届六中全会精神，深化文化体制改革，推动社会主义文化大发展大繁荣，不断增强国家文化软实力，根据《中共中央关于深化文化体制改革、推动社会主义文化大发展大繁荣若干重大问题的决定》、《中华人民共和国国民经济和社会发展第十二个五年规划纲要》和《国家“十二五”时期文化改革发展规划纲要》，编制本规划。

序　　言

　　文化是民族的血脉，是人民的精神家园。当今世界，文化地位和作用更加凸显，文化越来越成为民族凝聚力和创造力的重要源泉、越来越成为综合国力竞争的重要因素、越来越成为经济社会发展的重要支撑，丰富精神文化生活越来越成为我国人民的热切愿望。文化的发展关系实现全面建设小康社会奋斗目标，关系坚持和发展中国特色社会主义，关系实现中华民族伟大复兴。
　　“十一五”时期是我国文化建设的创新发展期，文化建设取得令人瞩目的新成就。文艺创作生产进一步繁荣，推出一大批优秀作品。覆盖城乡的公共文化服务体系初步建立，人民群众的基本文化权益得到有效保障。一大批文化设施相继建成投入使用，有效改善了文化发展的基本物质条件。文化产业蓬勃发展，日益成为经济发展新的增长点。文化遗产保护制度初步建立，保护状况得到明显改善。文化体制改革取得实质性进展，推动文化科学发展的体制机制初步形成。文化与科技的融合日益加深，文化创新能力不断增强。文化市场体系更加完善，监管水平不断提高。对外及对港澳台文化交流与贸易不断拓展，中华文化的国际影响力明显提升。“人才兴文”战略顺利实施，文化人才队伍不断壮大。文化投入稳步增长，为文化发展提供了重要支撑。
　　当前和今后一段时期，我国发展仍处于可以大有作为的重要战略机遇期，文化领域正在发生广泛而深刻的变革，推动文化大发展大繁荣既具备许多有利条件，也面临一系列新情况新问题。我国经济持续快速发展、综合国力日益增强，为文化建设奠定了坚实的物质基础；中国特色社会主义理论和实践的丰硕成果，为文化建设提供了宝贵的精神文化资源；全社会重视、参与文化建设的热情日益高涨，为文化建设营造了良好的社会氛围；人民群众快速增长的精神文化需求，为文化发展拓展了巨大的空间；我国的国际地位和影响力显著提高，为中华文化走出去提供了重要契机。文化改革发展面临难得的历史机遇。同时，我们还必须看到，面对新形势新要求，我国文化发展的整体质量和水平还不够高，与人民群众日益增长的精神文化需求，与快速发展的现代传播手段，与不断扩大对外开放、推动我国经济社会又好又快发展的新形势相比，还不完全适应。文化建设自身还存在一些不足，城乡、区域文化发展不均衡的情况仍然存在，文化人才结构和布局不够合理，制约文化科学发展的体制机制障碍尚未完全破除。必须不断增强机遇意识和忧患意识，抓住机遇，迎接挑战，更加自觉、更加主动地推动社会主义文化大发展大繁荣，努力开创中国特色社会主义文化建设新局面。
　　**一、指导思想和方针原则**
　　（一）指导思想
　　高举中国特色社会主义伟大旗帜，以马克思列宁主义、毛泽东思想、邓小平理论和“三个代表”重要思想为指导，深入贯彻落实科学发展观，坚持社会主义先进文化前进方向，以科学发展为主题，以建设社会主义核心价值体系为根本任务，以满足人民精神文化需求为出发点和落脚点，以改革创新为动力，发展面向现代化、面向世界、面向未来的民族的科学的大众的社会主义文化，培养高度的文化自觉和文化自信，提高全民族文明素质，增强国家文化软实力，弘扬中华文化，坚持中国特色社会主义文化发展道路，努力建设社会主义文化强国。
　　（二）方针原则
　　1．坚持社会主义先进文化前进方向。贯彻“二为”方向、“双百”方针，弘扬主旋律，提倡多样化，推进社会主义核心价值体系建设，始终把社会效益放在首位，实现经济效益和社会效益的有机统一。
　　2．坚持以人为本。发挥人民群众的主体作用，不断激发全社会的文化创造活力。充分调动广大文化工作者的积极性和创造性，贴近实际、贴近生活、贴近群众。坚持文化发展为了人民、文化发展依靠人民、文化发展成果由人民共享。
　　3．坚持改革创新。树立新的文化发展理念，创新文化管理思路，提高文化的科学发展水平。加快推进体制机制、内容形式、传播手段和发展业态创新，不断解放和发展文化生产力，增强社会主义文化的吸引力和凝聚力。
　　4．坚持统筹兼顾。正确处理和把握文化改革发展中的重大关系。一手抓公益性文化事业，一手抓经营性文化产业，实现两轮驱动。统筹城乡、区域发展，形成城市带动农村和东中西优势互补、良性互动的发展格局。统筹国内国际两种资源，继承弘扬中华优秀文化，吸收借鉴世界文明有益成果，维护世界文化多样性。处理好改革与发展、繁荣与管理的关系，促进文化协调健康发展。
　　5．坚持重在建设。着眼长远，立足当前，切实推进思想建设、队伍建设、制度建设、业务建设，加强基础设施建设，实施重大文化工程，不断推动各项文化工作取得新突破。
　　**二、发展目标和主要指标**
　　（一）发展目标
　　围绕建设社会主义文化强国的宏伟目标，全面落实到2020年文化改革发展的总体部署，到2015年，文化建设的主要目标是：文化产品创作生产体系不断完善，创作生产更多无愧于历史、无愧于时代、无愧于人民的优秀作品，为人民提供更好更多的精神食粮。覆盖城乡、结构合理、功能健全、实用高效的公共文化服务体系基本建立，各级各类文化设施更加完善，使人民群众能够公平、就近、便捷享受公共文化服务，基本文化权益得到更好保障。推动文化产业实现跨越式发展，逐步成为促进经济发展方式转变、优化经济结构、扩大就业创业的国民经济支柱性产业。科技进步成为文化发展的重要动力和引擎，文化与科技融合在深度与广度上得到实质性推进。统一开放竞争有序的现代文化市场体系基本构建，文化市场监管进一步加强，文化市场经营秩序更加规范。文化遗产保护理念深入人心，保护体系基本形成，实现全面保护与有效传承。对外文化交流和贸易迈上新台阶，基本形成官民并举的对外和对港澳台文化工作新格局，中华文化影响力不断扩大。文化体制改革重点任务基本完成，文化体制机制充满活力、富有效率，有力促进文化科学发展。文化人才队伍发展壮大，人才结构更加合理。文化引领风尚、教育人民、服务社会、推动发展的功能充分发挥，国家文化软实力和国际竞争力显著提升。
　　（二）主要指标
　　——保证公共财政对文化建设投入的增长幅度高于财政经常性收入增长幅度，提高文化支出占财政支出比例。
　　——“十二五”期间，推出100部以上深受人民群众喜爱、久演不衰的优秀保留剧目和精品剧目，保护和扶持60个左右全国重点地方戏曲院团，扶持创作60台左右优秀地方戏剧目，30台左右优秀京剧剧目，挖掘整理改编20台左右优秀昆曲剧目，重点扶持20台左右交响乐、15台左右歌剧（音乐剧）、10台左右舞剧（芭蕾舞剧），扶持10个左右全国重点美术馆。
　　——到“十二五”期末，全国60%以上图书馆达到部颁三级以上评估标准，全国60%以上省市群艺馆、文化馆达到部颁三级以上评估标准。基本实现全国所有地市级城市均有设施达标、布局合理、功能完善的公共图书馆、文化馆。
　　——到“十二五”期末，全国人均拥有公共图书馆藏书达到0.7册左右。各级公共图书馆，文化共享工程乡镇、街道、社区基层服务点基本建有公共电子阅览室。文化信息资源共享工程资源量争取达到530百万兆字节以上，入户率达到50%左右。国家数字图书馆资源总量争取达到1000百万兆字节以上，并提供全媒体服务。中西部地区争取每县配备1台流动文化车，中西部地区已完成转制的县级剧团每团配备1辆流动舞台车。
　　——“十二五”期间，文化部门管理的文化产业增加值年平均现价增长速度高于20%，2015年比2010年至少翻一番，实现倍增。建成10家左右具有重大影响的国家级文化产业示范园区，培育100个左右特色鲜明、主导产业突出的特色文化产业集群，培育30家左右上市文化企业，形成10家左右全国性或跨区域的文艺演出院线，打造3-5个具有国际影响的文化产业展会。
　　——到“十二五”期末，第一至六批全国重点文物保护单位的重大文物险情排除率达到100%，全国博物馆总数达到3500个，免费开放博物馆总数达到2500个，文化遗存较丰富的地市级以上中心城市拥有1个功能健全的博物馆。国有博物馆一级文物的建账建档率达到100%。文物博物馆一级风险单位中文物收藏单位的防火、防盗设施达标率达到100%。“十二五”期间，新设立20个国家级文化生态保护区，在非物质文化遗产资源丰富的地区建设100个非物质文化遗产保护利用设施。
　　——“十二五”期间，安排150个左右重点科技攻关项目、300个左右基础科研项目、75个科技转化推广项目。国家社科基金艺术学项目立项600个、文化部文化艺术科学研究项目立项300个。
　　——“十二五”期间，在国际、多边、双边等场合举办国家级重大涉外文化活动30项以上，邀请500名国际文化名人与1000名青少年文化使者来华访问，对外文化援助的受援国家达20个以上。海外中国文化中心形成合理布局，到“十二五”期末，总数达到25-30所。
　　**三、加强文化产品创作生产的引导**
　　（一）实施精品战略。引导广大文化工作者自觉践行社会主义核心价值体系，坚持社会主义先进文化前进方向，反映人民主体地位和现实生活，积极发挥重大文化工程的示范作用，把精品意识贯穿于文艺创作生产全过程，不断增强艺术院团、美术馆、画院的创作演出展览能力，提高文艺作品质量。继续实施“国家舞台艺术精品工程”、“国家重点京剧院团保护与扶持规划”、“国家昆曲艺术抢救、保护和扶持工程”、“国家美术收藏和捐赠奖励工程”、“国家重大现实题材美术创作工程”等重点项目，推出一批深受群众喜爱、思想性艺术性观赏性相统一的精品力作。
　　（二）促进各艺术门类全面协调发展。切实加强对艺术事业的宏观管理，建立国家扶持艺术创作、生产、传播的长效机制。加大投入力度，设立国家艺术基金，面向全社会文化机构和个人进行资助和奖励，支持优秀艺术作品创作和优秀文艺人才培养，引导文化产品创作生产。大力推动精品创作展演，推动戏剧（戏曲）、音乐、舞蹈、杂技、曲艺、美术等艺术形式繁荣发展，特别重视民族民间艺术形式的繁荣发展。开拓艺术传播渠道。办好中国艺术节、中国京剧节等重大艺术活动。通过组织巡演、下基层慰问演出、高雅艺术进校园、低票价运营、演出交易会等办法，进一步加强宣传，扩大优秀艺术作品在国内外的知名度及影响力。
　　（三）切实加强和改进文艺评奖和文艺评论。坚持把遵循社会主义先进文化前进方向、人民群众满意作为评价作品最高标准，把群众评价、专家评价和市场检验统一起来，形成科学的评价标准。建立公开、公平、公正评奖机制，精简评奖种类，改进评奖办法，不断提高国家级文艺奖项的权威性和公信度。加强和改进文艺批评，坚持正确创作方向，倡导主流价值取向，着力增强文艺评论的针对性。培育文艺评论骨干力量，推动文艺精品宣传推介的常态化、制度化。
　　（四）发挥艺术科研的导向和促进作用。充分发挥国家社科基金艺术学项目和文化部文化艺术科学研究项目的导向作用，进一步深化和拓展我国艺术学学科体系建设和当代文化发展问题研究。加强艺术研究院所建设，拓展职能，提升效能。发挥艺术科研工作在文化决策咨询、文艺档案管理、文化活动策划、文艺刊物建设等方面的作用。

| 专栏1.艺术创作重点工程 |
| --- |
| 国家艺术创作引导扶持工程：实施精品剧目扶持计划、地方戏剧种保护和扶持计划、部分艺术品种阶段性引导扶持计划、西部和少数民族地区艺术创作重点扶持计划、优秀艺术院团引导扶持计划、优秀艺术作品推广计划、国家美术发展计划、文艺理论与批评扶持计划。  　　国家艺术基金：由国家设立旨在繁荣艺术创作、推出精品力作、培养艺术创作人才的专项艺术基金，面向社会文化机构和个人进行资助和奖励。完善基金运作方式。通过项目补贴、优秀奖励、匹配资助等多种方式对艺术创作、宣传推广、征集收藏和人才培养四个方面进行资助。 |

**四、加快构建公共文化服务体系**
　　（一）创新公共文化服务机制。以国家公共文化服务体系示范区建设为抓手，进一步强化地方党委、政府主导责任，努力突破体制障碍，盘活文化资源，加大跨部门、跨领域、跨系统文化项目的交流与合作，实现基层公共文化资源综合利用，共建共享。加强公共文化服务体系软件建设，推进理论政策和制度设计研究，探索建立符合我国国情、符合文化发展特点和社会主义市场经济规律的公共文化服务体系制度基本框架。重点建立群众文化需求动态反馈、公共文化服务经费保障、社会力量参与公共文化绩效评价和监督等机制，加快制定公共文化服务机构的服务标准和服务规范，逐步推进公共文化服务的制度化、标准化和规范化。
　　（二）完善公共文化设施网络。适应推进城市化和建设社会主义新农村的要求，统筹规划，合理布局，以城乡基层文化设施建设为重点，以流动文化设施和数字文化阵地建设为补充，继续加强公共文化设施建设，努力形成比较完备的国家、省、市、县（区）、乡镇（街道）、村（社区）六级公共文化设施网络。进一步加大城乡基层文化设施建设力度，重点向贫困地区、落后地区、革命老区和基层农村倾斜。以服务人口为依据，制定和完善设施建设标准和设备配置标准，推进公共文化设施建设的规范化、标准化。实施全国地市级公共文化设施建设规划，建设一批地市级公共图书馆、文化馆、博物馆。继续实施县级图书馆、文化馆修缮和社区文化中心（活动室）服务能力建设等项目。建立灵活机动、方便群众的流动服务网络。
　　（三）加大公共文化产品和服务供给力度。充分发挥公共文化单位在公共文化产品创作和服务提供方面的重要作用，为群众提供优质高效、普遍均等的公共文化产品和服务。继续推动文化馆（站）、博物馆、图书馆、美术馆、纪念馆向社会免费开放。推广公共图书馆总分馆制。依托公共图书馆讲座联盟等平台，鼓励各级公共图书馆开展立法决策咨询、讲座、展览等服务。加强流动文化服务。推广政府购买、集中配送、连锁服务等公共文化产品提供方式，健全市场化提供机制。引导社会力量有序参与公共文化服务，支持各种民办博物馆、图书馆等公益性文化机构发展，努力形成良性竞争、多元互补的公共文化服务供给体系。
　　（四）大力推动数字文化建设。大力推进全国文化信息资源共享工程，充分发挥其在公共文化服务中的战略性、基础性作用，建立公共文化资源提供平台，推进数字服务进入家庭。建立内容丰富的数字文化资源库群，加强少数民族语言数字资源译制工作。实施公共电子阅览室建设计划，利用文化信息资源共享工程工作网络，依托公益性文化单位，建立公共电子阅览室，为基层群众特别是广大青少年提供内容健康、服务规范、环境良好的公益性互联网服务。加强数字图书馆建设，借助“三网融合”工程，实现全国图书馆资源的无障碍共享。实施数字图书馆推广工程，以技术手段整合国家数字图书馆与全国各级公共图书馆数字资源，形成覆盖全国的数字图书馆服务网络。搭建满足不同需求的全媒体数字图书馆服务平台。推进数字博物馆建设工程，建立博物馆信息资源共享平台。努力形成覆盖城乡的数字文化服务体系。
　　（五）广泛开展群众性文化活动。以“群星奖”、“中国民间文化艺术之乡”为龙头，推出一批优秀的、具有可持续发展价值的文化品牌，提高影响力，发挥导向性、示范性和带动性作用，实现群众文化活动的整体推进、全面提高。以群众文化需求为导向，鼓励广大文化工作者生产创作一批深刻反映时代精神，具有浓厚生活气息和较高艺术价值，为群众喜闻乐见的文化精品剧（节）目，并加强推广力度。挖掘各类节庆活动的文化内涵，丰富其内容和形式，坚持面向基层，服务群众，组织好“文化下乡”、“文化进社区”、群众文艺精品巡演展演、老年合唱节、少儿合唱节等公益性文化活动，广泛开展群众乐于参与、便于参与的城乡基层文化活动，丰富城乡基层群众的精神文化生活。
　　（六）推进基本公共文化服务均等化。加强面向特定地域、特殊群体的文化关怀。以农民、进城务工人员、老年人、未成年人、下岗失业人员、低收入人群、残障人群等群体为对象，通过政府补贴、发放文化消费券等措施，提高公共文化供给能力。将进城务工人员纳入城市公共文化服务范畴，合理配置公共文化资源。完善东部地区对西部地区、发达地区对欠发达地区、城市对农村的文化援助机制。实施文化建设“春雨工程”。继续加快推进边疆少数民族地区基层公共文化基础设施建设。鼓励艺术院校学生、文艺工作者和热心文化公益事业的各界人士开展文化志愿服务。扩大实施“全国文化志愿者边疆行”活动，丰富边疆少数民族地区文化生活。

| 专栏2.公共文化服务体系建设重点工程 |
| --- |
| 重大文化设施建设：推进国家美术馆、中国工艺美术馆、中国非物质文化遗产展示馆、中央歌剧院剧场、国家图书馆一期维修改造、国家文献战略储备库、中国国家画院扩建、中国交响乐团改扩建、中国歌剧舞剧院剧场、中国东方大剧院、中央文化管理干部学院改扩建、中国艺术研究院研究生院等重点文化设施建设。  　　全国地市级公共文化设施建设规划：完成532个地市级公共图书馆、文化馆、博物馆建设项目，其中，地市级公共图书馆189个，地市级文化馆221个，地市级博物馆122个。规划实施完成后，基本实现全国地市都建有设施达标、功能完善、布局合理的公共图书馆和文化馆，文物资源特别丰富的地市文物馆藏及展示条件得到明显改善。  　　全国文化信息资源共享工程：实现从城市到农村服务网络全面覆盖。大力推进服务网络建设，积极推进进村入户，建立“公共文化数字资源基础库群”和“红色历史文化多媒体资源库”，加强少数民族语言数字资源译制等。 　　 公共电子阅览室建设计划：利用全国文化信息资源共享工程工作网络，依托公益性文化单位，建立公共电子阅览室，为基层群众，特别是广大青少年提供绿色上网空间。 数字图书馆推广工程：建立海量分布式数字资源库群，构建以国家数字图书馆为核心，以省级数字图书馆为主要节点的全国性数字图书馆虚拟网，形成覆盖全国的数字图书馆服务网络，搭建全媒体服务平台，使数字图书馆建设成果实现全民共享。  　　文化馆（站）、公共图书馆、美术馆免费开放计划：深入推进文化馆（站）、公共图书馆、全国美术馆设施免费向群众开放，与其职能相适应的基本服务项目健全并免费向群众提供。  　　国家公共文化服务体系示范区（项目）创建工程：创建国家公共文化服务体系建设示范区90个左右，示范项目180个左右，涵盖全国1/3市县。  　　公共文化单位服务能力建设项目：用于图书馆、文化馆（站）等基层公共文化机构制度创新、丰富服务内容、强化管理、提高队伍素质等软件建设。  　　文化建设“春雨工程”：以新疆为试点，在边疆和少数民族地区加快推进以基层为重点的公共文化基础设施建设，着力构建公共文化服务体系运行经费保障机制，加强文化活动和文化内容建设，加大文化艺术人才培养和文化干部队伍建设。 |

**国务院关于加快发展养老服务业的若干意见**
国发〔2013〕35号

各省、自治区、直辖市人民政府，国务院各部委、各直属机构：
　　近年来，我国养老服务业快速发展，以居家为基础、社区为依托、机构为支撑的养老服务体系初步建立，老年消费市场初步形成，老龄事业发展取得显著成就。但总体上看，养老服务和产品供给不足、市场发育不健全、城乡区域发展不平衡等问题还十分突出。当前，我国已经进入人口老龄化快速发展阶段，2012年底我国60周岁以上老年人口已达1.94亿，2020年将达到2.43亿，2025年将突破3亿。积极应对人口老龄化，加快发展养老服务业，不断满足老年人持续增长的养老服务需求，是全面建成小康社会的一项紧迫任务，有利于保障老年人权益，共享改革发展成果，有利于拉动消费、扩大就业，有利于保障和改善民生，促进社会和谐，推进经济社会持续健康发展。为加快发展养老服务业，现提出以下意见：
　　**一、总体要求**
　　（一）指导思想。以邓小平理论、“三个代表”重要思想、科学发展观为指导，从国情出发，把不断满足老年人日益增长的养老服务需求作为出发点和落脚点，充分发挥政府作用，通过简政放权，创新体制机制，激发社会活力，充分发挥社会力量的主体作用，健全养老服务体系，满足多样化养老服务需求，努力使养老服务业成为积极应对人口老龄化、保障和改善民生的重要举措，成为扩大内需、增加就业、促进服务业发展、推动经济转型升级的重要力量。
　　（二）基本原则。
　　深化体制改革。加快转变政府职能，减少行政干预，加大政策支持和引导力度，激发各类服务主体活力，创新服务供给方式，加强监督管理，提高服务质量和效率。
　　坚持保障基本。以政府为主导，发挥社会力量作用，着力保障特殊困难老年人的养老服务需求，确保人人享有基本养老服务。加大对基层和农村养老服务的投入，充分发挥社区基层组织和服务机构在居家养老服务中的重要作用。支持家庭、个人承担应尽责任。
　　注重统筹发展。统筹发展居家养老、机构养老和其他多种形式的养老，实行普遍性服务和个性化服务相结合。统筹城市和农村养老资源，促进基本养老服务均衡发展。统筹利用各种资源，促进养老服务与医疗、家政、保险、教育、健身、旅游等相关领域的互动发展。
　　完善市场机制。充分发挥市场在资源配置中的基础性作用，逐步使社会力量成为发展养老服务业的主体，营造平等参与、公平竞争的市场环境，大力发展养老服务业，提供方便可及、价格合理的各类养老服务和产品，满足养老服务多样化、多层次需求。
　　（三）发展目标。到2020年，全面建成以居家为基础、社区为依托、机构为支撑的，功能完善、规模适度、覆盖城乡的养老服务体系。养老服务产品更加丰富，市场机制不断完善，养老服务业持续健康发展。
　　——服务体系更加健全。生活照料、医疗护理、精神慰藉、紧急救援等养老服务覆盖所有居家老年人。符合标准的日间照料中心、老年人活动中心等服务设施覆盖所有城市社区，90%以上的乡镇和60%以上的农村社区建立包括养老服务在内的社区综合服务设施和站点。全国社会养老床位数达到每千名老年人35-40张，服务能力大幅增强。
　　——产业规模显著扩大。以老年生活照料、老年产品用品、老年健康服务、老年体育健身、老年文化娱乐、老年金融服务、老年旅游等为主的养老服务业全面发展，养老服务业增加值在服务业中的比重显著提升，全国机构养老、居家社区生活照料和护理等服务提供1000万个以上就业岗位。涌现一批带动力强的龙头企业和大批富有创新活力的中小企业，形成一批养老服务产业集群，培育一批知名品牌。
　　——发展环境更加优化。养老服务业政策法规体系建立健全，行业标准科学规范，监管机制更加完善，服务质量明显提高。全社会积极应对人口老龄化意识显著增强，支持和参与养老服务的氛围更加浓厚，养老志愿服务广泛开展，敬老、养老、助老的优良传统得到进一步弘扬。
　　**二、主要任务**
　　（一）统筹规划发展城市养老服务设施。
　　加强社区服务设施建设。各地在制定城市总体规划、控制性详细规划时，必须按照人均用地不少于0.1平方米的标准，分区分级规划设置养老服务设施。凡新建城区和新建居住（小）区，要按标准要求配套建设养老服务设施，并与住宅同步规划、同步建设、同步验收、同步交付使用；凡老城区和已建成居住（小）区无养老服务设施或现有设施没有达到规划和建设指标要求的，要限期通过购置、置换、租赁等方式开辟养老服务设施，不得挪作他用。
　　综合发挥多种设施作用。各地要发挥社区公共服务设施的养老服务功能，加强社区养老服务设施与社区服务中心（服务站）及社区卫生、文化、体育等设施的功能衔接，提高使用率，发挥综合效益。要支持和引导各类社会主体参与社区综合服务设施建设、运营和管理，提供养老服务。各类具有为老年人服务功能的设施都要向老年人开放。
　　实施社区无障碍环境改造。各地区要按照无障碍设施工程建设相关标准和规范，推动和扶持老年人家庭无障碍设施的改造，加快推进坡道、电梯等与老年人日常生活密切相关的公共设施改造。
　　（二）大力发展居家养老服务网络。
　　发展居家养老便捷服务。地方政府要支持建立以企业和机构为主体、社区为纽带、满足老年人各种服务需求的居家养老服务网络。要通过制定扶持政策措施，积极培育居家养老服务企业和机构，上门为居家老年人提供助餐、助浴、助洁、助急、助医等定制服务；大力发展家政服务，为居家老年人提供规范化、个性化服务。要支持社区建立健全居家养老服务网点，引入社会组织和家政、物业等企业，兴办或运营老年供餐、社区日间照料、老年活动中心等形式多样的养老服务项目。
　　发展老年人文体娱乐服务。地方政府要支持社区利用社区公共服务设施和社会场所组织开展适合老年人的群众性文化体育娱乐活动，并发挥群众组织和个人积极性。鼓励专业养老机构利用自身资源优势，培训和指导社区养老服务组织和人员。
　　发展居家网络信息服务。地方政府要支持企业和机构运用互联网、物联网等技术手段创新居家养老服务模式，发展老年电子商务，建设居家服务网络平台，提供紧急呼叫、家政预约、健康咨询、物品代购、服务缴费等适合老年人的服务项目。
　　（三）大力加强养老机构建设。
　　支持社会力量举办养老机构。各地要根据城乡规划布局要求，统筹考虑建设各类养老机构。在资本金、场地、人员等方面，进一步降低社会力量举办养老机构的门槛，简化手续、规范程序、公开信息，行政许可和登记机关要核定其经营和活动范围，为社会力量举办养老机构提供便捷服务。鼓励境外资本投资养老服务业。鼓励个人举办家庭化、小型化的养老机构，社会力量举办规模化、连锁化的养老机构。鼓励民间资本对企业厂房、商业设施及其他可利用的社会资源进行整合和改造，用于养老服务。
　　办好公办保障性养老机构。各地公办养老机构要充分发挥托底作用，重点为“三无”（无劳动能力，无生活来源，无赡养人和扶养人、或者其赡养人和扶养人确无赡养和扶养能力）老人、低收入老人、经济困难的失能半失能老人提供无偿或低收费的供养、护理服务。政府举办的养老机构要实用适用，避免铺张豪华。
　　开展公办养老机构改制试点。有条件的地方可以积极稳妥地把专门面向社会提供经营性服务的公办养老机构转制成为企业，完善法人治理结构。政府投资兴办的养老床位应逐步通过公建民营等方式管理运营，积极鼓励民间资本通过委托管理等方式，运营公有产权的养老服务设施。要开展服务项目和设施安全标准化建设，不断提高服务水平。
　　（四）切实加强农村养老服务。
　　健全服务网络。要完善农村养老服务托底的措施，将所有农村“三无”老人全部纳入五保供养范围，适时提高五保供养标准，健全农村五保供养机构功能，使农村五保老人老有所养。在满足农村五保对象集中供养需求的前提下，支持乡镇五保供养机构改善设施条件并向社会开放，提高运营效益，增强护理功能，使之成为区域性养老服务中心。依托行政村、较大自然村，充分利用农家大院等，建设日间照料中心、托老所、老年活动站等互助性养老服务设施。农村党建活动室、卫生室、农家书屋、学校等要支持农村养老服务工作，组织与老年人相关的活动。充分发挥村民自治功能和老年协会作用，督促家庭成员承担赡养责任，组织开展邻里互助、志愿服务，解决周围老年人实际生活困难。
　　拓宽资金渠道。各地要进一步落实《中华人民共和国老年人权益保障法》有关农村可以将未承包的集体所有的部分土地、山林、水面、滩涂等作为养老基地，收益供老年人养老的要求。鼓励城市资金、资产和资源投向农村养老服务。各级政府用于养老服务的财政性资金应重点向农村倾斜。
　　建立协作机制。城市公办养老机构要与农村五保供养机构等建立长期稳定的对口支援和合作机制，采取人员培训、技术指导、设备支援等方式，帮助其提高服务能力。建立跨地区养老服务协作机制，鼓励发达地区支援欠发达地区。
　　（五）繁荣养老服务消费市场。
　　拓展养老服务内容。各地要积极发展养老服务业，引导养老服务企业和机构优先满足老年人基本服务需求，鼓励和引导相关行业积极拓展适合老年人特点的文化娱乐、体育健身、休闲旅游、健康服务、精神慰藉、法律服务等服务，加强残障老年人专业化服务。
　　开发老年产品用品。相关部门要围绕适合老年人的衣、食、住、行、医、文化娱乐等需要，支持企业积极开发安全有效的康复辅具、食品药品、服装服饰等老年用品用具和服务产品，引导商场、超市、批发市场设立老年用品专区专柜；开发老年住宅、老年公寓等老年生活设施，提高老年人生活质量。引导和规范商业银行、保险公司、证券公司等金融机构开发适合老年人的理财、信贷、保险等产品。
　　培育养老产业集群。各地和相关行业部门要加强规划引导，在制定相关产业发展规划中，要鼓励发展养老服务中小企业，扶持发展龙头企业，实施品牌战略，提高创新能力，形成一批产业链长、覆盖领域广、经济社会效益显著的产业集群。健全市场规范和行业标准，确保养老服务和产品质量，营造安全、便利、诚信的消费环境。
　　（六）积极推进医疗卫生与养老服务相结合。
　　推动医养融合发展。各地要促进医疗卫生资源进入养老机构、社区和居民家庭。卫生管理部门要支持有条件的养老机构设置医疗机构。医疗机构要积极支持和发展养老服务，有条件的二级以上综合医院应当开设老年病科，增加老年病床数量，做好老年慢病防治和康复护理。要探索医疗机构与养老机构合作新模式，医疗机构、社区卫生服务机构应当为老年人建立健康档案，建立社区医院与老年人家庭医疗契约服务关系，开展上门诊视、健康查体、保健咨询等服务，加快推进面向养老机构的远程医疗服务试点。医疗机构应当为老年人就医提供优先优惠服务。
　　健全医疗保险机制。对于养老机构内设的医疗机构，符合城镇职工（居民）基本医疗保险和新型农村合作医疗定点条件的，可申请纳入定点范围，入住的参保老年人按规定享受相应待遇。完善医保报销制度，切实解决老年人异地就医结算问题。鼓励老年人投保健康保险、长期护理保险、意外伤害保险等人身保险产品，鼓励和引导商业保险公司开展相关业务。
　　**三、政策措施**
　　（一）完善投融资政策。要通过完善扶持政策，吸引更多民间资本，培育和扶持养老服务机构和企业发展。各级政府要加大投入，安排财政性资金支持养老服务体系建设。金融机构要加快金融产品和服务方式创新，拓宽信贷抵押担保物范围，积极支持养老服务业的信贷需求。积极利用财政贴息、小额贷款等方式，加大对养老服务业的有效信贷投入。加强养老服务机构信用体系建设，增强对信贷资金和民间资本的吸引力。逐步放宽限制，鼓励和支持保险资金投资养老服务领域。开展老年人住房反向抵押养老保险试点。鼓励养老机构投保责任保险，保险公司承保责任保险。地方政府发行债券应统筹考虑养老服务需求，积极支持养老服务设施建设及无障碍改造。
　　（二）完善土地供应政策。各地要将各类养老服务设施建设用地纳入城镇土地利用总体规划和年度用地计划，合理安排用地需求，可将闲置的公益性用地调整为养老服务用地。民间资本举办的非营利性养老机构与政府举办的养老机构享有相同的土地使用政策，可以依法使用国有划拨土地或者农民集体所有的土地。对营利性养老机构建设用地，按照国家对经营性用地依法办理有偿用地手续的规定，优先保障供应，并制定支持发展养老服务业的土地政策。严禁养老设施建设用地改变用途、容积率等土地使用条件搞房地产开发。
　　（三）完善税费优惠政策。落实好国家现行支持养老服务业的税收优惠政策，对养老机构提供的养护服务免征营业税，对非营利性养老机构自用房产、土地免征房产税、城镇土地使用税，对符合条件的非营利性养老机构按规定免征企业所得税。对企事业单位、社会团体和个人向非营利性养老机构的捐赠，符合相关规定的，准予在计算其应纳税所得额时按税法规定比例扣除。各地对非营利性养老机构建设要免征有关行政事业性收费，对营利性养老机构建设要减半征收有关行政事业性收费，对养老机构提供养老服务也要适当减免行政事业性收费，养老机构用电、用水、用气、用热按居民生活类价格执行。境内外资本举办养老机构享有同等的税收等优惠政策。制定和完善支持民间资本投资养老服务业的税收优惠政策。
　　（四）完善补贴支持政策。各地要加快建立养老服务评估机制，建立健全经济困难的高龄、失能等老年人补贴制度。可根据养老服务的实际需要，推进民办公助，选择通过补助投资、贷款贴息、运营补贴、购买服务等方式，支持社会力量举办养老服务机构，开展养老服务。民政部本级彩票公益金和地方各级政府用于社会福利事业的彩票公益金，要将50%以上的资金用于支持发展养老服务业，并随老年人口的增加逐步提高投入比例。国家根据经济社会发展水平和职工平均工资增长、物价上涨等情况，进一步完善落实基本养老、基本医疗、最低生活保障等政策，适时提高养老保障水平。要制定政府向社会力量购买养老服务的政策措施。
　　（五）完善人才培养和就业政策。教育、人力资源社会保障、民政部门要支持高等院校和中等职业学校增设养老服务相关专业和课程，扩大人才培养规模，加快培养老年医学、康复、护理、营养、心理和社会工作等方面的专门人才，制定优惠政策，鼓励大专院校对口专业毕业生从事养老服务工作。充分发挥开放大学作用，开展继续教育和远程学历教育。依托院校和养老机构建立养老服务实训基地。加强老年护理人员专业培训，对符合条件的参加养老护理职业培训和职业技能鉴定的从业人员按规定给予相关补贴，在养老机构和社区开发公益性岗位，吸纳农村转移劳动力、城镇就业困难人员等从事养老服务。养老机构应当积极改善养老护理员工作条件，加强劳动保护和职业防护，依法缴纳养老保险费等社会保险费，提高职工工资福利待遇。养老机构应当科学设置专业技术岗位，重点培养和引进医生、护士、康复医师、康复治疗师、社会工作者等具有执业或职业资格的专业技术人员。对在养老机构就业的专业技术人员，执行与医疗机构、福利机构相同的执业资格、注册考核政策。
　　（六）鼓励公益慈善组织支持养老服务。引导公益慈善组织重点参与养老机构建设、养老产品开发、养老服务提供，使公益慈善组织成为发展养老服务业的重要力量。积极培育发展为老服务公益慈善组织。积极扶持发展各类为老服务志愿组织，开展志愿服务活动。倡导机关干部和企事业单位职工、大中小学学生参加养老服务志愿活动。支持老年群众组织开展自我管理、自我服务和服务社会活动。探索建立健康老人参与志愿互助服务的工作机制，建立为老志愿服务登记制度。弘扬敬老、养老、助老的优良传统，支持社会服务窗口行业开展“敬老文明号”创建活动。
　　**四、组织领导**
　　（一）健全工作机制。各地要将发展养老服务业纳入国民经济和社会发展规划，纳入政府重要议事日程，进一步强化工作协调机制，定期分析养老服务业发展情况和存在问题，研究推进养老服务业加快发展的各项政策措施，认真落实养老服务业发展的相关任务要求。民政部门要切实履行监督管理、行业规范、业务指导职责，推动公办养老机构改革发展。发展改革部门要将养老服务业发展纳入经济社会发展规划、专项规划和区域规划，支持养老服务设施建设。财政部门要在现有资金渠道内对养老服务业发展给予财力保障。老龄工作机构要发挥综合协调作用，加强督促指导工作。教育、公安消防、卫生计生、国土、住房城乡建设、人力资源社会保障、商务、税务、金融、质检、工商、食品药品监管等部门要各司其职，及时解决工作中遇到的问题，形成齐抓共管、整体推进的工作格局。
　　（二）开展综合改革试点。国家选择有特点和代表性的区域进行养老服务业综合改革试点，在财政、金融、用地、税费、人才、技术及服务模式等方面进行探索创新，先行先试，完善体制机制和政策措施，为全国养老服务业发展提供经验。
　　（三）强化行业监管。民政部门要健全养老服务的准入、退出、监管制度，指导养老机构完善管理规范、改善服务质量，及时查处侵害老年人人身财产权益的违法行为和安全生产责任事故。价格主管部门要探索建立科学合理的养老服务定价机制，依法确定适用政府定价和政府指导价的范围。有关部门要建立完善养老服务业统计制度。其他各有关部门要依照职责分工对养老服务业实施监督管理。要积极培育和发展养老服务行业协会，发挥行业自律作用。
　　（四）加强督促检查。各地要加强工作绩效考核，确保责任到位、任务落实。省级人民政府要根据本意见要求，结合实际抓紧制定实施意见。国务院相关部门要根据本部门职责，制定具体政策措施。民政部、发展改革委、财政部等部门要抓紧研究提出促进民间资本参与养老服务业的具体措施和意见。发展改革委、民政部和老龄工作机构要加强对本意见执行情况的监督检查，及时向国务院报告。国务院将适时组织专项督查。

**关于进一步加强老年人优待工作的意见**

各省、自治区、直辖市及新疆生产建设兵团老龄工作委员会办公室、高级人民法院、党委宣传部、发展改革委、科技厅(委、局)、公安厅(局)、民政厅(局)、司法厅(局)、财政厅(局)、人力资源社会保障厅(局)、住房城乡建设厅(委、局)、交通厅(委、局)、农业厅(局、委)、商务主管部门、文化厅(局)、卫生计生委(卫生厅、局、人口计生委)、新闻出版局、广电局、体育局(委)、林业厅(局)、旅游局(委)、铁路局、民航管理局、文物局、总工会：

　老年人优待是政府和社会在做好公民社会保障和基本公共服务的基础上，在医、食、住、用、行、娱等方面，积极为老年人提供的各种形式的经济补贴、优先优惠和便利服务。做好老年人优待工作，是增进老年人福祉的重要举措，也是社会文明进步的重要标志。根据新修订的《中华人民共和国老年人权益保障法》和《中共中央、国务院关于加强老龄工作的决定》的有关规定，现就进一步加强老年人优待工作，提出以下意见：

**一、总体要求**

　　(一)指导思想

　　以邓小平理论、“三个代表”重要思想、科学发展观为指导，立足我国基本国情和经济社会发展现状，针对老年人的特殊需求，积极完善优待政策法规体系，逐步拓展优待项目和范围、创新优待工作方式、提升优待水平，让老年人更好地共享经济社会发展成果，不断提升老年人生活质量。

　　(二)基本原则

　　——政府主导，社会参与。发挥政府在政策制定、督查检查、示范引领方面的主导作用，在社会保障、基本公共服务等方面积极为老年人提供优待，采取措施鼓励、引导社会力量参与优待工作。

　　——因地制宜，积极推进。根据经济社会发展实际，合理确定优待范围、优待对象和优待标准。积极推进优待工作，坚持积极稳妥、循序渐进，稳步提升。

　　——突出重点，适度普惠。从不同老年群体的实际需求出发，对各优待项目的服务对象进行细分，优先考虑高龄、失能等困难老年群体的特殊需要，逐步发展面向老年人的普惠性优待项目。

　　——统筹协调，和谐共融。统筹社会优待与社会保障、优待工作与老龄事业、物质帮助与精神关爱协调发展；统筹推进城乡老年人优待工作，加快发展农村老年人优待项目；统筹不同年龄群体的利益诉求，促进代际共融与社会和谐。

　　(三)主要目标

　　2015年，实现县级以上地方人民政府全面建立健全老年人优待政策，社会敬老氛围更加浓厚，各项优待规定得到有效落实；2020年，实现优待工作管理进一步规范，优待项目进一步拓展，优待水平进一步提升，老年人过上更加幸福的小康生活。

**二、优待项目和范围**

　　优待的基本对象为60周岁以上的老年人。各地可因地制宜，在本意见基础上合理确定优待对象和优待标准，率先在卫生保健、交通出行、商业服务、文体休闲等方面，对常住本行政区域内的老年人给予同等优待，并根据本地实际情况，逐步拓展同等优待范围。

　　(一)政务服务优待

　　1.各地在落实和完善社会保障制度和公共服务政策时，应对老年人予以适度倾斜。

　　2.鼓励地方建立八十周岁以上低收入老年人高龄津贴制度。

　　3.政府投资兴办的养老机构，要在保障“三无”老年人、“五保”老年人服务需求的基础上，优先照顾经济困难的孤寡、失能、高龄老年人。

　　4.各地对经济困难的老年人要逐步给予养老服务补贴。对生活长期不能自理、经济困难的老年人，要根据其失能程度等情况给予护理补贴。

　　5.各地在实施廉租住房、公共租赁住房等住房保障制度时，要照顾符合条件的老年人，优先配租配售保障性住房；进行危旧房屋改造时，优先帮助符合条件的老年人进行危房改造。

　　6.政府有关部门要为老年人及时、便利地领取养老金、结算医疗费和享受其他物质帮助，创造条件，提供便利。鼓励和引导公共服务机构、社会志愿服务组织优先为老年人提供服务。

　　7.政府有关部门在办理房屋权属关系变更等涉及老年人权益的重大事项时，应依法优先办理，并就办理事项是否为老年人的真实意愿进行询问，有代理人的要严格审查代理资格。

　　8.免除农村老年人兴办公益事业的筹劳任务。经农村集体经济组织全体成员同意，将未承包的集体所有的部分土地、山林、水面、滩涂等作为养老基地，收益供老年人养老，纳入国家和地方湿地保护体系及其自然保护区的重要湿地除外。

　　9.政府有关部门要完善老年人社会参与方面的支持政策，充分发挥老年人参与社会发展的积极性和创造性。

　　10.对有老年人去世的城乡生活困难家庭，减免其基本殡葬服务费用，或者为其提供基本殡葬服务补贴。对有老年人去世的家庭，选择生态安葬方式的，或者在土葬改革区自愿实行火葬的，要给予补贴或奖励。

　　(二)卫生保健优待

　　11.医疗卫生机构要优先为辖区内65周岁以上常住老年人免费建立健康档案，每年至少提供1次免费体格检查和健康指导，开展健康管理服务。定期对老年人进行健康状况评估，及时发现健康风险因素，促进老年疾病早发现、早诊断、早治疗。积极开展老年疾病防控的知识宣传，开展老年慢性病和老年期精神障碍的预防控制工作。为行动不便的老年人提供上门服务。

　　12.鼓励设立老年病医院，加强老年护理院、老年康复医院建设，有条件的二级以上综合医院应设立老年病科。

　　13.医疗卫生机构应为老年人就医提供方便和优先优惠服务。通过完善挂号、诊疗系统管理，开设专用窗口或快速通道、提供导医服务等方式，为老年人特别是高龄、重病、失能老年人挂号(退换号)、就诊、转诊、综合诊疗提供便利条件。

　　14.鼓励各地医疗机构减免老年人普通门诊挂号费和贫困老年人诊疗费。提倡为老年人义诊。

　　15.倡导医疗卫生机构与养老机构之间建立业务协作机制，开通预约就诊绿色通道，协同做好老年人慢性病管理和康复护理，加快推进面向养老机构的远程医疗服务试点，为老年人提供便捷、优先、优惠的医疗服务。

　　16.支持符合条件的养老机构内设医疗机构，申请纳入城镇职工(居民)基本医疗保险和新型农村合作医疗定点范围。

　　(三)交通出行优待

　　17.城市公共交通、公路、铁路、水路和航空客运，要为老年人提供便利服务。

　　18.交通场所和站点应设置老年人优先标志，设立等候专区，根据需要配备升降电梯、无障碍通道、无障碍洗手间等设施。对于无人陪同、行动不便的老年人给予特别关照。

　　19.城市公共交通工具应为老年人提供票价优惠，鼓励对65周岁以上老年人实行免费，有条件的地方可逐步覆盖全体老年人。各地可根据实际情况制定具体的优惠办法，对落实老年优待任务的公交企业要给予相应经济补偿。

　　20.倡导老年人投保意外伤害保险，保险公司对参保老年人应给予保险费、保险金额等方面的优惠。

　　21.公共交通工具要设立不低于坐席数10%的“老幼病残孕”专座。铁路部门要为列车配备无障碍车厢和座位，对有特殊需要的老年人订票和选座位提供便利服务。

　　22.严格执行《无障碍环境建设条例》、《社区老年人日间照料中心建设标准》和《养老设施建筑设计规范》等建设标准，重点做好居住区、城市道路、商业网点、文化体育场馆、旅游景点等场所的无障碍设施建设，优先推进坡道、电梯等与老年人日常生活密切相关的公共设施改造，适当配备老年人出行辅助器具，为老年人提供安全、便利、舒适的生活和出行环境。

　　23.公厕应配备便于老年人使用的无障碍设施，并对老年人实行免费。

　　(四)商业服务优待

　　24.各地要根据老年人口规模和消费需求，合理布局商业网点，有条件的商场、超市设立老年用品专柜。

　　25.商业饮食服务网点、日常生活用品经销单位，以及水、电、暖气、燃气、通讯、电信、邮政等服务行业和网点，要为老年人提供优先、便利和优惠服务。

　　26.金融机构应为老年人办理业务提供便捷服务，设置老年人取款优先窗口，并提供导银服务，对有特殊困难、行动不便的老年人提供特需服务或上门服务。鼓励对养老金客户实施减费让利，对异地领取养老金的客户减免手续费。对办理转账、汇款业务或购买金融产品的老年人，应提示相应风险。

　　(五)文体休闲优待

　　27.各级各类博物馆、美术馆、科技馆、纪念馆、公共图书馆、文化馆等公共文化服务设施，向老年人免费开放。减免老年人参观文物建筑及遗址类博物馆的门票。

　　28.公共文化体育部门应对老年人优惠开放，免费为老年人提供影视放映、文艺演出、体育赛事、图片展览、科技宣传等公益性流动文化体育服务。关注农村老年人文化体育需求，适当安排面向农村老年人的专题专场公益性文化体育服务。

　　29.公共文化体育场所应为老年人健身活动提供方便和优惠服务，安排一定时段向老年人减免费用开放，有条件的可适当增加面向老年人的特色文化体育服务项目。提倡体育机构每年为老年人进行体质测定，为老年人体育健身提供咨询、服务和指导，提高老年人科学健身水平。

　　30.提倡经营性文化体育单位对老年人提供优待。鼓励影剧院、体育场馆为老年人提供优惠票价，为老年文艺体育团体优惠提供场地。

　　31.公园、旅游景点应对老年人实行门票减免，鼓励景区内的观光车、缆车等代步工具对老年人给予优惠。

　　32.老年活动场所、老年教育资源要对城乡老年人公平开放，公共教育资源应为老年人学习提供指导和帮助。贫困老年人进入老年大学(学校)学习的，给予学费减免。

　　(六)维权服务优待

　　33.各级人民法院对侵犯老年人合法权益的案件，要依法及时立案受理、及时审判和执行。

　　34.司法机关应开通电话和网络服务、上门服务等形式，为高龄、失能等行动不便的老年人报案、参与诉讼等提供便利。

　　35.老年人因其合法权益受到侵害提起诉讼，需要律师帮助但无力支付律师费用的，可依法获得法律援助。对老年人提出的法律援助申请，要简化程序，优先受理、优先审查和指派。各地可根据经济社会发展水平，适度放宽老年人经济困难标准，将更多与老年人权益保护密切相关的事项纳入法律援助补充事项范围，扩大老年人法律援助覆盖面。

　　36.要健全完善老年人法律援助体系，不断拓展老年人申请法律援助的渠道，科学设置基层法律援助站点，简化程序和手续，为老年人就近申请和获得法律援助提供便利条件。

　　37.老年人因追索赡养费、扶养费、养老金、退休金、抚恤金、医疗费、劳动报酬、人身伤害事故赔偿金等提起诉讼，交纳诉讼费确有困难的，可以申请司法救助，缓交、减交或者免交诉讼费。因情况紧急需要先予执行的，可依法裁定先予执行。

　　38.鼓励律师事务所、公证处、司法鉴定机构、基层法律服务所等法律服务机构，为经济困难的老年人提供免费或优惠服务。

**三、组织实施**

　　(一)切实加强领导。各地要高度重视老年人优待工作，健全政府主导、老龄委组织协调、相关部门各司其职、企事业单位和社会团体以及志愿者积极参与的工作体制和运行机制。要保障老年人优待工作经费，进一步落实各项财税优惠政策，调动社会力量积极参与。加强对老年人优待工作年度目标责任考核，确保责任到位、任务落实。县级以上地方人民政府和相关部门要结合实际制定老年人优待政策和具体实施办法。

　　(二)协力推进实施。优待老年人是全社会的共同责任。国家机关、社会团体、企事业单位和其他组织，都要履行为老年人提供优待的职责义务，积极为老年人提供优待服务。各级涉老主管单位要规范服务，加强管理，督促各优待服务场所、设施和窗口设置优待标识，公布优待内容。有关部门要加强尊老敬老思想教育和道德宣传、老年维权法制教育活动，增强社会成员优待老年人的自觉性，提高老年人自我维权意识和能力。深入推进“敬老爱老助老”主题教育、“敬老文明号”和“老年人维权示范岗”活动，在全社会弘扬孝亲敬老传统美德，进一步营造尊重老年人的社会氛围。

　　(三)监督检查落实。各级老龄工作委员会负责老年人优待工作的组织协调和监督指导，各级老龄工作委员会办公室承担老年人优待工作的日常事务管理，要会同有关部门定期开展监督检查。要进一步发挥行政监督和社会监督的作用，建立健全信息反馈和监督机制，设立服务和监督热线，依法妥善解决好举报和投诉问题，对老年人优待工作中反映强烈的突出问题，要尽早发现、及时解决。对不按规定履行优待老年人义务的，由有关主管部门责令改正。

**商务部关于推动养老服务产业发展的指导意见**

各省、自治区、直辖市、计划单列市及新疆生产建设兵团商务主管部门：

当前，我国已经进入人口老龄化快速发展阶段。积极应对人口老龄化，加快发展养老服务业，不断满足老年人持续增长的养老服务需求，是全面建成小康社会的一项紧迫任务。商务部门在养老服务产业化发展中有义不容辞的责任，要在健全家政服务体系建设的基础上，加快推动居家养老、社区养老和集中养老的发展，探索以市场化方式发展养老服务产业的新途径、新模式。为全面贯彻落实党的十八届三中全会精神和《国务院关于发展养老服务业的若干意见》（国发〔2013〕35号文），充分发挥社会力量的主体作用，扩大养老服务产业规模，推动养老服务产业化发展，现提出以下意见：

**一、工作目标**

通过推动养老服务产业发展，建成功能完善、规模适度、覆盖城乡的养老服务产业化发展模式，形成各具特色的典型经验、先进做法和可持续、可复制的政策措施及体制机制创新成果。探索多元化发展的居家养老服务体系，努力使城市居家养老服务网络实现全覆盖，服务设施不断充实，服务内容和形式不断丰富，服务队伍不断扩大；建设运作规范的社区日间照料中心、老年人活动中心以及农村养老服务综合设施和站点；培育一批带动力强的龙头企业、富有创新活力的中小企业，竞争力强、经济社会效益显著的服务机构和产业集聚群以及知名养老服务品牌等，为养老服务产业中长期发展奠定基础和积累经验。

**二、工作任务**

（一）加快推动居家养老服务的多元化发展。

依托非政府组织、社区组织、企业和社区医院等多种供给主体，建立健全省、市、县、乡镇（街道）、村（社区）等不同层次的居家养老服务网络，满足多层次的居家养老服务需求。鼓励社会中介组织、家政服务企业等社会力量参与居家养老服务，提供日常生活、医疗保健、精神生活、法律咨询等养老服务需求。重点支持有实力且运作规范的家政服务企业承担居家养老服务任务，为有需求的老年人提供助餐、助浴、助洁、助急、助医等定制服务，完善居家养老服务体系。

（二）加快推动社区养老服务的便利化发展。

依托社区综合服务设施，整合社区服务资源，按照有关标准建设老年人日间照料中心、老年人活动中心等社区养老服务设施。创建一批服务设施完善、信息网络健全、管理服务规范的养老服务示范社区。努力使符合标准的老年人日间照料中心、老年人活动中心等服务设施覆盖所有城市社区，90%的乡镇和60%以上的农村社区建设包括居家养老服务在内的社区综合服务设施和站点。鼓励家政服务企业参与社区养老服务体系建设，丰富养老服务内容，拓展养老服务项目，增强社区养老服务功能，提供便捷的养老服务。

（三）加快推动集中养老服务的特色化发展。

鼓励各类市场主体针对养老服务需求，通过市场化运作方式，参与集中养老服务体系建设。制订城市总体规划、控制性详细规划时，要分区分级规划设置养老服务设施。鼓励扶持民办养老服务机构发展，全面推进养老机构向社会延伸服务，形成理疗、美食、休闲、娱乐、健身等各具特色的集中养老服务模式，探索景区养老、生态养老、田园养老等集聚式养老发展模式。

（四）加快推动养老服务的信息化发展。

依托已建的家政服务网络中心等现有信息服务资源，建立统一的养老服务信息平台。全面调查老年人基本现状和养老服务需求，规范收集养老服务行业基础数据；按照“分类建档、分层服务”的原则，为每位老年人建立个人服务需求档案；提供养老服务信息咨询服务，对接老年人服务需求和各类社会主体服务供给；有条件的地方，要为高龄老人、低收入失能老人免费配置电子呼叫设备；完善紧急呼叫监控服务设施，提高服务的便捷化和可及性；支持养老机构建立以采集老年人信息、服务缴费、日常管理的信息系统；鼓励养老服务企业加强信息管理系统建设，提升养老服务能力。

（五）加快推动养老服务的融合发展。

一是推动居家养老与社区养老的融合。充分发挥社区一对多的优势，鼓励个人利用家庭资源在社区开展助老服务，倡导邻里相助和结对帮扶。推动社区养老服务进家庭，探索建立健康老人参与社区志愿互助服务的工作机制，逐步形成居家养老为基础，社区养老为补充的融合发展模式。二是推动居家养老服务、社区养老服务与集中养老服务的融合。发挥居家养老、社区养老和集中养老的各自优势，取长补短，开展互利合作，逐步实现居家养老、社区养老和集中养老在服务对象、服务信息、服务人员、服务标准和服务管理等方面的共享。三是推动养老服务与医疗卫生的融合。推进医养结合，构建居家养老与医疗相互融合的服务模式；鼓励有条件的医疗机构为患有严重疾病的老年人提供上门服务；支持有条件的综合医院派出医护人员走进社区，普及专业性知识；引导养老服务机构和医疗服务机构合作，开展养老服务人员培训，提高老年人健康管理服务水平；建立养老服务机构与医疗、保健机构长期稳定的契约合作关系，通过调整卫生资源配置，利用技术设备，为老年人提供日常医疗保健和咨询服务。四是推动养老服务与关联行业的融合。鼓励住宿、餐饮、居民生活服务、批发、零售等行业针对老年人的特殊需求，及时提供有效的服务；支持生产企业针对老年人的需求，研发、生产老年人的适用产品。

**三、工作重点**

（六）培育龙头企业。

加快培育运作规范的养老服务企业，增强养老服务保障和调控市场的能力，打造一批养老服务品牌。鼓励养老服务企业提高专业化、产业化程度，形成一批有影响力的大型养老服务企业集团。支持养老服务企业利用现代科技手段和信息技术，提升养老服务业发展水平，增强可持续发展能力。鼓励大型养老服务企业建立服务人员供应保障基地，增强其人员保障能力。支持大型养老服务企业建立从业人员信用档案，完善企业信息采集、利用、查询、披露制度。推动大型养老服务企业发展连锁经营，支持其开展质量管理体系认证，开展商标、专利注册和保护工作。支持养老服务企业上市融资，增强其可持续发展能力。

（七）丰富服务内容。

鼓励家政服务企业积极承担居家养老、社区养老和集中养老服务，推动家庭自主养老、完善居家和社区养老、充分利用机构养老、鼓励社会志愿参与养老、倡导社区内互助养老，形成以家政服务企业为主体、其他社会力量广泛参与、政府协助的养老服务体系。一是保障日常生活服务。为居住在家中的老年人提供日常生活照料、购物、家政、家庭理财等服务。二是提供医疗保健服务。为老年人提供疾病防治、康复护理、心理卫生、健康教育、应急救助、体育健身等服务。三是丰富精神生活服务。为老年人提供精神抚慰、知识讲座、学习培训、娱乐活动等服务。四是开展法律咨询服务。为老年人提供法律咨询、法律援助、司法维权等服务。五是开发养老服务产品和拓展服务形式。加强与实力强、品牌优、信誉好的企业合作，为老年人提供功能多、效果好、价格优的老年用食品、老年用品、医疗保健产品等；鼓励开发适合老年人居住的休闲养老公寓、保健项目和老年生活设施，引导金融机构开发适合老年人的理财、信贷、保险等产品，探索老年人住房反向抵押养老保险制度；引导商场、超市、批发市场设立老年用品专区专柜，切实为老年人提供更多更好的服务。

（八）创新服务模式。

发挥已建成的家政服务网络中心作用，开通养老服务专线，为老年人提供标准化、专业化、亲情化服务，推动养老服务信息化发展。一是开展订单服务。根据订单信息和老年人消费需求，社区居家养老服务点或委托区域内其他企业派服务人员上门提供日常生活照料服务，满足老年人多元化、专业化和个性化需求。二是发展网络服务。探索面向居家养老服务机构的远程医疗保健、养老护理等服务。建设综合性的养老服务信息平台，提供紧急呼叫、家政预约、健康咨询、物品代购、服务缴费等适合老年人的服务项目。三是开发日托服务。根据老年人需求和服务网点实际，采取“朝至夕归”养老或全日托养老模式，提供生活照料、餐饮服务、心理咨询、心理健康、医疗保健、文化娱乐、代订代购、网络购物等服务项目。

（九）强化服务质量。

从硬件设施、服务标准、服务功能、软件系统、互联互通等方面制订网点建设标准和从业人员服务标准，加大标准的宣传贯彻力度。研究制订居家养老服务培训大纲，选定培训教材，完善培训流程，加强培训管理，培养合格的养老服务人员。要加强管理人员培训，提高企业管理水平。坚持培训工作与职业技能鉴定工作相结合，鼓励和支持服务人员参加国家职业技能鉴定考试，切实提高从业人员技术水平。建立养老服务对象和企业、从业人员的评估指标体系，形成科学合理的评价标准。利用信息系统进行跟踪回访，加强对企业和从业人员服务质量的监管和考核力度。

**四、工作要求**

（十）加强组织领导。

要高度重视养老服务产业发展，加强此项工作的组织领导，积极协调财政、发展改革、民政、规划、城建、人力资源、工商、税务、妇联等部门，完善政策措施，创新工作方式，合力推动养老服务产业化发展。

（十一）制订规划和方案。

加快制订养老服务产业发展规划，内容主要包括发展目标、主要任务、工作重点、实施步骤、保障措施等。工作方案主要包括工作目标、工作任务、工作重点、实施计划、工作进度、保障措施、效果评估等。

（十二）配套相关政策。

一是完善税费政策。全面落实国家关于发展家庭服务业、养老服务业等相关的税费扶持政策，根据建设中发现的新情况，及时研究制订推进养老产业化发展的政策措施，努力构建良好的政策环境。二是保障项目用地。优先保障养老重点项目用地，制订和落实用地优惠政策。探索以土地作价入股、土地租赁等形式支持养老服务体系建设。对于政府投资建设的不以盈利为目的、具有公益性质的居家养老服务网点和家政服务网络中心，可按作价出资方式办理用地手续，但禁止改变用途和性质。三是加强金融支持。加大养老服务体系建设的贷款投入力度，加强对居家养老服务企业和养老服务产品供应链上下游企业的信贷支持。鼓励金融机构针对养老服务企业特点开展动产、网点经营权、租赁权等质押融资以及小额贷款保证保险，支持融资担保公司提供融资担保服务，提高对中小养老服务企业的融资担保能力。四是加大保险补贴。加快研究制订促进员工制养老服务企业快速发展的保险补贴政策措施，促进企业向员工制企业发展。鼓励商业保险机构开发养老服务保险产品，推行养老服务机构职业责任险、人生意外险等险种，防范和化解风险。五是加强收费管理。对养老服务企业收取服务费，要实施收费公示制度，每年至少公示一次养老服务价格。发挥养老基金作用，鼓励居家养老服务企业对低收入、家庭困难的老年人提供基本居家养老免费服务。

（十三）开展绩效评估。

要及时开展工作评估，总结工作中存在的问题、采取的主要措施、工作的实施效果、推动养老服务产业发展的不同模式。要按照绩效评估的要求，加强跟踪管理，开展年度绩效评估，并于第二年1月底前将上年工作的绩效评估报告报商务部（服贸司）。

（十四）加大宣传力度。

通过新闻媒体和政府公众信息网，大力宣传推进养老服务产业化发展的重要意义和政策措施，宣传先进典型及成功经验，扩大社会影响，调动社会资本积极参与产业发展和老年人消费的积极性，在全社会形成关心、支持发展养老服务行业的良好氛围。

**国家卫生计生委办公厅关于印发老年健康核心信息的通知**

各省、自治区、直辖市卫生计生委，新疆生产建设兵团卫生局：
　　为增强老年人健康意识，进一步开展老年健康教育，营造全社会关心支持老年健康的社会氛围，提高老年人健康素养水平，我委组织专家编写了老年健康核心信息。现印发各地，供参考使用。

国家卫生计生委办公厅
2014年10月7日

老年健康核心信息

　　1.积极认识老龄化和衰老。老年人要不断强化自我保健意识，学习自我监护知识，掌握自我管理技能，早期发现和规范治疗疾病，对于中晚期疾病以维持功能为主。
　　2.合理膳食，均衡营养。老年人饮食要定时、定量，每日食物品种应包含粮谷类、杂豆类及薯类（粗细搭配），动物性食物，蔬菜、水果，奶类及奶制品，以及坚果类等，控制烹调油和食盐摄入量。建议老年人三餐两点，一日三餐能量分配为早餐约30%，午餐约40%，晚餐约30%，上下午各加一次零食或水果。
　　3.适度运动，循序渐进。老年人最好根据自身情况和爱好选择轻中度运动项目，如快走、慢跑、游泳、舞蹈、太极拳等。上午10～11点和下午3～5点为最佳运动时间，每次运动时间30～60分钟为宜。
　　4.及早戒烟，限量饮酒。戒烟越早越好。如饮酒，应当限量，避免饮用45度以上烈性酒，切忌酗酒。
　　5.保持良好睡眠。每天最好午休1小时左右。如果长期入睡困难或有严重的打鼾并呼吸暂停者，应当及时就医。如使用安眠药，请遵医嘱。
　　6.定期自我监测血压。测前应当休息5分钟，避免情绪激动、劳累、吸烟、憋尿。每次测量两遍，间隔1分钟，取两次的平均值。高血压患者每天至少自测血压3次（早、中、晚各1次）。警惕血压晨峰现象，防止心肌梗死和脑卒中；同时应当避免血压过低，特别是由于用药不当所致的低血压。
　　7.定期监测血糖。老年人应该每1～2个月监测血糖一次，不仅要监测空腹血糖，还要监测餐后2小时血糖。糖尿病患者血糖稳定时，每周至少监测1～2次血糖。老年糖尿病患者血糖控制目标应当适当放宽，空腹血糖＜7.8毫摩/升，餐后2小时血糖＜11.1毫摩/升，或糖化血红蛋白水平控制在7.0%～7.5%即可。
　　8.预防心脑血管疾病。老年人应当保持健康生活方式，控制心脑血管疾病危险因素。如控制油脂、盐分的过量摄入，适度运动，保持良好睡眠，定期体检，及早发现冠心病和脑卒中的早期症状，及时治疗。
　　9.关注脑卒中早期症状，及早送医。一旦发觉老年人突然出现一侧面部或肢体无力或麻木，偏盲，语言不利，眩晕伴恶心、呕吐，复视等症状，必须拨打“120”，紧急送到有条件的医院救治。
　　10.重视视听功能下降。避免随便挖耳；少喝浓茶、咖啡；严格掌握应用耳毒性药物（如庆大霉素、链霉素等）的适应证；力求相对安静的生活环境。听力下降严重时，老年人要及时到医疗机构检查，必要时佩戴助听器。定期检查视力，发现视力下降及时就诊。
　　11.重视口腔保健。坚持饭后漱口、早晚刷牙，合理使用牙线或牙签；每隔半年进行1次口腔检查，及时修补龋齿孔洞；及时镶补缺失牙齿，尽早恢复咀嚼功能。
　　12.预防跌倒。老年人90%以上的骨折由跌倒引起。平时应当保持适度运动，佩戴适当的眼镜以改善视力，避免单独外出和拥挤环境，室内规则摆放物品，增加照明，保持地面干燥及平整。
　　13.预防骨关节疾病和预防骨质疏松症。注意膝关节保暖，避免过量体育锻炼，尽量少下楼梯，控制体重以减轻下肢关节压力。增加日晒时间。提倡富含钙、低盐和适量蛋白质的均衡饮食，通过步行或跑步等适度运动提高骨强度。
　　14.预防压力性尿失禁。注意改变使腹压增高的行为方式和生活习惯，如长期站立、蹲位、负重、长期慢性咳嗽、便秘等。
　　15.保持良好心态，学会自我疏导。一旦发觉老年人出现失眠、头痛、眼花、耳鸣等症状，并且心情压抑、郁闷、坐卧不安，提不起精神，为一点儿小事提心吊胆、紧张恐惧，对日常活动缺乏兴趣，常常自卑、自责、内疚，处处表现被动和过分依赖，感到生活没有意义等或心情烦躁、疲乏无力、胸闷、睡眠障碍、体重下降、头晕头痛等抑郁症早期症状，要及时就诊，请专科医生进行必要的心理辅导和药物治疗。
　　16.预防阿尔茨海默病的发生发展。阿尔茨海默病多数起病于65岁以后，主要表现为持续进行性的记忆、语言、视空间障碍及人格改变等。老年人一旦出现记忆力明显下降、近事遗忘突出等早期症状，要及早就诊，预防或延缓阿尔茨海默病的发生发展。
　　17.合理用药。用药需严格遵守医嘱，掌握适应证、禁忌证，避免重复用药、多重用药。不滥用抗生素、镇静睡眠药、麻醉药、消炎止痛药、抗心律失常药、强心药等。不轻易采用“秘方”、“偏方”、“验方”、“新药”、“洋药”等。用药期间出现不良反应可暂时停药，及时就诊。
　　18.定期体检。老年人每年至少做1次体检，积极参与由政府和大型医院等组织的普查，高度重视异常肿块、肠腔出血、体重减轻等癌症早期危险信号，一旦发现异常应当去肿瘤专科医院就诊，发现癌症要去正规医院接受规范化治疗。早发现、早干预慢性疾病，采取有效干预措施，降低疾病风险。保存完整病历资料。
　　19.外出随身携带健康应急卡。卡上注明姓名、家庭住址、工作单位、家属联系方式等基本信息，患有哪些疾病，可能会发生何种情况及就地进行简单急救要点，必要时注明请求联系车辆、护送医院等事项。
　　20.促进老年人积极进行社会参与，结合自身情况参加有益身心健康的体育健身、文化娱乐等活动，提倡科学文明健康的生活方式。注重生殖健康，避免不安全性行为。倡导全社会关爱老年人，实现老有所养、老有所医、老有所为、老有所学、老有所乐。

**关于进一步加强新形势下老年人体育工作的意见**

为深入贯彻落实国务院关于加快发展体育产业促进体育消费、加快发展养老服务业、加快构建现代公共文化服务体系、促进健康服务业发展的精神，充分发挥体育在应对人口老龄化过程中的积极作用，推进全民健身事业全面发展，根据《[中华人民共和国体育法](javascript:SLC(167119))》、《[中华人民共和国老年人权益保障法](javascript:SLC(252608))》、《[全民健身条例](javascript:SLC(207834))》等有关法律法规，现就进一步加强新时期老年人体育工作提出如下意见。
　　一、充分认识加强老年人体育工作的重要性
　　（一）当前，我国已进入老龄化社会，对国家经济发展和社会和谐稳定提出了十分严峻的挑战，应对人口老龄化已经成为国家一项长期发展战略。尊老敬老是中华民族的传统美德，爱老助老是全社会的共同责任，党和政府始终高度重视和关心老龄工作，把老龄工作作为构建社会主义和谐社会、全面建成小康社会，实现中华民族伟大复兴的中国梦的重要组成部分。
　　（二）提高老年人健康水平是提高老年人生活质量的基础和前提。推进健康关口前移，延长健康寿命，增强自主活动能力，对于老年人自立自强、积极向上具有重要意义。体育健身活动是积极应对人口老龄化的便捷、经济、有效方式，也是老年人保持健康、延缓衰老的理想途径。老年人有迫切的健康长寿愿望，有强烈的体育健身热情，有大量的闲暇时间，因此，加强老年人体育工作对丰富老年人精神文化生活，提高老年人健康水平和生活质量具有不可替代的作用。
　　（三）老年人体育工作是我国老龄事业和体育事业的重要组成部分，要站在落实全民健身国家战略的高度，将老年人体育工作作为协调推进四个全面战略布局的重要举措，与经济发展、社会稳定、服务民生等工作紧密结合，充分认识新形势下进一步加强老年人体育工作的重要意义，认真贯彻落实党和政府有关老年人体育工作的各项方针政策，掌握新情况、适应新趋势，切实加强对老年人体育工作的指导与协调，大力发展老年人体育事业，维护和保障老年人体育健身权益。
　　（四）在发展全民健身事业和建设体育强国的过程中，抓好老年人体育工作是带动其他年龄人群参与全民健身的有效方法。改革开放以来，老年人体育工作蓬勃发展，并且随着经济发展、社会进步和人民生活水平的不断提高，老年人体育健身的需求越来越旺盛，参与体育健身的热情越来越高涨，但目前还普遍存在对老年人体育工作的重要性认识不充分，老年人体育组织网络不健全，老年人体育健身场地设施缺乏，老年人体育健身方式较少、科学性较差和经费投入不足等亟待解决的问题，新变化、新要求和新任务使老年人体育工作面临新的发展机遇和挑战。

二、明确老年人体育工作的目标任务。

(五)要努力探索并遵循老年人体育工作规律，拓展老年人体育工作的新领域、新空间和新路子。要注重社会化，加快政府职能转变，创造条件吸引社会力量参与老年人公共体育服务；要注重制度化，整合利用各种资源，促进老年人体育健身与养老服务、健康服务、公共文化服务、文化创意和设计、教育培训、医疗卫生、家政、保险、旅游等相关领域交互融通；要注重科学化，创新老年人体育公共服务方式，提高能力和质量，使基础更加坚实，产品更加丰富，供给更加充足，努力满足老年人日益增长的多元化体育健身需求；要注重生活化，重点推进基层老年人体育工作，切实解决老年人体育健身的实际问题，引导老年人养成健康、文明、科学的生活方式。

(六)要把增强老年人体质、提高健康水平、丰富精神文化生活作为新形势下老年人体育工作的根本任务，建立健全有中国特色的老年人体育工作理论体系和政策法规体系，定期制定并实施老年人体育发展规划，并纳入全民健身计划；要切实建立“党政主导、部门尽责、协会组织、社会支持、重在基层、面向全体”的老年人体育工作格局，以加强体育场地设施建设为基础，以完善体育组织网络为依托，以开展体育健身活动为手段，实现老年人体育工作有组织、有人员、有阵地、有经费，并确保持续健康发展；要逐步建成惠及全体老年人的公共体育服务体系，提供适应老年人需求的公共体育产品和服务，使经常参加体育健身活动的老年人数逐年递增，老年人体育健身和消费意识显著增强，人均体育消费支出明显提高。

三、建立健全老年人体育组织网络。

(七)建立健全老年人体育组织网络是加强老年人体育工作的重要前提和关键环节。要以“重在基层，面向全体”为工作方针，鼓励发展多种类型的老年人体育组织，满足老年人的不同健身需要。要鼓励、支持老年人体育组织自上而下延伸，县以上地区都要在民政部门依法登记成立老年人体育协会，在街道和乡镇普遍建立老年人基层文化体育组织，在城乡社区广泛建立老年人健身活动站点和体育健身团队，逐步形成并完善老年人体育组织网络。要加强对老年人体育组织的服务和引导，按照政社分开、管办分离的原则，切实帮助解决人、财、物和科学健身指导等方面的问题，提供办公和开展体育健身活动保障，保持人员队伍的稳定和活力，使老年人体育组织有人想事、管事、做事。

(八)老年人体育协会是为老年人体育健身提供服务的社会团体，是党和政府联系老年人的桥梁和纽带，是实现老年人公共体育服务职能的得力助手。要引导、支持各级老年人体协加强自身建设，健全工作机构，规范退(离)休领导干部在老年人体协兼职行为，聘用熟悉体育工作、组织协调能力较强的人员从事日常事务，不断提高老年人体协“自我发展、自我管理、自我服务、自律规范”的能力，增强吸引力、凝聚力，始终保持生机与活力。

(九)老年人体育工作始终与社区体育工作、农村体育工作紧密联系、相辅相成，体育部门要结合城乡社区老年人体育组织建设，充分发挥老年人和其他年龄人群体育健身的良性互动作用，街道办事处和乡镇政府要依托社区体育活动中心(站)、体育俱乐部、乡镇(街道)综合文化站等建立为老年人体育健身服务的基层文化体育组织。街道办事处和乡镇政府要通过对老年人体育健身活动站点和体育健身团队进行备案和以奖代补等形式予以扶持，使其成为老年人身边体育健身、文化娱乐的重要组织。

(十)要加强老年人体育工作骨干队伍建设。有计划、有针对性地培训建立服务老年人的社会体育指导员等志愿者队伍，不断提高其思想道德素质和服务能力，并充实到各级各类老年人体育组织；要加强老年人体育健身项目教练员、裁判员队伍建设，并对符合条件的颁发资格证书；要规范并加快培养服务老年人的职业社会体育指导员等从业人员，鼓励街道、乡镇聘用体育专业人才从事老年人体育健身服务工作，并与其他涉老组织在人员上统筹安排。

四、加强适合老年人体育健身的场地设施建设和使用。

(十一)体育健身场地设施是老年人开展体育健身活动的必要条件和重要保障。要根据《公共文化体育设施条例》，将适合老年人体育健身的场地设施纳入体育健身圈建设内容，不断健全适合老年人体育健身的场地设施设计和施工规范以及技术要求等标准；要按照均衡配置、规模适当、功能优先、经济适用、节能环保的原则，根据当地经济发展状况、老年人数量和分布、地域特点以及体育健身习惯等因素，将适合老年人体育健身的场地设施建设纳入规划，因地制宜地与其他服务老年人的场地设施建设项目统筹安排。

(十二)要拓宽适合老年人体育健身的场地设施建设和运行管理的投融资渠道，将适合老年人体育健身的基本公共体育场地设施建设列入各级政府财政预算和投资计划。集中使用的彩票公益金支持体育事业专项资金要充分考虑老年人体育健身的需求，并加大对经济欠发达地区的支持力度；使用彩票公益金建设的“全民健身工程”要统筹考虑老年人体育健身功能，配置老年人喜爱、适用面广、便捷实用、健身效果显著的体育器材；要充分利用现有公共设施，在公园、广场、绿地及城市空置场所等建设适合老年人体育健身的场地设施，为老年人提供广场舞活动场地，做好电源、夜间照明等基础配套设施，有条件的配置移动音箱等器材设备；要盘活存量资源，改造旧厂房、仓库、老旧商业设施等用于老年人体育健身，对现有公共体育健身场地设施进行无障碍或者适老性改造，有条件的乡镇(街道)综合文化站要建设室外体育健身场地，配备适合老年人开展文体活动的器材和设备；要鼓励、支持企事业单位、社会组织、个人捐赠和赞助，要鼓励政府和社会资本通过PPP模式，积极兴办适合老年人体育健身的场地设施。

(十三)要通过财政补助、政府购买服务等方式，支持公共和民办体育场地设施免费低收费向老年人开放，并不断健全运营管理和服务标准体系，规范服务项目和服务流程，提高服务水平，并按照国家有关规定，争取对适合老年人体育健身的非营利性场地设施减免费用；要整合资源，加强社区公共体育场地设施与社区综合服务设施及社区卫生、文化、养老等社区专项服务设施的功能衔接，提高使用率，发挥综合效益。机关、企事业单位和社会团体内部的体育场地设施要为老年人参加体育健身活动提供便利和服务。公园、广场、绿地等公共场所要为老年人体育健身活动站点和体育健身团队开展活动创造条件。已有的老年人体育健身活动场地设施不得擅自改变用途，并加强管理和维护，确保其功能完好、使用安全，不被侵占、破坏。

五、广泛开展老年人体育健身活动。

(十四)体育部门要支持、指导老年人体育组织利用全民健身日、节假日、纪念日、庆典日，按照“经常自愿、重在参与、就地就近、小型多样、文体结合、科学文明、有益健康”的原则，因时、因人、因地制宜地动员、组织老年人举办社区运动会、家庭运动会、楼群运动会等活动，开展体育表演展示交流，突出参与性、健身性、娱乐性、趣味性和多样性，不断创新活动方式，打造具有地方特色的老年人品牌活动，引导老年人选择一项活动、加入一个团队、享受一种快乐、收获一份健康，推动老年人经常性体育健身活动广泛深入地开展，使老年人体育健身活动常态化。

(十五)要积极为老年人开展体育赛事活动提供服务保障，并通过市场机制引入社会力量承办赛事；要定期举办全国性和区域性老年人体育健身活动，并逐步形成传统和制度，使之成为具有示范性的全民健身活动；要积极引导老年人健康、文明、有序地开展广场舞活动，将广场舞纳入文化、体育部门的重要工作内容，采取划片指导、结对帮扶、公益培训、展演展示等多种方式，探索规范老年人广场舞活动的模式；举办老年人体育活动要坚持“安全第一”和“重在参与、重在健康、重在交流、重在快乐”的原则，有条件的要购买运动伤害类保险，做好人身安全防范工作；体育部门要建立老年人体育健身志愿服务长效化工作机制，结合开展“三关爱”志愿服务活动，广泛组织社会体育指导员、体育科技工作者、体育院校师生、体育运动队等到基层为老年人送服务、送温暖、送健康，并加强对空巢老人、残障老人的体育健身服务。

(十六)要加强老年人体育健身方法的研究和体育健身活动的指导，举办体育健身培训讲座和健身指导咨询等，普及体育健身知识、传授体育健身技能；要不断挖掘整理、普及推广适合老年人特点，简便易行、科学、文明、有效的体育健身方法，根据老年人需求特点创编具有文化艺术内涵、体现科学健身理念、符合群众审美特点的广场舞作品，开展原创作品征集评选，特别是保健娱乐类项目，满足不同年龄、性别、爱好和健康程度老年人体育健身的多样化需要；要引导、支持老年人体育组织培育形成具有民族、民间传统特色的体育健身项目和示范队伍，推动老年人体育健身项目的传承和普及发展。

六、加大对老年人体育工作的经费投入和支持保障。

(十七)体育部门要与发展改革、民政、财政、农业、文化、卫生和计划生育、旅游、老龄、工会、妇联、残联等有关部门加强沟通协调，建立健全并不断完善多部门密切合作、齐抓共管的老年人体育工作体制和充满活力的工作机制。要按照老年人体育工作发展的目标要求，在掌握老年人体育工作发展状况，总结经验的基础上，深入分析存在的问题，问需于老年人，研究制定符合实际的政策措施，积极为老年人参加体育健身活动创造条件，推动老年人体育工作与有关工作融合、互动发展。

(十八)老年人体育工作经费要坚持财政投入为主，社会赞助为辅的原则，实现经费来源多渠道、多元化。县级以上人民政府要把老年人体育工作经费作为全民健身工作经费的组成部分，纳入财政预算，并随着经济的发展逐年增加对老年人体育事业的投入。鼓励、支持机关、企事业单位、社会团体、个人向老年人体育组织赞助和捐赠活动经费。

(十九)要加大对老年人体育工作的宣传力度，充分利用广播、电视、互联网、报纸等各类媒体，开辟老年人体育健身专题、专栏，积极宣传、倡导在全社会形成关注老年人身心健康，有利于老年人参加体育健身的良好氛围，通过树立健康老人典型等手段，宣传体育健身效果，传播“多买健康少买药”、“多去运动，少去医院”、“我运动我健康，我快乐我长寿”的观念，提高老年人体育健身意识，激发老年人参与体育健身的热情；要鼓励、支持形式多样的老年人体育题材文艺创作，推广老年人体育文化，弘扬奥林匹克精神和中华体育精神，践行社会主义核心价值观。

(二十)要鼓励、支持相关行业积极拓展老年人体育健身产品和服务，组织编写出版老年人体育健身丛书等教学制品；要鼓励、支持社会力量提供公益性老年人体育健身服务，引导、支持老年人体育社会组织积极参与老年人体育公共服务的购买；要发展老年人体育健身服务业，提倡、引导老年人健康投资和体育健身消费，大力开发老年人体育健身消费市场；要鼓励、支持体育用品制造企业采用新工艺、新材料、新技术，研发老年人体育健身器材、可穿戴式运动设备、运动健身指导技术装备、运动功能饮料、营养保健食品药品等，不断提高产品的质量和科技含量。

(二十一)要争取各级人民政府把老年人体育工作纳入政府重要议事日程，纳入有关部门的目标责任考核内容；要充分发挥各级全民健身工作委员会的作用，每年专题研究老年人体育工作，提出明确目标任务，指导开展工作；要建立老年人体育工作激励机制，将老年人体育工作成绩显著的单位和个人作为群众体育先进单位和先进个人表彰对象；要根据本意见的要求，结合实际情况，抓紧制定具体实施意见和配套文件，并加强督查落实，总结推广成功经验和做法，保证老年人体育事业持续健康发展。

**关于推进医疗卫生与养老服务
相结合的指导意见**

　　为贯彻落实《国务院关于加快发展养老服务业的若干意见》（国发〔2013〕35号）和《国务院关于促进健康服务业发展的若干意见》（国发〔2013〕40号）等文件要求，进一步推进医疗卫生与养老服务相结合，现提出以下意见。
　　**一、充分认识推进医疗卫生与养老服务相结合的重要性**　　我国是世界上老年人口最多的国家，老龄化速度较快。失能、部分失能老年人口大幅增加，老年人的医疗卫生服务需求和生活照料需求叠加的趋势越来越显著，健康养老服务需求日益强劲，目前有限的医疗卫生和养老服务资源以及彼此相对独立的服务体系远远不能满足老年人的需要，迫切需要为老年人提供医疗卫生与养老相结合的服务。医疗卫生与养老服务相结合，是社会各界普遍关注的重大民生问题，是积极应对人口老龄化的长久之计，是我国经济发展新常态下重要的经济增长点。加快推进医疗卫生与养老服务相结合，有利于满足人民群众日益增长的多层次、多样化健康养老服务需求，有利于扩大内需、拉动消费、增加就业，有利于推动经济持续健康发展和社会和谐稳定，对稳增长、促改革、调结构、惠民生和全面建成小康社会具有重要意义。
　　**二、基本原则和发展目标**　　（一）基本原则。
　　保障基本，统筹发展。把保障老年人基本健康养老需求放在首位，对有需求的失能、部分失能老年人，以机构为依托，做好康复护理服务，着力保障特殊困难老年人的健康养老服务需求；对多数老年人，以社区和居家养老为主，通过医养有机融合，确保人人享有基本健康养老服务。推动普遍性服务和个性化服务协同发展，满足多层次、多样化的健康养老需求。
　　政府引导，市场驱动。发挥政府在制定规划、出台政策、引导投入、规范市场、营造环境等方面的引导作用，统筹各方资源，推动形成互利共赢的发展格局。充分发挥市场在资源配置中的决定性作用，营造平等参与、公平竞争的市场环境，充分调动社会力量的积极性和创造性。
　　深化改革，创新机制。加快政府职能转变，创新服务供给和资金保障方式，积极推进政府购买服务，激发各类服务主体潜力和活力，提高医养结合服务水平和效率。加强部门协作，提升政策引导、服务监管等工作的系统性和协同性，促进行业融合发展。
　　（二）发展目标。
　　到2017年，医养结合政策体系、标准规范和管理制度初步建立，符合需求的专业化医养结合人才培养制度基本形成，建成一批兼具医疗卫生和养老服务资质和能力的医疗卫生机构或养老机构（以下统称医养结合机构），逐步提升基层医疗卫生机构为居家老年人提供上门服务的能力，80%以上的医疗机构开设为老年人提供挂号、就医等便利服务的绿色通道，50%以上的养老机构能够以不同形式为入住老年人提供医疗卫生服务，老年人健康养老服务可及性明显提升。
　　到2020年，符合国情的医养结合体制机制和政策法规体系基本建立，医疗卫生和养老服务资源实现有序共享，覆盖城乡、规模适宜、功能合理、综合连续的医养结合服务网络基本形成，基层医疗卫生机构为居家老年人提供上门服务的能力明显提升。所有医疗机构开设为老年人提供挂号、就医等便利服务的绿色通道，所有养老机构能够以不同形式为入住老年人提供医疗卫生服务，基本适应老年人健康养老服务需求。
　　**三、重点任务**　　（三）建立健全医疗卫生机构与养老机构合作机制。鼓励养老机构与周边的医疗卫生机构开展多种形式的协议合作，建立健全协作机制，本着互利互惠原则，明确双方责任。医疗卫生机构为养老机构开通预约就诊绿色通道，为入住老年人提供医疗巡诊、健康管理、保健咨询、预约就诊、急诊急救、中医养生保健等服务，确保入住老年人能够得到及时有效的医疗救治。养老机构内设的具备条件的医疗机构可作为医院（含中医医院）收治老年人的后期康复护理场所。鼓励二级以上综合医院（含中医医院，下同）与养老机构开展对口支援、合作共建。通过建设医疗养老联合体等多种方式，整合医疗、康复、养老和护理资源，为老年人提供治疗期住院、康复期护理、稳定期生活照料以及临终关怀一体化的健康和养老服务。
　　（四）支持养老机构开展医疗服务。养老机构可根据服务需求和自身能力，按相关规定申请开办老年病医院、康复医院、护理院、中医医院、临终关怀机构等，也可内设医务室或护理站，提高养老机构提供基本医疗服务的能力。养老机构设置的医疗机构要符合国家法律法规和卫生计生行政部门、中医药管理部门的有关规定，符合医疗机构基本标准，并按规定由相关部门实施准入和管理，依法依规开展医疗卫生服务。卫生计生行政部门和中医药管理部门要加大政策规划支持和技术指导力度。养老机构设置的医疗机构，符合条件的可按规定纳入城乡基本医疗保险定点范围。鼓励执业医师到养老机构设置的医疗机构多点执业，支持有相关专业特长的医师及专业人员在养老机构规范开展疾病预防、营养、中医调理养生等非诊疗行为的健康服务。
　　（五）推动医疗卫生服务延伸至社区、家庭。充分依托社区各类服务和信息网络平台，实现基层医疗卫生机构与社区养老服务机构的无缝对接。发挥卫生计生系统服务网络优势，结合基本公共卫生服务的开展为老年人建立健康档案，并为65岁以上老年人提供健康管理服务，到2020年65岁以上老年人健康管理率达到70%以上。鼓励为社区高龄、重病、失能、部分失能以及计划生育特殊家庭等行动不便或确有困难的老年人，提供定期体检、上门巡诊、家庭病床、社区护理、健康管理等基本服务。推进基层医疗卫生机构和医务人员与社区、居家养老结合，与老年人家庭建立签约服务关系，为老年人提供连续性的健康管理服务和医疗服务。提高基层医疗卫生机构为居家老年人提供上门服务的能力，规范为居家老年人提供的医疗和护理服务项目，将符合规定的医疗费用纳入医保支付范围。
　　（六）鼓励社会力量兴办医养结合机构。鼓励社会力量针对老年人健康养老需求，通过市场化运作方式，举办医养结合机构以及老年康复、老年护理等专业医疗机构。在制定医疗卫生和养老相关规划时，要给社会力量举办医养结合机构留出空间。按照“非禁即入”原则，凡符合规划条件和准入资质的，不得以任何理由加以限制。整合审批环节，明确并缩短审批时限，鼓励有条件的地方提供一站式便捷服务。通过特许经营、公建民营、民办公助等模式，支持社会力量举办非营利性医养结合机构。支持企业围绕老年人的预防保健、医疗卫生、康复护理、生活照料、精神慰藉等方面需求，积极开发安全有效的食品药品、康复辅具、日常照护、文化娱乐等老年人用品用具和服务产品。
　　（七）鼓励医疗卫生机构与养老服务融合发展。鼓励地方因地制宜，采取多种形式实现医疗卫生和养老服务融合发展。统筹医疗卫生与养老服务资源布局，重点加强老年病医院、康复医院、护理院、临终关怀机构建设，公立医院资源丰富的地区可积极稳妥地将部分公立医院转为康复、老年护理等接续性医疗机构。提高综合医院为老年患者服务的能力，有条件的二级以上综合医院要开设老年病科，做好老年慢性病防治和康复护理相关工作。提高基层医疗卫生机构康复、护理床位占比，鼓励其根据服务需求增设老年养护、临终关怀病床。全面落实老年医疗服务优待政策，医疗卫生机构要为老年人特别是高龄、重病、失能及部分失能老年人提供挂号、就诊、转诊、取药、收费、综合诊疗等就医便利服务。有条件的医疗卫生机构可以通过多种形式、依法依规开展养老服务。鼓励各级医疗卫生机构和医务工作志愿者定期为老年人开展义诊。充分发挥中医药（含民族医药，下同）的预防保健特色优势，大力开发中医药与养老服务相结合的系列服务产品。
　　**四、保障措施**　　（八）完善投融资和财税价格政策。对符合条件的医养结合机构，按规定落实好相关支持政策。拓宽市场化融资渠道，探索政府和社会资本合作（PPP）的投融资模式。鼓励和引导各类金融机构创新金融产品和服务方式，加大金融对医养结合领域的支持力度。有条件的地方可通过由金融和产业资本共同筹资的健康产业投资基金支持医养结合发展。用于社会福利事业的彩票公益金要适当支持开展医养结合服务。积极推进政府购买基本健康养老服务，逐步扩大购买服务范围，完善购买服务内容，各类经营主体平等参与。
　　（九）加强规划布局和用地保障。各级政府要在土地利用总体规划和城乡规划中统筹考虑医养结合机构发展需要，做好用地规划布局。对非营利性医养结合机构，可采取划拨方式，优先保障用地；对营利性医养结合机构，应当以租赁、出让等有偿方式保障用地，养老机构设置医疗机构，可将在项目中配套建设医疗服务设施相关要求作为土地出让条件，并明确不得分割转让。依法需招标拍卖挂牌出让土地的，应当采取招标拍卖挂牌出让方式。
　　（十）探索建立多层次长期照护保障体系。继续做好老年人照护服务工作。进一步开发包括长期商业护理保险在内的多种老年护理保险产品，鼓励有条件的地方探索建立长期护理保险制度，积极探索多元化的保险筹资模式，保障老年人长期护理服务需求。鼓励老年人投保长期护理保险产品。建立健全长期照护项目内涵、服务标准以及质量评价等行业规范和体制机制，探索建立从居家、社区到专业机构等比较健全的专业照护服务提供体系。
　　落实好将偏瘫肢体综合训练、认知知觉功能康复训练、日常生活能力评定等医疗康复项目纳入基本医疗保障范围的政策，为失能、部分失能老年人治疗性康复提供相应保障。
　　（十一）加强人才队伍建设。做好职称评定、专业技术培训和继续医学教育等方面的制度衔接，对养老机构和医疗卫生机构中的医务人员同等对待。完善薪酬、职称评定等激励机制，鼓励医护人员到医养结合机构执业。建立医疗卫生机构与医养结合机构人员进修轮训机制，促进人才有序流动。将老年医学、康复、护理人才作为急需紧缺人才纳入卫生计生人员培训规划。加强专业技能培训，大力推进养老护理员等职业技能鉴定工作。支持高等院校和中等职业学校增设相关专业课程，加快培养老年医学、康复、护理、营养、心理和社会工作等方面专业人才。
　　（十二）强化信息支撑。积极开展养老服务和社区服务信息惠民试点，利用老年人基本信息档案、电子健康档案、电子病历等，推动社区养老服务信息平台与区域人口健康信息平台对接，整合信息资源，实现信息共享，为开展医养结合服务提供信息和技术支撑。组织医疗机构开展面向养老机构的远程医疗服务。鼓励各地探索基于互联网的医养结合服务新模式，提高服务的便捷性和针对性。
　　**五、组织实施**　　（十三）加强组织领导和部门协同。各地区、各有关部门要高度重视，把推进医养结合工作摆在重要位置，纳入深化医药卫生体制改革和促进养老、健康服务业发展的总体部署，各地要及时制定出台推进医养结合的政策措施、规划制度和具体方案。各相关部门要加强协同配合，落实和完善相关优惠扶持政策，共同支持医养结合发展。发展改革部门要将推动医疗卫生与养老服务相结合纳入国民经济和社会发展规划。卫生计生、民政和发展改革部门要做好养老机构和医疗卫生机构建设的规划衔接，加强在规划和审批等环节的合作，制定完善医养结合机构及为居家老年人提供医疗卫生和养老服务的标准规范并加强监管。财政部门要落实相关投入政策，积极支持医养结合发展。人力资源社会保障、卫生计生部门要将符合条件的医养结合机构纳入城乡基本医疗保险定点范围。国土资源部门要切实保障医养结合机构的土地供应。城乡规划主管部门要统筹规划医养结合机构的用地布局。老龄工作部门要做好入住医养结合机构和接受居家医养服务老年人的合法权益保障工作。中医药管理部门要研究制定中医药相关服务标准规范并加强监管，加强中医药适宜技术和服务产品推广，加强中医药健康养老人才培养，做好中医药健康养老工作。
　　（十四）抓好试点示范。国家选择有条件、有代表性的地区组织开展医养结合试点，规划建设一批特色鲜明、示范性强的医养结合试点项目。各地要结合实际积极探索促进医养结合的有效形式，每个省（区、市）至少设1个省级试点地区，积累经验、逐步推开。卫生计生、民政部门要会同相关部门密切跟踪各地进展，帮助解决试点中的重大问题，及时总结推广好的经验和做法，完善相关政策措施。
　　（十五）加强考核督查。各地区、各有关部门要建立以落实医养结合政策情况、医养结合服务覆盖率、医疗卫生机构和养老机构无缝对接程度、老年人护理服务质量、老年人满意度等为主要指标的考核评估体系，加强绩效考核。卫生计生、民政部门要会同相关部门加强对医养结合工作的督查，定期通报地方工作进展情况，确保各项政策措施落到实处。

**国家人口发展规划（2016—2030年）**

**第三节　完善家庭发展支持体系**

建立完善包括生育支持、幼儿养育、青少年发展、老人赡养、病残照料、善后服务等在内的家庭发展政策。完善税收、抚育、教育、社会保障、住房等政策，减轻生养子女家庭负担。完善计划生育奖励假制度和配偶陪产假制度。鼓励雇主为孕期和哺乳期妇女提供灵活的工作时间安排及必要的便利条件。支持妇女生育后重返工作岗位。增强社区幼儿照料、托老日间照料和居家养老等服务功能。完善殡葬基本公共服务。加强家庭信息采集和管理，为家庭发展政策的制定和实施提供依据。大力发展家庭服务业。加强婚姻家庭辅导，推进新型家庭文化建设，开展幸福家庭创建活动。

加大对计划生育家庭的扶助力度，对全面两孩政策实施前的独生子女家庭和农村计划生育双女家庭，继续实行现行各项奖励扶助政策，在社会保障、集体收益分配、就业创业、新农村建设等方面予以倾斜。完善计划生育家庭特别扶助制度，加大对残疾人家庭、贫困家庭、计划生育特殊家庭、老年空巢家庭、单亲家庭等的帮扶支持力度，充分发挥社会工作服务机构和社会工作者的专业作用。

第四章　增加劳动力有效供给

——注重人口与经济良性互动

综合应对劳动年龄人口总量下降和结构老化趋势，全面提升劳动力质量，挖掘劳动力供给潜能，加强与就业政策和劳动力市场建设的有机衔接，为经济社会发展提供有效人力资本支撑。

| **专栏3　大龄劳动力人力资本开发行动** |
| --- |
| 适应我国老龄化发展趋势，积极借鉴国际有益经验，通过教育培训、健康服务、就业促进等方式鼓励大龄失业人员回归劳动力市场。探索建立养老金长缴多得的激励约束机制；加强大龄劳动力在岗继续教育培训，落实完善职业培训补贴、职业技能鉴定补贴等政策，支持大龄劳动力提升就业技能；加强职业健康服务，提高大龄劳动力健康水平；落实税收优惠、社会保险补贴、创业担保贷款等扶持政策，鼓励各类企业吸纳大龄失业人员就业；加强公共就业服务网络平台建设，为大龄失业人员提供更多个性化职业指导、职业介绍、政策咨询等公共就业服务；结合大龄失业人员特点，提供更多非全职就业、志愿服务和社区工作等岗位。 |

**第三节　积极开发老年人力资源**

充分发挥老年人参与经济社会活动的主观能动性和积极作用。实施渐进式延迟退休年龄政策，逐步完善职工退休年龄政策，有效挖掘开发老年人力资源。大力发展老年教育培训。鼓励专业技术领域人才延长工作年限，积极发挥其在科学研究、学术交流和咨询服务等方面的作用。鼓励老年人积极参与家庭发展、互助养老、社区治理、社会公益等活动，继续发挥余热并实现个人价值。

老年人、妇女、儿童、残疾人和贫困人口，是人口发展中必须特别关注的重点人群。要构建管长远的制度框架，制定有针对性的政策措施，创造条件让重点人群共享发展成果，促进社会和谐与公平正义。

**第一节　积极应对人口老龄化**

针对人口老龄化程度不断加深的趋势，要加强顶层设计，做到及早应对、科学应对、综合应对。坚持持续、健康、参与、公平的原则，加快构建以社会保障、养老服务、健康支持、宜居环境为核心的应对老龄化制度框架，完善以人口政策、人才开发、就业促进、社会参与为支撑的政策体系。建立更加公平可持续的社会保障制度，加快城乡居民全覆盖，逐步提高基本养老和基本医疗保险统筹层次，确保基金安全可持续运行。大力发展企业年金、职业年金、个人储蓄性养老保险和商业医疗保险，在试点基础上推出个人税收递延型养老保险。探索建立长期护理保险制度，开展长期护理保险试点。全面建立针对经济困难高龄、失能老年人的补贴制度，做好与长期护理保险的衔接。加快完善以居家为基础、社区为依托、机构为补充、医养结合的养老服务体系，增加养老服务和产品供给。建设预防、医疗、康复、护理、安宁疗护等相衔接的覆盖全生命周期的医疗服务体系，强化对老年常见病、慢性病的健康指导和综合干预，提升中医保健、体检体测、体育健身等健康管理水平。完善家庭养老支持措施，建设无障碍的老年友好型社区和城市，营造良好社会氛围，形成敬老、养老、助老的社会风尚。

**国务院办公厅关于印发老年教育**

**发展规划（2016—2020年）的通知**

**老年教育发展规划（2016—2020年）**

老年人是国家和社会的宝贵财富。老年教育是我国教育事业和老龄事业的重要组成部分。发展老年教育，是积极应对人口老龄化、实现教育现代化、建设学习型社会的重要举措，是满足老年人多样化学习需求、提升老年人生活品质、促进社会和谐的必然要求。为贯彻落实《中华人民共和国老年人权益保障法》、《国家中长期教育改革和发展规划纲要（2010—2020年）》，促进老年教育事业科学发展，制定本规划。

**一、规划背景**

当前我国已进入老龄化社会，2015年底我国60岁以上老年人口已经达到2.22亿，占总人口的16.1%，预计2020年老年人口将达到2.43亿，未来20年我国人口老龄化形势将更加严峻，“未富先老”的特征日益凸显，对我国社会主义现代化进程产生全面而深远影响，特别是老年人的精神文化和学习需求增长较快，发展老年教育的形势和任务更加紧迫。

世界上较早进入老龄化社会的国家和地区普遍出台终身教育、老年教育领域法律法规，并将老年教育政策作为重要的社会政策。许多国家通过兴办第三年龄大学、推动社区老年人互助学习、倡导老年人利用网络自主学习等多种形式发展老年教育。

党和国家高度重视老龄工作，积极推动老年教育事业发展。目前有700多万老年人在老年大学等机构学习，有上千万老年人通过社区教育、远程教育等各种形式参与学习，初步形成了多部门推动、多形式办学的老年教育发展格局。同时必须清醒地看到，我国老年教育还存在资源供给不足，城乡、区域间发展不平衡，保障机制不够健全，部门协调亟待加强，社会力量参与的深度和广度需进一步拓展等问题。解决这些问题，推动老年教育持续健康发展，是当前和今后一个时期积极应对人口老龄化、大力发展老龄服务事业和产业的迫切任务。

**二、总体要求**

（一）指导思想。全面贯彻党的十八大及十八届三中、四中、五中全会精神和习近平总书记系列重要讲话精神，落实党中央、国务院决策部署，按照“五位一体”总体布局和“四个全面”战略布局，牢固树立和贯彻落实创新、协调、绿色、开放、共享的新发展理念，坚持“党委领导、政府主导、社会参与、全民行动”的老龄工作方针，以扩大老年教育供给为重点，以创新老年教育体制机制为关键，以提高老年人的生命和生活质量为目的，整合社会资源、激发社会活力，提升老年教育现代化水平，让老年人共享改革发展成果，进一步实现老有所教、老有所学、老有所为、老有所乐，努力形成具有中国特色的老年教育发展新格局。

（二）基本原则。

保障权益、机会均等。保障老年人受教育权利，努力让不同年龄层次、文化程度、收入水平、健康状况的老年人均有接受教育的机会。充分利用各种资源，统筹加强组织管理，实现资源共享和协调发展，提高老年教育的可及性，最大限度满足各类老年群体学习需求。

政府主导、市场调节。发挥政府在制定规划、营造环境、加大投入等方面的作用，统筹协调各部门老年教育工作。激发社会活力，继续探索和完善政府购买服务机制，引导社会力量积极参与，带动相关产业发展。

优化布局、面向基层。在办好现有老年教育的基础上，将老年教育的增量重点放在基层和农村，形成以基层需求为导向的老年教育供给结构，优化城乡老年教育布局，促进老年教育与经济社会协调发展。

开放便利、灵活多样。促进各类教育机构开放，运用互联网等科技手段开展老年教育，为全体老年人创造学习条件、提供学习机会、做好学习服务。畅通学习渠道，方便就近学习，办好家门口的老年教育。

因地制宜、特色发展。从区域发展不平衡的实际和多样化的学习需求出发，因地制宜开展老年教育。鼓励结合当地历史、人文资源和民俗民风等特点，推动老年教育特色发展。

（三）主要目标。到2020年，基本形成覆盖广泛、灵活多样、特色鲜明、规范有序的老年教育新格局。老年教育法规制度逐步健全，职责明确、主体多元、平等参与、管办分离的管理体制和运行机制得到完善。老年教育基础能力有较大幅度提升，教育内容不断丰富，形式更加多样。各类老年教育机构服务能力进一步提升，全社会关注支持老年教育、参与举办老年教育的积极性显著提高。以各种形式经常性参与教育活动的老年人占老年人口总数的比例达到20%以上。

**三、主要任务**

（一）扩大老年教育资源供给。

优先发展城乡社区老年教育。完善基层社区老年教育服务体系，整合利用现有的社区教育机构、县级职教中心、乡镇成人文化技术学校等教育资源，以及群众艺术馆、文化馆、体育场、社区文化活动中心（文化活动室）、社区科普学校等，开展老年教育活动。建立健全“县（市、区）—乡镇（街道）—村（居委会）”三级社区老年教育网络，方便老年人就近学习。发展农村社区老年教育，有效整合乡村教育文化资源，以村民喜爱的形式开展适应农村老年人需求的教育活动。加强对农村散居、独居老人的教育服务。推进城乡老年教育对口支援，鼓励发达地区以建立分校或办学点、选送教师、配送学习资源、提供人员培训等方式，为边远地区和农村社区老年教育提供支援。

促进各级各类学校开展老年教育。推动各级各类学校向区域内老年人开放场地、图书馆、设施设备等资源，为他们便利化学习提供支持，积极接收有学习需求的老年人入校学习。探索院校利用自身教育资源举办老年教育（学校）的模式。推动普通高校和职业院校面向老年人提供课程资源，特别是艺术类、医药卫生类、师范类院校和开设有养生保健、文化艺术、信息技术、家政服务、社会工作、医疗护理、园艺花卉、传统工艺等专业的职业院校，应结合学校特色开发老年教育课程，为社区、老年教育机构及养老服务机构等积极提供支持服务，共享课程与教学资源。推动开放大学和广播电视大学举办“老年开放大学”或“网上老年大学”，并延伸至乡镇（街道）、城乡社区，建立老年学习网点。

推动老年大学面向社会办学。部门、行业企业、高校等举办的老年大学要树立新的办学理念，积极创造条件，采取多种形式，提高办学开放度，逐步从服务本单位、本系统离退休职工向服务社会老年人转变。省、市两级老年大学在开展教育教学工作的同时，要在办学模式示范、教学业务指导、课程资源开发等方面对区域内老年教育发挥带动和引领作用，将老年大学集聚的教育资源向基层和社区辐射。加强老年大学与社会教育机构的合作，组建老年教育联盟（集团）。

（二）拓展老年教育发展路径。

丰富老年教育内容和形式。积极开展老年人思想道德、科学文化、养生保健、心理健康、职业技能、法律法规、家庭理财、闲暇生活、代际沟通、生命尊严等方面的教育，帮助老年人提高生活品质，实现人生价值。创新教学方法，将课堂学习和各类文化活动相结合，积极探索体验式学习、远程学习、在线学习等模式，引导开展读书、讲座、参观、展演、游学、志愿服务等多种形式的老年教育活动。鼓励老年人自主学习，支持建立不同类型的学习团队。

探索养教结合新模式。整合利用社区居家养老资源，在社区老年人日间照料中心、托老所等各类社区居家养老场所内，开展形式多样的老年教育。积极探索在老年养护院、城市社会福利院、农村敬老院等养老服务机构中设立固定的学习场所，配备教学设施设备，通过开设课程、举办讲座、展示学习成果等形式，推进养教一体化，推动老年教育融入养老服务体系，丰富住养老人的精神文化生活。关注失能失智及盲聋等特殊老人群体，提供康复教育一体化服务。

积极开发老年人力资源。用好老年人这一宝贵财富，充分发挥老年人的智力优势、经验优势、技能优势，为其参与经济社会活动搭建平台、提供教育支持。发挥老年人在传承中华优秀传统文化、引导全社会特别是青少年培育和践行社会主义核心价值观等方面的积极作用，彰显长者风范。鼓励老年人利用所学所长，在科学普及、环境保护、社区服务、治安维稳等方面积极服务社会、奉献社会。

（三）加强老年教育支持服务。

运用信息技术服务老年教育。加强数字化学习资源跨区域、跨部门共建共享，开展对现有老年教育课程的数字化改造，开发适合老年人远程学习的数字化资源。通过互联网、数字电视等渠道，加强优质老年学习资源对农村、边远、贫困、民族地区的辐射。推动信息技术融入老年教育教学全过程，推进线上线下一体化教学，支持老年人网上学习。运用信息化手段，为老年人提供导学服务、个性化学习推荐等学习支持。

整合文化体育科技资源服务老年教育。推动美术馆、图书馆、文化馆（站、中心）、科技馆、博物馆、纪念馆、公共体育设施、爱国主义示范基地、科普教育基地等向老年人免费开放。鼓励有条件的地区发挥文化、教育、体育、科技等资源优势，结合区域实际，建设不同主题、富有特色的老年教育学习体验基地。充分发挥广播电视、报刊杂志、门户网站等媒体作用，开设贴近老年人生活的专栏专题。

（四）创新老年教育发展机制。

鼓励社会力量参与老年教育。充分激发市场活力，推进举办主体、资金筹措渠道的多元化，通过政府购买服务、项目合作等多种方式，支持和鼓励各类社会力量通过独资、合资、合作等形式举办或参与老年教育。运用市场机制调节供需关系，进一步优化老年教育的市场结构、内容和布局。加强规划指导和外部监管，营造平等参与、公平竞争的市场环境。充分发挥社会组织在老年教育中的作用，鼓励其通过提供师资、开发课程等方式支持开展老年教育。支持老年教育领域社会组织和老年志愿服务团队发展。

促进老年教育与相关产业联动。扩大老年教育消费，发掘与老年教育密切相关的养老服务、旅游、服装服饰、文化等产业价值，促进生活性服务业提档升级，拉动内需，推动投资增长和相关产业发展。

（五）促进老年教育可持续发展。

加强学科建设与人才培养培训。鼓励综合类高校、师范类院校、职业院校开设老年教育相关专业，其他高校也要加强老年教育相关专业建设。支持有条件的高校开展老年教育方向的研究生教育，加快培养老年教育教学、科研和管理人才。鼓励老年教育机构的专任教师和管理人员在职进修老年教育专业课程，攻读相关专业学位。

加强理论与政策研究。依托有关高校、科研院所、老年教育机构等建立若干个老年教育研究基地，开展老年教育基础理论研究、政策研究和应用研究，探讨和解决老年教育发展中的重大理论和实践问题。加强老年教育学术期刊建设，搭建优秀成果共享和推广平台。鼓励社会组织开展老年教育优秀研究成果交流活动。

加强国际交流合作。积极参与有关国际教育组织的活动，加强与国外老年教育机构的交流与合作，借鉴国外老年教育先进理念和做法，宣传推广我国发展老年教育的经验与成果，扩大我国老年教育的国际影响力。

**四、重点推进计划**

（一）社会主义核心价值观培育计划。将培育和践行社会主义核心价值观作为老年教育的重要内容，编写相关读本，设计形式多样的教育活动项目，将社会主义核心价值观融入老年人学习和活动之中。积极推进校园文化建设，培育优良校风、教风、学风，打造一批在培育和践行社会主义核心价值观方面具有示范作用的老年学校、老年学习团队。

（二）老年教育机构基础能力提升计划。整合资源，改善基层社区老年教育机构设施设备，建设一批在本区域发挥示范作用的乡镇（街道）社区老年人学习场所，建设好村（居委会）老年社区学习点。改善现有老年大学办学条件，提升其教学场所和设施的现代化、规范化水平，进一步增强其社会服务能力。到2020年，全国县级以上城市原则上至少应有一所老年大学，50%的乡镇（街道）建有老年学校，30%的行政村（居委会）建有老年学习点。探索“养、医、体、文”等场所与老年人学习场所的结合，推出一批创新老年教育办学模式的典型。各省（区、市）选取若干个养老服务机构，开展养教结合试点。

（三）学习资源建设整合计划。研究制定老年人学习发展指南，为不同年龄层次的老年人提供包括学习规划在内的咨询服务。探索建立老年教育通用课程教学大纲，促进资源建设规范化、多样化。遴选、开发一批通用型老年学习资源，整合一批优秀传统文化、非物质文化遗产、地方特色老年教育资源，推介一批科普知识和健康知识学习资源，引进一批国外优质学习资源，形成系列优质课程推荐目录。定期举办老年学习资源建设交流活动。到2020年，各省（区、市）都应初步建立起支撑区域内老年教育发展的老年学习资源库。

（四）远程老年教育推进计划。探索以开放大学和广播电视大学为主体建设老年开放大学，开发整合远程老年教育多媒体课程资源。支持国家开放大学率先建设在全国发挥示范作用的老年健康艺术教育体验基地。推动有条件的省（区、市）老年大学、开放大学和广播电视大学建设具有地方特色的示范性老年教育体验基地。到2020年，力争全国50%的县（市、区）可通过远程教育开展老年教育工作。

（五）老有所为行动计划。组织引导离退休老干部、老同志讲好中国故事、弘扬中国精神、传播中国好声音。积极搭建服务平台，建立由离退休干部、专业技术人员及其他有所专长的老同志组成的老年教育兼职教师队伍。推动各类老年社会团体与大中小学校合作，发挥老年人在教育引导青少年继承优良传统、培育科学精神等方面的积极作用。广泛开展老年志愿服务活动，到2020年，力争每个老年大学培育1—2支老年志愿者队伍，老年学校普遍建有志愿者服务组织。

**五、保障措施**

（一）加强组织实施。建立健全党委领导、政府统筹，教育、组织、民政、文化、老龄部门密切配合，其他相关部门共同参与的老年教育管理体制。各相关部门要按照职责分工，加强沟通协调，通过规划编制、政策制定、指导监督，共同研究解决老年教育发展中的重大问题。老年教育工作要纳入对各级政府相关部门绩效考评内容。各省（区、市）要把老年教育纳入本地区经济社会发展规划和教育事业发展规划，结合实际，提出落实本规划、加快发展老年教育的具体实施方案和举措，分阶段、分步骤组织实施。对各地区在实施本规划中好的做法和经验，要及时总结推广。

（二）推动法规制度建设。研究完善涉及老年教育的相关制度。支持鼓励有条件的地区通过制定相关地方法规促进老年教育事业规范健康发展。在老龄事业相关政策措施中重视支持发展老年教育。探索开展老年教育发展情况调查统计工作，支持社会组织等第三方开展老年教育发展状况评估和研究。

（三）加强队伍建设。鼓励普通高校、职业院校相关专业毕业生及相关行业优秀人才到老年教育机构工作。各级各类学校要鼓励教师参与老年教育相关工作，并纳入本校工作考核，支持教师到校外老年教育机构兼职任教或从事志愿服务。建立老年教育教师岗位培训制度，支持老年教育机构教师、技术和管理人员的专业发展。专职人员在薪酬福利、业务进修、职务（职称）评聘、绩效考核等方面享有同类学校工作人员的同等权利和待遇。鼓励专业社工等参与从事老年教育工作。建立老年教育师资库。加快培养一支结构合理、数量充足、素质优良，以专职人员为骨干、与兼职人员和志愿者相结合的教学和管理队伍。

（四）完善经费投入机制。各地区要采取多种方式努力增加对老年教育的投入，切实拓宽老年教育经费投入渠道，形成政府、市场、社会组织和学习者等多主体分担和筹措老年教育经费的机制。老年教育经费应主要用于老年教育公共服务。鼓励和支持行业企业、社会组织和个人设立老年教育发展基金，企业和个人对老年教育的公益性捐赠支出按照税收法律法规规定享受所得税税前扣除政策。

（五）营造良好氛围。各地区各部门要广泛宣传党和国家关于发展老年教育的方针政策，广泛宣传老年教育发展中的典型经验、案例、做法和成效，努力使全社会关心、支持和参与老年教育的氛围更加浓厚。要充分调动老年人参与学习的积极性和主动性，积极培育老年学习文化，使学习风尚融入老年人生活，使老年教育成为增进老年人福祉的重要内容。

**国务院办公厅关于全面放开养老服务市场提升养老服务质量的若干意见**

各省、自治区、直辖市人民政府，国务院各部委、各直属机构：
　　养老服务业既是涉及亿万群众福祉的民生事业，也是具有巨大发展潜力的朝阳产业。近年来，我国养老服务业快速发展，产业规模不断扩大，服务体系逐步完善，但仍面临供给结构不尽合理、市场潜力未充分释放、服务质量有待提高等问题。随着人口老龄化程度不断加深和人民生活水平逐步提高，老年群体多层次、多样化的服务需求持续增长，对扩大养老服务有效供给提出了更高要求。为促进养老服务业更好更快发展，经国务院同意，现提出如下意见：
　　一、总体要求
　　（一）指导思想。
　　全面贯彻党的十八大和十八届三中、四中、五中、六中全会精神，深入学习贯彻习近平总书记系列重要讲话精神和治国理政新理念新思想新战略，认真落实党中央、国务院决策部署，紧紧围绕“五位一体”总体布局和“四个全面”战略布局，坚持以新发展理念引领经济发展新常态，坚持中国特色卫生与健康发展道路，持续深化简政放权、放管结合、优化服务改革，积极应对人口老龄化，培育健康养老意识，加快推进养老服务业供给侧结构性改革，保障基本需求，繁荣养老市场，提升服务质量，让广大老年群体享受优质养老服务，切实增强人民群众获得感。
　　（二）基本原则。
　　深化改革，放开市场。进一步降低准入门槛，营造公平竞争环境，积极引导社会资本进入养老服务业，推动公办养老机构改革，充分激发各类市场主体活力。
　　改善结构，突出重点。补齐短板，将养老资源向居家社区服务倾斜，向农村倾斜，向失能、半失能老年人倾斜。进一步扩大护理型服务资源，大力培育发展小型化、连锁化、专业化服务机构。
　　鼓励创新，提质增效。树立健康养老理念，注重管理创新、产品创新和品牌创新，积极运用新技术，培育发展新业态，促进老年产品用品丰富多样、养老服务方便可及。
　　强化监管，优化环境。完善监督机制，健全评估制度，推动行业标准化和行业信用建设，加强行业自律，促进规范发展，维护老年人合法权益。
　　（三）发展目标。
　　到2020年，养老服务市场全面放开，养老服务和产品有效供给能力大幅提升，供给结构更加合理，养老服务政策法规体系、行业质量标准体系进一步完善，信用体系基本建立，市场监管机制有效运行，服务质量明显改善，群众满意度显著提高，养老服务业成为促进经济社会发展的新动能。 　　二、全面放开养老服务市场
　　（四）进一步放宽准入条件。
　　降低准入门槛。设立营利性养老机构，应按“先照后证”的简化程序执行，在工商行政管理部门办理登记后，在辖区县级以上人民政府民政部门申请设立许可。在民政部门登记的非营利性养老机构，可以依法在其登记管理机关管辖范围内设立多个不具备法人资格的服务网点。非本地投资者举办养老服务项目与当地投资者享受同等政策待遇，当地不得以任何名目对此加以限制。
　　放宽外资准入。在鼓励境外投资者在华举办营利性养老机构的基础上，进一步放开市场，鼓励境外投资者设立非营利性养老机构，其设立的非营利性养老机构与境内投资者设立的非营利性养老机构享受同等优惠政策。
　　精简行政审批环节。全面清理、取消申办养老机构的不合理前置审批事项，优化审批程序，简化审批流程。申请设立养老服务类社会组织，符合直接登记条件的可以直接向民政部门依法申请登记，不再经由业务主管单位审查同意。支持新兴养老业态发展，对于养老机构以外的其他提供养老服务的主体，鼓励其依法办理法人登记并享受相关优惠政策。
　　（五）优化市场环境。
　　进一步改进政府服务。举办养老机构审批过程中涉及的各有关部门，都要主动公开审批程序和审批时限，推进行政审批标准化，加强对筹建养老机构的指导服务。加快推行养老机构申办一站式服务，建立“一门受理、一并办理”的网上并联审批平台，进一步提高审批效率。根据[消防法](javascript:SLC(109890))和有关规定，制定既保障安全、又方便合理的养老机构设立和管理配套办法。
　　完善价格形成机制。加快建立以市场形成价格为主的养老机构服务收费管理机制。对于民办营利性养老机构，服务收费项目和标准由经营者自主确定。对于民办非营利性养老机构，服务收费标准由经营者合理确定，有关部门对其财务收支状况、收费项目和调价频次进行必要监管，同时加强对价格水平的监测分析。对于政府运营的养老机构，以扣除政府投入、社会捐赠后的实际服务成本为依据，按照非营利原则，实行政府定价或政府指导价；对于以公建民营等方式运营的养老机构，采用招投标、委托运营等竞争性方式确定运营方，具体服务收费标准由运营方依据委托协议等合理确定。
　　加快公办养老机构改革。各地要因地制宜设置改革过渡期，加快推进具备向社会提供养老服务条件的公办养老机构转制成为企业或开展公建民营，到2020年政府运营的养老床位数占当地养老床位总数的比例应不超过50%。鼓励社会力量通过独资、合资、合作、联营、参股、租赁等方式，参与公办养老机构改革。完善公建民营[养老机构管理办法](javascript:SLC(205904))，政府投资建设和购置的养老设施、新建居民区按规定配建并移交给民政部门的养老设施、国有单位培训疗养机构等改建的养老设施，均可实施公建民营。改革公办养老机构运营方式，鼓励实行服务外包。
　　加强行业信用建设。建立覆盖养老服务行业法人、从业人员和服务对象的行业信用体系。建立健全信用信息记录和归集机制，加强与全国信用信息共享平台的信息交换和共享，通过企业信用信息公示系统向社会公示相关企业的行政许可、行政处罚等信息。引入第三方征信机构，参与养老行业信用建设和信用监管。建立多部门、跨地区的联合奖惩机制，将信用信息作为各项支持政策的重要衡量因素，对诚实守信者在政府购买服务、债券发行等方面实行优先办理、简化程序等绿色通道支持激励政策，建立养老服务行业黑名单制度和市场退出机制，加强行业自律和监管。 　　三、大力提升居家社区养老生活品质
　　（六）推进居家社区养老服务全覆盖。
　　开展老年人养老需求评估，加快建设社区综合服务信息平台，对接供求信息，提供助餐、助洁、助行、助浴、助医等上门服务，提升居家养老服务覆盖率和服务水平。依托社区服务中心（站）、社区日间照料中心、卫生服务中心等资源，为老年人提供健康、文化、体育、法律援助等服务。鼓励建设小型社区养老院，满足老年人就近养老需求，方便亲属照护探视。
　　（七）提升农村养老服务能力和水平。
　　依托农村社区综合服务设施，拓展养老服务功能。鼓励各地建设农村幸福院等自助式、互助式养老服务设施，加强与农村危房改造等涉农基本住房保障政策的衔接。农村集体经济、农村土地流转等收益分配应充分考虑解决本村老年人的养老问题。加强农村敬老院建设和改造，推动服务设施达标，满足农村特困人员集中供养需求，为农村低收入老年人和失能、半失能老年人提供便捷可及的养老服务。鼓励专业社会工作者、社区工作者、志愿服务者加强对农村留守、困难、鳏寡、独居老年人的关爱保护和心理疏导、咨询等服务。充分依托农村基层党组织、自治组织和社会组织等，开展基层联络人登记，建立应急处置和评估帮扶机制，关注老年人的心理、安全等问题。
　　（八）提高老年人生活便捷化水平。
　　通过政府补贴、产业引导和业主众筹等方式，加快推进老旧居住小区和老年人家庭的无障碍改造，重点做好居住区缘石坡道、轮椅坡道、公共出入口、走道、楼梯、电梯候梯厅及轿厢等设施和部位的无障碍改造，优先安排贫困、高龄、失能等老年人家庭设施改造，组织开展多层老旧住宅电梯加装。支持开发老年宜居住宅和代际亲情住宅。各地在推进易地扶贫搬迁以及城镇棚户区、城乡危房改造和配套基础设施建设等保障性安居工程中，要统筹考虑适老化设施配套建设。 　　四、全力建设优质养老服务供给体系
　　（九）推进“互联网＋”养老服务创新。
　　发展智慧养老服务新业态，开发和运用智能硬件，推动移动互联网、云计算、物联网、大数据等与养老服务业结合，创新居家养老服务模式，重点推进老年人健康管理、紧急救援、精神慰藉、服务预约、物品代购等服务，开发更加多元、精准的私人订制服务。支持适合老年人的智能化产品、健康监测可穿戴设备、健康养老移动应用软件（APP）等设计开发。打通养老服务信息共享渠道，推进社区综合服务信息平台与户籍、医疗、社会保障等信息资源对接，促进养老服务公共信息资源向各类养老服务机构开放。
　　（十）建立医养结合绿色通道。
　　建立医疗卫生机构设置审批绿色通道，支持养老机构开办老年病院、康复院、医务室等医疗卫生机构，将符合条件的养老机构内设医疗卫生机构按规定纳入城乡基本医疗保险定点范围。鼓励符合条件的执业医师到养老机构、社区老年照料机构内设的医疗卫生机构多点执业。开通预约就诊绿色通道，推进养老服务机构、社区老年照料机构与医疗机构对接，为老年人提供便捷医疗服务。提升医保经办服务能力，切实解决老年人异地就医直接结算问题。探索建立长期护理保险制度，形成多元化的保险筹资模式，推动解决失能人员基本生活照料和相关医疗护理等所需费用问题。
　　（十一）促进老年产品用品升级。
　　支持企业利用新技术、新工艺、新材料和新装备开发为老年人服务的产品用品，研发老年人乐于接受和方便使用的智能科技产品，丰富产品品种，提高产品安全性、可靠性和实用性；上述企业经认定为高新技术企业的，按规定享受企业所得税优惠。及时更新康复辅助器具配置目录，重点支持自主研发和生产康复辅助器具。
　　（十二）发展适老金融服务。
　　规范和引导商业银行、保险公司等金融机构开发适合老年人的理财、保险产品，满足老年人金融服务需求，鼓励金融机构建设老年人无障碍设施，开辟服务绿色通道。强化老年人金融安全意识，加大金融消费权益保护力度。稳步推进养老金管理公司试点，按照国家有关规定，积极参与养老金管理相关业务，做好相关受托管理、投资管理和账户管理等服务工作。 　　五、切实增强政策保障能力
　　（十三）加强统筹规划。
　　发挥规划引领作用，分级制定养老服务相关规划，与城乡规划、土地利用总体规划、城镇化规划、区域规划等相衔接，系统提升服务能力和水平。各地要进一步扩大面向居家社区、农村、失能半失能老年人的服务资源，结合实际提出养老床位结构的合理比例，到2020年护理型床位占当地养老床位总数的比例应不低于30%。
　　（十四）完善土地支持政策。
　　统筹利用闲置资源发展养老服务，有关部门应按程序依据规划调整其土地使用性质。营利性养老服务机构利用存量建设用地建设养老设施，涉及划拨建设用地使用权出让（租赁）或转让的，在原土地用途符合规划的前提下，允许补缴土地出让金（租金），办理协议出让或租赁手续。企事业单位、个人对城镇现有空闲的厂房、学校、社区用房等进行改造和利用，举办养老服务机构，经有关部门批准临时改变建筑使用功能从事非营利性养老服务且连续经营一年以上的，五年内土地使用性质可暂不作变更。民间资本举办的非营利性养老机构与政府举办的养老机构可依法使用农民集体所有的土地。对在养老服务领域采取政府和社会资本合作（PPP）方式的项目，可以国有建设用地使用权作价出资或者入股建设。
　　（十五）提升养老服务人才素质。
　　将养老护理员培训作为职业培训和促进就业的重要内容。对参加养老服务技能培训或创业培训且培训合格的劳动者，按规定给予培训补贴。推动普通高校和职业院校开发养老服务和老年教育课程，为社区、老年教育机构及养老服务机构等提供教学资源及服务。完善职业技能等级与养老服务人员薪酬待遇挂钩机制。建立养老服务行业从业人员奖惩机制，提升养老护理队伍职业道德素养。将养老护理员纳入企业新型学徒制试点和城市积分入户政策范围。积极开发老年人力资源，为老年人的家庭成员提供养老服务培训，倡导“互助养老”模式。
　　（十六）完善财政支持和投融资政策。
　　完善财政支持政策。各地要建立健全针对经济困难的高龄、失能老年人的补贴制度，统一设计、分类施补，提高补贴政策的精准度。对养老机构的运行补贴应根据接收失能老年人等情况合理发放。各级政府要加大投入，支持养老服务设施建设，切实落实养老机构相关税费优惠政策，落实彩票公益金支持养老服务体系建设政策要求。鼓励各地向符合条件的各类养老机构购买服务。
　　拓宽投融资渠道。鼓励社会资本采取建立基金、发行企业债券等方式筹集资金，用于建设养老设施、购置设备和收购改造社会闲置资源等。鼓励银行业金融机构以养老服务机构有偿取得的土地使用权、产权明晰的房产等固定资产和应收账款、动产、知识产权、股权等抵质押，提供信贷支持，满足养老服务机构多样化融资需求。有条件的地方在风险可控、不改变养老机构性质和用途的前提下，可探索养老服务机构其他资产抵押贷款的可行模式。 　　六、加强监管和组织实施
　　（十七）加强服务监管。
　　各地要建立健全民政部门和相关部门协同配合的监管机制，加强对养老机构运营和服务的监管。严禁以举办养老机构名义从事房地产开发，严禁利用养老机构的房屋、场地、设施开展与养老服务无关的活动，严禁改变机构的养老服务性质。做好养老服务领域非法集资信息监测和分析工作，做好政策宣传和风险提示工作。对养老服务中虐老欺老等行为，对养老机构在收取保证金、办理会员卡和发行金融产品等活动中的违法违规行为，要依法严厉查处。加强养老设施和服务安全管理，建立定期检查机制，确保老年人人身安全。
　　（十八）加强行业自律。
　　民政、质检等部门要进一步完善养老服务标准体系，抓紧制定管理和服务标准。落实养老机构综合评估和报告制度，开展第三方评估并向社会公布，评估结果应与政府购买服务、发放建设运营补贴等挂钩。政府运营的养老机构要实行老年人入住评估制度，综合评估申请入住老年人的情况，优先保障特困人员集中供养需求和其他经济困难的孤寡、失能、高龄等老年人的服务需求。
　　（十九）加强宣传引导。
　　坚持以社会主义核心价值观为引领，弘扬中华民族尊老、敬老的社会风尚和传统美德，开展孝敬教育，营造养老、助老的良好社会氛围，加强对养老服务业发展过程中涌现出的先进典型和先进事迹的宣传报道，及时总结推广养老服务业综合改革试点中的好经验、好做法。依法打击虐待、伤害老年人及侵害老年人合法权益的行为。积极组织开展适合老年人的文化体育娱乐活动，引导老年人积极参与社区服务、公益活动和健康知识培训，丰富老年人精神文化生活。
　　（二十）加强督促落实。
　　各地要把全面放开养老服务市场、提升养老服务质量摆在重要位置，建立组织实施机制，及时制定配套实施意见，对政策落实情况进行跟踪分析和监督检查，确保责任到位、工作到位、见到实效。各部门要加强协同配合，落实和完善相关优惠政策，共同促进养老服务提质增效。对不落实养老服务政策，或者在养老机构运营和服务中有违反法律法规行为的，依法依规追究相关人员的责任。国家发展改革委、民政部要会同有关部门加强对地方的指导，及时督促检查并报告工作进展情况。

**关于推进老年宜居环境建设的指导意见**

   为改善老年人生活环境，提升老年人生活生命质量，增强老年人幸福感、获得感，根据《中华人民共和国老年人权益保障法》，现就加强老年宜居环境建设，提出如下指导意见。

**一、重要意义**

   近年来，各地区、各有关部门在推进老年宜居环境建设，改善老年人居住、生活和社会文化环境等方面进行了积极探索，取得了明显成效，但在老年人居住、出行、就医、养老以及社会参与等方面依然存在着不适老、不宜居的问题。随着我国人口老龄化的快速发展和新型城镇化进程的不断加快，公共基础设施与老龄社会要求之间不适应的矛盾将日益凸显。推进老年宜居环境建设有利于增进老年民生福祉，有利于促进经济发展、增进社会和谐，有利于有效应对人口老龄化挑战，是开展积极应对人口老龄化行动的重要举措，也是扩大内需、拉动消费、促进经济增长的重要措施，对推动老龄事业全面协调可持续发展具有重要意义。

**二、基本原则和发展目标**

   （一）基本原则

   ——理念引领，规划先行。在经济社会发展中，要综合考虑人口老龄化的影响，树立适老宜居新理念。将老年宜居环境建设纳入国民经济和社会发展规划、城乡规划及相关专项规划，加强前瞻性规划和安排，以规划带动老年宜居环境建设工作的全面开展。
   ——城乡统筹，突出重点。统筹兼顾，全面推进，促进城乡老年宜居环境建设协调发展。树立问题导向，聚焦城乡社区老年宜居环境建设的重点领域和薄弱环节，集合运用保障民生的各方面资源，创新供给方式，提升资源使用效率，优先解决老年人生活环境中存在的突出问题。
   ——多元参与，共建共享。引导市场、社会、家庭、个人多元参与，形成合力，发挥财政资金撬动功能，创新公共基础设施投融资体制，推广政府和社会资本合作模式。弘扬孝亲美德，塑造敬老风尚，促进代际和谐，使人人既是老年宜居环境建设工作的参与者，又是建设成果的受益者。
   ——改革创新，注重实效。既要加强顶层设计，又要尊重群众首创精神，积极推进老年宜居环境建设的理论创新、实践创新和制度创新。鼓励各地立足实际，创新实现方式，建立长效机制，形成地方特色。

   （二）发展目标

   到2025年，安全、便利、舒适的老年宜居环境体系基本建立，“住、行、医、养”等环境更加优化，敬老养老助老社会风尚更加浓厚。
   ——老年宜居环境理念普遍树立，老年群体的特性和需求得到充分考虑，形成人人关注、全民参与老年宜居环境建设的良好社会氛围。
   ——老年人保持健康、活力、独立的软硬件环境不断优化，适宜老年人的居住环境、安全保障、社区支持、家庭氛围、人文环境持续改善。
   ——老年人融入社会、参与社会的障碍不断消除，老年人信息交流、尊重与包容、自我价值实现的有利环境逐渐形成。
   ——各地普遍开展老年宜居环境建设工作，形成一批各具特色的老年友好城市、老年宜居社区。

**三、重点任务**

   根据现阶段老年人在日常生活和社会参与等方面存在的不适老、不宜居的问题，今后一个时期老年宜居环境建设的重点任务是建设适老居住、出行、就医、养老等的物质环境和包容、支持老年人融入社会的文化环境。

   （一）适老居住环境

   1.推进老年人住宅适老化改造。建立社区防火和紧急救援网络，完善老年人住宅防火和紧急救援救助功能，鼓励发展老年人紧急呼叫产品与服务，鼓励安装独立式感烟火灾探测报警器等设施设备。对老年人住宅室内设施中存在的安全隐患进行排查和改造，有条件的地方可对于特困老年人家庭的改造给予适当补助。引导老年人家庭对日常生活设施进行适老化改造。
   2.支持适老住宅建设。在城镇住房供应政策中，对开发老年公寓、老少同居的新社区和有适老功能的新型住宅提供相应政策扶持。鼓励发展通用住宅，注重住宅的通用性，满足各年龄段家庭成员，尤其是老年人对居住环境的必要需求。在推进老（旧）居住（小）区、棚户区、农村危房改造中，将符合条件的老年人优先纳入住房保障范围。加大对住宅小区消防安全保障设施建设力度，完善公共消防基础设施建设。

   （二）适老出行环境

  3.强化住区无障碍通行。加强老年人住宅公共设施无障碍改造，重点对坡道、楼梯、电梯、扶手等公共建筑节点进行改造，满足老年人基本的安全通行需求。加强对《无障碍环境建设条例》的执法监督检查，新建住宅应严格执行无障碍设施建设相关标准，规范建设无障碍设施。
   4.构建社区步行路网。遵循安全便利原则，加强社区路网设施规划与建设，加强对社区道路系统、休憩设施、标识系统的综合性无障碍改造。清除步行道路障碍物，保持小区步行道路平整安全,严禁非法占用小区步行道。
   5.发展适老公共交通。加强城市道路、公共交通建筑、公共交通工具的无障碍建设与改造。继续落实老年人乘车优惠政策，不断扩大优惠覆盖范围和优惠力度，改善老年人乘车环境，按规定设置“老幼病残孕”专座，鼓励老年人错峰出行。完善公共交通标志标线，强化对老年人的安全提醒，重点对大型交叉路口的安全岛、隔离带及信号灯进行适老化改造。
   6.完善老年友好交通服务。有条件的地区，要在机场、火车站、汽车站、港口码头、旅游景区等人流密集场所为老年人设立等候区域和绿色通道，加大对老年人的服务力度，提供志愿服务，方便老年人出行。乘务和服务人员应为老年人提供礼貌友好服务。

   （三）适老健康支持环境

   7.优化老年人就医环境。加强老年病医院、护理院、老年康复医院和综合医院老年科建设，推进基层老年医疗卫生服务网点建设，积极推进乡镇卫生院和村卫生室一体化管理，为老年人提供便利的就医环境。推进基层医疗卫生机构和医务人员参与社区、居家养老，与老年人家庭建立签约服务关系，为老年人提供连续性的社区健康支持环境。鼓励医疗卫生机构与养老机构开展对口支援、合作共建，支持养老机构开展医疗服务，为入住老年人提供无缝对接的医疗服务环境。
   8.提升老年健康服务科技水平。开展智慧家庭健康养老示范应用，鼓励发挥地方积极性开展试点，调动各级医疗资源、基层组织以及相关养老服务机构、产业企业等方面力量，开展健康养老服务。研究制定鼓励性政策引导产业发展，鼓励运用云计算、大数据等技术搭建社区、家庭健康服务平台，提供实时监测、长期跟踪、健康指导、评估咨询等老年人健康管理服务。发展血糖、心率、脉搏监测等生物医学传感类可穿戴设备，开发适用于基层医疗卫生机构和社区家庭的各类诊疗终端和康复治疗设备。

   （四）适老生活服务环境

   9.加快配套设施规划建设。在市政建设中，统筹考虑，统一规划，同步建设涉老公共服务设施，增强老年人生活的便利性。鼓励综合利用城乡社区中存量房产、设施、土地服务老年人，优化老年人居家养老的社区支持环境，养老机构、日间照料中心、老年人就餐点、老年人活动中心等各类生活服务设施，与社区相关配套设施集约建设、资源共享。
   10.加强公共设施无障碍改造。按照无障碍设施工程建设相关标准和规范，加强对银行、商场、超市、便民网点、图书馆、影剧院、博物馆、公园、景区等与老年人日常生活密切相关的公共设施的无障碍设计与改造。鼓励公共场所提供老花镜、放大镜等方便老年人阅读的物品，有条件的可配备大字触屏读报系统，使公共设施更适合老年人使用。
   11.健全社区生活服务网络。扶持专业化居家养老服务组织，不断开发服务产品、提高服务质量。广泛发展睦邻互助养老服务。依托社区自治组织，发挥物业管理企业及驻区单位的积极作用，向有需求的老年人提供基本生活照料等多种服务。发挥各类志愿服务组织的积极作用，引导社会各界开展多种形式的助老惠老志愿服务活动。
   12.构建适老信息交流环境。进行信息无障碍改造，提升互联网网站等通信设施服务老年群体的能力和水平, 全面促进和改善信息无障碍服务环境，消除老年人获取信息的障碍，缩小“数字鸿沟”。
   13.加强老年用品供给。着力开发老年用品市场，重点设计和研发老年人迫切需求的食品、医药用品、日用品、康复护理、服饰、辅助生活器具、老年科技文化产品。推进适宜老年人特点的通用产品及实用技术的研发和推广。严格老年用品规范标准，加强监督管理。
   14.大力发展老年教育。结合多层次养老服务体系建设，改善基层社区老年人的学习环境，完善老年人社区学习网络。建设一批在本区域发挥示范作用的乡镇（街道）老年人学习场所和老年大学，努力提高老年教育的参与率和满意度。

   （五）敬老社会文化环境

   15.营造老年社会参与支持环境。树立积极老龄观，倡导老年人自尊自立自强，鼓励老年人自愿量力、依法依规参与经济社会发展，改善自身生活，实现自我价值。以积极的态度看待老年人，破解制约老年人参与经济社会发展的法规政策束缚和思想观念障碍，积极拓展老年人力资源开发的渠道，为广大老年人在更大程度、更宽领域参与经济社会发展搭建平台，提供便利。
   16.弘扬敬老、养老、助老社会风尚。全社会积极开展应对人口老龄化行动，弘扬敬老、养老、助老社会风尚。开展“敬老养老助老”主题教育活动，弘扬中华民族孝亲敬老传统美德。开展老龄法律法规普法宣传教育，增强全社会依法保护老年人合法权益的意识，反对和打击对老年人采取任何形式的歧视、侮辱、虐待、遗弃和家庭暴力，引导律师、公证、基层法律服务所和法律援助机构深入开展老年人法律服务和法律援助工作。
   17.倡导代际和谐社会文化。巩固经济供养、生活照料和精神慰藉的家庭养老功能，完善家庭支持政策。加强家庭美德教育，开展寻找“最美家庭”活动和“好家风好家训”宣传展示活动。引导全社会增强接纳、尊重、帮助老年人的关爱意识，增强不同代际间的文化融合和社会认同，统筹解决各年龄群体的责任分担、利益调处、资源共享等问题，实现家庭和睦、代际和顺、社会和谐，为老年人创造良好的生活氛围。

**四、保障措施**

   （一）加强组织领导。老年宜居环境建设是一项跨领域、跨部门的战略性系统工程。加强老年宜居环境建设，既关乎当前，又关乎长远。各地区、各有关部门要充分认识推进老年宜居环境建设的重要意义，加强组织领导，健全工作机制，强化部门协同，制定具体的实施方案，确立基本目标和主要任务，明确责任，切实抓好落实。
   （二）加强规划统筹。充分考虑人口老龄化发展因素，根据人口老龄化发展趋势、老年人口分布和老年人的特点，在制定城乡规划中综合考虑适合老年人的公共基础、公共安全、生活服务、养老服务、医疗卫生、教育服务、文化体育等设施建设，提高规划编制的科学性、前瞻性、适老性。
   （三）加强政策支持。各地区、各有关部门要运用更加灵活务实的财政政策，依法落实税收政策，统筹政府资金、社会资本、集体收入及产业基金等，鼓励社会资本参与老年宜居环境建设。鼓励金融机构面向老年宜居环境重点工程开发相关金融产品和服务。对免费或优惠向老年人开放的公共服务设施，按照有关规定给予财政补贴。加大养老用地政策落实力度，支持老年宜居环境建设。
   （四）加强示范引导。组织开展老年友好城市、老年宜居社区示范活动。鼓励有条件的地方结合本地实际，选择不同类型的城市（社区），积极稳妥地开展老年宜居环境建设示范工作。示范地区要制定具体的实施方案，明确工作分工，落实工作责任，合理配置资源，加大财力保障，营造良好政策环境，积极推进建设工作的落实。中央和国家机关有关部门要加强对地方老年宜居环境建设示范工作的指导，及时制定完善相关配套政策。条件成熟的示范城市，可纳入全球老年友好型城市网络平台。
   （五）加强宣传推广。要组织新闻媒体，加大宣传工作力度，宣传老年宜居环境建设的重要意义，宣传老年宜居环境建设的新理念，宣传老年宜居环境建设的优秀典型和先进经验，使老年宜居环境建设理念深入人心。积极利用全球老年友好型城市网络等平台，拓展与其他国家和相关国际组织的交流，开展老年友好型城市、老年宜居社区建设等多领域、多形式的交流合作。

**国务院关于印发“十三五”国家老龄事业发展和**

**养老体系建设规划的通知**
国发〔2017〕13号

**“十三五”国家老龄事业发展和**

**养老体系建设规划**

为积极开展应对人口老龄化行动，推动老龄事业全面协调可持续发展，健全养老体系，根据《中华人民共和国老年人权益保障法》和《中华人民共和国国民经济和社会发展第十三个五年规划纲要》，制定本规划。

第一章　规划背景

**第一节　“十二五”时期的成就**

“十二五”时期我国老龄事业和养老体系建设取得长足发展。《中国老龄事业发展“十二五”规划》、《社会养老服务体系建设规划（2011—2015年）》确定的目标任务基本完成。老年人权益保障和养老服务业发展等方面的法规政策不断完善；基本养老、基本医疗保障覆盖面不断扩大，保障水平逐年提高；以居家为基础、社区为依托、机构为补充、医养相结合的养老服务体系初步形成，养老床位数量达到672.7万张；老年宜居环境建设持续推进，老年人社会参与条件继续优化；老年文化、体育、教育事业快速发展，老年人精神文化生活日益丰富；老年人优待项目更加丰富、范围大幅拓宽，敬老养老助老社会氛围日益浓厚，老年人的获得感和幸福感明显增强。

**第二节　“十三五”时期的形势**

“十三五”时期是我国全面建成小康社会决胜阶段，也是我国老龄事业改革发展和养老体系建设的重要战略窗口期。

严峻形势。预计到2020年，全国60岁以上老年人口将增加到2.55亿人左右，占总人口比重提升到17.8%左右；高龄老年人将增加到2900万人左右，独居和空巢老年人将增加到1.18亿人左右，老年抚养比将提高到28%左右；用于老年人的社会保障支出将持续增长；农村实际居住人口老龄化程度可能进一步加深。

明显短板。涉老法规政策系统性、协调性、针对性、可操作性有待增强；城乡、区域老龄事业发展和养老体系建设不均衡问题突出；养老服务有效供给不足，质量效益不高，人才队伍短缺；老年用品市场供需矛盾比较突出；老龄工作体制机制不健全，社会参与不充分，基层基础比较薄弱。

有利条件。党中央、国务院高度重视老龄事业发展和养老体系建设，“十三五”规划纲要对积极应对人口老龄化提出明确要求。经济社会平稳健康发展，供给侧结构性改革加快推进，公共服务和民生保障能力不断增强，科技创新成果加快推广应用，劳动年龄人口仍较为充足，社会参与老龄事业发展积极性不断提高。

制定实施“十三五”国家老龄事业发展和养老体系建设规划是贯彻落实党中央、国务院关于积极应对人口老龄化决策部署的重要措施，对于保障和改善民生，增强老年人参与感、获得感和幸福感，实现全面建成小康社会奋斗目标具有重要战略意义。

第二章　指导思想、基本原则和发展目标

**第一节　指导思想**

高举中国特色社会主义伟大旗帜，全面贯彻党的十八大和十八届三中、四中、五中、六中全会精神，深入贯彻习近平总书记系列重要讲话精神和治国理政新理念新思想新战略，认真落实党中央、国务院决策部署，统筹推进“五位一体”总体布局和协调推进“四个全面”战略布局，牢固树立和贯彻落实创新、协调、绿色、开放、共享的发展理念，坚持党委领导、政府主导、社会参与、全民行动，着力加强全社会积极应对人口老龄化的各方面工作，着力完善老龄政策制度，着力加强老年人民生保障和服务供给，着力发挥老年人积极作用，着力改善老龄事业发展和养老体系建设支撑条件，确保全体老年人共享全面建成小康社会新成果。

**第二节　基本原则**

以人为本，共建共享。坚持保障和改善老年人民生，逐步增进老年人福祉，大力弘扬孝亲敬老、养老助老优秀传统文化，为老年人参与社会发展、社会力量参与老龄事业发展和养老体系建设提供更多更好支持，实现不分年龄、人人共建共享。

补齐短板，提质增效。坚持问题导向，注重质量效益，着力保基本、兜底线、补短板、调结构，不断健全完善社会保障制度体系，促进资源合理优化配置，强化薄弱环节，加大投入力度，有效保障面向老年人的基本公共服务供给。

改革创新，激发活力。坚持政府引导、市场驱动，深化简政放权、放管结合、优化服务改革，不断增强政府依法履职能力，加快形成统一开放、竞争有序的市场体系，保障公平竞争，改善营商环境，支持创业创新，激发市场活力。

统筹兼顾，协调发展。坚持把应对人口老龄化与促进经济社会发展相结合，促进老龄事业发展和养老体系建设城乡协调、区域协调、事业产业协调，统筹做好老年人经济保障、服务保障和精神关爱等制度安排，实现协调可持续发展。

**第三节　发展目标**

到2020年，老龄事业发展整体水平明显提升，养老体系更加健全完善，及时应对、科学应对、综合应对人口老龄化的社会基础更加牢固。

多支柱、全覆盖、更加公平、更可持续的社会保障体系更加完善。城镇职工和城乡居民基本养老保险参保率达到90%，基本医疗保险参保率稳定在95%以上，社会保险、社会福利、社会救助等社会保障制度和公益慈善事业有效衔接，老年人的基本生活、基本医疗、基本照护等需求得到切实保障。

居家为基础、社区为依托、机构为补充、医养相结合的养老服务体系更加健全。养老服务供给能力大幅提高、质量明显改善、结构更加合理，多层次、多样化的养老服务更加方便可及，政府运营的养老床位数占当地养老床位总数的比例不超过50%，护理型床位占当地养老床位总数的比例不低于30%，65岁以上老年人健康管理率达到70%。

有利于政府和市场作用充分发挥的制度体系更加完备。老龄事业发展和养老体系建设的法治化、信息化、标准化、规范化程度明显提高。政府职能转变、“放管服”改革、行政效能提升成效显著。市场活力和社会创造力得到充分激发，养老服务和产品供给主体更加多元、内容更加丰富、质量更加优良，以信用为核心的新型市场监管机制建立完善。

支持老龄事业发展和养老体系建设的社会环境更加友好。全社会积极应对人口老龄化、自觉支持老龄事业发展和养老体系建设的意识意愿显著增强，敬老养老助老社会风尚更加浓厚，安全绿色便利舒适的老年宜居环境建设扎实推进，老年文化体育教育事业更加繁荣发展，老年人合法权益得到有效保护，老年人参与社会发展的条件持续改善。

| **专栏2　“十三五”期间国家老龄事业发展和养老体系建设主要指标** | | |
| --- | --- | --- |
| **类　别** | **指　标** | **目标值** |
| 社会保障 | 基本养老保险参保率 | 达到90% |
|  | 基本医疗保险参保率 | 稳定在95%以上 |
| 养老服务 | 政府运营的养老床位占比 | 不超过50% |
|  | 护理型养老床位占比 | 不低于30％ |
| 健康支持 | 老年人健康素养 | 提升至10% |
|  | 二级以上综合医院设老年病科比例 | 35%以上 |
|  | 65岁以上老年人健康管理率 | 达到70% |
| 精神文化生活 | 建有老年学校的乡镇（街道）比例 | 达到50% |
|  | 经常性参与教育活动的老年人口比例 | 20%以上 |
| 社会参与 | 老年志愿者注册人数占老年人口比例 | 达到12% |
|  | 城乡社区基层老年协会覆盖率 | 90%以上 |
| 投入保障 | 福彩公益金用于养老服务业的比例 | 50%以上 |

第三章　健全完善社会保障体系

**第一节　社会保险制度**

完善养老保险制度。制定实施完善和改革基本养老保险制度总体方案。完善社会统筹与个人账户相结合的基本养老保险制度，构建包括职业年金、企业年金，以及个人储蓄性养老保险和商业保险的多层次养老保险体系。推进个人税收递延型商业养老保险试点。建立基本养老金合理调整机制，适当提高退休人员基本养老金标准。加快健全社会保障管理体制和经办服务体系。建立更加便捷的养老保险转移接续机制。

健全医疗保险制度。健全稳定可持续筹资和报销比例调整机制，完善缴费参保政策。加快推进基本医疗保险全国联网和异地就医结算，实现跨省异地安置退休人员住院费用直接结算。鼓励有条件的地方研究将基本治疗性康复辅助器具按规定逐步纳入基本医疗保险支付范围。巩固完善城乡居民大病保险。鼓励发展补充医疗保险和商业健康保险、老年人意外伤害保险。

探索建立长期护理保险制度。开展长期护理保险试点的地区要统筹施策，做好长期护理保险与重度残疾人护理补贴、经济困难失能老年人护理补贴等福利性护理补贴项目的整合衔接，提高资源配置效率效益。鼓励商业保险公司开发适销对路的长期护理保险产品和服务，满足老年人多样化、多层次长期护理保障需求。

**第二节　社会福利制度**

制定实施老年人照顾服务项目，鼓励地方丰富照顾服务项目、创新和优化照顾服务提供方式。着力保障特殊困难老年人的养老服务需求，确保人人能够享有基本养老服务。在全国范围内基本建成针对经济困难的高龄、失能老年人的补贴制度。对经济困难的老年人，地方各级人民政府逐步给予养老服务补贴。完善农村计划生育家庭奖励扶助和特别扶助制度。

**第三节　社会救助制度**

确保所有符合条件的老年人按规定纳入最低生活保障、特困人员救助供养等社会救助制度保障范围。完善医疗救助制度，全面开展重特大疾病医疗救助，逐步将低收入家庭老年人纳入救助范围。完善临时救助制度，加强对老年人的“救急难”工作，按规定对流浪乞讨、遭受遗弃等生活无着老年人给予救助。落实农村最低生活保障制度与扶贫开发政策有效衔接有关政策要求，确保现行扶贫标准下农村贫困老年人实现脱贫。

**第四节　公益慈善事业**

鼓励面向老年人开展募捐捐赠、志愿服务、慈善信托、安全知识教育、急救技能培训、突发事故防范等形式多样的公益慈善活动。依法加强对公益慈善组织和公益慈善活动的扶持和监管，依法及时查处以公益慈善为名实施的侵害老年人合法权益等违反法律法规、违背公序良俗的行为。加强民政部门与公益慈善组织、社会服务机构之间的信息对接和工作衔接，实现政府救助与社会帮扶有机结合。

第四章　健全养老服务体系

**第一节　夯实居家社区养老服务基础**

大力发展居家社区养老服务。逐步建立支持家庭养老的政策体系，支持成年子女与老年父母共同生活，履行赡养义务和承担照料责任。支持城乡社区定期上门巡访独居、空巢老年人家庭，帮助老年人解决实际困难。支持城乡社区发挥供需对接、服务引导等作用，加强居家养老服务信息汇集，引导社区日间照料中心等养老服务机构依托社区综合服务设施和社区公共服务综合信息平台，创新服务模式，提升质量效率，为老年人提供精准化个性化专业化服务。鼓励老年人参加社区邻里互助养老。鼓励有条件的地方推动扶持残疾、失能、高龄等老年人家庭开展适应老年人生活特点和安全需要的家庭住宅装修、家具设施、辅助设备等建设、配备、改造工作，对其中的经济困难老年人家庭给予适当补助。大力推行政府购买服务，推动专业化居家社区养老机构发展。

加强社区养老服务设施建设。统筹规划发展城乡社区养老服务设施，新建城区和新建居住（小）区按要求配套建设养老服务设施，老城区和已建成居住（小）区无养老服务设施或现有设施未达到规划要求的，通过购置、置换、租赁等方式建设。加强社区养老服务设施与社区综合服务设施的整合利用。支持在社区养老服务设施配备康复护理设施设备和器材。鼓励有条件的地方通过委托管理等方式，将社区养老服务设施无偿或低偿交由专业化的居家社区养老服务项目团队运营。

| **专栏3　居家社区养老服务工程** |
| --- |
| 依托城乡社区公共服务综合信息平台，以失能、独居、空巢老年人为重点，整合建立居家社区养老服务信息平台、呼叫服务系统和应急救援服务机制，方便养老服务机构和组织向居家老年人提供助餐、助洁、助行、助浴、助医、日间照料等服务。  实施“互联网+”养老工程。支持社区、养老服务机构、社会组织和企业利用物联网、移动互联网和云计算、大数据等信息技术，开发应用智能终端和居家社区养老服务智慧平台、信息系统、APP应用、微信公众号等，重点拓展远程提醒和控制、自动报警和处置、动态监测和记录等功能，规范数据接口，建设虚拟养老院。 |

**第二节　推动养老机构提质增效**

加快公办养老机构改革。加快推进具备向社会提供养老服务条件的公办养老机构转制为企业或开展公建民营。实行老年人入住评估制度，优先保障特困供养人员集中供养需求和其他经济困难的孤寡、失能、高龄等老年人的服务需求。完善公建民营养老机构管理办法，鼓励社会力量通过独资、合资、合作、联营、参股、租赁等方式参与公办养老机构改革。政府投资建设和购置的养老设施、新建居住（小）区按规定配建并移交给民政部门的养老设施、党政机关和国有企事业单位培训疗养机构等改建的养老设施，均可实施公建民营。

支持社会力量兴办养老机构。贯彻全面放开养老服务市场、提升养老服务质量的有关政策要求，加快推进养老服务业“放管服”改革。对民间资本和社会力量申请兴办养老机构进一步放宽准入条件，加强开办支持和服务指导。落实好对民办养老机构的投融资、税费、土地、人才等扶持政策。鼓励采取特许经营、政府购买服务、政府和社会资本合作等方式支持社会力量举办养老机构。允许养老机构依法依规设立多个服务网点，实现规模化、连锁化、品牌化运营。鼓励整合改造企业厂房、商业设施、存量商品房等用于养老服务。

全面提升养老机构服务质量。加快建立全国统一的服务质量标准和评价体系，完善安全、服务、管理、设施等标准，加强养老机构服务质量监管。建立健全养老机构分类管理和养老服务评估制度，引入第三方评估，实行评估结果报告和社会公示。加强养老服务行业自律和信用体系建设。支持发展养老机构责任保险，提高养老机构抵御风险能力。

**第三节　加强农村养老服务**

推动农村特困人员供养服务机构服务设施和服务质量达标，在保障农村特困人员集中供养需求的前提下，积极为低收入、高龄、独居、残疾、失能农村老年人提供养老服务。通过邻里互助、亲友相助、志愿服务等模式和举办农村幸福院、养老大院等方式，大力发展农村互助养老服务。发挥农村基层党组织、村委会、老年协会等作用，积极培育为老服务社会组织，依托农村社区综合服务中心（站）、综合性文化服务中心、村卫生室、农家书屋、全民健身等设施，为留守、孤寡、独居、贫困、残疾等老年人提供丰富多彩的关爱服务。

第五章　健全健康支持体系

**第一节　推进医养结合**

完善医养结合机制。统筹落实好医养结合优惠扶持政策，深入开展医养结合试点，建立健全医疗卫生机构与养老机构合作机制，建立养老机构内设医疗机构与合作医院间双向转诊绿色通道，为老年人提供治疗期住院、康复期护理、稳定期生活照料以及临终关怀一体化服务。大力开发中医药与养老服务相结合的系列服务产品，鼓励社会力量举办以中医药健康养老为主的护理院、疗养院，建设一批中医药特色医养结合示范基地。

支持养老机构开展医疗服务。支持养老机构按规定开办康复医院、护理院、临终关怀机构和医务室、护理站等。鼓励执业医师到养老机构设置的医疗机构多点执业，支持有相关专业特长的医师及专业人员在养老机构开展疾病预防、营养、中医养生等非诊疗性健康服务。对养老机构设置的医疗机构，符合条件的按规定纳入基本医疗保险定点范围。

**第二节　加强老年人健康促进和疾病预防**

开展老年人健康教育，促进健康老龄化理念和医疗保健知识宣传普及进社区、进家庭，增强老年人的自我保健意识和能力。加强对老年人健康生活方式和健身活动指导，提升老年人健康素养水平至10%。基层医疗卫生机构为辖区内65周岁以上老年人普遍建立健康档案，开展健康管理服务。加强对老年人心脑血管疾病、糖尿病、恶性肿瘤、呼吸系统疾病、口腔疾病等常见病、慢性病的健康指导、综合干预。指导老年人合理用药，减少不合理用药危害。研究推广老年病防治适宜技术，及时发现健康风险因素，促进老年病早发现、早诊断、早治疗。面向老年人开展中医药健康管理服务项目。加强老年严重精神障碍患者的社区管理和康复服务。

**第三节　发展老年医疗与康复护理服务**

加强老年康复医院、护理院、临终关怀机构和综合医院老年病科建设。有条件的地区可将部分公立医院转为康复、护理等机构。提高基层医疗卫生机构康复护理床位占比，积极开展家庭医生签约服务，为老年人提供连续的健康管理和医疗服务。到2020年，35%以上的二级以上综合医院设立老年病科。落实老年人医疗服务优待政策，为老年人特别是高龄、重病、残疾、失能老年人就医提供便利服务。鼓励各级医疗卫生机构和医务工作志愿者为老年人开展义诊。加强康复医师、康复治疗师、康复辅助器具配置人才培养，广泛开展偏瘫肢体综合训练、认知知觉功能康复训练等老年康复护理服务。

**第四节　加强老年体育健身**

结合贯彻落实全民健身计划，依托公园、广场、绿地等公共设施及旧厂房、仓库、老旧商业设施等城市空置场所，建设适合老年人体育健身的场地设施，广泛开展老年人康复健身体育活动。支持乡镇（街道）综合文化站建设体育健身场地，配备适合老年人的设施和器材。支持公共和民办体育设施向老年人免费或优惠开放。加强老年人体育健身方法和项目研究，分层分类引导老年运动项目发展。继续举办全国老年人体育健身大会。鼓励发展老年人体育组织，到2020年，90%的街道和乡镇建立老年人基层体育组织，城乡社区普遍建立老年人健身活动站点和体育团队。

第六章　繁荣老年消费市场

**第一节　丰富养老服务业态**

大力发展养老服务企业，鼓励连锁化经营、集团化发展，实施品牌战略，培育一批各具特色、管理规范、服务标准的龙头企业，加快形成产业链长、覆盖领域广、经济社会效益显著的养老服务产业集群。支持养老服务产业与健康、养生、旅游、文化、健身、休闲等产业融合发展，丰富养老服务产业新模式、新业态。鼓励金融、地产、互联网等企业进入养老服务产业。利用信息技术提升健康养老服务质量和效率。

**第二节　繁荣老年用品市场**

增加老年用品供给。引导支持相关行业、企业围绕健康促进、健康监测可穿戴设备、慢性病治疗、康复护理、辅助器具和智能看护、应急救援、通信服务、电子商务、旅游休闲等重点领域，推进老年人适用产品、技术的研发和应用。支持老年用品制造业创新发展，采用新工艺、新材料、新技术，促进产品升级换代。丰富适合老年人的食品、药品、服装等供给；加强老年用品测试和质量监管，鼓励开辟老年用品展示、体验场所，发展老年用品租赁市场，支持办好老龄产业博览会。

提升老年用品科技含量。加强对老年用品产业共性技术的研发和创新。支持推动老年用品产业领域大众创业、万众创新。支持符合条件的老年用品企业牵头承担各类科技计划（专项、基金等）科研项目。支持技术密集型企业、科研院所、高校及老龄科研机构加强适老科技研发和成果转化应用。落实相关税收优惠政策，支持老年用品产业领域科技创新与应用项目。

第七章　推进老年宜居环境建设

**第一节　推动设施无障碍建设和改造**

严格执行无障碍环境建设相关法律法规，完善涉老工程建设标准规范体系，在规划、设计、施工、监理、验收、运行、维护、管理等环节加强相关标准的实施与监督。加强与老年人自主安全地通行道路、出入相关建筑物、搭乘公共交通工具、交流信息、获得社区服务密切相关的公共设施的无障碍设计与改造。加强居住区公共设施无障碍改造，重点对坡道、楼梯、电梯、扶手等公共建筑节点进行改造。探索鼓励市场主体参与无障碍设施建设和改造的政策措施。

**第二节　营造安全绿色便利生活环境**

在推进老旧居住（小）区改造、棚户区改造、农村危房改造等工程中优先满足符合住房救助条件的老年人的基本住房安全需求。加强对养老服务设施的安全隐患排查和监管。加强养老服务设施节能宜居改造，将各类养老机构和城乡社区养老服务设施纳入绿色建筑行动重点扶持范围。推动老年人共建共享绿色社区、传统村落、美丽宜居村庄和生态文明建设成果。支持多层老旧住宅加装电梯。引导、支持开发老年宜居住宅和代际亲情住宅。继续推进街道、社区“老年人生活圈”配套设施建设，为老年人提供一站式便捷服务。

| **专栏4　老年宜居环境建设示范行动** |
| --- |
| 完善老年宜居环境建设评价标准体系，开展“老年友好型城市”和“老年宜居社区”建设示范行动，继续开展全国无障碍建设城市创建工作。到2020年，60%以上城市社区达到老年宜居社区基本条件，40%以上农村具备老年宜居社区基本条件，大部分老年人的基本公共服务需求能够在社区得到满足。 |

**第三节　弘扬敬老养老助老的社会风尚**

把敬老养老助老纳入社会公德、职业道德、家庭美德、个人品德建设，纳入文明城市、文明村镇、文明单位、文明校园、文明家庭考评。利用春节、清明节、中秋节、重阳节等传统节日，开展创意新、影响大、形式多的宣传教育活动，推动敬老养老助老教育进学校、进家庭、进机关、进社区。继续开展“敬老月”和全国敬老爱老助老评选表彰活动。推进非本地户籍常住老年人与本地户籍老年人同等享受优待。到2020年，老年人优待制度普遍建立完善。

第八章　丰富老年人精神文化生活

**第一节　发展老年教育**

落实老年教育发展规划，扩大老年教育资源供给，拓展老年教育发展路径，加强老年教育支持服务，创新老年教育发展机制，促进老年教育可持续发展，优先发展城乡社区老年教育，促进各级各类学校开展老年教育，部门、行业企业、高校举办的老年大学要进一步提高面向社会办学开放度，支持鼓励各类社会力量举办或参与老年教育。实施社会主义核心价值观培育、老年教育机构基础能力提升、学习资源建设整合、远程老年教育推进等计划。到2020年，基本形成覆盖广泛、灵活多样、特色鲜明、规范有序的老年教育新格局。全国县级以上城市至少应有一所老年大学。

| **专栏5　老年教育机构基础能力提升计划** |
| --- |
| 改善现有老年大学（学校）办学条件。建设一批在本区域发挥示范作用的乡镇（街道）老年人学习场所。改善基层社区老年教育机构设施设备，建设好村、社区老年学习点。探索“养、医、体、文”等场所与老年人学习场所的结合。开展养教结合试点。编辑出版有时代特色、科学内涵、文化品位，适应社会需要的老年教育系列教材。 |

**第二节　繁荣老年文化**

完善覆盖城乡的公共文化设施网络，在基层公共文化设施内开辟适宜老年人的文化娱乐活动场所，增加适合老年人的特色文化服务项目。推动公共文化服务设施向老年人免费或优惠开放，为老年人开展文化活动提供便利。文化信息资源共享、农村电影放映、农家书屋等重大文化惠民工程增加面向老年人的服务内容和资源。广泛开展群众性老年文化活动，培育老年文化活动品牌。鼓励创作发行老年人喜闻乐见的图书、报刊以及影视剧、戏剧、广播剧等文艺作品。鼓励制作适合微博、微信、手机客户端等新媒体传播的优秀老年文化作品。加强数字图书馆建设，拓展面向老年人的数字资源服务。加强专业人才和业余爱好者相结合的老年文化队伍建设。

**第三节　加强老年人精神关爱**

健全老年人精神关爱、心理疏导、危机干预服务网络，督促家庭成员加强对老年人的情感关怀和心理沟通；依托专业精神卫生机构和社会工作服务机构、专业心理工作者和社会工作者开展老年心理健康服务试点，为老年人提供心理关怀和精神关爱；支持企事业单位、社会组织、志愿者等社会力量开展形式多样的老年人关爱活动。鼓励城乡社区为老年人精神关爱提供活动场地、工作条件等支持。

第九章　扩大老年人社会参与

**第一节　培育积极老龄观**

引导老年人树立终身发展理念，始终保持自尊自爱自信自强的精神状态，积极面对老年生活，参与社会发展，发挥正能量，作出新贡献。引导全社会正确认识、积极接纳、大力支持老年人参与社会发展。

**第二节　加强老年人力资源开发**

将老年人才开发利用纳入各级人才队伍建设总体规划，鼓励各地制定老年人才开发利用专项规划。鼓励专业技术领域人才延长工作年限。鼓励各有关方面建立老年人才信息库，实现互联互通、资源共享。支持老年人才自主创业，帮助有意愿且身体状况允许的贫困老年人和其他老年人接受岗位技能培训或农业实用技术培训，通过劳动脱贫或致富。推动用人单位与受聘老年人依法签订书面协议。依法保障老年人在生产劳动过程中的合法收入、安全和健康权益。对老有所为贡献突出的老年人和在老有所为工作中贡献突出的单位、个人，可按规定给予表彰或奖励。

**第三节　发展老年志愿服务**

支持老年人积极参与基层民主监督、社会治安、公益慈善、移风易俗、民事调解、文教卫生、全民健身等工作。发挥老年人优良品行传帮带作用，支持老党员、老专家、老军人、老劳模、老干部开展关心教育下一代活动。深入开展“银龄行动”，组织医疗卫生、文化教育、农业科技等老专家、老知识分子参与东部援助西部、发达地区援助落后地区等志愿服务。推行志愿服务记录制度，鼓励老年人参加志愿服务，到2020年老年志愿者注册人数达到老年人口总数的12%。

**第四节　引导基层老年社会组织规范发展**

坚持扶持发展和规范管理并重，加强老年社会组织的培育扶持和登记管理。采取政府购买服务等措施加大对公益性、互助性、服务性、专业性基层老年社会组织的支持力度。继续推动老年社会组织加强能力建设和规范化建设，提高专业素质、服务能力和社会公信力，促进老年人通过社会组织实现自我管理、自我教育、自我服务。支持老年社会组织参加或承办政府有关人才培养、项目开发、课题研究、咨询服务等活动。

| **专栏6　基层老年协会规范化建设工程** |
| --- |
| 多渠道筹措资金支持基层老年协会建设，改善基层老年协会活动设施和条件，加强基层老年协会骨干培训和活动辅导，鼓励专业人士在基层老年协会能力建设中发挥骨干作用。积极推进基层老年协会党建工作，探索发挥基层老年协会在促进当地发展、调解涉老纠纷、开展互助服务、活跃老年人精神文化生活等方面积极作用的有效方式和途径。城乡社区基层老年协会覆盖率达到90%以上。 |

第十章　保障老年人合法权益

**第一节　完善老龄事业法规政策体系**

完善老年人权益保障配套法规，积极听取老年人的意见建议，研究建立老年人监护制度，加快老年人社会服务、社会优待、社会参与等制度建设。健全优待老年人的财政投入、服务评价、检查监督、奖励表彰等政策。

**第二节　健全老年人权益保障机制**

健全贯彻老年人权益保障法律法规的联合执法、执法检查、综合评估等制度。充分发挥基层党组织、基层群众性自治组织、老年社会组织作用，完善维护老年人合法权益社会监督、矛盾纠纷排查调解、多部门快速反应联合查处综合治理等机制。做好老年人来信来访工作。建立老年人法律维权热线，加强老年人法律服务和法律援助，针对老年群体特点开展适应老年人特殊需求的专项法律服务活动。扩大老年人法律援助范围，拓展基层服务网络，推进法律援助工作站点向城市社区和农村延伸，方便老年人及时就近寻求法律帮助。重点做好农村和贫困、高龄、空巢、失能等特殊困难老年群体的法律服务、法律援助和司法救助。

**第三节　加大普法宣传教育力度**

落实国家“七五”普法规划要求，加强老年人权益保障法律法规普法宣传教育，深入结合“法律六进”活动，推动普法宣传教育规范化、常态化，强化全社会维护老年人合法权益的法治观念。开展更多适合老年人的法治宣传活动，帮助老年人学法、懂法、用法，提高守法意识和依法维权意识。

第十一章　强化工作基础和规划实施保障

**第一节　强化工作基础保障**

推进信息化建设。落实促进大数据发展行动纲要，在切实保障数据安全的前提下，着力推动各有关部门涉及老年人的人口、保障、服务、信用、财产等基础信息分类分级互联共享，消除信息孤岛。在此基础上推动搭建全国互联、上下贯通的老龄工作信息化平台，加强涉老数据、信息的汇集整合和发掘运用，建立基于大数据的可信统计分析决策机制。支持各地积极推进为老服务综合信息平台在城市社区全覆盖、在农村地区扩大覆盖，推进信息惠民服务向老年人覆盖、数据资源向社会开放，更好地服务于保障改善老年人民生和大众创业、万众创新。

完善投入机制。各级政府要根据经济社会发展状况和老年人口增长情况，建立稳定的老龄事业经费投入保障机制。民政部本级彩票公益金和地方各级政府用于社会福利事业的彩票公益金，50%以上要用于支持发展养老服务业，并随老年人口的增加逐步提高投入比例。落实和完善鼓励政策，引导各类社会资本投入老龄事业，倡导社会各界对老龄事业进行慈善捐赠，形成财政资金、社会资本、慈善基金等多元结合的投入机制。

壮大人才队伍。推进涉老相关专业教育体系建设，加快培养老年医学、康复、护理、营养、心理和社会工作、经营管理、康复辅具配置等人才。建立以品德、能力和业绩为导向的职称评价和技能等级评价制度，拓宽养老服务专业人员职业发展空间。推动各地保障和逐步提高养老服务从业人员薪酬待遇。

| **专栏7　人才培养工程** |
| --- |
| 在养老服务、医养结合、科技助老等重点领域，每年培养造就一批高层次人才，符合条件的享受人才引进政策，示范带动养老服务业发展。在全国各类养老服务机构中，培养选拔优秀护理员，提供居住落户、住房保障、子女就学等方面的政策扶持。  实施养老护理人员培养培训计划，“十三五”时期力争使全国养老机构护理人员都得到至少一次专业培训。  对各级老龄工作机构的人员定期开展老龄政策和相关知识培训。 |

加强基层工作。进一步完善老龄工作机制，保证城乡社区老龄工作有人抓、老年人事情有人管、老年人困难有人帮。建立基层老龄工作先进典型激励机制。继续推进离退休人员管理服务社会化，建立健全老年人原工作单位、居住社区、老年社会组织和基层党组织齐抓共管的工作机制。总结创建离退休干部基层服务型党组织的好经验好做法，积极探索老年社会组织党建工作的新途径新办法。探索建立工会、共青团、妇联、残联等群团组织参与老年人管理服务的常态化机制和制度化渠道。

加强科学研究和调查统计。按照深化中央财政科技计划（专项、基金等)管理改革的总体部署，通过优化整合后的国家科技计划（专项、基金等）、社会科学基金等支持老龄事业领域的科技创新、基础理论研究和政策应用研究。完善老龄科学学科体系，加快老龄科学人才培养。在高校、研究机构、企业和地方，设立一批老龄科学理论研究基地、老龄产业实践研究基地、老龄政策创制试点基地。组建高层次老龄问题智库，健全重大决策专家咨询制度。加强国家人口老龄化中长期应对策略研究。完善老龄事业统计指标体系，建立老龄事业统计公报定期发布制度。推动城乡老年人生活状况抽样调查制度化、常态化、规范化。

加强宣传和国际合作。坚持正确舆论导向，充分发挥各类媒体作用，加大对人口老龄化国情、老龄政策法规、老龄事业发展重大主题以及老龄工作典型人物、事迹、经验等的宣传报道力度，提升舆情研判引导能力，营造全社会关注老龄问题、关心老龄事业、支持老龄工作的良好氛围。加强对外宣传，适时向国际社会推介老龄事业发展中国模式，进一步提升我国在国际老龄领域影响力。积极参与全球及地区老龄问题治理，加强与联合国有关机构、国际涉老组织和有关国家的交流与合作。研究筹办应对人口老龄化相关国际会议。推动中国老龄事业发展与落实2030年可持续发展议程相关目标有机对接。

**第二节　强化规划实施保障**

加强组织领导。坚持党对老龄工作的统一领导，发挥各级党委总揽全局、协调各方的领导核心作用，为规划实施提供坚强保证；强化各级政府落实规划的主体责任，将本规划主要任务指标纳入当地经济社会发展规划，纳入为民办实事项目，纳入政府工作议事日程和目标责任考核内容。健全老龄工作体制机制，形成推进规划实施的合力。加强专家支持系统建设，建立由多学科、多领域专家参与的专家顾问制度，为规划实施提供技术咨询、评估和指导。

加强督促检查。全国老龄办、民政部、国家发展改革委会同有关部门，加强对各地的指导、督促，及时检查并向国务院报告工作进展情况。搭建社会监督平台，健全第三方评估机制，适时对规划执行情况进行评估，向社会公布评估结果。县级以上地方政府要结合实际制定本规划实施方案，细化相关指标，确保责任到位、工作到位、投入到位、见到实效。鼓励各地积极探索，勇于创新，创造性地实施规划。

**国务院办公厅关于制定和实施**

**老年人照顾服务项目的意见**
国办发〔2017〕52号

各省、自治区、直辖市人民政府，国务院各部委、各直属机构：

大力弘扬敬老养老助老社会风尚，做好老年人照顾服务工作，提升老年人的获得感和幸福感，是社会主义制度优越性的具体体现，是社会主义核心价值观的内在要求，是实现脱贫攻坚、全面建成小康社会的重要任务，是积极应对人口老龄化，推动民生改善、促进社会和谐的实际举措。根据《中华人民共和国老年人权益保障法》，经党中央、国务院同意，现就制定和实施老年人照顾服务项目提出如下意见：

**一、总体要求**

（一）指导思想。

全面贯彻党的十八大和十八届三中、四中、五中、六中全会精神，深入贯彻习近平总书记系列重要讲话精神和治国理政新理念新思想新战略，落实党中央、国务院关于老年人照顾服务工作的决策部署，从我国国情出发，立足老年人法定权益保障和服务需求，整合服务资源，拓展服务内容，创新服务方式，提升服务质量，让老年人享受到更多看得见、摸得着的实惠，使老年人共享改革发展成果，推动实现老有所养、老有所医、老有所为、老有所学、老有所乐。

（二）基本原则。

党政主导，社会参与。坚持党委领导、政府主导，发挥党委和政府在统筹规划、示范引领、监督管理等方面的作用。坚持社会参与、全民行动，注重发挥家庭养老的基础作用，鼓励和引导社会力量开展专业化、多元化照顾服务。

突出重点，适度普惠。根据经济社会发展水平细化照顾服务项目，合理确定照顾服务的对象、内容和标准，兼顾不同年龄特点，重点关注高龄、失能、贫困、伤残、计划生育特殊家庭等困难老年人的特殊需求。

因地制宜，循序渐进。引导和推动各地结合实际，积极稳妥地开展老年人照顾服务工作，坚持量力而行、稳步推进。鼓励有条件的地方探索创新、先行先试，为逐步扩大照顾服务范围积累经验。

政策衔接，强化服务。注重与社会保险、社会救助、社会福利、慈善事业等政策制度有效衔接，统筹各类服务资源，形成保障合力，让老年人享受更多优质、便捷、公平、安全的优先优惠服务。

城乡统筹，和谐共融。加大基本公共服务资源向农村倾斜配置力度，提高农村老年人照顾服务的可及性和便利性。强化照顾服务过程中的代际支持，营造互尊互爱互助的良好氛围，增进社会和谐。

**二、重点任务**

（一）全面建立针对经济困难高龄、失能老年人的补贴制度，并做好与长期护理保险的衔接。将符合最低生活保障条件的贫困家庭中的老年人全部纳入最低生活保障范围，实现应保尽保。

（二）发展居家养老服务，为居家养老服务企业发展提供政策支持。鼓励与老年人日常生活密切相关的各类服务行业为老年人提供优先、便利、优惠服务。大力扶持专业服务机构并鼓励其他组织和个人为居家老年人提供生活照料、医疗护理、精神慰藉等服务。鼓励和支持城乡社区社会组织和相关机构为失能老年人提供临时或短期托养照顾服务。

（三）除极少数超大城市需按政策落户外，80周岁及以上老年人可自愿随子女迁移户口，依法依规享受迁入地基本公共服务。

（四）推进老年宜居社区、老年友好城市建设。提倡在推进与老年人日常生活密切相关的公共设施改造中，适当配备老年人出行辅助器具。加强社区、家庭的适老化设施改造，优先支持老年人居住比例高的住宅加装电梯等。

（五）深化敬老月活动，各级党委和政府坚持每年组织开展走访慰问困难老年人活动。发挥基层服务型党组织和工会、共青团、妇联等群团组织以及城乡基层社会组织的优势，开展经常性为老志愿服务活动。

（六）农村老年人不承担兴办公益事业的筹劳义务。

（七）贫困老年人因合法权益受到侵害提起诉讼的，依法依规给予其法律援助和司法救助。鼓励律师事务所、公证处、司法鉴定机构、基层法律服务所等法律服务机构为经济困难老年人提供免费或优惠服务。

（八）进一步推动扩大法律援助覆盖面，降低法律援助门槛，有条件的地方可适度放宽老年人申请法律援助的经济困难标准和受案范围。

（九）支持城市公共交通为老年人提供优惠和便利，鼓励公路、铁路、民航等公共交通工具为老年人提供便利服务。

（十）综合考虑老、幼、病、残、孕等重点旅客出行需求，有条件的公共交通场所、站点和公共交通工具要按照无障碍环境建设要求，加快无障碍设施建设和改造，在醒目位置设置老年人等重点人群服务标志，开辟候乘专区或专座，为无人陪同、行动不便等有服务需求的老年人提供便利服务。

（十一）鼓励通过基本公共卫生服务项目，为老年人免费建立电子健康档案，每年为65周岁及以上老年人免费提供包括体检在内的健康管理服务。

（十二）对符合条件的低收入家庭老年人参加城乡居民基本医疗保险所需个人缴费部分，由政府给予适当补贴。

（十三）加大推进医养结合力度，鼓励医疗卫生机构与养老服务融合发展，逐步建立完善医疗卫生机构与养老机构的业务合作机制，倡导社会力量兴办医养结合机构，鼓励有条件的医院为社区失能老年人设立家庭病床，建立巡诊制度。

（十四）积极开展长期护理保险试点，探索建立长期护理保险制度，切实保障失能人员特别是失能老年人的基本生活权益。

（十五）加快推进基本医疗保险异地就医结算工作，2017年底前基本实现符合转诊规定的老年人异地就医住院费用直接结算。

（十六）鼓励相关职业院校和培训机构每年面向老年人及其亲属开设一定学时的老年人护理、保健课程或开展专项技能培训。

（十七）鼓励制定家庭养老支持政策，引导公民自觉履行赡养义务和承担照料老年人责任。倡导制定老年人参与社会发展支持政策，发挥老年人积极作用。

（十八）推动具有相关学科的院校开发老年教育课程，为社区、老年教育机构及养老服务机构等提供教学资源及教育服务。支持兴办老年电视（互联网）大学，完善老年人社区学习网络。鼓励社会教育机构为老年人开展学习活动提供便利和优惠服务。

（十九）老年教育资源向老年人公平有序开放，减免贫困老年人进入老年大学（学校）学习的学费。提倡乡镇（街道）、城乡社区落实老年人学习场所，提供适合老年人的学习资源。

（二十）支持老年人开展文体娱乐、精神慰藉、互帮互助等活动，鼓励和支持为乡镇（街道）、城乡社区综合服务设施、为老服务机构和组织因地制宜配备适合老年人的文体器材。引导有条件的公共图书馆开设老年阅览区域，提供大字阅读设备、触屏读报系统等。

**三、组织实施**

（一）加强组织领导。各级党委和政府要充分认识做好老年人照顾服务工作的重要意义，将其列入议事日程和民心工程，纳入目标管理绩效考核内容，及时研究解决工作中遇到的困难和问题，做到认识到位、部署到位、措施到位、检查到位、落实到位。

（二）健全保障机制。县级以上政府要把老年人照顾服务工作所需资金和工作经费纳入财政预算。建立多渠道资金筹措机制，积极引导社会组织和企事业单位以结对帮扶、设立公益基金、开展公益捐赠等多种形式参与和支持老年人照顾服务工作。创新和优化照顾服务提供方式，加大政府购买服务力度，依据相关规定，通过市场化方式，把适合的老年人照顾服务项目交由具备条件的社会组织和企业承担。督促指导照顾服务提供方制定服务清单和办事指南，简化流程，提高效率。

（三）营造浓厚氛围。各地区、各部门要进一步强化服务意识，推动公共服务行业履行社会责任，为老年人提供更多更好的照顾服务。各级各类媒体要广泛宣传老年人照顾服务政策，积极开展敬老养老助老教育活动，大力宣传在老年人照顾服务工作中涌现出的先进单位和个人，按有关规定进行表彰奖励，努力营造全社会关心、支持、参与老年人照顾服务工作的良好氛围。

（四）强化督促检查。各地区、各部门要加大对老年人照顾服务工作的检查指导力度，健全综合督查、专项督查、第三方评估等工作机制。充分发挥社会监督的作用，建立健全信息反馈机制，妥善解决照顾服务过程中老年人反映的问题。强化问责机制，对落实老年人照顾服务政策不力的单位和个人要严肃追究责任。

**中华人民共和国老年人权益保障法**

1996年8月29日第八届全国人民代表大会常务委员会第二十一次会议通过　根据2009年8月27日第十一届全国人民代表大会常务委员会第十次会议《关于修改部分法律的决定》第一次修正　2012年12月28日第十一届全国人民代表大会常务委员会第三十次会议修订　根据2015年4月24日第十二届全国人民代表大会常务委员会第十四次会议《关于修改〈中华人民共和国电力法〉等六部法律的决定》第二次修正　根据2018年12月29日第十三届全国人民代表大会常务委员会第七次会议《关于修改〈中华人民共和国劳动法〉等七部法律的决定》第三次修正）

目录

**第一章　总则**

**第二章　家庭赡养与扶养**

**第三章　社会保障**

**第四章　社会服务**

**第五章　社会优待**

**第六章　宜居环境**

**第七章　参与社会发展**

**第八章　法律责任**

**第九章　附则**

第一章　总则

第一条　为了保障老年人合法权益，发展老龄事业，弘扬中华民族敬老、养老、助老的美德，根据宪法，制定本法。

第二条　本法所称老年人是指六十周岁以上的公民。

第三条　国家保障老年人依法享有的权益。

老年人有从国家和社会获得物质帮助的权利，有享受社会服务和社会优待的权利，有参与社会发展和共享发展成果的权利。

禁止歧视、侮辱、虐待或者遗弃老年人。

第四条　积极应对人口老龄化是国家的一项长期战略任务。

国家和社会应当采取措施，健全保障老年人权益的各项制度，逐步改善保障老年人生活、健康、安全以及参与社会发展的条件，实现老有所养、老有所医、老有所为、老有所学、老有所乐。

第五条　国家建立多层次的社会保障体系，逐步提高对老年人的保障水平。

国家建立和完善以居家为基础、社区为依托、机构为支撑的社会养老服务体系。

倡导全社会优待老年人。

第六条　各级人民政府应当将老龄事业纳入国民经济和社会发展规划，将老龄事业经费列入财政预算，建立稳定的经费保障机制，并鼓励社会各方面投入，使老龄事业与经济、社会协调发展。

国务院制定国家老龄事业发展规划。县级以上地方人民政府根据国家老龄事业发展规划，制定本行政区域的老龄事业发展规划和年度计划。

县级以上人民政府负责老龄工作的机构，负责组织、协调、指导、督促有关部门做好老年人权益保障工作。

第七条　保障老年人合法权益是全社会的共同责任。

国家机关、社会团体、企业事业单位和其他组织应当按照各自职责，做好老年人权益保障工作。

基层群众性自治组织和依法设立的老年人组织应当反映老年人的要求，维护老年人合法权益，为老年人服务。

提倡、鼓励义务为老年人服务。

第八条　国家进行人口老龄化国情教育，增强全社会积极应对人口老龄化意识。

全社会应当广泛开展敬老、养老、助老宣传教育活动，树立尊重、关心、帮助老年人的社会风尚。

青少年组织、学校和幼儿园应当对青少年和儿童进行敬老、养老、助老的道德教育和维护老年人合法权益的法制教育。

广播、电影、电视、报刊、网络等应当反映老年人的生活，开展维护老年人合法权益的宣传，为老年人服务。

第九条　国家支持老龄科学研究，建立老年人状况统计调查和发布制度。

第十条　各级人民政府和有关部门对维护老年人合法权益和敬老、养老、助老成绩显著的组织、家庭或者个人，对参与社会发展做出突出贡献的老年人，按照国家有关规定给予表彰或者奖励。

第十一条　老年人应当遵纪守法，履行法律规定的义务。

第十二条　每年农历九月初九为老年节。

第二章　家庭赡养与扶养

第十三条　老年人养老以居家为基础，家庭成员应当尊重、关心和照料老年人。

第十四条　赡养人应当履行对老年人经济上供养、生活上照料和精神上慰藉的义务，照顾老年人的特殊需要。

赡养人是指老年人的子女以及其他依法负有赡养义务的人。

赡养人的配偶应当协助赡养人履行赡养义务。

第十五条　赡养人应当使患病的老年人及时得到治疗和护理；对经济困难的老年人，应当提供医疗费用。

对生活不能自理的老年人，赡养人应当承担照料责任；不能亲自照料的，可以按照老年人的意愿委托他人或者养老机构等照料。

第十六条　赡养人应当妥善安排老年人的住房，不得强迫老年人居住或者迁居条件低劣的房屋。

老年人自有的或者承租的住房，子女或者其他亲属不得侵占，不得擅自改变产权关系或者租赁关系。

老年人自有的住房，赡养人有维修的义务。

第十七条　赡养人有义务耕种或者委托他人耕种老年人承包的田地，照管或者委托他人照管老年人的林木和牲畜等，收益归老年人所有。

第十八条　家庭成员应当关心老年人的精神需求，不得忽视、冷落老年人。

与老年人分开居住的家庭成员，应当经常看望或者问候老年人。

用人单位应当按照国家有关规定保障赡养人探亲休假的权利。

第十九条　赡养人不得以放弃继承权或者其他理由，拒绝履行赡养义务。

赡养人不履行赡养义务，老年人有要求赡养人付给赡养费等权利。

赡养人不得要求老年人承担力不能及的劳动。

第二十条　经老年人同意，赡养人之间可以就履行赡养义务签订协议。赡养协议的内容不得违反法律的规定和老年人的意愿。

基层群众性自治组织、老年人组织或者赡养人所在单位监督协议的履行。

第二十一条　老年人的婚姻自由受法律保护。子女或者其他亲属不得干涉老年人离婚、再婚及婚后的生活。

赡养人的赡养义务不因老年人的婚姻关系变化而消除。

第二十二条　老年人对个人的财产，依法享有占有、使用、收益和处分的权利，子女或者其他亲属不得干涉，不得以窃取、骗取、强行索取等方式侵犯老年人的财产权益。

老年人有依法继承父母、配偶、子女或者其他亲属遗产的权利，有接受赠与的权利。子女或者其他亲属不得侵占、抢夺、转移、隐匿或者损毁应当由老年人继承或者接受赠与的财产。

老年人以遗嘱处分财产，应当依法为老年配偶保留必要的份额。

第二十三条　老年人与配偶有相互扶养的义务。

由兄、姐扶养的弟、妹成年后，有负担能力的，对年老无赡养人的兄、姐有扶养的义务。

第二十四条　赡养人、扶养人不履行赡养、扶养义务的，基层群众性自治组织、老年人组织或者赡养人、扶养人所在单位应当督促其履行。

第二十五条　禁止对老年人实施家庭暴力。

第二十六条　具备完全民事行为能力的老年人，可以在近亲属或者其他与自己关系密切、愿意承担监护责任的个人、组织中协商确定自己的监护人。监护人在老年人丧失或者部分丧失民事行为能力时，依法承担监护责任。

老年人未事先确定监护人的，其丧失或者部分丧失民事行为能力时，依照有关法律的规定确定监护人。

第二十七条　国家建立健全家庭养老支持政策，鼓励家庭成员与老年人共同生活或者就近居住，为老年人随配偶或者赡养人迁徙提供条件，为家庭成员照料老年人提供帮助。

第三章　社　会　保　障

第二十八条　国家通过基本养老保险制度，保障老年人的基本生活。

第二十九条　国家通过基本医疗保险制度，保障老年人的基本医疗需要。享受最低生活保障的老年人和符合条件的低收入家庭中的老年人参加新型农村合作医疗和城镇居民基本医疗保险所需个人缴费部分，由政府给予补贴。

有关部门制定医疗保险办法，应当对老年人给予照顾。

第三十条　国家逐步开展长期护理保障工作，保障老年人的护理需求。

对生活长期不能自理、经济困难的老年人，地方各级人民政府应当根据其失能程度等情况给予护理补贴。

第三十一条　国家对经济困难的老年人给予基本生活、医疗、居住或者其他救助。

老年人无劳动能力、无生活来源、无赡养人和扶养人，或者其赡养人和扶养人确无赡养能力或者扶养能力的，由地方各级人民政府依照有关规定给予供养或者救助。

对流浪乞讨、遭受遗弃等生活无着的老年人，由地方各级人民政府依照有关规定给予救助。

第三十二条　地方各级人民政府在实施廉租住房、公共租赁住房等住房保障制度或者进行危旧房屋改造时，应当优先照顾符合条件的老年人。

第三十三条　国家建立和完善老年人福利制度，根据经济社会发展水平和老年人的实际需要，增加老年人的社会福利。

国家鼓励地方建立八十周岁以上低收入老年人高龄津贴制度。

国家建立和完善计划生育家庭老年人扶助制度。

农村可以将未承包的集体所有的部分土地、山林、水面、滩涂等作为养老基地，收益供老年人养老。

第三十四条　老年人依法享有的养老金、医疗待遇和其他待遇应当得到保障，有关机构必须按时足额支付，不得克扣、拖欠或者挪用。

国家根据经济发展以及职工平均工资增长、物价上涨等情况，适时提高养老保障水平。

第三十五条　国家鼓励慈善组织以及其他组织和个人为老年人提供物质帮助。

第三十六条　老年人可以与集体经济组织、基层群众性自治组织、养老机构等组织或者个人签订遗赠扶养协议或者其他扶助协议。

负有扶养义务的组织或者个人按照遗赠扶养协议，承担该老年人生养死葬的义务，享有受遗赠的权利。

第四章　社　会　服　务

第三十七条　地方各级人民政府和有关部门应当采取措施，发展城乡社区养老服务，鼓励、扶持专业服务机构及其他组织和个人，为居家的老年人提供生活照料、紧急救援、医疗护理、精神慰藉、心理咨询等多种形式的服务。

对经济困难的老年人，地方各级人民政府应当逐步给予养老服务补贴。

第三十八条　地方各级人民政府和有关部门、基层群众性自治组织，应当将养老服务设施纳入城乡社区配套设施建设规划，建立适应老年人需要的生活服务、文化体育活动、日间照料、疾病护理与康复等服务设施和网点，就近为老年人提供服务。

发扬邻里互助的传统，提倡邻里间关心、帮助有困难的老年人。

鼓励慈善组织、志愿者为老年人服务。倡导老年人互助服务。

第三十九条　各级人民政府应当根据经济发展水平和老年人服务需求，逐步增加对养老服务的投入。

各级人民政府和有关部门在财政、税费、土地、融资等方面采取措施，鼓励、扶持企业事业单位、社会组织或者个人兴办、运营养老、老年人日间照料、老年文化体育活动等设施。

第四十条　地方各级人民政府和有关部门应当按照老年人口比例及分布情况，将养老服务设施建设纳入城乡规划和土地利用总体规划，统筹安排养老服务设施建设用地及所需物资。

公益性养老服务设施用地，可以依法使用国有划拨土地或者农民集体所有的土地。

养老服务设施用地，非经法定程序不得改变用途。

第四十一条　政府投资兴办的养老机构，应当优先保障经济困难的孤寡、失能、高龄等老年人的服务需求。

第四十二条　国务院有关部门制定养老服务设施建设、养老服务质量和养老服务职业等标准，建立健全养老机构分类管理和养老服务评估制度。

各级人民政府应当规范养老服务收费项目和标准，加强监督和管理。

第四十三条　设立公益性养老机构，应当依法办理相应的登记。

设立经营性养老机构，应当在市场监督管理部门办理登记。

养老机构登记后即可开展服务活动，并向县级以上人民政府民政部门备案。

第四十四条　地方各级人民政府加强对本行政区域养老机构管理工作的领导，建立养老机构综合监管制度。

县级以上人民政府民政部门负责养老机构的指导、监督和管理，其他有关部门依照职责分工对养老机构实施监督。

第四十五条　县级以上人民政府民政部门依法履行监督检查职责，可以采取以下措施：

（一）向养老机构和个人了解情况；

（二）进入涉嫌违法的养老机构进行现场检查；

（三）查阅或者复制有关合同、票据、账簿及其他有关资料；

（四）发现养老机构存在可能危及人身健康和生命财产安全风险的，责令限期改正，逾期不改正的，责令停业整顿。

县级以上人民政府民政部门调查养老机构涉嫌违法的行为，应当遵守《中华人民共和国行政强制法》和其他有关法律、行政法规的规定。

第四十六条　养老机构变更或者终止的，应当妥善安置收住的老年人，并依照规定到有关部门办理手续。有关部门应当为养老机构妥善安置老年人提供帮助。

第四十七条　国家建立健全养老服务人才培养、使用、评价和激励制度，依法规范用工，促进从业人员劳动报酬合理增长，发展专职、兼职和志愿者相结合的养老服务队伍。

国家鼓励高等学校、中等职业学校和职业培训机构设置相关专业或者培训项目，培养养老服务专业人才。

第四十八条　养老机构应当与接受服务的老年人或者其代理人签订服务协议，明确双方的权利、义务。

养老机构及其工作人员不得以任何方式侵害老年人的权益。

第四十九条　国家鼓励养老机构投保责任保险，鼓励保险公司承保责任保险。

第五十条　各级人民政府和有关部门应当将老年医疗卫生服务纳入城乡医疗卫生服务规划，将老年人健康管理和常见病预防等纳入国家基本公共卫生服务项目。鼓励为老年人提供保健、护理、临终关怀等服务。

国家鼓励医疗机构开设针对老年病的专科或者门诊。

医疗卫生机构应当开展老年人的健康服务和疾病防治工作。

第五十一条　国家采取措施，加强老年医学的研究和人才培养，提高老年病的预防、治疗、科研水平，促进老年病的早期发现、诊断和治疗。

国家和社会采取措施，开展各种形式的健康教育，普及老年保健知识，增强老年人自我保健意识。

第五十二条　国家采取措施，发展老龄产业，将老龄产业列入国家扶持行业目录。扶持和引导企业开发、生产、经营适应老年人需要的用品和提供相关的服务。

第五章　社　会　优　待

第五十三条　县级以上人民政府及其有关部门根据经济社会发展情况和老年人的特殊需要，制定优待老年人的办法，逐步提高优待水平。

对常住在本行政区域内的外埠老年人给予同等优待。

第五十四条　各级人民政府和有关部门应当为老年人及时、便利地领取养老金、结算医疗费和享受其他物质帮助提供条件。

第五十五条　各级人民政府和有关部门办理房屋权属关系变更、户口迁移等涉及老年人权益的重大事项时，应当就办理事项是否为老年人的真实意思表示进行询问，并依法优先办理。

第五十六条　老年人因其合法权益受侵害提起诉讼交纳诉讼费确有困难的，可以缓交、减交或者免交；需要获得律师帮助，但无力支付律师费用的，可以获得法律援助。

鼓励律师事务所、公证处、基层法律服务所和其他法律服务机构为经济困难的老年人提供免费或者优惠服务。

第五十七条　医疗机构应当为老年人就医提供方便，对老年人就医予以优先。有条件的地方，可以为老年人设立家庭病床，开展巡回医疗、护理、康复、免费体检等服务。

提倡为老年人义诊。

第五十八条　提倡与老年人日常生活密切相关的服务行业为老年人提供优先、优惠服务。

城市公共交通、公路、铁路、水路和航空客运，应当为老年人提供优待和照顾。

第五十九条　博物馆、美术馆、科技馆、纪念馆、公共图书馆、文化馆、影剧院、体育场馆、公园、旅游景点等场所，应当对老年人免费或者优惠开放。

第六十条　农村老年人不承担兴办公益事业的筹劳义务。

第六章　宜　居　环　境

第六十一条　国家采取措施，推进宜居环境建设，为老年人提供安全、便利和舒适的环境。

第六十二条　各级人民政府在制定城乡规划时，应当根据人口老龄化发展趋势、老年人口分布和老年人的特点，统筹考虑适合老年人的公共基础设施、生活服务设施、医疗卫生设施和文化体育设施建设。

第六十三条　国家制定和完善涉及老年人的工程建设标准体系，在规划、设计、施工、监理、验收、运行、维护、管理等环节加强相关标准的实施与监督。

第六十四条　国家制定无障碍设施工程建设标准。新建、改建和扩建道路、公共交通设施、建筑物、居住区等，应当符合国家无障碍设施工程建设标准。

各级人民政府和有关部门应当按照国家无障碍设施工程建设标准，优先推进与老年人日常生活密切相关的公共服务设施的改造。

无障碍设施的所有人和管理人应当保障无障碍设施正常使用。

第六十五条　国家推动老年宜居社区建设，引导、支持老年宜居住宅的开发，推动和扶持老年人家庭无障碍设施的改造，为老年人创造无障碍居住环境。

第七章　参与社会发展

第六十六条　国家和社会应当重视、珍惜老年人的知识、技能、经验和优良品德，发挥老年人的专长和作用，保障老年人参与经济、政治、文化和社会生活。

第六十七条　老年人可以通过老年人组织，开展有益身心健康的活动。

第六十八条　制定法律、法规、规章和公共政策，涉及老年人权益重大问题的，应当听取老年人和老年人组织的意见。

老年人和老年人组织有权向国家机关提出老年人权益保障、老龄事业发展等方面的意见和建议。

第六十九条　国家为老年人参与社会发展创造条件。根据社会需要和可能，鼓励老年人在自愿和量力的情况下，从事下列活动：

（一）对青少年和儿童进行社会主义、爱国主义、集体主义和艰苦奋斗等优良传统教育；

（二）传授文化和科技知识；

（三）提供咨询服务；

（四）依法参与科技开发和应用；

（五）依法从事经营和生产活动；

（六）参加志愿服务、兴办社会公益事业；

（七）参与维护社会治安、协助调解民间纠纷；

（八）参加其他社会活动。

第七十条　老年人参加劳动的合法收入受法律保护。

任何单位和个人不得安排老年人从事危害其身心健康的劳动或者危险作业。

第七十一条　老年人有继续受教育的权利。

国家发展老年教育，把老年教育纳入终身教育体系，鼓励社会办好各类老年学校。

各级人民政府对老年教育应当加强领导，统一规划，加大投入。

第七十二条　国家和社会采取措施，开展适合老年人的群众性文化、体育、娱乐活动，丰富老年人的精神文化生活。

第八章　法　律　责　任

第七十三条　老年人合法权益受到侵害的，被侵害人或者其代理人有权要求有关部门处理，或者依法向人民法院提起诉讼。

人民法院和有关部门，对侵犯老年人合法权益的申诉、控告和检举，应当依法及时受理，不得推诿、拖延。

第七十四条　不履行保护老年人合法权益职责的部门或者组织，其上级主管部门应当给予批评教育，责令改正。

国家工作人员违法失职，致使老年人合法权益受到损害的，由其所在单位或者上级机关责令改正，或者依法给予处分；构成犯罪的，依法追究刑事责任。

第七十五条　老年人与家庭成员因赡养、扶养或者住房、财产等发生纠纷，可以申请人民调解委员会或者其他有关组织进行调解，也可以直接向人民法院提起诉讼。

人民调解委员会或者其他有关组织调解前款纠纷时，应当通过说服、疏导等方式化解矛盾和纠纷；对有过错的家庭成员，应当给予批评教育。

人民法院对老年人追索赡养费或者扶养费的申请，可以依法裁定先予执行。

第七十六条　干涉老年人婚姻自由，对老年人负有赡养义务、扶养义务而拒绝赡养、扶养，虐待老年人或者对老年人实施家庭暴力的，由有关单位给予批评教育；构成违反治安管理行为的，依法给予治安管理处罚；构成犯罪的，依法追究刑事责任。

第七十七条　家庭成员盗窃、诈骗、抢夺、侵占、勒索、故意损毁老年人财物，构成违反治安管理行为的，依法给予治安管理处罚；构成犯罪的，依法追究刑事责任。

第七十八条　侮辱、诽谤老年人，构成违反治安管理行为的，依法给予治安管理处罚；构成犯罪的，依法追究刑事责任。

第七十九条　养老机构及其工作人员侵害老年人人身和财产权益，或者未按照约定提供服务的，依法承担民事责任；有关主管部门依法给予行政处罚；构成犯罪的，依法追究刑事责任。

第八十条　对养老机构负有管理和监督职责的部门及其工作人员滥用职权、玩忽职守、徇私舞弊的，对直接负责的主管人员和其他直接责任人员依法给予处分；构成犯罪的，依法追究刑事责任。

第八十一条　不按规定履行优待老年人义务的，由有关主管部门责令改正。

第八十二条　涉及老年人的工程不符合国家规定的标准或者无障碍设施所有人、管理人未尽到维护和管理职责的，由有关主管部门责令改正；造成损害的，依法承担民事责任；对有关单位、个人依法给予行政处罚；构成犯罪的，依法追究刑事责任。

**全国老龄办、中共中央组织部、中共中央宣传部、国家发展改革委、教育部、民政部、司法部、人力资源社会保障部、文化部、国家卫生计生委、国家新闻出版广电总局、全国总工会、共青团中央、全国妇联关于开展人口老龄化国情教育的通知**

增强全社会人口老龄化国情意识，根据全国老龄工作委员会第十九次全体会议部署，现就开展人口老龄化国情教育提出以下意见。
　　一、意义目标
　　（一）重要意义。人口老龄化是贯穿我国21世纪的基本国情，积极应对人口老龄化是国家的一项长期战略任务。在全社会开展人口老龄化国情教育，是贯彻落实***总书记关于加强老龄工作重要讲话和重要指示精神，开展积极应对人口老龄化行动的重要举措，有利于营造全社会关心、支持、参与积极应对人口老龄化的良好氛围，激发全社会增强应对人口老龄化的主动性、针对性、自觉性，对于构建党委领导、政府主导、社会参与、全民行动的老龄工作大格局，确保我国老龄事业全面协调可持续发展，确保全体老年人共享改革发展成果，确保实现决胜全面建成小康社会、夺取新时代中国特色社会主义伟大胜利、实现中华民族伟大复兴中国梦的宏伟目标，具有重大而深远的战略意义。
　　（二）总体目标。人口老龄化国情教育要面向全社会，重点对象是党政干部、青少年和老年人。到2020年，人口老龄化的国情意识明显增强，关爱老年人的意识和老年人的自爱意识大幅提升，积极应对人口老龄化的社会氛围更加浓厚。 　　二、主要内容
　　（一）人口老龄化形势教育。要坚持不懈地用***总书记关于加强老龄工作重要讲话和重要指示精神武装头脑，全面阐释和深入分析我国人口老龄化的基本情况、主要特点、发展历程、深刻影响等，引导全社会准确把握人口老龄化对我国经济、政治、文化、社会、生态发展带来的机遇和挑战，正确看待我国人口老龄化的发展规律，进一步增强全社会及时应对、科学应对、综合应对人口老龄化的共识和观念。
　　（二）老龄政策法规教育。重点宣传和解读好《[中华人民共和国老年人权益保障法](javascript:SLC(252608))》《“十三五”国家老龄事业发展和养老体系建设规划》及《[关于制定和实施老年人照顾服务项目的意见](javascript:SLC(296575))》等重要老龄政策法规，宣传各级党委、政府出台的老龄政策法规，宣传其重要意义、创新举措、实际成效，加大老龄政策法规的执行力度，推动促进各项政策制度有效衔接，积极构建系统完备的老龄政策制度体系。
　　（三）应对人口老龄化成就教育。党中央、国务院历来高度重视老龄工作，特别是党的十九大提出“积极应对人口老龄化，构建养老、孝老、敬老政策体系和社会环境，推进医养结合，加快老龄事业和产业发展”。要积极向国内外宣传我国在应对人口老龄化过程中取得的巨大成就，宣传我国在老年人社会保障、养老服务业发展、健康老龄化、老年宜居环境、老年维权、老年优待、老年教育、老年文化体育、老年社会参与、老年社会组织、老年志愿服务等方面取得的积极进展，宣传各级党委和政府对老龄事业的重视支持和对广大老年人的关心关爱，提升全社会积极应对人口老龄化的信心和决心。
　　（四）孝亲敬老文化教育。开展具有民族特色、时代特征的孝亲敬老文化教育，把弘扬孝亲敬老文化纳入社会主义核心价值观宣传教育，激励人们向上向善、孝老爱亲。深入挖掘和阐发中华优秀敬老传统文化蕴含的思想观念、人文精神、道德规范，在历史文化传承中不断丰富教育内容。重视家庭建设，教育引导人们自觉承担家庭责任，树立良好家风，实现家庭和睦、代际和顺，巩固家庭养老基础地位。大力倡导全社会接纳、尊重、帮助老年人，宣传敬老典型，讲好敬老故事。
　　（五）积极老龄观教育。倡导全社会树立积极老龄观，积极看待老龄社会，积极看待老年人和老年生活，积极做好全生命周期养老准备。把广大老年人当作社会的宝贵财富，当作党执政兴国的重要资源，当作推进中国特色社会主义伟大事业的重要力量。充分发挥广大老年人的积极作用，对老年人进行自尊、自立、自强教育，引导广大老年人增强自爱意识。讲好老龄故事、传播老龄声音，发挥正能量，作出新贡献。 　　三、具体措施
　　（一）开展主题宣讲。把人口老龄化国情教育纳入大、中、小学教育教学内容及党校、行政学院和各级领导班子理论学习中心组和干部培训教育内容。组织编写人口老龄化国情教育知识读本和培训教材。建立由党政领导、专家学者、老龄工作者等组成的宣讲队伍，重点在机关、企业、学校、社区、养老机构、老年大学、基层老年协会等场所开展宣讲，围绕人口老龄化国情教育的主要内容，重点宣讲党的十九大精神，宣讲***总书记关于加强老龄工作重要讲话和重要指示精神，宣讲党中央、国务院关于加强老龄工作的决策部署，宣讲党的十八大以来我国老龄事业取得的辉煌成就，宣讲中华民族敬老爱老助老传统美德。
　　（二）进行集中宣传。中央和地方主流媒体要发挥引领作用，开设专刊专版专栏进行集中重点宣传，包括教育活动的部署、重要行动、社会反响、经验典型等。各级各类公共文化场所，要充分利用电子屏、宣传栏、展板、标语、海报等开展专题宣传。各类网站、微博、微信、客户端、公众号等新媒体要进行线上线下广泛宣传，确保活动有声音、有氛围、有影响。
　　（三）举办文化活动。各地、各有关部门要结合传统节庆日、“敬老月”、“老年节”等开展特色鲜明、吸引力强的人口老龄化国情教育主题活动。广泛运用知识竞赛、主题征文、文艺演出、书画创作、摄影展览、体育健身等有效途径，寓教育于文化活动之中。
　　（四）组织文艺创作。各地、各有关部门要坚持以人民为中心的创作导向，坚持思想精深、艺术精湛、制作精良相统一，要以满足老年人精神文化需求作为出发点和落脚点，重点规划实施一批反映老龄事业发展成就的文化项目，组织创作生产一批继承和发扬中华民族敬老传统文化的电影、电视剧、纪录片、动画片、节目栏目、音乐作品、图书等，广泛组织涉老题材优秀作品、敬老养老助老公益广告等展演展播活动，加大播出频率和力度。
　　（五）创新教育形式。各地、各有关部门要结合实际，针对不同的教育对象和活动阶段，探索创新开展人口老龄化国情教育的有效形式，及时总结活动的好做法和新成效，提高活动的参与性、实效性、针对性。 　　四、组织保障
　　（一）加强领导。各地、各有关部门要高度重视，加强领导，精心组织。要建立健全开展人口老龄化国情教育的领导和工作机制，把人口老龄化国情教育纳入年度工作计划，制定工作方案，明确工作要求，抓好组织实施。各级老龄部门作为人口老龄化国情教育的牵头单位，要切实履行综合协调职能，抓好统筹推进，抓好工作落实。
　　（二）明确分工。各地、各有关部门要加强协同配合，发挥各自优势，形成工作合力，共同推动人口老龄化国情教育开展。党委组织、宣传等部门要把人口老龄化国情教育纳入各级党校、行政学院、社会主义学院培训课程，纳入党委（党组）理论学习中心组学习内容。文化、新闻出版广电等部门要加强人口老龄化国情教育形势和成就的宣传及敬老文化项目的规划实施。发展改革、司法、民政、人力资源社会保障、卫生计生等部门要把人口老龄化国情教育纳入制定涉老政策规划并抓好实施。教育部门要把人口老龄化国情教育纳入大、中、小学教育教学内容。工会、共青团、妇联等部门要结合工作实际充分发挥密切联系群众的优势作用，积极开展人口老龄化国情教育特色活动。
　　（三）抓好督导。各地、各有关部门要加强指导，强化督查，把人口老龄化国情教育作为年度工作考核和《“十三五”国家老龄事业发展和养老体系建设规划》终期评估的重要内容。加大对基层单位的督促指导力度，落实责任，形成上下联动的工作态势。每年年底，各地、各有关部门要将活动开展情况进行汇总，以书面形式报全国老龄办。

**关于建立完善老年健康服务体系的指导意见**

各省、自治区、直辖市人民政府，国务院各部委、各直属机构：
　　当前，我国老年人口规模持续扩大，对健康服务的需求愈发迫切，为解决老年健康服务体系不健全，有效供给不足，发展不平衡不充分的问题，建立完善符合我国国情的老年健康服务体系，满足老年人日益增长的健康服务需求，根据《“健康中国2030”规划纲要》，经国务院同意，现提出如下意见。
　　一、总体要求
　　（一）指导思想。以***新时代中国特色社会主义思想为指导，全面贯彻党的十九大和十九届二中、三中全会精神，深入贯彻落实全国卫生与健康大会精神，以维护老年人健康权益为中心，以满足老年人健康服务需求为导向，大力发展老年健康事业，着力构建包括健康教育、预防保健、疾病诊治、康复护理、长期照护、安宁疗护的综合连续、覆盖城乡的老年健康服务体系，努力提高老年人健康水平，实现健康老龄化，建设健康中国。
　　（二）基本原则。
　　健康引领，全程服务。以大卫生、大健康的理念引领老年健康服务体系建设，将健康融入所有政策，着眼生命全过程，对影响健康的因素进行干预，提供综合连续的全程服务。
　　兜底保障，公平可及。以基层为重点，提高服务效能，保障经济困难的失能（含失智）、计划生育特殊家庭老年人的基本健康服务。促进资源优化配置，逐步缩小城乡、区域差距，促进老年健康服务公平可及。
　　政策支持，激发活力。履行政府在制定规划和政策、引导投入等方面的职责，发挥市场在资源配置中的决定性作用，激发市场活力，鼓励社会参与，满足多层次、多样化的老年健康服务需求。
　　统筹资源，共建共享。统筹政府各部门、社会各方面资源，动员引导全社会广泛参与，共同促进老年健康服务发展，实现共建共享。
　　（三）主要目标。到2022年，老年健康相关制度、标准、规范基本建立，老年健康服务机构数量显著增加，服务内容更加丰富，服务质量明显提升，服务队伍更加壮大，服务资源配置更趋合理，综合连续、覆盖城乡的老年健康服务体系基本建立，老年人的健康服务需求得到基本满足。 　　二、主要任务
　　（一）加强健康教育。利用多种方式和媒体媒介，面向老年人及其照护者开展健康教育活动，内容包括营养膳食、运动健身、心理健康、伤害预防、疾病预防、合理用药、康复护理、生命教育和中医养生保健等，促进老年人形成健康生活方式，提高老年人健康素养。积极开展中医药膳食疗科普等活动，推广中医传统运动项目，加强中医药健康养生养老文化宣传。开展老年健康宣传周等活动，宣传老年健康科学知识和相关政策，营造关心支持老年健康的社会氛围。老年大学和老年教育机构要将健康教育纳入课程体系和教学内容。依托社区服务中心、基层老龄协会、老年大学等，鼓励老年人积极参与社会活动，自觉主动维护身心健康。（国家卫生健康委、教育部、工业和信息化部、民政部、农业农村部、广电总局、体育总局、国家中医药局、中国老龄协会按职责分工负责）
　　（二）加强预防保健。建立健全老年健康危险因素干预、疾病早发现早诊断早治疗、失能预防三级预防体系。落实国家基本公共卫生服务项目，加强老年人健康管理，提供生活方式和健康状况评估、体格检查、辅助检查和健康指导服务，将老年人健康管理作为基本公共卫生服务项目绩效评价的重要内容，把老年人满意度作为重要评价指标，县（市、区）卫生健康行政部门要落实对绩效评价的主体责任，每年组织开展一次绩效评价。以老年人为重点，做实家庭医生签约服务。开展老年人营养改善行动，监测、评价和改善老年人营养状况。加强老年人群重点慢性病的早期筛查、早期干预及分类管理，积极开展阿尔茨海默病、帕金森病等神经退行性疾病的早期筛查和健康指导。实施失能预防项目，宣传失能预防核心信息，降低老年人失能发生率。加强适老环境建设和改造，减少老年人意外伤害。重视老年人心理健康，完善精神障碍类疾病的早期预防及干预机制，针对抑郁、焦虑等常见精神障碍和心理行为问题，开展心理健康状况评估和随访管理，为老年人特别是有特殊困难的老年人提供心理辅导、情绪纾解、悲伤抚慰等心理关怀服务。（国家卫生健康委、工业和信息化部、民政部、财政部、住房城乡建设部、国家中医药局按职责分工负责）
　　（三）加强疾病诊治。完善老年医疗资源布局，建立健全以基层医疗卫生机构为基础，老年医院和综合性医院老年医学科为核心，相关教学科研机构为支撑的老年医疗服务网络。有条件的二级及以上综合性医院要开设老年医学科，到2022年，二级及以上综合性医院设立老年医学科的比例达到50%。各地可根据实际，加大老年医院建设力度。重视老年人综合评估和老年综合征诊治，推动老年医疗服务从以疾病为中心的单病种模式向以患者为中心的多病共治模式转变。强化老年人用药保障，开展老年人用药使用监测，加强老年人用药指导，建立老年慢性疾病长期处方制度。开展社区和居家中医药健康服务，促进优质中医药资源向社区、家庭延伸。
　　全面落实老年人医疗服务优待政策，医疗机构普遍建立老年人挂号、就医绿色通道，优化老年人就医流程，为老年人看病就医提供便利服务。开展老年友善医疗卫生机构创建活动，推动医疗卫生机构开展适老化改造，开展老年友善服务，到2022年，80%以上的综合性医院、康复医院、护理院和基层医疗卫生机构成为老年友善医疗卫生机构。鼓励医疗卫生机构为居家失能老年人提供家庭病床、巡诊等上门医疗服务。（国家卫生健康委、国家发展改革委、财政部、国家中医药局按职责分工负责）
　　（四）加强康复和护理服务。充分发挥康复医疗在老年医疗服务中的作用，为老年患者提供早期、系统、专业、连续的康复医疗服务。大力发展老年护理服务，建立完善以机构为支撑、社区为依托、居家为基础的老年护理服务网络。开展中医特色老年人康复、护理服务。加强护理、康复医疗机构建设，鼓励医疗资源丰富的地区将部分公立医疗机构转型为护理、康复医疗机构，鼓励二级及以上综合性医院设立康复医学科，提高基层医疗卫生机构的康复、护理床位占比。支持农村医疗卫生机构利用现有富余编制床位开设康复、护理床位。到2022年，基层医疗卫生机构护理床位占比达到30%。（国家卫生健康委、国家发展改革委、民政部、财政部、国家中医药局按职责分工负责）
　　（五）加强长期照护服务。探索建立从居家、社区到专业机构的失能老年人长期照护服务模式。实施基本公共卫生服务项目，为失能老年人上门开展健康评估和健康服务。通过政府购买服务等方式，支持社区嵌入式为老服务机构发展。依托护理院（站）、护理中心、社区卫生服务中心、乡镇卫生院等医疗卫生机构以及具备提供长期照护服务能力的社区日间照料中心、乡镇敬老院等养老机构，为失能老年人提供长期照护服务。鼓励各地通过公建民营、政府购买服务、发放运营补贴等方式，支持各类医养结合机构接收经济困难的高龄失能老年人。
　　增加从事失能老年人护理工作的护士数量，鼓励退休护士从事失能老年人护理指导、培训和服务等工作。进一步开展职业技能培训和就业指导服务，充实长期照护服务队伍。面向居家失能老年人照护者开展应急救护和照护技能培训，提高家庭照护者的照护能力和水平。（国家卫生健康委、教育部、民政部、财政部、人力资源社会保障部按职责分工负责）
　　（六）加强安宁疗护服务。根据医疗机构的功能和定位，推动相应医疗卫生机构，按照患者“充分知情、自愿选择”的原则开展安宁疗护服务，开设安宁疗护病区或床位，有条件的地方可建设安宁疗护中心，加快安宁疗护机构标准化、规范化建设。积极开展社区和居家安宁疗护服务。探索建立机构、社区和居家安宁疗护相结合的工作机制，形成畅通合理的转诊制度。制定安宁疗护进入和用药指南。营利性医疗机构可自行确定安宁疗护服务内容和收费标准。非营利性医疗机构提供的安宁疗护服务，属于治疗、护理、检查检验等医疗服务的，按现有项目收费；属于关怀慰藉、生活照料等非医疗服务的，不作为医疗服务价格项目管理，收费标准由医疗机构自主确定。
　　建立完善安宁疗护多学科服务模式，为疾病终末期患者提供疼痛及其他症状控制、舒适照护等服务，对患者及家属提供心理支持和人文关怀。加强对公众的宣传教育，将生命教育纳入中小学校健康课程，推动安宁疗护理念得到社会广泛认可和接受。认真总结安宁疗护试点经验，稳步扩大试点。（国家卫生健康委、国家发展改革委、教育部、国家医保局按职责分工负责） 　　三、保障措施
　　（一）强化标准建设。制定老年人健康干预及评价标准。建立健全长期照护服务标准和管理规范，制定长期照护专业人员职业技能标准。制定老年医疗、康复、护理、安宁疗护等老年健康服务机构基本标准和服务规范，制定综合医院老年医学科建设和管理指南，制定老年友善医疗卫生机构标准。研究完善上门医疗护理和家庭病床服务的内容、标准、规范及收费和支付政策，建立健全保障机制，鼓励相关机构投保责任险、医疗意外险、人身意外险等，防范应对执业风险和人身安全风险，适当提高上门服务人员的待遇水平。（国家卫生健康委、民政部、人力资源社会保障部、市场监管总局、国家医保局、银保监会、中国残联按职责分工负责）
　　（二）强化政策支持。各地要积极出台实施扶持政策，在土地供应、政府购买服务等方面对老年健康服务发展予以支持和倾斜。鼓励社会力量举办老年医院、康复医院、护理院、安宁疗护中心等。加大对贫困地区老年健康服务机构建设的支持力度，推动实现城乡、区域老年健康服务均等化。全面建立经济困难的高龄、失能老年人补贴制度，并做好与长期护理保险制度的衔接。研究建立稳定可持续的筹资机制，推动形成符合国情的长期护理保险制度框架。（国家发展改革委、民政部、财政部、国家医保局、银保监会按职责分工负责）
　　（三）强化学科发展。推进老年医学研究中心、国家老年疾病临床医学研究中心等创新基地建设，打造高水平的技术创新与成果转化基地。加强老年健康相关科学研究，通过各级财政科技计划支持老年健康相关预防、诊断、治疗技术和产品研发。加强老年健康相关适宜技术研发与推广。引导普通高校和职业院校开设老年医学、药学、护理、康复、心理、安宁疗护等相关专业和课程，开展学历教育。（教育部、科技部、国家卫生健康委、国家中医药局按职责分工负责）
　　（四）强化队伍建设。加强老年健康人才培养，支持开展老年健康服务相关从业人员的继续教育，壮大老年健康人才队伍。加强老年健康促进、老年医学及其相关专业人员培训，建立培训机制，建设培训基地，提高相关人员的服务能力和水平。扩大老年护理服务队伍，补齐服务短板，到2022年基本满足老年人护理服务需求。完善老年健康相关职业资格认证制度和以技术技能价值激励为导向的薪酬分配体系，拓宽职业发展前景。（国家卫生健康委、教育部、民政部、人力资源社会保障部、国家中医药局按职责分工负责）
　　（五）强化信息支撑。充分利用人工智能等技术，研发可穿戴的老年人健康支持技术和设备，探索开展远程实时查看、实时定位、健康监测、紧急救助呼叫等服务。加强老年健康服务相关信息系统建设，促进各类健康数据的汇集和融合，整合信息资源，实现信息共享。积极探索“互联网＋老年健康”服务模式，推动线上线下结合，开展一批智慧健康服务示范项目。（国家卫生健康委、工业和信息化部、民政部按职责分工负责）
　　（六）强化组织保障。建立政府主导、部门协作、社会参与的工作机制，各地各有关部门要高度重视老年健康服务体系建设，将其纳入经济社会发展相关规划，纳入深化医药卫生体制改革和促进养老、健康服务业发展的总体部署，结合实际制定老年健康服务体系建设的具体规划和实施办法。

**国务院关于实施健康中国行动的意见**

各省、自治区、直辖市人民政府，国务院各部委、各直属机构：
　　人民健康是民族昌盛和国家富强的重要标志，预防是最经济最有效的健康策略。党中央、国务院发布《“健康中国2030”规划纲要》，提出了健康中国建设的目标和任务。党的十九大作出实施健康中国战略的重大决策部署，强调坚持预防为主，倡导健康文明生活方式，预防控制重大疾病。为加快推动从以治病为中心转变为以人民健康为中心，动员全社会落实预防为主方针，实施健康中国行动，提高全民健康水平，现提出以下意见。
　　一、行动背景
　　新中国成立后特别是改革开放以来，我国卫生健康事业获得了长足发展，居民主要健康指标总体优于中高收入国家平均水平。随着工业化、城镇化、人口老龄化进程加快，我国居民生产生活方式和疾病谱不断发生变化。心脑血管疾病、癌症、慢性呼吸系统疾病、糖尿病等慢性非传染性疾病导致的死亡人数占总死亡人数的88%，导致的疾病负担占疾病总负担的70%以上。居民健康知识知晓率偏低，吸烟、过量饮酒、缺乏锻炼、不合理膳食等不健康生活方式比较普遍，由此引起的疾病问题日益突出。肝炎、结核病、艾滋病等重大传染病防控形势仍然严峻，精神卫生、职业健康、地方病等方面问题不容忽视。
　　为坚持预防为主，把预防摆在更加突出的位置，积极有效应对当前突出健康问题，必须关口前移，采取有效干预措施，细化落实《“健康中国2030”规划纲要》对普及健康生活、优化健康服务、建设健康环境等部署，聚焦当前和今后一段时期内影响人民健康的重大疾病和突出问题，实施疾病预防和健康促进的中长期行动，健全全社会落实预防为主的制度体系，持之以恒加以推进，努力使群众不生病、少生病，提高生活质量。 　　二、总体要求
　　（一）指导思想。以***新时代中国特色社会主义思想为指导，全面贯彻党的十九大和十九届二中、三中全会精神，坚持以人民为中心的发展思想，坚持改革创新，贯彻新时代卫生与健康工作方针，强化政府、社会、个人责任，加快推动卫生健康工作理念、服务方式从以治病为中心转变为以人民健康为中心，建立健全健康教育体系，普及健康知识，引导群众建立正确健康观，加强早期干预，形成有利于健康的生活方式、生态环境和社会环境，延长健康寿命，为全方位全周期保障人民健康、建设健康中国奠定坚实基础。
　　（二）基本原则。
　　普及知识、提升素养。把提升健康素养作为增进全民健康的前提，根据不同人群特点有针对性地加强健康教育与促进，让健康知识、行为和技能成为全民普遍具备的素质和能力，实现健康素养人人有。
　　自主自律、健康生活。倡导每个人是自己健康第一责任人的理念，激发居民热爱健康、追求健康的热情，养成符合自身和家庭特点的健康生活方式，合理膳食、科学运动、戒烟限酒、心理平衡，实现健康生活少生病。
　　早期干预、完善服务。对主要健康问题及影响因素尽早采取有效干预措施，完善防治策略，推动健康服务供给侧结构性改革，提供系统连续的预防、治疗、康复、健康促进一体化服务，加强医疗保障政策与健康服务的衔接，实现早诊早治早康复。
　　全民参与、共建共享。强化跨部门协作，鼓励和引导单位、社区（村）、家庭和个人行动起来，形成政府积极主导、社会广泛动员、人人尽责尽力的良好局面，实现健康中国行动齐参与。
　　（三）总体目标。
　　到2022年，健康促进政策体系基本建立，全民健康素养水平稳步提高，健康生活方式加快推广，重大慢性病发病率上升趋势得到遏制，重点传染病、严重精神障碍、地方病、职业病得到有效防控，致残和死亡风险逐步降低，重点人群健康状况显著改善。
　　到2030年，全民健康素养水平大幅提升，健康生活方式基本普及，居民主要健康影响因素得到有效控制，因重大慢性病导致的过早死亡率明显降低，人均健康预期寿命得到较大提高，居民主要健康指标水平进入高收入国家行列，健康公平基本实现。 　　三、主要任务
　　（一）全方位干预健康影响因素。
　　1.实施健康知识普及行动。维护健康需要掌握健康知识。面向家庭和个人普及预防疾病、早期发现、紧急救援、及时就医、合理用药等维护健康的知识与技能。建立并完善健康科普专家库和资源库，构建健康科普知识发布和传播机制。强化医疗卫生机构和医务人员开展健康促进与教育的激励约束。鼓励各级电台电视台和其他媒体开办优质健康科普节目。到2022年和2030年，全国居民健康素养水平分别不低于22%和30%。
　　2.实施合理膳食行动。合理膳食是健康的基础。针对一般人群、特定人群和家庭，聚焦食堂、餐厅等场所，加强营养和膳食指导。鼓励全社会参与减盐、减油、减糖，研究完善盐、油、糖包装标准。修订预包装食品营养标签通则，推进食品营养标准体系建设。实施贫困地区重点人群营养干预。到2022年和2030年，成人肥胖增长率持续减缓，5岁以下儿童生长迟缓率分别低于7%和5%。
　　3.实施全民健身行动。生命在于运动，运动需要科学。为不同人群提供针对性的运动健身方案或运动指导服务。努力打造百姓身边健身组织和“15分钟健身圈”。推进公共体育设施免费或低收费开放。推动形成体医结合的疾病管理和健康服务模式。把高校学生体质健康状况纳入对高校的考核评价。到2022年和2030年，城乡居民达到《国民体质测定标准》合格以上的人数比例分别不少于90.86%和92.17%，经常参加体育锻炼人数比例达到37%及以上和40%及以上。
　　4.实施控烟行动。吸烟严重危害人民健康。推动个人和家庭充分了解吸烟和二手烟暴露的严重危害。鼓励领导干部、医务人员和教师发挥控烟引领作用。把各级党政机关建设成无烟机关。研究利用税收、价格调节等综合手段，提高控烟成效。完善卷烟包装烟草危害警示内容和形式。到2022年和2030年，全面无烟法规保护的人口比例分别达到30%及以上和80%及以上。
　　5.实施心理健康促进行动。心理健康是健康的重要组成部分。通过心理健康教育、咨询、治疗、危机干预等方式，引导公众科学缓解压力，正确认识和应对常见精神障碍及心理行为问题。健全社会心理服务网络，加强心理健康人才培养。建立精神卫生综合管理机制，完善精神障碍社区康复服务。到2022年和2030年，居民心理健康素养水平提升到20%和30%，心理相关疾病发生的上升趋势减缓。
　　6.实施健康环境促进行动。良好的环境是健康的保障。向公众、家庭、单位（企业）普及环境与健康相关的防护和应对知识。推进大气、水、土壤污染防治。推进健康城市、健康村镇建设。建立环境与健康的调查、监测和风险评估制度。采取有效措施预防控制环境污染相关疾病、道路交通伤害、消费品质量安全事故等。到2022年和2030年，居民饮用水水质达标情况明显改善，并持续改善。
　　（二）维护全生命周期健康。
　　7.实施妇幼健康促进行动。孕产期和婴幼儿时期是生命的起点。针对婚前、孕前、孕期、儿童等阶段特点，积极引导家庭科学孕育和养育健康新生命，健全出生缺陷防治体系。加强儿童早期发展服务，完善婴幼儿照护服务和残疾儿童康复救助制度。促进生殖健康，推进农村妇女宫颈癌和乳腺癌检查。到2022年和2030年，婴儿死亡率分别控制在7.5‰及以下和5‰及以下，孕产妇死亡率分别下降到18/10万及以下和12/10万及以下。
　　8.实施中小学健康促进行动。中小学生处于成长发育的关键阶段。动员家庭、学校和社会共同维护中小学生身心健康。引导学生从小养成健康生活习惯，锻炼健康体魄，预防近视、肥胖等疾病。中小学校按规定开齐开足体育与健康课程。把学生体质健康状况纳入对学校的绩效考核，结合学生年龄特点，以多种方式对学生健康知识进行考试考查，将体育纳入高中学业水平测试。到2022年和2030年，国家学生体质健康标准达标优良率分别达到50%及以上和60%及以上，全国儿童青少年总体近视率力争每年降低0.5个百分点以上，新发近视率明显下降。
　　9.实施职业健康保护行动。劳动者依法享有职业健康保护的权利。针对不同职业人群，倡导健康工作方式，落实用人单位主体责任和政府监管责任，预防和控制职业病危害。完善职业病防治法规标准体系。鼓励用人单位开展职工健康管理。加强尘肺病等职业病救治保障。到2022年和2030年，接尘工龄不足5年的劳动者新发尘肺病报告例数占年度报告总例数的比例实现明显下降，并持续下降。
　　10.实施老年健康促进行动。老年人健康快乐是社会文明进步的重要标志。面向老年人普及膳食营养、体育锻炼、定期体检、健康管理、心理健康以及合理用药等知识。健全老年健康服务体系，完善居家和社区养老政策，推进医养结合，探索长期护理保险制度，打造老年宜居环境，实现健康老龄化。到2022年和2030年，65至74岁老年人失能发生率有所下降，65岁及以上人群老年期痴呆患病率增速下降。
　　（三）防控重大疾病。
　　11.实施心脑血管疾病防治行动。心脑血管疾病是我国居民第一位死亡原因。引导居民学习掌握心肺复苏等自救互救知识技能。对高危人群和患者开展生活方式指导。全面落实35岁以上人群首诊测血压制度，加强高血压、高血糖、血脂异常的规范管理。提高院前急救、静脉溶栓、动脉取栓等应急处置能力。到2022年和2030年，心脑血管疾病死亡率分别下降到209.7/10万及以下和190.7/10万及以下。
　　12.实施癌症防治行动。癌症严重影响人民健康。倡导积极预防癌症，推进早筛查、早诊断、早治疗，降低癌症发病率和死亡率，提高患者生存质量。有序扩大癌症筛查范围。推广应用常见癌症诊疗规范。提升中西部地区及基层癌症诊疗能力。加强癌症防治科技攻关。加快临床急需药物审评审批。到2022年和2030年，总体癌症5年生存率分别不低于43.3%和46.6%。
　　13.实施慢性呼吸系统疾病防治行动。慢性呼吸系统疾病严重影响患者生活质量。引导重点人群早期发现疾病，控制危险因素，预防疾病发生发展。探索高危人群首诊测量肺功能、40岁及以上人群体检检测肺功能。加强慢阻肺患者健康管理，提高基层医疗卫生机构肺功能检查能力。到2022年和2030年，70岁及以下人群慢性呼吸系统疾病死亡率下降到9/10万及以下和8.1/10万及以下。
　　14.实施糖尿病防治行动。我国是糖尿病患病率增长最快的国家之一。提示居民关注血糖水平，引导糖尿病前期人群科学降低发病风险，指导糖尿病患者加强健康管理，延迟或预防糖尿病的发生发展。加强对糖尿病患者和高危人群的健康管理，促进基层糖尿病及并发症筛查标准化和诊疗规范化。到2022年和2030年，糖尿病患者规范管理率分别达到60%及以上和70%及以上。
　　15.实施传染病及地方病防控行动。传染病和地方病是重大公共卫生问题。引导居民提高自我防范意识，讲究个人卫生，预防疾病。充分认识疫苗对预防疾病的重要作用。倡导高危人群在流感流行季节前接种流感疫苗。加强艾滋病、病毒性肝炎、结核病等重大传染病防控，努力控制和降低传染病流行水平。强化寄生虫病、饮水型燃煤型氟砷中毒、大骨节病、氟骨症等地方病防治，控制和消除重点地方病。到2022年和2030年，以乡（镇、街道）为单位，适龄儿童免疫规划疫苗接种率保持在90%以上。 　　四、组织实施
　　（一）加强组织领导。国家层面成立健康中国行动推进委员会，制定印发《健康中国行动（2019-2030年）》，细化上述15个专项行动的目标、指标、任务和职责分工，统筹指导各地区各相关部门加强协作，研究疾病的综合防治策略，做好监测考核。要根据医学进步和相关技术发展等情况，适时组织修订完善《健康中国行动（2019-2030年）》内容。各地区要结合实际健全领导推进工作机制，研究制定实施方案，逐项抓好任务落实。各相关部门要按照职责分工，将预防为主、防病在先融入各项政策举措中，研究具体政策措施，推动落实重点任务。
　　（二）动员各方广泛参与。凝聚全社会力量，形成健康促进的强大合力。鼓励个人和家庭积极参与健康中国行动，落实个人健康责任，养成健康生活方式。各单位特别是各学校、各社区（村）要充分挖掘和利用自身资源，积极开展健康细胞工程建设，创造健康支持性环境。鼓励企业研发生产符合健康需求的产品，增加健康产品供给，国有企业特别是中央企业要作出表率。鼓励社会捐资，依托社会力量依法成立健康中国行动基金会，形成资金来源多元化的保障机制。鼓励金融机构创新健康类产品和服务。卫生健康相关行业学会、协会和群团组织以及其他社会组织要充分发挥作用，指导、组织健康促进和健康科普工作。
　　（三）健全支撑体系。加强公共卫生体系建设和人才培养，提高疾病防治和应急处置能力。加强财政支持，强化资金统筹，优化资源配置，提高基本公共卫生服务项目、重大公共卫生服务项目资金使用的针对性和有效性。加强科技支撑，开展一批影响健康因素和疑难重症诊疗攻关重大课题研究，国家科技重大专项、重点研发计划要给予支持。完善相关法律法规体系，开展健康政策审查，保障各项任务落实和目标实现。强化信息支撑，推动部门和区域间共享健康相关信息。
　　（四）注重宣传引导。采取多种形式，强化舆论宣传，及时发布政策解读，回应社会关切。设立健康中国行动专题网站，大力宣传实施健康中国行动、促进全民健康的重大意义、目标任务和重大举措。编制群众喜闻乐见的解读材料和文艺作品，以有效方式引导群众了解和掌握必备健康知识，践行健康生活方式。加强科学引导和典型报道，增强社会的普遍认知，营造良好的社会氛围。

**国家积极应对人口老龄化中长期规划**

为积极应对人口老龄化，按照党的十九大决策部署，近日，中共中央、国务院印发了《国家积极应对人口老龄化中长期规划》（以下简称《规划》）。《规划》近期至2022年，中期至2035年，远期展望至2050年，是到本世纪中叶我国积极应对人口老龄化的战略性、综合性、指导性文件。
　　《规划》指出，人口老龄化是社会发展的重要趋势，是人类文明进步的体现，也是今后较长一段时期我国的基本国情。人口老龄化对经济运行全领域、社会建设各环节、社会文化多方面乃至国家综合实力和国际竞争力，都具有深远影响，挑战与机遇并存。
　　《规划》强调，积极应对人口老龄化，是贯彻以人民为中心的发展思想的内在要求，是实现经济高质量发展的必要保障，是维护国家安全和社会和谐稳定的重要举措。要按照经济高质量发展的要求，坚持以供给侧结构性改革为主线，构建管长远的制度框架，制定见实效的重大政策，坚持积极应对、共建共享、量力适度、创新开放的基本原则，走出一条中国特色应对人口老龄化道路。
　　《规划》明确了积极应对人口老龄化的战略目标，即积极应对人口老龄化的制度基础持续巩固，财富储备日益充沛，人力资本不断提升，科技支撑更加有力，产品和服务丰富优质，社会环境宜居友好，经济社会发展始终与人口老龄化进程相适应，顺利建成社会主义现代化强国，实现中华民族伟大复兴的中国梦。到2022年，我国积极应对人口老龄化的制度框架初步建立；到2035年，积极应对人口老龄化的制度安排更加科学有效；到本世纪中叶，与社会主义现代化强国相适应的应对人口老龄化制度安排成熟完备。
　　《规划》从5个方面部署了应对人口老龄化的具体工作任务。
　　一是夯实应对人口老龄化的社会财富储备。通过扩大总量、优化结构、提高效益，实现经济发展与人口老龄化相适应。通过完善国民收入分配体系，优化政府、企业、居民之间的分配格局，稳步增加养老财富储备。健全更加公平更可持续的社会保障制度，持续增进全体人民的福祉水平。
　　二是改善人口老龄化背景下的劳动力有效供给。通过提高出生人口素质、提升新增劳动力质量、构建老有所学的终身学习体系，提高我国人力资源整体素质。推进人力资源开发利用，实现更高质量和更加充分就业，确保积极应对人口老龄化的人力资源总量足、素质高。
　　三是打造高质量的为老服务和产品供给体系。积极推进健康中国建设，建立和完善包括健康教育、预防保健、疾病诊治、康复护理、长期照护、安宁疗护的综合、连续的老年健康服务体系。健全以居家为基础、社区为依托、机构充分发展、医养有机结合的多层次养老服务体系，多渠道、多领域扩大适老产品和服务供给，提升产品和服务质量。
　　四是强化应对人口老龄化的科技创新能力。深入实施创新驱动发展战略，把技术创新作为积极应对人口老龄化的第一动力和战略支撑，全面提升国民经济产业体系智能化水平。提高老年服务科技化、信息化水平，加大老年健康科技支撑力度，加强老年辅助技术研发和应用。
　　五是构建养老、孝老、敬老的社会环境。强化应对人口老龄化的法治环境，保障老年人合法权益。构建家庭支持体系，建设老年友好型社会，形成老年人、家庭、社会、政府共同参与的良好氛围。
　　《规划》要求，坚持党对积极应对人口老龄化工作的领导，坚持党政主要负责人亲自抓、负总责，强化各级政府落实规划的主体责任，进一步完善组织协调机制。推进国际合作，推动与“一带一路”相关国家开展应对人口老龄化的政策对话和项目对接。选择有特点和代表性的区域进行应对人口老龄化工作综合创新试点。建立健全工作机制、实施监管和考核问责制度，强化对规划实施的监督，确保规划落实。

**教育部关于印发《高校银龄教师支援西部计划实施方案》的通知**教师函〔2020〕1号

为深入贯彻落实习近平新时代中国特色社会主义思想和党的十九大精神，根据中共中央、国务院关于全面深化新时代教师队伍建设改革和新时代推进西部大开发形成新格局的有关精神，按照《国务院办公厅关于加快中西部教育发展的指导意见》等文件要求，进一步加强西部高校教师队伍建设，推动东部地区高校对口支援西部地区高校工作，充分利用高校退休教师优势资源，调动高校优秀退休教师继续投身教育事业的积极性，推动西部高等教育振兴发展，现就实施高校银龄教师支援西部计划，制定如下方案。

**一、目标任务**

面向西部地区行业、产业、企业急需的紧缺专业，遴选组织一批高校优秀退休教师支教、支研，发挥高校优秀退休教师的政治优势、经验优势和专业优势，帮助提升西部高校立德树人、队伍建设和科研创新的能力，推动西部地区高校“双一流”建设，缓解西部地区高校师资总量不足和结构不合理等矛盾，提升西部高等教育发展水平。

**二、工作要求**

（一）实施范围。2020年，首批试点高校为中国石油大学（北京）克拉玛依校区、塔里木大学、滇西应用技术大学，首批试点对口支援关系表详见附件1，后期根据实施效果和地方需求确定试点范围。同时，鼓励对口支援关系表外的各部属高校积极支持本校优秀退休教师参加本计划。

（二）遴选人数。2020年，计划遴选120至140名教师，后期根据试点情况确定遴选规模。

（三）资格条件。一般从教育部直属高校退休人员中遴选援派教师，申请教师年龄一般在70（含）岁以下（身体情况较好者可适当放宽年龄要求），具有副高级以上职称，一线教学科研经验丰富；政治可靠、师德高尚、爱岗敬业、业务精良；身体健康、甘于奉献、不怕吃苦、作风扎实。

（四）岗位职责

1. 以课程教学、教学指导、课题研究、团队建设指导为主，短期授课、远程教育、同步课堂、学术讲座（报告）为辅，采取传、帮、带的方式，指导受援高校教师做好教学和科研工作，把先进教学方法和科研理念传授给受援高校教师。

2. 长期援派教师，支援服务时间原则上不少于1学年，每学年承担不少于64课时的教学工作，参与指导1项课题研究，通过传、帮、带方式指导青年教师，组织开展若干学术讲座、教研等活动。鼓励考核合格的长期援派教师连续支援服务。

3. 短期和远程支援教师，按照“突出实效、形式多样、时间灵活”的原则，根据受援高校需求，认真做好支教、支研工作。

**三、保障措施**

（一）经费保障

1. 长期援派教师税前补助标准为，副高级职称教师8万元/年、正高级职称教师10万元/年，按月发放。受援高校根据实际情况，可适当提高补助标准，高出部分由受援高校自行负担。

2. 短期援派教师税前补助标准为，副高级职称教师6400元/月、正高级职称教师8000元/月。

3. 通过远程教育、同步课堂、远程传帮带方式援教，课时费为100元/课时。

4. 到校开展学术讲座、报告等的劳务费或咨询费参照受援高校相关标准执行。

5. 受援高校提供岗前培训费、派出地往返交通差旅费、意外保险费、必要的商业医疗保险费、采暖费等保障性经费。援派高校提供派出前体检费、遴选工作经费等。

（二）政策保障

1. 教师援派期间人事关系、现享受的退休待遇不变。长期援派教师，享受受援单位同类同级别人员的各项福利待遇，按受援地有关规定正常享受探亲和寒暑假。未休假的，按有关规定可由受援单位报销一名家属往返受援地的交通费。

2. 受援高校应为援派教师创造良好的工作环境，提供必要的教学科研设备和生活条件，做好日常服务工作，落实相关待遇与保障措施。

3. 援派高校要关心关注援派教师及其家属，全力解决他们工作和生活中的困难；负责提供远程授课设施设备，协助受援高校做好后勤保障工作。

4. 援派期间因病因伤发生医疗费用，按本人医疗关系和有关规定办理；援受双方可通过商业医疗保险、校内医疗互助基金等多种方式灵活提供补充支持。患有慢性疾病需定期开药的教师，援派高校应为其提供必要的便利。援派期间生病或受伤的教师，参照受援学校相关制度，给予探望慰问等待遇。

**四、组织实施**

1. 教育部教师工作司、发展规划司、财务司、民族教育司、离退休干部局、学校规划建设发展中心等司局及直属事业单位结合职能分工，做好指导协调和跟踪督查工作，共同组织实施高校银龄教师支援西部计划。

2. 受援高校提出需求，制订具体工作方案，指定专门机构和人员负责本校方案实施和协调管理工作，制订服务协议，明确权利和义务，规范资金使用，落实待遇保障措施。实施方案报教育部备案。

3. 援派高校党委应高度重视，将本项目工作纳入学校援派工作体系，统筹考虑，统一部署，成立领导小组，相关职能部门结合自身分工共同实施。领导小组名单（附件2）应及时向教育部教师工作司报备。

4. 援派教师遴选程序如下：（1）汇总受援高校需求；（2）组织动员退休教师按需求报名；（3）资格审核或遴选；（4）公示公布；（5）协助签订援派高校、受援高校、援派教师三方协议；（6）上岗任教。首批援派教师于2020年春季学期到位。

5. 援受双方高校应做好政策宣传，弘扬教师奉献精神，营造良好的工作氛围。后期要及时收集积累相关信息资料，深入挖掘援派教师中的先进典型和感人事迹。

各省、自治区、直辖市及新疆生产建设兵团卫生健康委（老龄办）：
　　为深入贯彻落实党中央关于抓紧抓实抓细常态化疫情防控工作的决策部署，按照《[国务院应对新型冠状病毒感染肺炎疫情联防联控机制关于做好新冠肺炎疫情常态化防控工作的指导意见](javascript:SLC(342057))》要求，现就在常态化疫情防控中做好老年人照顾服务工作，提出以下要求：
　　一、充分认识在常态化疫情防控中做好老年人照顾服务工作的重要性
　　党中央高度重视老年人照顾服务工作。当前，在常态化疫情防控中，广大老年人特别是独居、高龄、失能等特殊困难老年人，在生活、就医、照护、心理健康等方面面临一系列实际困难和问题。各地要充分认识常态化疫情防控中做好老年人照顾服务工作的重要性，在全面落实疫情防控措施的同时，高度关注广大老年人的现实需求，及时帮助有需要的老年人解决生活中遇到的实际困难和问题，坚决防止冲击社会道德底线的事情发生，切实保障老年人生命安全和身体健康。 　　二、做好老年人健康服务
　　各地要创新工作手段，强化工作措施，做好常态化疫情防控中老年人健康服务。各级医疗卫生机构要及时公布医疗服务恢复情况，通过网络、电话预约等形式，减少老年人排队等候时间，确保有需求的老年人及时、便利就诊。积极开展“互联网＋老年健康”服务，利用线上咨询等方式，为老年人提供健康指导和服务。
　　要加强家庭医生签约服务工作，运用微信、手机APP等信息化手段，建立家庭医生与老年人及其照护者之间的互动沟通渠道。开展家庭医生随访服务，及时掌握老年人健康状况，督促慢性病老年患者加强血压、血糖自我监测，并进行针对性指导。采取慢性病长期处方等用药措施，加强老年人用药指导。对于出现发热等症状的老年人，要及时送到发热门诊就诊。根据本地区疫情防控形势，适时恢复开展基本公共卫生服务老年人健康管理项目，加强健康评估和指导。
　　要全面掌握辖区内独居、高龄、失能及患有多种慢性疾病的老年人信息，关注健康状况，做好健康管理。对辖区内失能老年人，要开展上门健康评估和服务。要针对老年人特点，做好老年新冠肺炎患者的康复服务和心理疏导工作。

　三、做好社区居家老年人照顾服务
　　针对社区内独居、高龄、失能等特殊困难老年人及计划生育特殊家庭的老年人，各地要积极指导协调各级民政、卫生健康等部门根据职责，推动乡镇政府（街道办事处）落实属地责任，建立落实走访探视和关爱服务制度，积极帮助他们解决在生活照料、看病就医等方面的困难。要适应常态化防控要求，有序放开养老护理员、社区养老服务机构人员上门服务，允许家政、维修、快递等服务人员进入社区，既“放权”又压实责任，落实好相应的防控措施，满足老年人的生活需求。
　　要以老年人喜闻乐见的方式，大力宣传防控知识和信息，指导老年人持续做好自身防护，保持良好的卫生习惯和健康生活方式，自觉服从社区防控管理。要指导老年人在疫情防控常态化情况下，科学佩戴口罩，减少人员聚集，加强室内通风消毒，提高健康素养，养成“一米线”、勤洗手、戴口罩、公筷制等卫生习惯和生活方式，错峰乘坐公共交通工具。指导老年人根据自身条件及居家环境，进行适宜的体育锻炼，提升主动健康意识和健康水平。
　　要充分发挥社区老年社会组织、老年志愿者的作用，鼓励其根据防控要求和自身条件，适度参与社区防控和社区服务工作，为疫情防控作出应有贡献。老年活动场所和社区为老服务场所，要按照当地疫情防控要求落实好常态化防控措施，根据疫情形势逐步有序恢复为老年人提供服务。

四、做好入住机构老年人照顾服务
　　各地要指导养老机构、医养结合机构进一步完善常态化疫情防控工作机制，强化责任落实，保障防疫物资供应，切实消除感染风险隐患，保障入住老年人生命安全和身体健康。养老机构、医养结合机构要按照国务院应对新冠肺炎疫情联防联控机制关于养老机构新冠肺炎疫情防控相关要求，在保证入住老年人生命安全和身体健康前提下，按照所在地区风险等级及时调整疫情防控策略，有序恢复正常服务。要落实人员进出管理、人员防护、健康监测、消毒等防控措施。重视加强老年人心理疏导，鼓励子女家人通过互联网等信息化手段与老年人亲情互动。
　　养老机构、医养结合机构要在本地联防联控机制的统一部署下，加强对无症状感染者的筛查检测工作，积极配合开展核酸检测筛查和流行病学调查。一旦发现无症状感染者，要立即上报并按要求进行集中隔离和医学管理；隔离期间若出现症状，要立即转运至定点医疗机构进行救治。
　　医养结合机构要按照本地工作部署，有序恢复正常医疗卫生服务，为老年人提供便捷、适宜的医疗卫生服务。内设医务室、护理站等医疗服务机构不得超出医疗许可服务范围对外服务。开展对外诊疗服务的医养结合机构，要完善门急诊预检分诊管理；设置发热门诊的，要加强发热门诊管理和感染防控，做好医务人员和入住老年人防护工作；有条件的机构，可开展分时段预约诊疗和互联网诊疗咨询。医疗机构举办养老机构或与养老机构毗邻的，应按照医疗机构分区管理要求开展交叉感染评估，评估有风险的应采取必要的控制措施。医疗卫生机构要在地方政府的领导和统筹安排下，指导签约合作的养老机构落实疫情防控和传染病防治措施，增强防控意识，提高防控能力，严防机构内感染。

五、强化对老年人的兜底民生保障
　　扎实做好老年人各项民生保障工作，加强各项政策配套衔接，切实保障老年人的基本生活、基本医疗。对符合条件的老年人及时足额发放护理补贴、养老服务补贴及高龄老年人津贴。对符合低保条件的老年人家庭要及时纳入，做到应保尽保。对符合条件的特殊困难老年人，要及时给予临时救助。扎实推进健康扶贫，针对贫困人口中的患病、失能老年人，要制定“一人一策”精准化帮扶措施，有条件的地方可实行集中救治、兜底保障，确保农村贫困老年人全部如期脱贫，同步进入小康社会。

六、加大对为老服务机构的扶持力度
　　各地要开展疫情对本地区为老服务发展影响的研究分析，及时将养老服务机构、医养结合机构等为老服务机构纳入扶持保障范围。全面落实好阶段性减免企业社保费、医保费和缓缴住房公积金、临时降低用气用水用电价格等国家及本地区出台的优惠扶持政策。要结合地方和机构实际，在落实落细已出台扶持政策措施的同时，采取针对性、差异化扶持措施，如提前拨付补助资金、提高补贴标准、减免租金、发放岗位临时补贴等，确保各类为老服务机构适应常态化防控要求，平稳有序运营服务。
　　各地要统筹做好疫情防控和老年人照顾服务工作，各级卫生健康行政部门（老龄办）要加强对老年人疫情防控和照顾服务工作的督导检查，对发现的问题要及时督促整改，确保老年人生命安全和身体健康

三、活动内容
　　（一）宣传老年健康政策。宣传《健康中国行动（2019-2030年）》老年健康促进行动、《[关于建立完善老年健康服务体系的指导意见](javascript:SLC(337092))》政策要求，宣传常态化疫情防控中对老年人的照顾服务内容，宣传老年人健康管理、老年健康与医养结合、高血压患者健康管理、糖尿病患者健康管理等国家基本公共卫生服务政策，宣传各地老年健康服务政策。
　　（二）宣传老年健康知识。结合老年人特点，围绕常态化疫情防控、营养膳食、运动健身、心理健康、伤害预防、疾病预防、合理用药、康复护理、生命教育和中医养生保健等，宣传老年健康科学知识，重点宣传《中国公民健康素养-基本知识与技能》《老年健康核心信息》《老年失能预防核心信息》《阿尔茨海默病预防与干预核心信息》等。

四、工作要求
　　（一）各级卫生健康行政部门要高度重视，精心组织。统筹推进常态化疫情防控和老年健康宣传教育工作，在做好新冠肺炎疫情防控工作的同时，创造性地开展2020年老年健康宣传周活动。我委将制作老年健康教育系列科普视频，并在国家卫生健康委网站老龄健康司子网站发布，供各地参考使用。
　　（二）各地要充分发挥传统媒体和网络、微博、微信等新媒体的优势，面向老年人及其照护者，通俗易懂地宣传相关政策和知识。根据新冠肺炎疫情防控要求，通过线上活动，采取趣味答题、在线问答、专家解读等方式，组织开展老年人喜闻乐见的宣传活动。在落实国家基本公共卫生服务和家庭医生签约服务过程中，开展个性化的宣传教育，实现送政策、送知识、送服务“三统一”。
　　（三）请各省级卫生健康行政部门做好工作总结，反映活动亮点、群众感受、发现的问题和工作建议等，于2020年9月15日前将活动开展情况总结报告报送我委老龄司。鼓励各地报送宣传资料、视频等，我委将择优在官方网站展播。

关于切实解决老年人运用智能技术困难的实施方案

　　随着我国互联网、大数据、人工智能等信息技术快速发展，智能化服务得到广泛应用，深刻改变了生产生活方式，提高了社会治理和服务效能。但同时，我国老龄人口数量快速增长，不少老年人不会上网、不会使用智能手机，在出行、就医、消费等日常生活中遇到不便，无法充分享受智能化服务带来的便利，老年人面临的“数字鸿沟”问题日益凸显。为进一步推动解决老年人在运用智能技术方面遇到的困难，让老年人更好共享信息化发展成果，制定本实施方案。
　　一、总体要求
　　（一）指导思想。
　　以习近平新时代中国特色社会主义思想为指导，全面贯彻党的十九大和十九届二中、三中、四中、五中全会精神，认真落实党中央、国务院决策部署，坚持以人民为中心的发展思想，满足人民日益增长的美好生活需要，持续推动充分兼顾老年人需要的智慧社会建设，坚持传统服务方式与智能化服务创新并行，切实解决老年人在运用智能技术方面遇到的困难。要适应统筹推进疫情防控和经济社会发展工作要求，聚焦老年人日常生活涉及的高频事项，做实做细为老年人服务的各项工作，增进包括老年人在内的全体人民福祉，让老年人在信息化发展中有更多获得感、幸福感、安全感。
　　（二）基本原则。
　　——坚持传统服务与智能创新相结合。在各类日常生活场景中，必须保留老年人熟悉的传统服务方式，充分保障在运用智能技术方面遇到困难的老年人的基本需求；紧贴老年人需求特点，加强技术创新，提供更多智能化适老产品和服务，促进智能技术有效推广应用，让老年人能用、会用、敢用、想用。坚持“两条腿”走路，使智能化管理适应老年人，并不断改进传统服务方式，为老年人提供更周全、更贴心、更直接的便利化服务。
　　——坚持普遍适用与分类推进相结合。强化问题导向和需求导向，针对老年人在运用智能技术方面遇到的突出共性问题，采取普遍适用的政策措施；对不同年龄段、不同教育背景、不同生活环境和习惯的老年人，分类梳理问题，采取有针对性、差异化的解决方案。
　　——坚持线上服务与线下渠道相结合。线上服务更加突出人性化，充分考虑老年人习惯，便利老年人使用；线下渠道进一步优化流程、简化手续，不断改善老年人服务体验，与线上服务融合发展、互为补充，有效发挥兜底保障作用。
　　——坚持解决突出问题与形成长效机制相结合。围绕老年人出行、就医等高频事项和服务场景，抓紧解决目前最突出、最紧迫的问题，切实保障老年人基本服务需要；在此基础上，逐步总结积累经验，不断提升智能化服务水平，完善服务保障措施，建立长效机制，有效解决老年人面临的“数字鸿沟”问题。
　　（三）工作目标。
　　在政策引导和全社会的共同努力下，有效解决老年人在运用智能技术方面遇到的困难，让广大老年人更好地适应并融入智慧社会。到2020年底前，集中力量推动各项传统服务兜底保障到位，抓紧出台实施一批解决老年人运用智能技术最迫切问题的有效措施，切实满足老年人基本生活需要。到2021年底前，围绕老年人出行、就医、消费、文娱、办事等高频事项和服务场景，推动老年人享受智能化服务更加普遍，传统服务方式更加完善。到2022年底前，老年人享受智能化服务水平显著提升、便捷性不断提高，线上线下服务更加高效协同，解决老年人面临的“数字鸿沟”问题的长效机制基本建立。

二、重点任务
　　（一）做好突发事件应急响应状态下对老年人的服务保障。
　　1．完善“健康码”管理，便利老年人通行。在新冠肺炎疫情低风险地区，除机场、铁路车站、长途客运站、码头和出入境口岸等特殊场所外，一般不用查验“健康码”。对需查验“健康码”的情形，通过技术手段将疫情防控相关信息自动整合到“健康码”，简化操作以适合老年人使用，优化代办代查等服务，继续推行“健康码”全国互通互认，便利老年人跨省通行。各地不得将“健康码”作为人员通行的唯一凭证，对老年人等群体可采取凭有效身份证件登记、持纸质证明通行、出示“通信行程卡”作为辅助行程证明等替代措施。有条件的地区和场所要为不使用智能手机的老年人设立“无健康码通道”，做好服务引导和健康核验。在充分保障个人信息安全前提下，推进“健康码”与身份证、社保卡、老年卡、市民卡等互相关联，逐步实现“刷卡”或“刷脸”通行。对因“健康码”管理不当造成恶劣影响的，根据有关规定追究相关单位负责人的责任。

　　2．保障居家老年人基本服务需要。在常态化疫情防控下，为有效解决老年人无法使用智能技术获取线上服务的困难，组织、引导、便利城乡社区组织、机构和各类社会力量进社区、进家庭，建设改造一批社区便民消费服务中心、老年服务站等设施，为居家老年人特别是高龄、空巢、失能、留守等重点群体，提供生活用品代购、餐饮外卖、家政预约、代收代缴、挂号取药、上门巡诊、精神慰藉等服务，满足基本生活需求。
　　3．在突发事件处置中做好帮助老年人应对工作。在自然灾害、事故灾难、公共卫生事件、社会安全事件等突发事件处置中，需采取必要智能化管理和服务措施的，要在应急预案中统筹考虑老年人需要，提供突发事件风险提醒、紧急避难场所提示、“一键呼叫”应急救援、受灾人群转移安置、救灾物资分配发放等线上线下相结合的应急救援和保障服务，切实解决在应急处置状态下老年人遇到的困难。

　　（二）便利老年人日常交通出行。
　　4．优化老年人打车出行服务。保持巡游出租车扬召服务，对电召服务要提高电话接线率。引导网约车平台公司优化约车软件，增设“一键叫车”功能，鼓励提供电召服务，对老年人订单优先派车。鼓励有条件的地区在医院、居民集中居住区、重要商业区等场所设置出租车候客点、临时停靠点，依托信息化技术提供便捷叫车服务。
　　5．便利老年人乘坐公共交通。铁路、公路、水运、民航客运等公共交通在推行移动支付、电子客票、扫码乘车的同时，保留使用现金、纸质票据、凭证、证件等乘车的方式。推进交通一卡通全国互通与便捷应用，支持具备条件的社保卡增加交通出行功能，鼓励有条件的地区推行老年人凭身份证、社保卡、老年卡等证件乘坐城市公共交通。

　　6．提高客运场站人工服务质量。进一步优化铁路、公路、水运、民航客运场站及轨道交通站点等窗口服务，方便老年人现场购票、打印票证等。高速公路服务区、收费站等服务窗口要为老年人提供咨询、指引等便利化服务和帮助。　　（三）便利老年人日常就医。
　　7．提供多渠道挂号等就诊服务。医疗机构、相关企业要完善电话、网络、现场等多种预约挂号方式，畅通家人、亲友、家庭签约医生等代老年人预约挂号的渠道。医疗机构应提供一定比例的现场号源，保留挂号、缴费、打印检验报告等人工服务窗口，配备导医、志愿者、社会工作者等人员，为老年人提供就医指导服务。
　　8．优化老年人网上办理就医服务。简化网上办理就医服务流程，为老年人提供语音引导、人工咨询等服务，逐步实现网上就医服务与医疗机构自助挂号、取号叫号、缴费、打印检验报告、取药等智能终端设备的信息联通，促进线上线下服务结合。推动通过身份证、社保卡、医保电子凭证等多介质办理就医服务，鼓励在就医场景中应用人脸识别等技术。
　　9．完善老年人日常健康管理服务。搭建社区、家庭健康服务平台，由家庭签约医生、家人和有关市场主体等共同帮助老年人获得健康监测、咨询指导、药品配送等服务，满足居家老年人的健康需求。推进“互联网＋医疗健康”，提供老年人常见病、慢性病复诊以及随访管理等服务。
　　（四）便利老年人日常消费。
　　10．保留传统金融服务方式。任何单位和个人不得以格式条款、通知、声明、告示等方式拒收现金。要改善服务人员的面对面服务，零售、餐饮、商场、公园等老年人高频消费场所，水电气费等基本公共服务费用、行政事业性费用缴纳，应支持现金和银行卡支付。强化支付市场监管，加大对拒收现金、拒绝银行卡支付等歧视行为的整改整治力度。采用无人销售方式经营的场所应以适当方式满足消费者现金支付需求，提供现金支付渠道或转换手段。
　　11．提升网络消费便利化水平。完善金融科技标准规则体系，推动金融机构、非银行支付机构、网络购物平台等优化用户注册、银行卡绑定和支付流程，打造大字版、语音版、民族语言版、简洁版等适老手机银行APP，提升手机银行产品的易用性和安全性，便利老年人进行网上购物、订餐、家政、生活缴费等日常消费。平台企业要提供技术措施，保障老年人网上支付安全。
　　（五）便利老年人文体活动。
　　12．提高文体场所服务适老化程度。需要提前预约的公园、体育健身场馆、旅游景区、文化馆、图书馆、博物馆、美术馆等场所，应保留人工窗口和电话专线，为老年人保留一定数量的线下免预约进入或购票名额。同时，在老年人进入文体场馆和旅游景区、获取电子讲解、参与全民健身赛事活动、使用智能健身器械等方面，提供必要的信息引导、人工帮扶等服务。
　　13．丰富老年人参加文体活动的智能化渠道。引导公共文化体育机构、文体和旅游类企业提供更多适老化智能产品和服务，同时开展丰富的传统文体活动。针对广场舞、群众歌咏等方面的普遍文化需求，开发设计适老智能应用，为老年人社交娱乐提供便利。探索通过虚拟现实、增强现实等技术，帮助老年人便捷享受在线游览、观赛观展、体感健身等智能化服务。
　　（六）便利老年人办事服务。
　　14．优化“互联网＋政务服务”应用。依托全国一体化政务服务平台，进一步推进政务数据共享，优化政务服务，实现社会保险待遇资格认证、津贴补贴领取等老年人高频服务事项便捷办理，让老年人办事少跑腿。各级政务服务平台应具备授权代理、亲友代办等功能，方便不使用或不会操作智能手机的老年人网上办事。
　　15．设置必要的线下办事渠道。医疗、社保、民政、金融、电信、邮政、信访、出入境、生活缴费等高频服务事项，应保留线下办理渠道，并向基层延伸，为老年人提供便捷服务。实体办事大厅和社区综合服务设施应合理布局，配备引导人员，设置现场接待窗口，优先接待老年人，推广“一站式”服务，进一步改善老年人办事体验。
　　（七）便利老年人使用智能化产品和服务应用。
　　16．扩大适老化智能终端产品供给。推动手机等智能终端产品适老化改造，使其具备大屏幕、大字体、大音量、大电池容量、操作简单等更多方便老年人使用的特点。积极开发智能辅具、智能家居和健康监测、养老照护等智能化终端产品。发布智慧健康养老产品及服务推广目录，开展应用试点示范，按照适老化要求推动智能终端持续优化升级。建设智慧健康养老终端设备的标准及检测公共服务平台，提升适老产品设计、研发、检测、认证能力。　　17．推进互联网应用适老化改造。组织开展互联网网站、移动互联网应用改造专项行动，重点推动与老年人日常生活密切相关的政务服务、社区服务、新闻媒体、社交通讯、生活购物、金融服务等互联网网站、移动互联网应用适老化改造，使其更便于老年人获取信息和服务。优化界面交互、内容朗读、操作提示、语音辅助等功能，鼓励企业提供相关应用的“关怀模式”、“长辈模式”，将无障碍改造纳入日常更新维护。
　　18．为老年人提供更优质的电信服务。持续开展电信普遍服务试点，推进行政村移动网络深度覆盖，加强偏远地区养老服务机构、老年活动中心等宽带网络覆盖。开展精准降费，引导基础电信企业为老年人提供更大力度的资费优惠，合理降低使用手机、宽带网络等服务费用，推出更多老年人用得起的电信服务。
　　19．加强应用培训。针对老年人在日常生活中的应用困难，组织行业培训机构和专家开展专题培训，提高老年人对智能化应用的操作能力。鼓励亲友、村（居）委会、老年协会、志愿者等为老年人运用智能化产品提供相应帮助。引导厂商针对老年人常用的产品功能，设计制作专门的简易使用手册和视频教程。　　20．开展老年人智能技术教育。将加强老年人运用智能技术能力列为老年教育的重点内容，通过体验学习、尝试应用、经验交流、互助帮扶等，引导老年人了解新事物、体验新科技，积极融入智慧社会。推动各类教育机构针对老年人研发全媒体课程体系，通过老年大学（学校）、养老服务机构、社区教育机构等，采取线上线下相结合的方式，帮助老年人提高运用智能技术的能力和水平。

三、保障措施
　　（一）健全工作机制。建立国家发展改革委、国家卫生健康委牵头，国务院各有关部门参加的部际联席会议机制，明确责任分工，加强统筹推进。各地区要建立相应的协调推进机制，细化措施，确保任务落实到位。各地区、各部门要加强工作协同和信息共享，形成统筹推进、分工负责、上下联动的工作格局，加快建立解决老年人面临“数字鸿沟”问题的长效机制。
　　（二）完善法规规范。加快推动制修订涉及现金支付、消费者权益保护、防止诈骗、无障碍改造等相关法律法规和部门规章，切实保障老年人使用智能技术过程中的各项合法权益。各地区要围绕出行、就医、消费、办事等老年人日常生活需求，推动相关地方性法规制修订工作。加快推进相关智能产品与服务标准规范制修订工作，进一步明确有关适老化的内容。
　　（三）加强督促落实。各地区、各部门要明确时间表、路线图，建立工作台账，强化工作落实，及时跟踪分析涉及本地区、本部门的相关政策措施实施进展及成效，确保各项工作措施做实做细、落实到位。要定期组织开展第三方评估，对各地区公共服务适老化程度进行评价，相关结果纳入积极应对人口老龄化综合评估。（国家发展改革委、国家卫生健康委牵头，相关部门及各地区按职责分工负责）
　　（四）保障信息安全。规范智能化产品和服务中的个人信息收集、使用等活动，综合运用多种安全防护手段和风险控制措施，加强技术监测和监督检查，及时曝光并处置违法违规获取个人信息等行为。实施常态化综合监管，加强与媒体等社会力量合作，充分依托各类举报投诉热线，严厉打击电信网络诈骗等违法行为，切实保障老年人安全使用智能化产品、享受智能化服务。

　　（五）开展普及宣传。将促进老年人融入智慧社会作为人口老龄化国情教育重点，加强正面宣传和舆论监督，弘扬尊重和关爱老年人的社会风尚。开展智慧助老行动，将解决老年人运用智能技术困难相关工作，纳入老年友好城市、老年友好社区、老年宜居环境等建设中统筹推进。对各地区有益做法、典型案例及时进行宣传报道，组织开展经验交流。

**国家卫生健康委、全国老龄办关于开展示范性全国老年友好型社区创建工作的通知**

一、工作目标
　　提升社区服务能力和水平，更好地满足老年人在居住环境、日常出行、健康服务、养老服务、社会参与、精神文化生活等方面的需要，探索建立老年友好型社区创建工作模式和长效机制，切实增强老年人的获得感、幸福感、安全感。到2025年，在全国建成5000个示范性城乡老年友好型社区，到2035年，全国城乡实现老年友好型社区全覆盖。

二、工作任务
　　（一）改善老年人的居住环境。支持对老年人住房的空间布局、地面、扶手、厨房设备、如厕洗浴设备、紧急呼叫设备等进行适老化改造、维修和配备，降低老年人生活风险。建立社区防火和紧急救援网络，完善老年人住宅防火和紧急救援救助功能。定期开展独居、空巢、留守、失能（含失智）、重残、计划生育特殊家庭老年人家庭用水、用电和用气等设施安全检查，对老化或损坏的设施及时进行改造维修，排除安全隐患。加强社区生态环境建设，大力绿化和美化社区，营造卫生清洁、空气清新的社区环境。
　　（二）方便老年人的日常出行。加强老年人住宅公共设施无障碍改造，重点对坡道、楼梯、电梯、扶手等进行改造，保障老年人出行安全。加强社区道路设施、休憩设施、信息化设施、服务设施等与老年人日常生活密切相关的设施和场所的无障碍建设。新建城乡社区提倡人车分流模式，加强步行系统安全设计和空间节点标志性设计。
　　（三）提升为老年人服务的质量。利用社区卫生服务中心（站）、乡镇卫生院等定期为老年人提供生活方式和健康状况评估、体格检查、辅助检查和健康指导等健康管理服务，为患病老年人提供基本医疗、康复护理、长期照护、安宁疗护等服务。开展老年人群营养状况监测和评价，制定满足不同老年人群营养需求的改善措施。深入推进医养结合，支持社区卫生服务机构、乡镇卫生院内部建设医养结合中心，为老年人提供多种形式的健康养老服务。利用社区日间照料中心及社会化资源为老年人提供生活照料、助餐助浴助洁、紧急救援、康复辅具租赁、精神慰藉、康复指导等多样化养老服务。广泛开展以老年人识骗、防骗为主要内容的宣传教育活动。建立定期巡访独居、空巢、留守、失能（含失智）、重残、计划生育特殊家庭老年人等的工作机制。
　　（四）扩大老年人的社会参与。引导和组织老年人参与社区建设和管理活动，参与社区公益慈善、教科文卫等事业，支持社区老年人广泛开展自助、互助和志愿活动，充分发挥老年人的积极作用。因地制宜改造或修建综合性活动场所，配建有利于各年龄群体共同活动的健身和文化设施，为老年人和老年社会组织参与社区活动提供必要的场地、设施和经费保障，满足老年人社会参与需求。
　　（五）丰富老年人的精神文化生活。鼓励社区自设老年教育学习点或与老年大学、教育机构和社会组织等合作在社区设立老年教育学习点，方便老年人就近学习。有效整合乡村教育文化资源，发展农村社区的老年教育，以村民喜爱的形式开展适应老年人需求的教育活动。丰富老年教育内容和手段，积极开展老年人思想道德、科学普及、休闲娱乐、健康知识、艺术审美、智能生活、法律法规、家庭理财、代际沟通、生命尊严等方面的教育。鼓励老年人自主学习，支持建立不同类型的学习团队。组织多种形式的社区敬老爱老助老主题教育活动，加大对“敬老文明号”和“敬老爱老助老模范人物”的宣传。开展有利于促进代际互动、邻里互助的社区活动，增强不同代际间的文化融合和社会认同。
　　（六）提高为老服务的科技化水平。提高社区为老服务信息化水平，利用社区综合服务平台，有效对接服务供给与需求信息，加强健康养老终端设备的适老化设计与开发，为老年人提供方便的智慧健康养老服务。依托智慧网络平台和相关智能设备，为老年人的居家照护、医疗诊断、健康管理等提供远程服务及辅助技术服务。开展“智慧助老”行动，依托社区加大对老年人智能技术使用的宣教和培训，并为老年人在其高频活动场所保留必要的传统服务方式。 　　三、工作安排
　　第一阶段：示范创建阶段（2020-2022年）。2020年，启动老年友好型社区创建工作。2021-2022年，在全国创建2000个示范性城乡老年友好型社区，为全国发挥示范引领作用。
　　第二阶段：示范推进阶段（2023-2025年）。进一步推进示范性城乡老年友好型社区创建，2023-2025年，在全国再创建3000个示范性城乡老年友好型社区。
　　第三阶段：总结深化阶段（2026-2030年）。认真总结示范性城乡老年友好型社区创建的工作经验和工作模式，加强工作宣传，扩大创建范围，开展中期评估，到2030年底，老年友好型社区在全国城乡社区的覆盖率达到50%以上。
　　第四阶段：全面评估阶段（2031-2035年）。大力推广老年友好型社区创建经验和工作机制，评估创建效果，加强分类指导，进一步扩大城乡老年友好型社区创建的覆盖面，到2035年底，全国城乡社区普遍达到老年友好型社区标准。 　　四、工作流程
　　（一）制定计划与组织推动。在示范创建和示范推进阶段，国家每年为各省（区、市）分配示范性城乡老年友好型社区创建的数量指标。各地要制定创建工作计划，组织开展创建工作，分期分批推选符合条件的示范性社区。
　　（二）自愿申报与县级初核。符合条件的社区按照自愿的原则，填写全国示范性城乡老年友好型社区申请表（详见附件1）并在社区内公示，经县级初审通过后，报送省（区、市）卫生健康委（老龄办）复核。
　　（三）省级复核与推荐。各省（区、市）卫生健康委（老龄办）对参评社区提交的申请材料进行审核把关，提出审核意见，并向国家卫生健康委（全国老龄办）推荐本省（区、市）的参评社区。
　　（四）国家评审、公示、命名与授牌。国家卫生健康委（全国老龄办）通过组织专家审核、现场抽查等方式进行综合评审，对符合条件的示范性城乡老年友好型社区进行公示后予以命名并授牌。 　　五、工作要求
　　（一）加强组织领导。各地要充分认识全国示范性老年友好型社区创建工作的重要意义，把创建工作作为实施积极应对人口老龄化国家战略的一项具体举措，纳入本地经济社会发展规划及当地党委、政府的重点工作任务，健全工作机制，强化部门协同，加大投入保障。要研究制定具体实施方案，明确任务分工，实行目标管理，确保创建工作稳步、持续、深入开展。
　　（二）加强统筹协调。各级卫生健康委（老龄办）负责创建工作的具体组织和协调，要建立健全跨部门的协调机制，及时解决工作中遇到的困难和问题，研究制定相关配套政策措施，共同推进创建任务的全面落实。
　　（三）加强指导检查。各地要加强指导和检查，督促参评社区对标对表，认真对照全国示范性城乡老年友好型社区标准（详见附件2）开展创建工作，确保创建过程不走样，创建标准不打折扣。国家卫生健康委（全国老龄办）将不断完善示范性老年友好型社区建设的工作机制，建立健全科学规范、公正合理、与时俱进的考评指标体系，加大对已命名示范性城乡老年友好型社区的抽查和公开力度，建立动态调整机制，对于创建后工作质量下降、老年人满意度不高的社区将撤销命名。
　　（四）加强宣传推广。各地要充分利用电视、网络等宣传媒体，采取多种形式，认真做好示范性城乡老年友好型社区创建的宣传和推广工作，在全社会大力培育和践行社会主义核心价值观，倡导“积极老龄观、健康老龄化、幸福老年人”的理念，努力营造养老、孝老、敬老的社会环境，推动社会各界广泛参与示范性老年友好型社区创建工作，不断扩大创建工作的参与度和影响力。

　　一、城镇社区
　　（一）居住环境安全整洁
　　1.定期对独居、空巢、失能（含失智）、重残、计划生育特殊老年人家庭用水、用电和用气等设施进行安全检查或入户排查，对老化或损坏的及时改造维修，排除安全隐患。
　　2.建立社区防火和紧急救援网络，完善老年人住宅防火和紧急救援救助功能，鼓励为老年人家庭安装独立式感烟火灾探测报警器等设施设备。
　　3.定期开展老年人安全知识讲座，提高老年人安全知识水平，鼓励老年人参与社区安全教育和安全管理。
　　4.通过市场化运作、政府资助等方式对老年人家庭实施住房适老化改造，对空间布局、地面、扶手、厨房设备、如厕洗浴设备、紧急呼叫设备等进行适老化改造和维修，降低老年人生活风险。
　　5.加强社区生态环境建设，大力绿化和美化社区，营造卫生清洁、空气清新的社区环境。
　　6.做到社区内垃圾清运及时、无卫生死角、无暴露积存垃圾。帮助老年人学习垃圾分类知识，鼓励和协助老年人实施垃圾分类回收。
　　（二）出行设施完善便捷
　　7.加强老年人住宅公共设施无障碍改造，重点对坡道、楼梯、电梯、扶手等公共建筑节点进行改造，满足老年人基本安全通行要求。老年人口、残疾人口比例高的老旧小区增设电梯、坡道、休息座椅等无障碍设施设备。
　　8.普及社区公共基础设施无障碍建设，重点是社区道路设施、休憩设施、信息化设施、社区服务设施等与老年人日常生活密切相关的场所。
　　9.社区道路和公共设施建筑物内外设置清晰明确的标识系统，标识的安装安全牢固。
　　10.老年人集中活动的场所附近设置公共厕所，有条件的社区设置无障碍公共厕所，并配置紧急呼叫设备。
　　11.社区步行道路满足安全便利要求，保证步行道路平整安全，消除步行障碍物，严禁非法占用小区步行道。步行道路、台阶、活动场地等设施设置照明设施，保持安全通行的亮度。
　　12.社区道路系统设计人车分流，机动车道路采用低噪或降噪路面，并设置限速行驶标识和路面减速设施。
　　13.社区道路系统保证救护车辆能停靠在建筑的主要出入口处。
　　（三）社区服务便利可及
　　14.基层医疗卫生机构通过家庭医生签约服务，定期为老年人提供生活方式和健康状况评估、体格检查、辅助检查和健康指导等健康管理服务。
　　15.基层医疗卫生机构符合老年友善医疗机构相关要求。鼓励基层医疗卫生机构为高龄、失能、行动不便等居家老年人提供家庭病床、巡诊等上门医疗服务。
　　16.鼓励基层医疗卫生机构增加康复、护理床位，开设安宁疗护病区或床位。
　　17.支持发展社区嵌入式医养结合机构，为失能老年人提供长期照护服务。
　　18.采取健康宣传栏、健康讲座等多种形式，大力宣传老年健康核心信息、失能预防核心信息和阿尔茨海默病预防与干预核心信息等健康知识，普及健康老龄化理念和健康科学知识。
　　19.建立社区养老服务机构或设施，为老年人提供生活照料、助餐助行、紧急救援、精神慰藉等服务。
　　20.社区养老服务设施配备包括康复辅助器具在内的老年用品，并向有需要的老年人提供专业指导。
　　21.建立居家社区探访制度，定期探访独居、空巢、失能（含失智）、重残、计划生育特殊家庭等特殊困难老年人。
　　22.以多种形式为社区老年人提供助餐、助浴、助洁、代购、康复护理、紧急救援、康复辅具租赁等多样化服务。
　　23.按照社区老年人需求，持续开展心理疏导、情绪抚慰、关系调适、社会融入等专业社会工作服务。
　　24.开展老年人防诈骗知识与技巧宣传教育工作，提高老年人识别和防范非法集资、电信诈骗等非法侵害的能力。
　　25.社区设立公共法律服务室，为老年人提供法律援助等公共法律服务，帮助解决涉及老年人的纠纷及相关事务。
　　26.鼓励发展居家社区养老服务等志愿服务机制，鼓励和支持社区居民为有需求的老年人提供非专业性的养老服务。
　　（四）社会参与广泛充分
　　27.引导和组织老年人参与社区治理和服务，充分发挥老年人的积极作用。居民代表会议有老年人代表参加，社区开展与老年人相关的服务项目或活动时，充分听取老年人的意见和建议。
　　28.建立老年协会等基层老年社会组织，实行老年人自我管理、自我服务、自我教育、自我监督。
　　29.积极开展社区“银龄行动”，拓展老年人力资源开发，支持老年人广泛参与社区公益慈善、教科文卫等事业。
　　30.鼓励老年人自愿量力、依法依规参与经济社会发展，改善自身生活，实现自我价值。
　　31.成立社区老年文体团队，方便老年人就近参加各类文化体育活动，丰富精神文化生活。
　　32.为老年人和老年社会组织参与社区活动提供便利条件。依托社区综合服务设施因地制宜改造或修建综合性活动场所，满足老年人社会参与和文化生活需要。
　　33.定期了解老年人对社区参与的需求及意见，促进老年人广泛参与社区活动，融入社区。
　　34.在社区设立公益岗位，引入社会工作专业服务，引导和支持老年人广泛开展自助、互助和志愿活动。
　　35.鼓励社区自设老年教育学习点或与老年大学、教育机构和社会组织等合作在社区设立老年教育学习点，积极开展老年人思想道德、科学普及、休闲娱乐、健康知识、艺术审美、智能生活、法律法规、家庭理财、代际沟通、生命尊严等方面的教育。
　　36.丰富老年教育教学方式，充分利用社区内各种资源，因地制宜，方便老年人以各种形式经常性参与教育活动。支持建立不同类型的老年人学习团队，满足老年人自主学习的多样化需求。
　　（五）孝亲敬老氛围浓厚
　　37.对社区老年人开展积极老龄观教育，引导老年人树立终身发展理念，增强老年人的自尊、自强、自爱意识。
　　38.倡导全体社区居民树立积极老龄观，积极看待老龄社会，积极看待老年人和老年生活，积极做好全生命周期养老准备。
　　39.每年开展“活力老人”等践行积极老龄观先进典型人物事迹宣传活动。
　　40.组织多种形式的社区敬老爱老助老主题教育活动，加大对“敬老文明号”和“敬老爱老助老”模范人物的宣传报道。
　　41.评选宣传“最美家庭”、“五好家庭”等，强化子女的尊老敬老意识。对不履行赡养义务的子女，社区对其开展批评教育。
　　42.开展家庭养老照护培训及服务，提高失能老年人照护者的护理知识和技能，履行好家庭照料职责。鼓励各类社会力量为失能老年人家庭提供所需的支持性照护服务。
　　43.开展有利于促进代际互动、邻里互助的社区活动，增强不同代际间的文化融合和社会认同。
　　44.在社区开展人口老龄化国情教育和老年友好型社区理念宣传活动，形成人人关注、全民参与老年友好型社区建设的良好氛围。
　　45.开展《[中华人民共和国老年人权益保障法](javascript:SLC(328224))》及地方老年人权益保障法规普法宣传教育工作，增强老年人依法保护自身合法权益以及社区居民依法保护老年人合法权益的意识。
　　（六）科技助老智慧创新
　　46.提高社区为老服务信息化水平，利用智慧健康养老信息平台（社区综合服务平台），有效对接服务供给与需求信息，加强健康养老终端设备的适老化设计与开发，为老年人提供方便的智慧健康养老服务。
　　47.鼓励智能健康养老产品进社区进家庭，依托智慧养老平台和相关智能设备，为开展居家照护、医疗诊断、健康管理等提供远程服务及技术辅助服务。
　　48.通过社区老年教育学习点等平台，帮助老年人学习电脑、智能手机等智能产品和智能技术的使用，缩小老年人群与青年人群之间的“数字鸿沟”。
　　49.为使用智能技术困难的老年人在其高频活动场所保留必要的传统服务方式。
　　（七）管理保障到位有力
　　50.社区工作者中有专人负责老龄工作。每个社区至少配备一名以老年人服务为主的社会工作者。
　　51.逐步增加社区为老服务设施的财力投入，扶持社区各类为老服务设施的建设和正常运营。
　　52.建立老年友好型社区建设长效机制，统筹安排老年友好型社区建设工作。 　　二、农村社区
　　（一）居住环境安全整洁
　　1.保证老年人取水安全、便利，帮助老年人家庭完成自来水入户。
　　2.定期对独居、留守、失能（含失智）、重残、计划生育特殊老年人家庭用水、用电和用煤等设施进行安全检查或入户排查，对老化或损坏的及时改造维修，排除安全隐患。
　　3.基本完成农村户厕改造，无露天粪坑和简易茅厕。
　　4.生活垃圾及时清扫、收集，日产日清，村内无暴露和积存垃圾。河沟渠塘无积存垃圾、无白色污染、水面无明显漂浮物，村内无黑臭水体。帮助老年人养成文明如厕习惯，调动农村老年人家庭积极参与农村户厕改造。
　　5.结合农村危房改造工作，采取政府补贴等方式，对所有纳入特困供养、建档立卡范围的高龄、失能、残疾老年人家庭实施老年人住房适老化改造。有条件的地方可积极引导其他农村老年人家庭进行适老化改造。
　　（二）出行设施完善便捷
　　6. 社区步行道路满足安全便利要求，对社区主干道路进行硬化处理，修缮破损路，整治低洼路，保持路面平整安全。村内（小组）次干道进行适度硬化处理，实现“户户通”。
　　7.在社区主干道路和老年人活动场所安装路灯，保持安全通行的亮度。
　　8.社区内电力、通信、有线电视线路架设安全规范，无违章交越和搭挂。
　　9.设置休息空间和座椅，老年人主要活动场所的临空侧设置栏杆和扶手等安全阻挡设施。
　　10.老年人集中活动的场所附近设置卫生公厕，鼓励有条件的村对公厕进行无障碍改造。
　　（三）社区服务便利可及
　　11.积极推进乡镇卫生院和村卫生室一体化管理，为老年人提供便利的基本医疗卫生服务。实现基本医疗保险联网直接结算。
　　12.通过家庭医生签约服务，定期为老年人提供生活方式和健康状况评估、体格检查、辅助检查和健康指导等健康管理服务。
　　13.加强村卫生室服务能力建设，鼓励村医参与健康养老服务，为老年人提供医、养、康、护结合的医养结合服务。
　　14.依托村卫生室，通过健康宣传栏、健康讲座多种形式，推进老年人健康促进和健康教育活动，宣传失能预防核心信息和阿尔茨海默病预防与干预核心信息等健康知识，普及健康老龄化理念和健康科普知识。
　　15.加强农村社区综合服务能力建设，为老年人提供养老、公共文化、医疗卫生、全民健身等综合性服务。
　　16.建立农村独居、留守、失能（含失智）、重残、计划生育特殊家庭老年人定期探访制度，做好老年人基本信息摸查，以电话问候、上门访问等方式，定期探访老年人，及时了解老年人生活情况，将存在安全风险和生活困难的老年人作为重点帮扶对象，及时通知其子女或其他法定赡养人。
　　17.建立农村互助幸福院，由党员干部、乡贤人士、热心村民及社会爱心人士组成志愿服务队伍，对留守、失能（含失智）、计划生育特殊家庭等特殊困难老年人实施结对帮扶。
　　18.鼓励村民依托自家居住地提供家庭式养老服务。
　　19.开展老年人防诈骗知识与技巧宣传教育工作，提高老年人识别和防范非法集资、电信诈骗等非法侵害的能力。
　　20.为老年人提供法律援助等公共法律服务，帮助解决涉及老年人的纠纷及相关事务。
　　21.探索农村养老服务志愿服务机制，鼓励村民和老年人参与各种公益性活动和志愿服务。支持家族成员和亲友对留守老年人给予生活照料和精神关爱，鼓励邻里乡亲为留守老年人提供关爱服务。
　　（四）社会参与广泛充分
　　22.帮助老年人拓展农副产品销售渠道，优先帮助经济困难的老年人申请社区公益性岗位或联系用工机会，促进农村老年人致富增收。
　　23.引导和组织老年人参与社区治理和服务，充分发挥老年人的积极作用。村代表会议有老年人代表参加，村里开展与老年人相关的服务项目或活动时，充分听取老年人的意见和建议。
　　24.建立老年协会等基层老年社会组织，实行老年人自我管理、自我服务、自我教育、自我监督。
　　25.成立老年文体团队，方便老年人就近参加各类活动，丰富精神文化生活。
　　26.为老年人和老年社会组织参与社区活动提供便利条件。
　　27.有效整合乡村教育文化资源，发展农村社区老年教育。鼓励自设老年教育学习点或与老年大学、教育机构和社会组织等合作在农村社区设立老年教育学习点，以村民喜爱的形式开展适应农村老年人需求的教育活动。
　　（五）孝亲敬老氛围浓厚
　　28.强化家庭在农村老年人赡养与关爱服务中的主体责任，增强村规民约对家庭赡养义务人的道德约束，在尊重老年人意愿的前提下，赡养义务人可与亲属或其他人签订委托照顾协议，并向村民委员会报备。对赡养人、扶养人不履行赡养、扶养义务的，由村民委员会及老年人组织监督其履行。
　　29.组织多种形式的敬老爱老助老主题教育活动，加大对“敬老文明号”和“敬老爱老助老模范人物”的宣传报道，发挥孝亲敬老典型的示范引导作用。
　　30.开展《[中华人民共和国老年人权益保障法](javascript:SLC(328224))》及地方老年人权益保障法规普法宣传教育工作，增强老年人依法保护自身合法权益以及村民依法保护老年人合法权益的意识。
　　31.对老年人进行积极老龄观教育，引导老年人树立终身发展理念，增强老年人的自尊、自强、自爱意识。
　　（六）科技助老智慧创新
　　32.加快互联网信息建设，促进宽带网络进入老年人家庭，支持设置公共电脑室，方便留守老年人与子女视频联络。
　　33.通过农村老年教育学习点等平台，帮助老年人学习电脑、智能手机等智能产品和智能技术使用，缩小老年人群与青年人群之间的“数字鸿沟”。
　　34.鼓励农村“互联网＋养老”发展，提升农村养老服务信息化水平，促进养老服务资源供需对接。
　　35.加快农村智能广播网（“村村响”广播）建设，安装规范，分布合理，每天固定时间安排播出，能实现应急插播。
　　（七）管理保障到位有力
　　36.社区工作者中有专人负责老龄工作。
　　37.逐步增加农村社区为老服务设施的财力投入，扶持农村社区各类为老服务设施的建设和正常运营。
　　38.建立农村老年友好型社区建设长效机制，统筹安排农村老年友好型社区建设工作。

**住房城乡建设部、发展改革委、民政部、卫生健康委、医保局、**

**全国老龄办关于推动物业服务企业发展居家社区养老服务的意见**

　　为贯彻落实党中央、国务院关于加快发展养老服务业的一系列决策部署，充分发挥物业服务企业常驻社区、贴近居民、响应快速等优势，推动和支持物业服务企业积极探索“物业服务＋养老服务”模式，切实增加居家社区养老服务有效供给，更好满足广大老年人日益多样化多层次的养老服务需求，着力破解高龄、空巢、独居、失能老年人生活照料和长期照护难题，促进家庭幸福、邻里和睦、社区和谐，现就推动物业服务企业发展居家社区养老服务提出以下意见。
　　一、补齐居家社区养老服务设施短板
　　（一）盘活小区既有公共房屋和设施。清理整合居住小区内各类闲置和低效使用的公共房屋和设施，经业主共同决策同意，可交由物业服务企业统一改造用于居家社区养老服务；政府所有的闲置房屋和设施，由房屋管理部门按规定履行程序后，可交由物业服务企业用于居家社区养老服务。鼓励物业服务企业与房地产开发企业协商，将开发企业自持的房屋改造为养老服务用房，允许按照适老化设计要求优化户均面积、小区车位配比等指标，相关建设工程应符合国家工程建设消防技术标准和消防安全管理要求。
　　（二）保障新建居住小区养老服务设施达标。新建居住小区应落实居家社区养老服务设施规划建设要求，按照相关政策和标准配套建设居家社区养老服务设施，并与住宅同步规划、同步建设、同步验收、同步交付使用。对缓建、缩建、停建、不建养老服务设施的项目，在整改到位之前不得组织竣工验收。支持利用集体建设用地发展养老服务设施。加强居家社区养老服务设施设计、施工、验收、备案等环节的监督管理，保障设施建设达标。
　　（三）加强居家社区养老服务设施布点和综合利用。按照集中和分散兼顾、独立和混合使用并重的原则，完善居家社区养老服务设施布点。在老年人较多的若干相邻小区，集中建设老年服务中心，可交由物业服务企业为老年人提供全托、日托、上门、餐饮、文体、健身等方面的服务，提高养老设施使用效率。因地制宜多点布局小型养老服务点，作为居家社区养老服务中心的有效补充，方便小区老年人就地就近接受服务。
　　（四）推进居家社区适老化改造。支持物业服务企业根据老年人日常生活和社会交往需要，进行增设无障碍通道、加装电梯等设施适老化改造，以及提供地面防滑、加装扶手、消除地面高差等居家社区适老化改造。 　　二、推行“物业服务＋养老服务”居家社区养老模式
　　（五）养老服务营收实行单独核算。物业服务企业开展居家社区养老服务，应当内设居家社区养老服务部门，专门提供助餐、助浴、助洁、助急、助行、助医、照料看护等定制养老服务，并按国家有关规定，建立健全财务会计制度，对社区养老服务的营业收支实行单独核算。
　　（六）支持养老服务品牌化连锁化经营。支持物业服务企业根据自身条件，结合养老需求，成立独立的居家社区养老服务机构，实现居家社区养老服务规模化、品牌化、连锁化经营。物业服务企业已经取得居家社区养老服务机构营业执照的，允许其跨区域经营居家社区养老服务。
　　（七）组建专业化养老服务队伍。鼓励开展居家社区养老服务的物业服务企业建立养老服务专业人员队伍，加强岗前培训及定期培训。协调职业院校、培训机构为物业服务企业培养养老护理、康复、社会工作、心理咨询等专业人员提供人力资源支持。按规定落实养老服务从业人员培训费补贴、职业技能鉴定补贴等政策。将符合条件的养老服务从业人员纳入公租房或政策性租赁住房保障范围。 　　三、丰富居家社区养老服务内容
　　（八）支持参与提供医养结合服务。鼓励物业服务企业开办社区医务室、护理站等医疗机构，招聘和培训专业人员，为老年人提供基本医护服务，支持将符合条件的医疗机构纳入医保支付范围。支持社区医务室、护理站与大型医疗机构建立长期合作关系和就医双向转介绿色通道。鼓励医护人员到社区医务室、护理站执业，并在职称评定等方面享有同等待遇。探索开展居家老年人上门医疗卫生服务。
　　（九）支持开展老年人营养服务和健康促进。鼓励物业服务企业因地制宜地开办小区老年餐桌，提供送餐上门服务。鼓励开办社区课堂，开展老年人思想道德、科学文化、养生保健、心理健康、法律法规、家庭理财、闲暇生活、代际沟通、生命尊严等方面教育，加强对老年人及其家属的营养和照护知识培训，指导老年人开展科学的体育健身活动，搭建老年文化活动平台。支持设置老年人康复辅助器具配置、租赁站点，满足老年人相应康复需求。
　　（十）发展社区助老志愿服务。鼓励物业服务企业加强与社区居民委员会、业主委员会的沟通合作，共同健全社区动员和参与机制，开展社区居民结对帮扶老年人志愿服务活动，以及敬老助老孝老主题教育和代际沟通活动，加强对老年人的精神关爱服务，为老年人参与社区生活搭建平台。
　　（十一）促进养老产业联动发展。支持物业服务企业在提供居家社区养老服务中，加强与专业养老机构信息和业务联通，开展技术交流、人员培训、资源共享和客户转介等方面的合作，推进居家、社区、机构养老融合发展，积极构建全方位、多层次、立体化的养老服务体系。 　　四、积极推进智慧居家社区养老服务
　　（十二）建设智慧养老信息平台。鼓励物业服务企业对接智慧城市和智慧社区数据系统，建设智慧养老信息平台，将社区老年人生活情况、健康状态、养老需求、就医诊疗等数据信息纳入统一的数据平台管理；开辟家政预约、购物购药、健康管理、就医挂号、绿色转诊等多项网上服务功能，提升居家社区养老服务智能化水平。
　　（十三）配置智慧养老服务设施。鼓励物业服务企业对居家社区养老服务设施进行智能化升级改造，配置健康管理、人身安全监护、家用电器监控、楼寓对讲和应急响应等智能设施。大力推广物联网和远程智能安防监控技术，实现24小时安全自动值守，提高突发事件应对能力，降低老年人意外风险。
　　（十四）丰富智慧养老服务形式。鼓励物业服务企业参与开发居家社区养老服务智能终端、应用程序等，拓展远程提醒和控制、自动报警和处置、动态监测和记录等功能。以失能、独居、空巢老年人为重点，建立呼叫服务系统和应急救援服务机制。支持打造“互联网＋养老”模式，整合线上线下资源，精准对接助餐、助浴、助洁、助行、助医等需求与供给，为老年人提供“点菜式”便捷养老服务。
　　（十五）创新智慧养老产品供给。鼓励物业服务企业参与研发推广智能可穿戴设备、便携式健康监测设备、智能养老监护设备、家庭服务机器人等智能养老服务产品，推进人工智能、虚拟现实、5G等新兴技术在居家社区养老智能产品中的应用。支持物业服务企业发展以老年产品为特色的电商服务平台，为老年人提供多元、个性、精准的智能产品。 　　五、完善监督管理和激励扶持措施
　　（十六）加强养老服务监管。物业服务企业开展居家社区养老服务和有关设施应符合消防、环保、卫生、应急管理等相关标准及行业管理要求。物业服务企业为老年人提供居家社区养老服务，应严格保障老年人的信息安全，避免个人信息泄露。
　　（十七）规范养老服务收费行为。居家社区养老服务收费应当遵循诚实信用、公平合理、费用与服务水平相适应的原则。居家社区养老服务收费必须明码标价，在服务区域内的显著位置公示企业名称、服务内容、收费标准、投诉方式等事项，确保老年人的知情权、参与权、选择权和监督权。
　　（十八）拓宽养老服务融资渠道。鼓励商业银行向提供居家社区养老服务的物业服务企业发放资产（设施）抵押贷款和应收账款质押贷款，并参照贷款基准利率，结合风险分担情况，合理确定贷款利率水平。支持物业服务企业以企业未来收益权、土地使用权为担保发行债券。鼓励商业保险、基金、信托、社保基金等资金投资居家社区养老服务项目，降低物业服务企业负债率。
　　（十九）建立协同推进机制。加强组织领导，强化部门协同，将发展居家社区养老服务工作纳入养老服务部际联席会议制度，统筹推进。住房和城乡建设部负责物业服务企业开展居家社区养老服务组织协调工作，民政部负责养老服务工作的业务指导、监督管理，国家卫生健康委负责指导社区医务室、护理站设立和运营工作，国家医保局负责指导将符合条件的医疗机构纳入医保协议管理范围，全国老龄办负责老年人照顾服务指导工作。地方省级政府有关部门按照职责分工负责本行政区内推动物业服务企业发展居家社区养老服务相关工作。市县政府有关部门负责落实落细各项政策措施，积极探索实践，及时研究解决问题，为物业服务企业开展居家社区养老服务创造条件。

**全国老龄办关于开展“智慧助老”行动的通知**

各省、自治区、直辖市及新疆生产建设兵团卫生健康委（老龄办），各涉老社会组织：
　　为深入贯彻党中央、国务院关于老龄工作的决策部署，落实《国务院办公厅关于切实解决老年人运用智能技术困难的实施方案》（国办发﹝2020﹞45号）要求，切实维护老年人在信息时代下的合法权益，帮助老年人跨越“数字鸿沟”，全国老龄办决定开展“智慧助老”行动，利用3年的时间，动员社会各方力量共同努力，推动老龄社会信息无障碍建设，促进全社会推进适老化的改造和升级，提升老年人运用智能技术方面的获得感、幸福感、安全感。现将有关事项通知如下：
　　一、行动目标
　　到2022年底，通过建立常态化工作机制、开展志愿服务、强化技能培训、加大宣传力度等措施，使用智能技术帮助老年人更好地适应信息社会的发展，让更多的老年人用得上、愿意用、用得好智能技术，充分保障在全社会智能化飞速发展过程中老年人的合法权益，推动实现老龄社会治理体系和治理能力现代化。 　　二、行动内容
　　（一）建立健全“智慧助老”的常态化工作机制。将开展“智慧助老”行动、切实解决老年人运用智能技术困难，列为各级老龄委年度工作要点，加强督促落实。协调推动各级各部门以老年人面临的实际困难为导向，采取具体措施予以解决。将帮助老年人运用智能技术相关内容纳入每年“敬老月”的主题活动，倡导全社会推动、关注、参与“智慧助老”行动。将在“智慧助老”行动中事迹突出的机构、企业和个人纳入全国“敬老文明号”和全国“敬老爱老助老模范人物”评选表彰。
　　（二）广泛动员各方力量为老年人提供志愿培训服务。培育壮大老年志愿服务队伍，鼓励在医疗卫生、金融服务、政务便民、交通出行、文化旅游等重点行业服务场所增设志愿服务岗。组织动员社区服务人员等以“一对一”或“一对多”的形式开展面向老年人的智能技术培训。组织涉老相关社会团体、志愿服务组织等，面向老年人开展以智能技术运用培训为主题的志愿服务活动。通过组织兴趣示范小组、智能技术运用比赛等形式，动员运用智能技术能力较强的老年人带动、帮助、培训其他老年人，提高有意愿的老年人运用智能技术的能力。
　　（三）充分发挥老年大学在智能技术培训中的重要作用。将智能技术运用相关内容纳入老年大学、社区老年学习点等的学习课程，并通过给予适当激励、授予证书等方式调动老年人学习运用智能技术的积极性。将认识风险、预防受骗等知识列为老年大学的必修课程，帮助老年人掌握防骗的知识和技能，提升运用智能技术的信心。
　　（四）引导老年人正确认识网络信息和智能技术。通过科普讲座、大众传媒等老年人喜闻乐见的形式，引导老年人正确认识网络信息和智能技术，将其作为丰富晚年生活、提升生活质量的重要工具，消除对网络信息和智能技术的恐惧和排斥心理。鼓励老年人主动学习网络防骗的有关政策、法规和知识，切实增强风险意识。
　　（五）加强智能技术运用和防骗知识的科普宣传。编制老年人智能技术运用指南，开发简明易懂的培训教材，利用广播、电视网络等媒介进行宣传推广，引导全社会增强帮助老年人运用智能技术的责任意识。鼓励各地依托全国智慧健康养老示范基地开展老年群体数字化生活场所体验，设立老年人智能产品用品体验区，并为老年人提供技术支持和科普服务。编制老年人防网络诈骗指导手册，以视频、一图读懂等通俗易懂的形式在大众媒体和老年人经常活动的场所进行广泛宣传，提高防骗知识技能的知晓度。以拍摄主题宣传片、公益广告等方式，加大对网络诈骗、电子通讯诈骗案件的曝光和宣传力度，发挥典型案件的警示作用。
　　（六）提倡家庭成员帮助老年人运用智能技术。探索将帮助老年人运用智能技术纳入其家庭成员照护培训内容，引导家庭成员帮助老年人提升智能技术运用能力。积极宣传引导，鼓励家庭成员加强对老年人运用智能技术的辅导，帮助老年人甄别各类不实信息。尊重老年人保持原有的生活方式，在银行、医院、购物、出行等必须运用智能技术的场所和生活场景，动员家庭成员为老年人代办相关业务。
　　（七）大力开展智能产品社会募捐活动。积极搭建慈善募捐平台，广泛动员爱心企业、社会人士以及通讯公司等开展慈善公益活动，为城乡贫困老年人捐赠智能产品，减免通讯服务资费，帮助贫困老年人解决无智能产品可用和服务费用昂贵的问题。 　　三、行动安排
　　（一）行动启动阶段（2020年11月至2020年12月）。印发通知，在全国启动“智慧助老”行动。建立行动组织协调办公室，组建行动咨询专家库。组织召开工作座谈会，开展专项调研。
　　（二）深入推进阶段（2021年1月至2022年6月）。各地围绕行动目标，完善相关制度和政策，制订年度工作计划，深入开展教育培训、志愿服务等主题活动。全国老龄办组织开展针对行动的调研评估，积极宣传各地的好经验、好做法，在全社会营造良好的舆论氛围。
　　（三）总结提升阶段（2022年7月至2022年12月）。对“智慧助老”行动进行总结评估，将各地在行动过程中形成的经验和做法在全国推广，并适时上升为国家政策。 　　四、保障措施
　　（一）加强组织领导。各地要高度重视，将开展“智慧助老”行动纳入重要议事日程，制定工作方案，明确任务分工，抓好组织实施。充分发挥老龄委的议事协调作用，协调动员各相关部门认真履行职责，确保行动的各项任务措施落到实处。
　　（二）加强评估指导。各地要注重对“智慧助老”专项行动的评估，建立健全评价机制，对于组织活动踊跃、活动效果显著的地方、部门和单位予以通报表扬，对于敷衍塞责、搞形式主义的予以通报批评。全国老龄办将结合舆情监测情况，对严重侵害老年人合法权益的事件进行通报和督促整改。各地要结合实际，针对重点部门、重点内容开展实地调研，加强协调配合和督促指导，切实保障“智慧助老”行动的实际效果。
　　（三）加强宣传倡导。各地要充分利用广播、电视、报纸、网络及新媒体平台，通过线上线下相结合的方式，大力宣传“智慧助老”的具体措施和典型事迹，在全社会营造全民行动、“智慧助老”的浓厚氛围。

**国务院办公厅关于促进养老托育服务健康发展的意见**

促进养老托育服务健康发展，有利于改善民生福祉，有利于促进家庭和谐，有利于培育经济发展新动能。为贯彻落实党中央、国务院决策部署，更好发挥各级政府作用，更充分激发社会力量活力，更好实现社会效益和经济效益相统一，持续提高人民群众的获得感、幸福感、安全感，经国务院同意，现提出以下意见。
　　一、健全老有所养、幼有所育的政策体系
　　（一）分层次加强科学规划布局。根据“一老一小”人口分布和结构变化，科学谋划“十四五”养老托育服务体系，促进服务能力提质扩容和区域均衡布局。省级人民政府要将养老托育纳入国民经济和社会发展规划统筹推进，并制定“十四五”养老托育专项规划或实施方案。建立常态化督查机制，督促专项规划或实施方案的编制和实施，确保新建住宅小区与配套养老托育服务设施同步规划、同步建设、同步验收、同步交付。
　　（二）统筹推进城乡养老托育发展。强化政府保基本兜底线职能，健全基本养老服务体系。优化乡村养老设施布局，整合区域内服务资源，开展社会化管理运营，不断拓展乡镇敬老院服务能力和辐射范围。完善老年人助餐服务体系，加强农村老年餐桌建设。探索在脱贫地区和城镇流动人口集聚区设置活动培训场所，依托基层力量提供集中托育、育儿指导、养护培训等服务，加强婴幼儿身心健康、社会交往、认知水平等方面早期发展干预。
　　（三）积极支持普惠性服务发展。大力发展成本可负担、方便可及的普惠性养老托育服务。引导各类主体提供普惠性服务，支持非营利性机构发展，综合运用规划、土地、住房、财政、投资、融资、人才等支持政策，扩大服务供给，提高服务质量，提升可持续发展能力。优化养老托育营商环境，推进要素市场制度建设，实现要素价格市场决定、流动自主有序、配置高效公平，促进公平竞争。
　　（四）强化用地保障和存量资源利用。在年度建设用地供应计划中保障养老托育用地需求，并结合实际安排在合理区位。调整优化并适当放宽土地和规划要求，支持各类主体利用存量低效用地和商业服务用地等开展养老托育服务。在不违反国家强制性标准和规定前提下，各地可结合实际制定存量房屋和设施改造为养老托育场所设施的建设标准、指南和实施办法。建立健全“一事一议”机制，定期集中处置存量房屋和设施改造手续办理、邻避民扰等问题。在城市居住社区建设补短板和城镇老旧小区改造中统筹推进养老托育服务设施建设，鼓励地方探索将老旧小区中的国企房屋和设施以适当方式转交政府集中改造利用。支持在社区综合服务设施开辟空间用于“一老一小”服务，探索允许空置公租房免费提供给社会力量供其在社区为老年人开展助餐助行、日间照料、康复护理、老年教育等服务。支持将各类房屋和设施用于发展养老托育，鼓励适当放宽最长租赁期限。非独立场所按照相关安全标准改造建设托育点并通过验收的，不需变更土地和房屋性质。
　　（五）推动财税支持政策落地。各地要建立工作协同机制，加强部门信息互通共享，确保税费优惠政策全面、及时惠及市场主体。同步考虑公建服务设施建设与后期运营保障，加强项目支出规划管理。完善运营补贴激励机制，引导养老服务机构优先接收经济困难的失能失智、高龄、计划生育特殊家庭老年人。对吸纳符合条件劳动者的养老托育机构按规定给予社保补贴。
　　（六）提高人才要素供给能力。加强老年医学、老年护理、社会工作、婴幼儿发展与健康管理、婴幼儿保育等学科专业建设，结合行业发展动态优化专业设置，完善教学标准，加大培养力度。按照国家职业技能标准和行业企业评价规范，加强养老托育从业人员岗前培训、岗位技能提升培训、转岗转业培训和创业培训。加大脱贫地区相关技能培训力度，推动大城市养老托育服务需求与脱贫地区劳动力供给有效对接。深化校企合作，培育产教融合型企业，支持实训基地建设，推行养老托育“职业培训包”和“工学一体化”培训模式。 　　二、扩大多方参与、多种方式的服务供给
　　（七）增强家庭照护能力。支持优质机构、行业协会开发公益课程，利用互联网平台等免费开放，依托居委会、村委会等基层力量提供养老育幼家庭指导服务，帮助家庭成员提高照护能力。建立常态化指导监督机制，加强政策宣传引导，强化家庭赡养老年人和监护婴幼儿的主体责任，落实监护人对孤寡老人、遗弃儿童的监护责任。
　　（八）优化居家社区服务。发展集中管理运营的社区养老和托育服务网络，支持具备综合功能的社区服务设施建设，引导专业化机构进社区、进家庭。建立家庭托育点登记备案制度，研究出台家庭托育点管理办法，明确登记管理、人员资质、服务规模、监督管理等制度规范，鼓励开展互助式服务。
　　（九）提升公办机构服务水平。加强公办和公建民营养老机构建设，坚持公益属性，切实满足特困人员集中供养需求。建立入住综合评估制度，结合服务能力适当拓展服务对象，重点为经济困难的失能失智、高龄、计划生育特殊家庭老年人提供托养服务。完善公建民营机制，打破以价格为主的筛选标准，综合从业信誉、服务水平、可持续性等质量指标，引进养老托育运营机构早期介入、全程参与项目工程建设，探索开展连锁化运营。
　　（十）推动培训疗养资源转型发展养老服务。按照“应改尽改、能转则转”的原则，将转型发展养老服务作为党政机关和国有企事业单位所属培训疗养机构改革的主要方向。各地要加大政策支持和协调推进力度，集中解决资产划转、改变土地用途、房屋报建、规划衔接等困难，确保转养老服务项目2022年底前基本投入运营。鼓励培训疗养资源丰富、养老需求较大的中东部地区先行突破，重点推进。
　　（十一）拓宽普惠性服务供给渠道。实施普惠养老托育专项行动，发挥中央预算内投资引领作用，以投资换机制，引导地方政府制定支持性“政策包”，带动企业提供普惠性“服务包”，建设一批普惠性养老服务机构和托育服务机构。推动有条件的用人单位以单独或联合相关单位共同举办的方式，在工作场所为职工提供托育服务。支持大型园区建设服务区内员工的托育设施。
　　（十二）引导金融机构提升服务质效。鼓励政府出资产业投资基金及市场化的创业投资基金、私募股权基金等按照市场化、法治化原则，加大对养老托育领域的投资力度。创新信贷支持方式，在依法合规、风险可控、商业可持续前提下，推进应收账款质押贷款，探索收费权质押贷款，落实好信贷人员尽职免责政策。鼓励金融机构合理确定贷款期限，灵活提供循环贷款、年审制贷款、分期还本付息等多种贷款产品和服务。扩大实施养老产业专项企业债券和养老项目收益债券，支持合理灵活设置债券期限、选择权及还本付息方式，鼓励发行可续期债券。引导保险等金融机构探索开发有针对性的金融产品，向养老托育行业提供增信支持。支持保险机构开发相关责任险及养老托育机构运营相关保险。

三、打造创新融合、包容开放的发展环境
　　（十三）促进康养融合发展。支持面向老年人的健康管理、预防干预、养生保健、健身休闲、文化娱乐、旅居养老等业态深度融合。发挥中医药独特优势，促进中医药资源广泛服务老年人群体。支持各类机构举办老年大学、参与老年教育，推动举办“老年开放大学”、“网上老年大学”，搭建全国老年教育资源共享和公共服务平台。
　　（十四）深化医养有机结合。发展养老服务联合体，支持根据老年人健康状况在居家、社区、机构间接续养老。为居家老年人提供上门医疗卫生服务，构建失能老年人长期照护服务体系。有效利用社区卫生服务机构、乡镇卫生院等基层医疗资源，开展社区医养结合能力提升行动。针对公共卫生突发事件，提升养老机构应急保障能力，增设隔离功能并配备必要的防控物资和设备，加强工作人员应急知识培训。
　　（十五）强化产品研发和创新设计。健全以企业为主体的创新体系，鼓励采用新技术、新工艺、新材料、新装备，增强以质量和信誉为核心的品牌意识，建立健全企业知识产权管理体系，推进高价值专利培育和商标品牌建设，培育养老托育服务、乳粉奶业、动画设计与制作等行业民族品牌。促进“一老一小”用品制造业设计能力提升，完善创新设计生态系统。
　　（十六）促进用品制造提质升级。逐步完善养老托育服务和相关用品标准体系，加强标准制修订，强化标准实施推广，探索建立老年用品认证制度。推进互联网、大数据、人工智能、5G等信息技术和智能硬件的深度应用，促进养老托育用品制造向智能制造、柔性生产等数字化方式转型。推进智能服务机器人后发赶超，启动康复辅助器具应用推广工程，实施智慧老龄化技术推广应用工程，构建安全便捷的智能化养老基础设施体系。鼓励国内外多方共建养老托育产业合作园区，加强市场、规则、标准方面的软联通，打造制造业创新示范高地。
　　（十七）培育智慧养老托育新业态。创新发展健康咨询、紧急救护、慢性病管理、生活照护、物品代购等智慧健康养老服务。发展“互联网＋养老服务”，充分考虑老年群体使用感受，研究开发适老化智能产品，简化应用程序使用步骤及操作界面，引导帮助老年人融入信息化社会，创新“子女网上下单、老人体验服务”等消费模式，鼓励大型互联网企业全面对接养老服务需求，支持优质养老机构平台化发展，培育区域性、行业性综合信息平台。发展互联网直播互动式家庭育儿服务，鼓励开发婴幼儿养育课程、父母课堂等。
　　（十八）加强宜居环境建设。普及公共基础设施无障碍建设，鼓励有条件的地区结合城镇老旧小区改造加装电梯。加强母婴设施配套，在具备条件的公共场所普遍设置专席及绿色通道。引导房地产项目开发充分考虑养老育幼需求。指导各地加快推进老年人居家适老化改造。以满足老年人生活需求和营造婴幼儿成长环境为导向，推动形成一批具有示范意义的活力发展城市和社区。 　　四、完善依法从严、便利高效的监管服务
　　（十九）完善养老托育服务综合监管体系。以养老托育机构质量安全、从业人员、运营秩序等方面为重点加强监管。落实政府在制度建设、行业规划、行政执法等方面的监管责任，实行监管清单式管理，明确监管事项、监管依据、监管措施、监管流程，监管结果及时向社会公布。养老托育机构对依法登记、备案承诺、履约服务、质量安全、应急管理、消防安全等承担主体责任。健全行业自律规约，加强正面宣传引导和社会舆论监督，加快构建以信用为基础的新型监管机制。
　　（二十）切实防范各类风险。加强突发事件应对，建立完善养老托育机构突发事件预防与应急准备、监测与预警、应急处置与救援、事后恢复与重建等工作机制。将养老托育纳入公共安全重点保障范围，支持服务机构安全平稳运转。完善退出机制，建立机构关停等特殊情况应急处置机制。严防“一老一小”领域以虚假投资、欺诈销售、高额返利等方式进行的非法集资，保护消费者合法权益。
　　（二十一）优化政务服务环境。完善机构设立办事指南，优化办事流程，实施并联服务，明确办理时限，推进“马上办、网上办、就近办”。制定养老托育政务服务事项清单，推进同一事项无差别受理、同标准办理，力争实现“最多跑一次”。推进养老托育政务服务的“好差评”工作，完善评价规则，加强评价结果运用，改进提升政务服务质量。
　　（二十二）积极发挥多方合力。支持公益慈善类社会组织参与，鼓励机构开发志愿服务项目，建立健全“一老一小”志愿服务项目库。引导互联网平台等社会力量建立养老托育机构用户评价体系。以普惠为导向建立多元主体参与的养老和托育产业合作平台，在要素配置、行业自律、质量安全、国际合作等方面积极作为。发挥行业协会商会等社会组织积极性，开展机构服务能力综合评价，引领行业规范发展，更好弘扬尊老爱幼社会风尚。
　　（二十三）强化数据资源支撑。依据[养老产业统计分类](javascript:SLC(340011))，开展养老产业认定方法研究，推进重要指标年度统计。探索构建托育服务统计指标体系。利用智库和第三方力量加强研究，开展人口趋势预测和养老托育产业前景展望，通过发布年度报告、白皮书等形式，服务产业发展，引导社会预期。
　　坚持党委领导、政府主导，地方各级政府要建立健全“一老一小”工作推进机制，结合实际落实本意见要求，以健全政策体系、扩大服务供给、打造发展环境、完善监管服务为着力点，促进养老托育健康发展，定期向同级人民代表大会常务委员会报告服务能力提升成效。国务院各部门要根据职责分工，制定具体落实举措，推动各项任务落地。国家发展改革委要建立“一老一小”服务能力评价机制，加强对本意见落实工作的跟踪督促，及时向国务院报告。

**中国银保监会办公厅关于银行保险机构切实解决老年人运用智能技术困难的通知**

　　一、保留和改进传统金融服务方式
　　（一）优化网点布局。各银行保险机构要积极融入老年友好型社会建设，发展服务老年人的特色网点。要加强金融服务下沉，进一步完善基础金融服务，根据老年客户群体数量和金融服务需求，合理科学进行网点布局。

（二）保留和改进人工服务。各银行保险机构要合理配置服务人员，为老年人办理业务提供引导。有条件的营业网点要开设绿色通道或专属服务窗口。完善自助设备和客服热线，精准识别客户年龄和需求，通过设置老年专属客服坐席，提供一键转接、呼叫人工或老年人专属菜单等方式，及时为遇到困难的老年人提供帮助。

　（三）完善柜面服务。各银行保险机构要进一步丰富服务项目，优化业务流程，简化办事手续，切实提高无障碍服务水平。根据实际情况，在营业网点内适当增加爱心座椅、老花镜等设施配置，方便老年人办理业务。有条件的银行保险机构可根据老年人数量和业务需要，在城乡结合部或偏远农村地区，部署可移动智能柜台、设置流动服务点或派出服务流动车等方式为老年人办理日常业务。 　　（四）尊重老年人使用习惯。商业银行要保留仍在使用中的纸质存折、存单等老年人熟悉的服务方式，不得强迫老年人使用银行卡，不得强制老年人通过自助式智能设备办理业务，不得违规代替老年人操作，不得对老年人使用柜面人工服务设置分流率等考核指标。

（五）完善应急保障措施。针对社保卡激活、社保资金发放、养老金领取等老年人阶段性集中办理业务，各银行保险机构要采取有效措施，灵活安排人力，减少等待时间，方便老年人办理。
　　二、提升网络消费便利化水平

　（六）优化使用流程。各银行保险机构要根据老年人的使用习惯，在用户注册、银行卡绑定和支付流程等环节，在保证严格认证身份并明确老年人办理意愿的基础上改进验证方式，提升老年人使用体验。

　（七）打造适老手机银行APP。各商业银行要开发手机银行APP的大字、语音、民族语言等服务，突出查询、转账及缴费等老年人常用功能，实现关键信息易读、主要功能易找、操作步骤易懂。探索开发“一键求助”功能，对老年人在操作过程中遇到问题的，快速介入提供引导帮助，及时解决问题。

（八）丰富适老产品和服务。各商业银行要进一步创新网络消费相关金融产品和服务方式，研发符合老年人需求和风险承受能力的网络消费金融产品及服务。
　　三、推进互联网应用适老化改造

（九）实施金融互联网网站、移动互联网应用适老化改造。各银行保险机构要完善互联网网站、移动互联网应用，进一步优化界面交互、内容朗读、操作提示、语音辅助等功能，便于老年人获取信息和服务。可根据老年人使用习惯，开发应用“关怀模式”“长辈模式”，页面内容要清晰、简洁，重点突出。

　（十）推广使用便携式智能服务终端。各银行保险机构可采用便携式智能服务终端设备，针对老年人实际需求，进一步扩展上门服务项目，促进传统上门服务和智能技术融合，为老年人提供贴身金融服务。
　　四、加强教育宣传和培训

（十一）提升教育精度。各银行保险机构要聚焦老年人日常生活涉及的高频服务事项，制作简易使用手册和视频教程，在老年人办理服务事项时，及时为老年人提供指引，帮助老年人提高运用智能技术的能力和水平。

　（十二）丰富教育形式。各银行保险机构可积极与老年大学（学校）、养老服务机构、社区教育机构合作开发培训课程，通过进社区、进农村、进家庭、进养老机构开展体验学习、尝试应用、经验交流、互助帮扶等，帮助老年人了解新事物、体验新科技，融入智慧社会。

　（十三）加大宣传力度。各银行保险机构要积极参与“3·15”国际消费者权益保护日、“7·8”全国保险公众宣传日和金融知识进万家等宣传活动，大力普及银行保险智能技术应用知识，推动防范非法集资、电信网络诈骗等宣传普及，增强老年人对智能技术的了解和信任。

　（十四）加强从业人员培训。各银行保险机构要倡导尊老爱老敬老理念，关心老年人需求，改进服务态度。要完善从业人员培训内容，加强解决老年人运用智能技术常见困难、预防提示电信网络诈骗等内容培训，提高窗口服务水平和应急处理能力。
　　五、保障信息安全

　（十五）网络安全政策。各银行保险机构应落实主体责任，建立完善网络安全相关政策和保障机制。规范智能化产品和服务中的个人信息收集、使用等政策，综合运用多种安全防护手段和风险控制措施，切实增强老年人使用智能技术的安全保障。

　（十六）网络安全治理。各银行保险机构应通过安全测试、现场检查、漏洞通报和问题处置，持续加强技术监测和监督检查，切实保障老年人信息安全。
　　各银行保险机构要高度重视解决老年人运用智能技术困难工作。一是迅速排查实际情况，明确涉及老年人运用智能技术的高频服务场景和具体困难。二是抓紧制定解决措施，明确目标成效、时间进度和责任分工，力求措施精准、可操作，行之有效、可评估。三是精心组织实施，密切跟踪分析有关措施进展及成效，加强指导督促，确保做实做细。四是加强宣传交流，营造安全放心的智能技术应用场景，让老年人能用、会用、敢用、想用。银保监会直接监管的银行保险机构要定期总结相关工作开展情况、工作成效亮点、存在的困难和问题等情况，于2021年、2022年每半年结束后15日内将有关情况报送银保监会消保局。

**国务院关于印发“十四五”国家老龄事业发展和养老服务体系规划的通知**

一、规划背景
　　党和国家高度重视老龄事业和养老服务体系发展。“十三五”时期，在党和国家重大规划和政策意见引领下，我国老龄事业发展和养老服务体系建设取得一系列新成就。一是老龄政策法规体系不断完备。涉老相关法律法规、规章制度和政策措施不断完善，老年人权益保障机制、优待政策等不断细化，养老服务体系建设、运营、发展的标准和监管制度更加健全。二是多元社会保障不断加强。基本社会保险进一步扩大覆盖范围，企业退休人员养老保险待遇和城乡居民基础养老金水平得到提升。稳步推进长期护理保险试点工作，明确了两批共49个试点城市，在制度框架、政策标准、运行机制、管理办法等方面作出探索。商业养老保险、商业健康保险快速发展。三是养老服务体系不断完善。“十三五”期间，全国各类养老服务机构（包括养老机构、社区养老服务机构，下同）和设施从11.6万个增加到32.9万个，床位数从672.7万张增加到821万张。各级政府持续推进公办养老机构建设，加强特困人员养老保障，对经济困难的高龄、失能（含失智，下同）老年人给予补贴，初步建立农村留守老年人关爱服务体系。居家社区养老服务发展迅速，机构养老服务稳步推进，普惠养老专项行动顺利实施。四是健康支撑体系不断健全。老年人健康水平持续提升，2020年人均预期寿命提高至77.9岁，65岁及以上老年人在基层医疗卫生机构免费获得健康管理服务。医养结合服务有序发展，照护服务能力明显提高，2020年全国两证齐全（具备医疗卫生机构资质，并进行养老机构备案）的医养结合机构5857家，床位数达到158万张。五是老龄事业和产业加快发展。老年教育机构持续增加，老年人精神文化生活不断丰富，更多老年人积极参与社区治理、文教卫生等活动。老年宜居环境建设积极推进，老年人权益保障持续加强。老年用品制造业和服务业加快转型升级，科技化水平显著提升，教育培训、文化娱乐、健康养生、旅居养老等融合发展的新业态不断涌现。
　　“十四五”时期，我国开启全面建设社会主义现代化国家新征程。党中央把积极应对人口老龄化上升为国家战略，在《[中华人民共和国国民经济和社会发展第十四个五年规划和2035年远景目标纲要](javascript:SLC(353607))》中作了专门部署。人口老龄化是人类社会发展的客观趋势，我国具备坚实的物质基础、充足的人力资本、历史悠久的孝道文化，完全有条件、有能力、有信心解决好这一重大课题。同时也要看到，我国老年人口规模大，老龄化速度快，老年人需求结构正在从生存型向发展型转变，老龄事业和养老服务还存在发展不平衡不充分等问题，主要体现在农村养老服务水平不高、居家社区养老和优质普惠服务供给不足、专业人才特别是护理人员短缺、科技创新和产品支撑有待加强、事业产业协同发展尚需提升等方面，建设与人口老龄化进程相适应的老龄事业和养老服务体系的重要性和紧迫性日益凸显，任务更加艰巨繁重。

　二、总体要求
　　（一）指导思想。
　　以习近平新时代中国特色社会主义思想为指导，全面贯彻党的十九大和十九届历次全会精神，统筹推进“五位一体”总体布局，协调推进“四个全面”战略布局，坚持稳中求进工作总基调，立足新发展阶段，完整、准确、全面贯彻新发展理念，构建新发展格局，坚持党委领导、政府主导、社会参与、全民行动，实施积极应对人口老龄化国家战略，以加快完善社会保障、养老服务、健康支撑体系为重点，把积极老龄观、健康老龄化理念融入经济社会发展全过程，尽力而为、量力而行，深化改革、综合施策，加大制度创新、政策供给、财政投入力度，推动老龄事业和产业协同发展，在老有所养、老有所医、老有所为、老有所学、老有所乐上不断取得新进展，让老年人共享改革发展成果、安享幸福晚年。
　　（二）基本原则。
　　--系统谋划，整体推进。坚持应对人口老龄化和促进经济社会发展相结合，坚持满足老年人需求和解决人口老龄化问题相结合，统筹把握老年群体与全体社会成员、老年期与全生命周期、老龄政策与公共政策的关系，系统整体推进老龄事业发展。
　　--以人为本，顺应趋势。贯彻以人民为中心的发展思想，聚焦老年人在社会保障、养老、医疗等民生问题上的“急难愁盼”，加快建设符合中国国情、顺应人口老龄化趋势的保障和服务体系，优化服务供给，提升发展质量，确保始终与经济社会发展相适应。
　　--兜好底线，广泛普惠。推进养老服务体系建设，强化政府保基本兜底线职能，促进资源均衡配置，确保基本养老服务保障到位。大力发展普惠型养老服务，充分调动社会力量积极性，为人民群众提供方便可及、价格可负担、质量有保障的养老服务。
　　--改革创新，扩大供给。深化放管服改革，优化营商环境，培育新产业、新业态、新模式，推动服务业多业态深度融合发展，打造制造业创新示范高地。大力发展银发经济，推动老龄事业与产业、基本公共服务与多样化服务协调发展，努力满足老年人多层次多样化需求。
　　--多方参与，共建共享。坚持政府、社会、家庭、个人共同参与、各尽其责，弘扬中华民族孝亲敬老传统美德，巩固家庭养老的基础地位，打造老年友好型社会。引导老年人树立主动健康和终身发展理念，鼓励老年人积极面对老年生活，在经济社会发展中充分发挥作用。
　　（三）发展目标。
　　“十四五”时期，积极应对人口老龄化国家战略的制度框架基本建立，老龄事业和产业有效协同、高质量发展，居家社区机构相协调、医养康养相结合的养老服务体系和健康支撑体系加快健全，全社会积极应对人口老龄化格局初步形成，老年人获得感、幸福感、安全感显著提升。
　　养老服务供给不断扩大。覆盖城乡、惠及全民、均衡合理、优质高效的养老服务供给进一步扩大，家庭养老照护能力有效增强，兜底养老服务更加健全，普惠养老服务资源持续扩大，多层次多样化养老服务优质规范发展。
　　老年健康支撑体系更加健全。老年健康服务资源供给不断增加，配置更加合理，人才队伍不断扩大。家庭病床、上门巡诊等居家医疗服务积极开展。老年人健康水平不断提升，健康需求得到更好满足。
　　为老服务多业态创新融合发展。老年人教育培训、文化旅游、健身休闲、金融支持等服务不断丰富，围绕老年人衣食住行、康复护理的老年用品产业不断壮大，科技创新能力明显增强，智能化产品和服务惠及更多老年人。
　　要素保障能力持续增强。行业营商环境持续优化，规划、土地、住房、财政、投资、融资、人才等支持政策更加有力，从业人员规模和能力不断提升，养老服务综合监管、长期护理保险等制度更加健全。
　　社会环境更加适老宜居。全国示范性老年友好型社区建设全面推进，敬老爱老助老的社会氛围日益浓厚，老年人社会参与程度不断提高。老年人在运用智能技术方面遇到的困难得到有效解决，广大老年人更好地适应并融入智慧社会。

| 专栏1　“十四五”国家老龄事业发展和养老服务体系主要指标 | |
| --- | --- |
| 指 标 | 2025年目标值 |
| 1．养老服务床位总量 | 达到900万张以上 |
| 2．特殊困难老年人月探访率 | 达到100% |
| 3．新建城区、新建居住区配套建设养老服务设施达标率 | 达到100% |
| 4．养老机构护理型床位占比 | 达到55% |
| 5．设立老年医学科的二级及以上综合性医院占比 | 达到60%以上 |
| 6．本科高校、职业院校养老服务相关专业招生规模 | 明显增长 |
| 7．每千名老年人配备社会工作者人数 | 保持1人以上 |
| 8．老年大学覆盖面 | 每个县（市、区、旗）至少1所 |
| 9．“敬老月”活动覆盖面 | 每个县（市、区、旗）每年开展1次 |

　　三、织牢社会保障和兜底性养老服务网
　　（四）进一步健全社会保障制度。
　　完善基本养老保险和基本医疗保险体系。不断扩大基本养老保险覆盖面。尽快实现企业职工基本养老保险全国统筹。实施渐进式延迟法定退休年龄。落实基本养老金合理调整机制，适时适度调整城乡居民基础养老金标准。大力发展企业年金、职业年金，提高企业年金覆盖率，促进和规范发展第三支柱养老保险，推动个人养老金发展。完善基本医保政策，逐步实现门诊费用跨省直接结算，扩大老年人慢性病用药报销范围，将更多慢性病用药纳入集中带量采购，降低老年人用药负担。
　　稳步建立长期护理保险制度。适应我国经济社会发展水平和老龄化发展趋势，构建长期护理保险制度政策框架，协同促进长期照护服务体系建设。从职工基本医疗保险参保人群起步，重点解决重度失能人员基本护理保障需求。探索建立互助共济、责任共担的多渠道筹资机制，参加长期护理保险的职工筹资以单位和个人缴费为主，形成与经济社会发展和保障水平相适应的筹资动态调整机制。建立公平适度的待遇保障机制，合理确定待遇保障范围和基金支付水平。制定全国统一的长期护理保险失能等级评估标准，建立并完善长期护理保险需求认定、等级评定等标准体系和管理办法，明确长期护理保险基本保障项目。做好与经济困难的高龄、失能老年人补贴以及重度残疾人护理补贴等政策的衔接。健全长期护理保险经办服务体系。
　　完善社会救助和社会福利制度。健全分层分类的社会救助体系，将符合条件的老年人纳入相应社会救助范围，予以救助。为经济困难的老年人提供养老服务补贴，为经济困难的失能老年人提供护理补贴，并建立补贴标准动态调整机制。推动地方探索通过政府购买服务等方式为经济困难的失能老年人等提供必要的访视、照料服务。
　　（五）建立基本养老服务清单制度。
　　建立老年人能力综合评估制度。统筹现有的老年人能力、健康、残疾、照护等相关评估制度，通过政府购买服务等方式，统一开展老年人能力综合评估，推动评估结果全国范围内互认、各部门按需使用，作为接受养老服务等的依据。研究制定可满足老年人能力综合评估需要的国家标准，提供统一、规范和可操作的评估工具。推动培育一批综合评估机构，加强能力建设和规范管理。
　　针对不同老年人群体分类提供服务。各地要根据财政承受能力，出台基本养老服务清单，对健康、失能、经济困难等不同老年人群体，分类提供养老保障、生活照料、康复照护、社会救助等适宜服务。清单要明确服务对象、服务内容、服务标准和支出责任，并根据经济社会发展和科技进步进行动态调整。
　　（六）强化公办养老机构兜底保障作用。
　　坚持公办养老机构公益属性。各地要根据特困老年人规模确定公办养老机构床位总量下限，做好规划建设和保运转等工作。在满足有意愿的特困老年人集中供养需求的前提下，公办养老机构重点为经济困难的空巢、留守、失能、残疾、高龄老年人以及计划生育特殊家庭老年人等（以下统称特殊困难老年人）提供服务。建立公办养老机构入住评估管理制度，明确老年人入住条件和排序原则。引导公建民营、民办公助等养老机构优先接收特殊困难老年人、作出特殊贡献的老年人。鼓励地方探索解决无监护人老年人入住养老机构难的问题。
　　提升公办养老机构服务水平。加大现有公办养老机构改造力度，提升失能老年人照护能力，增设失智老年人照护专区，在满足政策保障对象入住需求的基础上优先安排失能老年人入住。支持1000个左右公办养老机构增加护理型床位。针对公共卫生、自然灾害等突发事件，增设隔离功能，改造消防设施，配备必要的物资和设备，加强人员应急知识培训，提升公办养老机构应急保障能力。发挥公办养老机构作用，辐射带动周边各类养老机构完善突发事件预防与应急准备、监测与预警、应急处置与救援等机制。

| 专栏2　公办养老机构提升行动 |
| --- |
| 提升覆盖能力达标率。新建和升级改造设区的市级公办养老机构。县级、乡镇级重点支持特困人员供养服务设施（敬老院）建设，改造升级护理型床位，开辟失能老年人照护单元，到2025年，县级特困人员供养服务设施（敬老院）建有率达到100%。 　　提升服务质量安全达标率。加强公办养老机构规范化建设，使其符合养老机构服务安全基本规范等标准。依据养老机构等级划分与评定等标准，评定为一级至二级服务等级的乡镇级公办养老机构、评定为二级至三级服务等级的县级公办养老机构建有率均达到80%以上。 　　提升入住率。改善公办养老机构服务，优化供给结构，公办养老机构入住率明显提升，用好用足现有资源。 |

　　（七）加快补齐农村养老服务短板。
　　通过支持县级养老服务机构建设改造、将具备条件的乡镇级特困人员供养服务设施（敬老院）改扩建为区域养老服务中心、综合利用残疾人托养服务设施等方式，因地制宜实现农村有意愿的特困老年人集中供养。以村级邻里互助点、农村幸福院等为依托，构建农村互助式养老服务网络。支持乡镇级特困人员供养服务设施（敬老院）增加养老服务指导功能，将专业养老服务延伸至村级邻里互助点、农村幸福院和居家老年人。对于特困人员供养服务设施（敬老院）原地改造升级项目，不需要调整规划用途，不额外占用建设指标。加强农村养老服务和管理人才队伍建设，提高职业化、专业化水平。以行政村为单位，依托村民自治组织和邻里互助力量，建立特殊困难老年人定期巡访制度，督促家庭成员履行赡养扶养义务，提供必要的援助服务，帮助解决基本生活安全问题。 　　四、扩大普惠型养老服务覆盖面
　　（八）建设普惠养老服务网络。
　　发展社区养老服务机构。深化“十三五”时期居家和社区养老服务试点改革成果，培育一批以照护为主业、辐射社区周边、兼顾上门服务的社区养老服务机构，推动集中管理运营和标准化、品牌化发展。支持社区养老服务机构建设和运营家庭养老床位，将服务延伸至家庭。支持物业企业发挥贴近住户的优势，与社区养老服务机构合作提供居家养老服务。在乡镇（街道）层面，建设具备全日托养、日间照料、上门服务、供需对接、资源统筹等功能的区域养老服务中心。到2025年，乡镇（街道）层面区域养老服务中心建有率达到60%，与社区养老服务机构功能互补，共同构建“一刻钟”居家养老服务圈。
　　支持建设专业化养老机构。支持社会力量建设专业化、规模化、医养结合能力突出的养老机构，推动其在长期照护服务标准规范完善、专业人才培养储备、信息化智能化管理服务、康复辅助器具推广应用等方面发挥示范引领作用。支持养老机构针对失智老年人的特殊需求，提供专业照护服务。引导养老机构立足自身定位，合理延伸服务范围，依法依规开展医疗卫生服务，为老年人提供一体化的健康和养老服务。中央预算内投资重点支持新建护理型养老服务设施和照护服务能力改造提升项目。引导地方对普通型床位和护理型床位实行差异化补助，到2025年，全国养老机构护理型床位占比提高到55%。完善对护理型床位的认定办法，尽快建立长期照护服务的项目、标准、质量评价等规范。
　　积极推进公办养老机构改革。完善公办养老机构委托经营机制，改革以价格为主的筛选标准，综合考虑从业信誉、服务水平、可持续性等质量指标。引进养老服务领域专业能力较强的运营机构早期介入、全程参与委托经营的养老机构项目工程建设，支持规模化、连锁化运营。探索将具备条件的公办养老机构改制为国有养老服务企业或拓展为连锁服务机构。探索建立城市养老服务联合体，“以上带下”提升基层服务能力。
　　（九）支持普惠养老服务发展。
　　完善社区养老服务设施配套。各地要严格按照人均用地不少于0.1平方米的标准分区分级规划设置社区养老服务设施，老龄化程度较高的地区可结合实际适当上调标准。加强常态化督查，确保新建居住区与配套养老服务设施同步规划、同步建设、同步验收、同步交付。开展城镇配套养老服务设施专项治理，全面清查2014年以来新建城区、新建居住区配套情况，定期进行全国通报，2025年前完成整改。在城镇老旧小区改造中，统筹推进配套养老服务设施建设，通过补建、购置、置换、租赁、改造等方式，因地制宜补齐社区养老服务设施短板。支持在社区综合服务设施开辟空间用于养老服务。支持养老机构利用配套设施提供社区养老服务，具备条件的可重点开展失能老年人全日托养服务，无偿或低偿使用配套设施的，应当以普惠为导向确定服务价格。鼓励地方探索对相邻居住区的配套养老服务设施进行资源整合、统筹利用，统一管理运营。定期组织开展社区养老服务设施使用状况检查，对于未按养老服务用途使用的配套设施产权方，支持地方探索依法实施合理的经济处罚方式。
　　充分调动社会力量参与积极性。综合运用规划、土地、住房、财政、投资、融资、人才等支持政策，引导各类主体提供普惠养老服务，扩大供给，提高质量，提升可持续发展能力。进一步完善市场原则下的普惠价格形成机制，“十四五”期间，各地要结合实际，综合考虑企业建设运营成本、政策支持情况、消费者承受能力等因素，推动普惠养老服务价格在合理区间运行，价格水平显著低于当地同等服务水平的市场化养老服务机构。实施普惠养老专项行动，发挥中央预算内投资引导和撬动作用，引导地方政府制定支持性“政策包”，带动企业提供普惠型“服务包”，推动建设一批方便可及、价格可接受、质量有保障的养老服务机构。
　　加大国有经济对普惠养老的支持。建立国有经济对养老服务供给的补短板机制，强化中央国有经济在养老服务领域有效供给，加强地方国有经济在养老基础设施领域布局。引导地方国有资本积极培育发展以普惠养老服务为主责主业的国有企业。对主要承担养老服务功能的国有企业，重点考核服务质量、成本控制、运营效率等情况。 　　五、强化居家社区养老服务能力
　　（十）构建城乡老年助餐服务体系。
　　建立老年人助餐服务网络。综合利用社区养老服务设施和闲置房屋等资源，打造一批食材可溯、安全卫生、价格公道的标准化社区老年食堂（助餐服务点）。重点补齐农村、远郊等助餐服务短板，支持当地养老服务机构、餐饮场所等增加助餐功能，推广邻里互助的助餐模式。丰富和创新助餐服务提供机制，因地制宜采取中央厨房、社区食堂、流动餐车等形式，降低运营成本，便利老年人就餐。
　　支持高质量多元化供餐。围绕更好满足老年人多层次多样化就餐需求，鼓励助餐机构开发餐饮产品、丰富菜色品种、合理营养膳食。建立助餐服务合理回报机制，由经营者根据实际服务成本和适度利润水平确定收费标准，引导更多市场主体参与助餐服务。引导外卖平台等市场主体参与助餐配送。推动助餐机构投保食品安全责任保险。
　　（十一）开展助浴助洁和巡访关爱服务。
　　发展老年人助浴服务。支持社区助浴点、流动助浴车、入户助浴等多种业态发展，培育一批专业化、连锁化助浴机构。研究制定老年人助浴服务相关标准规范，加强养老护理员助浴技能培训。支持助浴服务相关产品研发，推广应用经济实用型产品。鼓励助浴机构投保相关保险，提高风险保障程度。
　　引导助洁服务覆盖更多老年人。支持家政企业开发被褥清洗、收纳整理、消毒除尘等适合老年人需求的保洁服务产品。引导物业企业将保洁服务范围由公共区域向老年人家庭延伸。支持有条件的地方通过政府购买服务、组织开展志愿服务等方式，为特殊困难老年人提供助洁服务。
　　加强居家老年人巡访关爱。建立居家养老巡访关爱服务制度，实行普遍巡访和重点巡访相结合，采取电话问候、上门探访等多种形式，运用互联网、物联网等技术手段，为老年人提供紧急救援服务。通过“社工＋邻里＋志愿者＋医生”相结合的方式，为特殊困难老年人提供身心关爱服务。
　　（十二）加快发展生活性为老服务业。
　　提高老年人生活服务可及性。依托社区养老服务设施，引导社区综合服务平台广泛对接老年人需求，提供就近就便消费服务。组织和引导物业企业、零售服务商、社会工作服务机构等拓展为老服务功能，提供生活用品代购、餐饮外卖、家政预约、代收代缴、挂号取药、精神慰藉等服务。
　　培育老年人生活服务新业态。推动“互联网＋养老服务”发展，推动互联网平台企业精准对接为老服务需求，支持社区养老服务机构平台化展示，提供“菜单式”就近便捷为老服务，鼓励“子女网上下单、老人体验服务”。培育城市级综合信息平台和行业垂直信息平台。引导有条件的养老服务机构线上线下融合发展，利用互联网、大数据、人工智能等技术创新服务模式。鼓励互联网企业开发面向老年人各种活动场景的监测提醒功能，利用大数据方便老年人的居家出行、健康管理和应急处置。 　　六、完善老年健康支撑体系
　　（十三）加强老年健康教育和预防保健。
　　完善健康教育和健康管理。开发老年健康教育科普教材，通过老年健康宣传周等多种活动，利用多种传播媒介普及健康知识和健康生活方式，提高老年人健康素养。落实基本公共卫生服务老年人健康管理项目，做实老年人家庭医生签约服务。加强老年人群重大传染病的早期筛查、干预，鼓励有条件的地方开展阿尔茨海默病、帕金森病等神经退行性疾病的早期筛查和健康指导。
　　实施老年健康促进工程。加强老年人群重点慢性病的早期筛查、干预及分类指导，开展老年口腔健康、老年营养改善、老年痴呆防治和心理关爱行动。推动老年健康领域科研成果转化，遴选推广一批老年健康适宜技术，提高基层的老年健康服务能力。发挥中医药在老年病、慢性病防治等方面的优势和作用。
　　（十四）发展老年医疗、康复护理和安宁疗护服务。
　　增强医疗卫生机构为老服务能力。加强国家老年医学中心建设，布局若干区域老年医疗中心。加强综合性医院老年医学科建设。支持医疗资源丰富的地区将部分公立医疗机构转型为护理院、康复医院。推动医疗卫生机构开展老年综合征管理，促进老年医疗服务从单病种模式向多病共治模式转变。加快建设老年友善医疗机构，方便老年人看病就医。
　　推动医疗服务向居家社区延伸。支持有条件的医疗卫生机构为失能、慢性病、高龄、残疾等行动不便或确有困难的老年人提供家庭病床、上门巡诊等居家医疗服务。公立医疗机构为老年人提供上门医疗服务，采取“医疗服务价格＋上门服务费”方式收费。提供的医疗服务、药品和医用耗材适用本医疗机构执行的医药价格政策，上门服务费可由公立医疗机构自主确定。鼓励社会力量开办社区护理站。积极开展社区和居家中医药健康服务。
　　开展安宁疗护服务。推动医疗卫生机构按照“充分知情、自愿选择”的原则开展安宁疗护服务。稳步扩大安宁疗护试点，推动安宁疗护机构标准化、规范化建设。支持社区和居家安宁疗护服务发展，建立机构、社区和居家相衔接的安宁疗护服务机制。加强对社会公众的生命教育。

| 专栏3　老年健康服务体系建设行动 |
| --- |
| 老年健康促进工程。监测老年人健康素养状况，开展有针对性的健康教育活动。将老年心理关爱行动覆盖至所有县（市、区、旗）。在先行试点的基础上，实施老年口腔健康行动和老年营养改善行动。实施老年痴呆防治行动，提升老年痴呆防治水平。 　　老年健康服务体系建设工程。构建综合连续、覆盖城乡的老年健康服务体系。加强综合性医院老年医学科以及老年医院、康复医院、护理院（中心、站）、安宁疗护机构建设。鼓励社会力量开办护理院（中心、站）。在国家安宁疗护试点市（区），每个县（市、区、旗）至少设立1个安宁疗护病区，有条件的社区卫生服务中心和乡镇卫生院设立安宁疗护病床。 |

　　（十五）深入推进医养结合。
　　丰富医养结合服务模式。鼓励大型或主要接收失能老年人的养老机构内部设置医疗卫生机构，将养老机构内设医疗卫生机构纳入医联体管理，根据服务老年人的特点，合理核定养老机构举办的医疗机构医保限额。推动养老机构与周边医疗卫生机构开展签约合作，做实合作机制和内容。到2025年，养老机构普遍具备医养结合能力（能够提供医疗卫生服务或与医疗卫生机构开展签约合作）。
　　增加医养结合服务供给。实施社区医养结合能力提升行动。积极开展基本公共卫生服务老年健康与医养结合服务项目。支持优抚医院、光荣院转型，开展医养结合服务。推动社区卫生服务中心与社区养老服务机构、乡镇卫生院与特困人员供养服务设施（敬老院）、村卫生室与农村幸福院毗邻建设，采取多种有效方式实现资源整合、服务衔接。
　　提升医养结合服务质量。健全医养结合标准规范体系。推动医疗卫生、养老服务数据共享，完善医养结合信息管理系统。推进“互联网＋医疗健康”、“互联网＋护理服务”、“互联网＋康复服务”，发展面向居家、社区和机构的智慧医养结合服务。

| 专栏4　医养结合能力提升专项行动 |
| --- |
| 社区医养结合能力提升行动。依托社区卫生服务中心、乡镇卫生院或养老服务机构、特困人员供养服务设施（敬老院），利用现有资源改建一批社区（乡镇）医养结合服务设施，重点为失能、慢性病、高龄、残疾等老年人提供健康教育、预防保健、疾病诊治、康复护理、安宁疗护为主，兼顾日常生活照料的医养结合服务。 　　医养结合示范行动。利用中央预算内投资支持建设专业化、规模化、医养结合能力突出的养老服务机构。组织开展医养结合人才能力提升培训。组织开展全国医养结合示范省（自治区、直辖市）、示范县（市、区、旗）和示范机构创建活动。 |

　　（十六）强化老年人疫情防控。
　　制定老年人突发公共卫生事件应急处置预案和指南，分类完善居家、社区和入住养老机构的老年人疫情防控措施。在疫情应急处置中，充分发挥基层党组织和基层自治组织的作用，做好特殊困难老年人的就医帮助、生活照顾、心理慰藉等服务。加强养老机构疫情防控制度和能力建设。 　　七、大力发展银发经济
　　（十七）发展壮大老年用品产业。
　　加强老年用品研发制造。大力开发满足老年人衣、食、住、行等需求的老年生活用品。针对不同生活场景，重点开发适老化家电、家具、洗浴装置、坐便器、厨房用品等日用产品以及智能轮椅、生物力学拐杖等辅助产品，推广易于抓握的扶手等支撑装置以及地面防滑产品、无障碍产品，发展老年益智类玩具、乐器等休闲陪护产品。针对机构养老、日间托养、上门护理等需求，重点开发清洁卫生、饮食起居、生活护理等方面产品，提升成人尿裤、护理垫、溃疡康复用品等产品的适老性能，发展辅助搬运、翻身、巡检等机器人。发展老年人监护、防走失定位等产品。
　　促进优质产品应用推广。制修订一批关键急需的老年用品和服务技术标准，促进质量提升，规范市场秩序，引导消费者正确选择和使用。建立老年用品产品目录，适时进行评估并动态调整。对自主研发、技术领先、市场认可的产品，优先纳入升级和创新消费品指南。在有条件的街道、社区，发展嵌入式康复辅助器具销售和租赁网点，提供用品展示、预约使用、指导教学、售后维修、回收利用等服务。
　　鼓励发展产业集群。鼓励国内外多方共建特色养老产业合作园区，加强市场、规则、标准方面的软联通，打造制造业创新示范高地。优先培育一批带动力强、辐射面广的龙头企业，打造一批产业链长、覆盖领域广、经济社会效益显著的产业集群，形成一批具有国际竞争力的知名品牌，推动我国相关产业迈向全球价值链中高端。

| 专栏5　规划布局一批银发经济重点发展区域 |
| --- |
| 在京津冀、长三角、粤港澳大湾区、成渝等区域，规划布局10个左右高水平的银发经济产业园区。支持北京、天津、上海、海南、重庆在开展服务业扩大开放综合试点中推进国际性、跨区域合作。结合积极应对人口老龄化重点联系城市评选，在全国打造一批银发经济标杆城市，推进在服务业融合发展、制造业转型升级、新技术新业态培育方面的探索创新。建立区域老年用品市场交易平台，支持有条件的地区举办老年用品博览会、展销会。 |

　　（十八）促进老年用品科技化、智能化升级。
　　强化老年用品的科技支撑。加快推进互联网、大数据、人工智能、第五代移动通信（5G）等信息技术和智能硬件在老年用品领域的深度应用。支持智能交互、智能操作、多机协作等关键技术研发，提升康复辅助器具、健康监测产品、养老监护装置、家庭服务机器人、日用辅助用品等适老产品的智能水平、实用性和安全性，开展家庭、社区、机构等多场景的试点试用。
　　加强老年科技的成果转化。利用现有资金渠道，支持老年用品关键技术和产品研发、成果转化、服务创新及应用推广，促进产业创新。支持在老年用品领域培育国家技术创新示范企业、“专精特新”企业、制造业单项冠军企业等，加强产学研用协同创新和关键共性技术产业化。加强老年用品领域知识产权保护，依法保护相关专利、商标和商誉等合法权益。
　　发展健康促进类康复辅助器具。加快人工智能、脑科学、虚拟现实、可穿戴等新技术在健康促进类康复辅助器具中的集成应用。发展外骨骼康复训练、认知障碍评估和训练、沟通训练、失禁康复训练、运动肌力和平衡训练、老年能力评估和日常活动训练等康复辅助器具。发展用药和护理提醒、呼吸辅助器具、睡眠障碍干预以及其他健康监测检测设备。
　　推广智慧健康养老产品应用。针对老年人康复训练、行为辅助、健康理疗和安全监护等需求，加大智能假肢、机器人等产品应用力度。开展智慧健康养老应用试点示范建设，建设众创、众包、众扶、众筹等创业支撑平台，建立一批智慧健康养老产业生态孵化器、加速器。编制智慧健康养老产品及服务推广目录，完善服务流程规范和评价指标体系，推动智慧健康养老规范化、标准化发展。

| 专栏6　老年用品研发制造应用重大科技攻关 |
| --- |
| 结合“十四五”国家重点研发计划相关专项的实施，加强对高龄老年人机能增强和照护、失能老年人用品等的研发。围绕神经系统损伤、损伤后脑认知功能障碍、瘫痪助行等康复治疗需求，突破脑机交互等技术，开发用于不同损伤康复的辅助机器人系列产品，实施智能服务机器人发展行动计划。研发穿戴式动态心电监测设备和其他生理参数检测设备，发展便携式健康监测设备、自助式健康检测设备等健康监测产品，开发新型信号采集芯片和智能数字医疗终端。 |

　　（十九）有序发展老年人普惠金融服务。
　　促进和规范发展第三支柱养老保险。支持商业保险机构开发商业养老保险和适合老年人的健康保险，引导全社会树立全生命周期的保险理念。引导商业保险机构加快研究开发适合居家护理、社区护理、机构护理等多样化护理需求的产品。研究建立寿险赔付责任与护理支付责任转换机制，支持被保险人在失能时提前获得保险金给付，用于护理费用支出。支持老年人住房反向抵押养老保险业务发展。积极推进老年人意外伤害保险。鼓励金融机构开发符合老年人特点的支付、储蓄、理财、信托、保险、公募基金等养老金融产品，研究完善金融等配套政策支持。加强涉老金融市场的风险管理，严禁金融机构误导老年人开展风险投资。 　　八、践行积极老龄观
　　（二十）创新发展老年教育。
　　加快发展城乡社区老年教育，支持各类有条件的学校举办老年大学（学校）、参与老年教育。鼓励养教结合创新实践，支持社区养老服务机构建设学习点。发挥社区教育办学网络的作用，办好家门口的老年教育。依托国家开放大学筹建国家老年大学，搭建全国老年教育资源共享和公共服务平台。推动各地开放大学举办“老年开放大学”，鼓励老年教育机构开展在线老年教育。创新机制，推动部门、行业企业、高校举办的老年大学面向社会开放办学。
　　（二十一）鼓励老年人继续发挥作用。
　　加强老年人就业服务。鼓励各地建立老年人才信息库，为有劳动意愿的老年人提供职业介绍、职业技能培训和创新创业指导服务。健全相关法律法规和政策，保障老年人劳动就业权益和创业权益。支持老年人依法依规从事经营和生产活动，兴办社会公益事业。按照单位按需聘请、个人自愿劳动原则，鼓励专业技术人才合理延长工作年限。
　　促进老年人社会参与。在全社会倡导积极老龄观，引导老年人根据自身情况，积极参与家庭、社区和社会发展。积极开展“银龄行动”，支持老年人参与文明实践、公益慈善、志愿服务、科教文卫等事业。建设高层次老年人才智库，在调查研究、咨询建言等方面发挥作用。鼓励和引导老年人在城乡社区建立基层老年协会等基层老年社会组织，搭建自我服务、自我管理、自我教育平台。指导和促进基层老年社会组织规范化建设。

| 专栏7　基层老年协会规范化建设行动 |
| --- |
| 发挥基层党组织作用，加强基层老年协会党建工作，改善基层老年协会活动设施和条件，加强骨干培训和活动指导。通过政府购买服务等方式，引入专业社会工作者、社会组织等对基层老年协会进行培育孵化，打造一批规范化、专业化基层老年协会。做好基层老年协会的登记（备案）工作，推动各地制定切实可行的具体监管措施，加强规范管理。 |

　　（二十二）丰富老年人文体休闲生活。
　　扩大老年文化服务供给。改扩建或新建一批老年公共文体活动场所，支持通过公建民营、委托经营、购买服务等方式提高运营效率。鼓励编辑出版适合老年人的大字本图书，加强弘扬孝亲敬老美德的艺术作品创作，在广播电视和互联网播放平台增加播出，推出养老相关公益广告。搭建老年文化活动交流展示平台，支持老年文化团体和演出队伍登上乡村、社区舞台。鼓励和支持电影院、剧场等经营性文化娱乐场所增加面向老年人的优惠时段。
　　支持老年人参与体育健身。在体育公园、全民健身中心等公共体育设施布局中充分考虑老年人健身需求，加强配套运动场所和设施的规划建设。鼓励开发适合老年人的体育健身项目，搭建平台组织相关赛事和锻炼展示活动。发布老年人科学健身活动指南，根据差异化的身体素质推荐适合的运动项目和锻炼强度，推广中国传统保健体育运动。鼓励建立老年人全民健身志愿服务队伍，指导和帮助老年人科学开展各类体育健身项目。营造良性的体育健身消费环境，鼓励推出适合老年人的体育服装、锻炼器材等产品以及健身指导、竞赛参与等服务。
　　促进养老和旅游融合发展。引导各类旅游景区、度假区加强适老化建设和改造，建设康养旅游基地。鼓励企业开发老年特色旅游产品，拓展老年医疗旅游、老年观光旅游、老年乡村旅游等新业态。支持社会力量建设旅居养老旅游服务设施，结合各地自然禀赋，形成季节性地方推介目录，加强跨区域对接联动，打造旅居养老旅游市场。以健康状况取代年龄约束，修改完善相关规定。 　　九、营造老年友好型社会环境
　　（二十三）传承弘扬家庭孝亲敬老传统美德。
　　巩固和增强家庭养老功能。在全社会开展人口老龄化国情教育，积极践行社会主义核心价值观，传承弘扬“百善孝为先”的中华民族传统美德。建立常态化指导监督机制，督促赡养人履行赡养义务，防止欺老虐老弃老问题发生，将有能力赡养而拒不赡养老年人的违法行为纳入个人社会信用记录。支持地方制定具体措施，推动解决无监护人的特殊困难老年人监护保障问题。
　　完善家庭养老支持政策体系。将家庭照护者纳入养老护理员职业技能培训等范围，支持有关机构、行业协会开发公益课程并利用互联网平台等免费开放，依托基层群众性自治组织等提供指导，帮助老年人家庭成员提高照护能力。支持有条件的地区对分散供养特困人员中的高龄、失能、残疾老年人家庭实施居家适老化改造，配备辅助器具和防走失装置等设施设备。探索设立独生子女父母护理假制度。探索开展失能老年人家庭照护者“喘息服务”。

| 专栏8　中华孝亲敬老文化传承和创新工程 |
| --- |
| 每年在重阳节当月开展为期一个月的“敬老月”活动，广泛组织动员政府部门、社会组织、企事业单位和家庭个人，以走访慰问、权益维护、文化活动、志愿服务、主题宣传等多种方式，为老年人办实事、做好事、献爱心。 　　每年举办一次中华孝亲敬老文化传承和创新大会。持续开展全国“敬老文明号”创建和全国敬老爱老助老模范人物评选，营造养老孝老敬老社会氛围。 　　深入开展人口老龄化国情教育，增强全社会人口老龄化国情意识，推动形成积极应对人口老龄化广泛共识。 |

　　（二十四）推进公共环境无障碍和适老化改造。
　　提升社区和家庭适老化水平。有序推进城镇老旧小区改造，完成小区路面平整、出入口和通道无障碍改造、地面防滑处理等，在楼梯沿墙加装扶手，在楼层间安装挂壁式休息椅等，做好应急避险等安全防护。有条件的小区可建设凉亭、休闲座椅等。完善社区卫生服务中心、社区综合服务设施等的适老化改造。推动将适老化标准融入农村人居环境建设。鼓励有条件的地方对经济困难的失能、残疾、高龄等老年人家庭实施无障碍和适老化改造。
　　推动公共场所适老化改造。大力推进无障碍环境建设。加大城市道路、交通设施、公共交通工具等适老化改造力度，在机场、火车站、三级以上汽车客运站等公共场所为老年人设置专席以及绿色通道，加强对坡道、电梯、扶手等的改造，全面发展适老型智能交通体系，提供便捷舒适的老年人出行环境。推动街道乡镇、城乡社区公共服务环境适老化改造。
　　（二十五）建设兼顾老年人需求的智慧社会。
　　完善传统服务保障措施。对医疗、社保、民政、金融、电信、邮政、出入境、生活缴费等高频服务事项，设置必要的线下办事渠道并向基层延伸。公共服务场所应保留人工窗口和电话专线，为老年人保留一定数量的线下名额。加强身份证信息归集和数据互联互通，在更多领域推广“一证通行”。定期开展拒收现金专项治理。
　　推进智能化服务适应老年人需求。依托全国一体化政务服务平台，推进政务数据共享，优化线上线下政务服务，让老年人办事少跑腿。持续推进互联网网站、移动互联网应用适老化改造，优化界面交互、内容朗读、操作提示、语音辅助等功能，鼓励企业提供相关应用的“关怀模式”、“长辈模式”，将无障碍改造纳入日常更新维护。支持终端设备制造商、应用产品提供商、养老服务机构联动，促进上下游功能衔接。以市场力量为主体推动出台一批智能技术适老化改造标准。组织开展老年人运用智能技术教育培训，通过体验学习、尝试应用、经验交流、互助帮扶等，引导老年人了解新事物、体验新科技、运用新技术。严厉打击电信网络诈骗等违法犯罪行为。
　　长效解决“数字鸿沟”难题。发挥解决老年人运用智能技术困难工作部际联席会议制度作用，总结各地创新经验和举措，及时推广并适时形成政策文件。组织开展第三方评估，对各地公共服务适老化程度进行评价，相关结果纳入积极应对人口老龄化综合评估。

| 专栏9　智慧助老行动 |
| --- |
| 在全国城乡社区普遍开展老年人运用智能技术教育培训。研究编制一批老年人运用智能技术教育培训教材，鼓励老年人家庭成员、相关社会组织加强对老年人的培训。遴选培育一批智慧助老志愿服务团队，为老年人运用智能技术提供志愿培训和服务。加强智慧助老公益宣传，营造帮助老年人解决运用智能技术困难的良好氛围。 |

　　（二十六）培育敬老爱老助老社会风尚。
　　营造良好社会氛围。健全老年人权益保障机制，加强老龄法治建设，加大普法宣传教育力度。鼓励各地争创积极应对人口老龄化重点联系城市，开展全国示范性老年友好型社区创建活动，将老年友好型社会建设情况纳入文明城市评选的重要内容。加强老年人优待工作，鼓励各地推广与当地文化风俗、经济社会发展水平相适应的敬老爱老优待服务和活动。
　　积极发挥多方合力。建立健全为老志愿服务项目库，鼓励机构开发志愿服务项目，支持公益慈善类社会组织参与，引导在校生志愿服务和暑期实践、相关专业学生社会实习、社会爱心人士志愿服务等与老年人生活服务、健康服务、精神慰藉、法律援助等需求有效对接。围绕关爱老年人开展慈善募捐、慈善信托等慈善活动，依法加强对慈善组织和慈善活动的扶持和监管。 　　十、增强发展要素支撑体系
　　（二十七）推动有关培训疗养机构转型发展养老服务。
　　加大改革力度。按照“脱钩是原则、保留是例外”的要求，推动党政机关等所属培训疗养机构撤销或脱钩，资产统一划转至负责接收的国有企业，整合资源、统筹规划、整体转型。坚持“应改尽改、能转则转”的原则，推动党政机关、国有企事业单位所属培训疗养机构主要转型为普惠型养老服务设施，不得以养老名义经营其他业务。各地要建立绿色通道，本着尊重历史的原则，积极协调解决培训疗养机构转型问题。
　　强化示范引领。将培训疗养机构数量较多、分布集中的北京、大连、青岛、深圳、成都、杭州、秦皇岛、苏州、扬州、九江等确定为重点联系城市，支持更多符合条件的培训疗养机构转型，打造一批转型优质项目，纳入普惠养老专项行动，争取在2022年年底前基本投入运营。制定北戴河地区培训疗养机构转型发展养老服务规划，建设北戴河地区培训疗养机构转型发展养老服务集中示范区。
　　（二十八）完善用地用房支持政策。
　　科学规划布局新增用地。根据人口结构现状和老龄化发展趋势，因地制宜提出养老服务设施用地的规模、标准和布局。科学编制供地计划，分阶段供应规划确定的养老服务设施用地，并落实到年度建设用地供应计划，做到应保尽保。涉及新增建设用地的，在土地利用年度计划中优先予以安排。制定支持发展养老服务业的土地政策，以多种方式供应养老服务设施用地。
　　优化存量设施利用机制。在符合规划的前提下，支持利用存量场所改建养老服务设施，进一步简化和优化存量土地用途的变更程序。利用存量商业服务用地开展养老服务的，允许按照适老化设计要求适当放宽户均面积、租赁期限等土地和规划要求。养老服务机构所使用存量房屋在符合规划且不改变用地主体的条件下适用过渡期政策，五年内继续按原用途和权利类型使用土地。研究制定过渡期后顺畅接续的政策措施，稳定养老服务机构预期。出台支持依法利用集体建设用地发展养老服务的实施细则和工作指引，由养老服务机构与村集体约定土地使用和收益分配方案。
　　（二十九）强化财政资金和金融保障。
　　强化支持老龄事业发展和养老服务的资金保障。适应今后一段时期老龄事业发展的资金需求，完善老龄事业发展财政投入政策和多渠道筹资机制，继续加大中央预算内投资支持力度。民政部本级和地方各级政府用于社会福利事业的彩票公益金要加大倾斜力度，自2022年起将不低于55%的资金用于支持发展养老服务。鼓励地方在养老服务设施建设中同步考虑运营问题，确保后续发展可持续。各地要根据本地实际，研究制定可操作的运营补贴等激励政策，引导各类养老服务机构优先接收特殊困难老年人，鼓励对接收外地老年人的机构同等适用相应补贴政策。
　　推动税费优惠举措落地。落实落细支持养老服务发展的税费优惠政策。落实养老服务机构用电、用水、用气、用热享受居民价格政策，不得以土地、房屋性质等为理由拒绝执行相关价格政策，因难以计量等操作性原因无法执行的，探索应用大数据等技术手段予以解决。
　　拓宽金融支持养老服务渠道。鼓励金融机构按照市场化、法治化原则，提供差异化信贷支持，满足养老服务机构合理融资需求。鼓励探索以应收账款、动产、知识产权、股权等抵质押贷款，满足养老服务机构多样化融资需求。在依法合规、风险可控的前提下，审慎有序探索养老服务领域资产证券化，支持保险资金加大对养老服务业的投资力度，支持保险机构开发相关责任险及机构运营相关保险。
　　　十一、维护老年人合法权益
　　（三十一）加强市场主体行为监管。
　　落实市场主体信用承诺。建立健全养老服务机构备案信用承诺制度，备案申请人书面承诺养老服务机构按照有关法律法规和国家标准开展活动，书面承诺向社会公开，履约情况记入信用记录。督促养老服务机构落实主体责任，主动防范消除本机构在建筑、消防、食品、医疗卫生等方面的风险隐患，提高养老服务、安全管理、风险防控的能力和水平。
　　加强市场秩序监管。对未依法取得营业执照以市场主体名义从事养老服务经营活动、未经登记擅自以社会服务机构名义开展养老服务活动、未经登记管理机关核准登记擅自以事业单位法人名义开展养老服务活动等无证无照违法经营行为，加大依法打击查处力度。严禁利用养老服务机构设施和场地开展与养老服务无关的活动。指导养老服务机构按照国家有关规定和当事方协议约定提供服务，建立纠纷协商调解机制，引导老年人及其代理人依法维权。
　　（三十二）引领全行业规范健康发展。
　　健全养老服务综合监管制度。加强协同监管，健全各部门协调配合机制，实现违法线索互联、监管标准互通、处理结果互认，避免多头多层重复执法，切实减轻养老服务机构和从业人员负担。加强对养老服务机构的行为监管，严防欺老虐老行为。利用大数据分析等多种手段，创新开展智能监管，推动行业自律。建立“养老服务＋信用”机制，充分运用全国信用信息共享平台、国家企业信用信息公示系统、中国社会组织政务服务平台，建立覆盖养老服务机构、从业人员的信用管理体系。
　　优化养老服务营商环境。完善养老机构备案办事指南，优化办事流程，实施并联服务，明确办理时限，推进“马上办、网上办、就近办”。制定养老服务领域政务服务事项清单，建立健全“好差评制度”，持续改进提升政务服务质量。推进要素市场制度建设，实现要素价格市场决定、流动自主有序、配置高效公平。
　　推进养老服务标准化建设。加快养老服务领域标准的制修订，研究制定一批与国际接轨、体现中国特色、适应服务管理需要的养老服务标准。加快建立全国统一的养老服务质量标准、等级评定与认证体系，推动养老机构服务安全基本规范、服务质量基本规范、等级划分与评定等国家标准的实施，引导养老服务机构通过养老服务质量认证。鼓励各地因地制宜制定养老服务相关地方标准，鼓励社会组织自主制定高于国家标准、行业标准技术要求的养老服务相关团体标准。积极参与养老服务领域国际标准化活动。支持养老服务领域行业组织和机构开展标准化管理。
　　（三十三）加强老年人消费权益保护。
　　切实防范各类侵权风险。加大联合执法力度，严厉查处老年人产品和服务消费领域的侵权行为，特别是向老年人欺诈销售各类产品和服务的违法行为。广泛开展老年人识骗防骗宣传教育活动，提升老年人抵御欺诈销售的意识和能力。加大养老诈骗重点防范和整治工作力度，做好政策宣传和风险提示，对涉嫌犯罪的依法打击。完善养老服务领域预付费管理制度，探索建立对预付费的资金监管机制。加强对金融机构开展养老服务领域金融产品和服务创新的监管。完善养老服务机构退出机制，指导退出机构妥善做好老年人服务协议解除、安置等工作，建立健全养老服务机构关停等特殊情况应急处置机制。
　　加强涉老矛盾纠纷化解和法律援助。充分发挥基层党组织、基层群众性自治组织、相关社会组织的作用，做好涉老矛盾纠纷预警、排查、化解。建立适老型诉讼服务机制。倡导律师事务所、公证机构、基层法律服务机构为老年人减免法律服务费用，为行动不便的老年人提供上门服务。做好特殊困难老年人的法律服务、法律援助和司法救助。完善老年人监护制度。
　　规范中高端机构养老发展。对建设、销售以老年人为主要居住群体的住宅或居住小区，要坚持以服务为本的功能定位，鼓励地方建立监管机制，落实信用承诺，强化日常监管，确保经营健康稳定可持续，严禁以养老之名“跑马圈地”。 　　十二、实施保障
　　（三十四）加强党的领导。
　　坚持党的集中统一领导，充分发挥党总揽全局、协调各方的领导核心作用，为规划实施提供坚强保障。强化各地落实规划的主体责任，加强对规划实施的组织、协调和督导，将本规划主要任务指标纳入当地经济社会发展规划，纳入为民办实事项目，纳入政府工作议事日程和目标责任考核内容。
　　（三十五）完善法治保障。
　　落实依法治国要求，依法保障老年人合法权益，推动制定养老服务法，构建以老年人权益保障、养老服务等法律为统领，行政法规、部门规章、规范性文件为主体，相关标准为支撑的养老服务政策法律体系，实现养老服务有法可依、有法必依。发挥养老服务法规在保护当事人权益、维护市场秩序、规范合同管理、调解处理服务纠纷等方面的重要作用。
　　（三十六）强化组织协调。
　　各省（自治区、直辖市）要根据人口老龄化发展形势，制定实施专项规划，加强与相关规划衔接。各级老龄工作委员会要发挥统筹协调作用，推动老龄工作委员会各成员单位履职尽责，形成工作合力。发挥养老服务联席会议制度作用，推进养老服务体系建设，强化区域养老服务资源统筹管理。支持城市群、都市圈打造养老服务体系一体化建设格局，形成服务能力衔接、产业发展协同的合作区域。支持大型城市和区域中心城市推动养老产业集聚发展，充分发挥辐射带动和示范作用。推动以地级行政区为单位制定“整体解决方案”，将老龄事业发展和养老服务体系建设纳入经济社会发展全局中通盘考虑，全方位整合资源力量，充分调动各方积极性，推动兜底性、普惠型、多样化三种路径协同发展。

**全民科学素质行动规划纲要(2021—2035年)的通知**

　　（四）老年人科学素质提升行动。
　　以提升信息素养和健康素养为重点，提高老年人适应社会发展能力，增强获得感、幸福感、安全感，实现老有所乐、老有所学、老有所为。
　　--实施智慧助老行动。聚焦老年人运用智能技术、融入智慧社会的需求和困难，依托老年大学（学校、学习点）、老年科技大学、社区科普大学、养老服务机构等，普及智能技术知识和技能，提升老年人信息获取、识别和使用能力，有效预防和应对网络谣言、电信诈骗。
　　--加强老年人健康科普服务。依托健康教育系统，推动老年人健康科普进社区、进乡村、进机构、进家庭，开展健康大讲堂、老年健康宣传周等活动，利用广播、电视、报刊、网络等各类媒体，普及合理膳食、食品安全、心理健康、体育锻炼、合理用药、应急处置等知识，提高老年人健康素养。充分利用社区老年人日间照料中心、科普园地、党建园地等阵地为老年人提供健康科普服务。
　　--实施银龄科普行动。积极开发老龄人力资源，大力发展老年协会、老科协等组织，充分发挥老专家在咨询、智库等方面的作用。发展壮大老年志愿者队伍。组建老专家科普报告团，在社区、农村、青少年科普中发挥积极作用。
　　　--实施智慧科普建设工程。推进科普与大数据、云计算、人工智能、区块链等技术深度融合，强化需求感知、用户分层、情景应用理念，推动传播方式、组织动员、运营服务等创新升级，加强“科普中国”建设，充分利用现有平台构建国家级科学传播网络平台和科学辟谣平台。强化科普信息落地应用，与智慧教育、智慧城市、智慧社区等深度融合，推动优质科普资源向革命老区、民族地区、边疆地区、脱贫地区倾斜。
　　（三）科普基础设施工程。
　　加强科普基础设施建设，建立政府引导、多渠道投入的机制，实现资源合理配置和服务均衡化、广覆盖。
　　--加强对科普基础设施建设的统筹规划与宏观指导。制定科普基础设施发展规划，将科普基础设施建设纳入各地国民经济和社会发展规划。完善科普基础设施建设管理的规范和标准，建立健全分级评价制度。完善社会资金投入科普基础设施建设的优惠政策和法规。推行科技馆登记注册制度和年报制度。推进符合条件的科技馆免费开放。
　　--创新现代科技馆体系。推动科技馆与博物馆、文化馆等融合共享，构建服务科学文化素质提升的现代科技馆体系。加强实体科技馆建设，开展科普展教品创新研发，打造科学家精神教育基地、前沿科技体验基地、公共安全健康教育基地和科学教育资源汇集平台，提升科技馆服务功能。推进数字科技馆建设，统筹流动科技馆、科普大篷车、农村中学科技馆建设，探索多元主体参与的运行机制和模式，提高服务质量和能力。
　　--大力加强科普基地建设。深化全国科普教育基地创建活动，构建动态管理和长效激励机制。鼓励和支持各行业各部门建立科普教育、研学等基地，提高科普服务能力。推进图书馆、文化馆、博物馆等公共设施开展科普活动，拓展科普服务功能。引导和促进公园、自然保护区、风景名胜区、机场、车站、电影院等公共场所强化科普服务功能。开发利用有条件的工业遗产和闲置淘汰生产设施，建设科技博物馆、工业博物馆、安全体验场馆和科普创意园。
　
　　--健全基层科普服务体系。构建省域统筹政策和机制、市域构建资源集散中心、县域组织落实，以新时代文明实践中心（所、站）、党群服务中心、社区服务中心（站）等为阵地，以志愿服务为重要手段的基层科普服务体系。动员学校、医院、科研院所、企业、科学共同体和社会组织等组建科技志愿服务队，完善科技志愿服务管理制度，推进科技志愿服务专业化、规范化、常态化发展，推广群众点单、社区派单、部门领单、科技志愿服务队接单的订单认领模式。建立完善跨区域科普合作和共享机制，鼓励有条件的地区开展全领域行动、全地域覆盖、全媒体传播、全民参与共享的全域科普行动。
　　（五）科学素质国际交流合作工程。
　　拓展科学素质建设交流渠道，搭建开放合作平台，丰富交流合作内容，增进文明互鉴，推动价值认同，提升开放交流水平，参与全球治理。
　　--拓展国际科技人文交流渠道。围绕提升科学素质、促进可持续发展，充分发挥科学共同体优势和各类人文交流机制作用。开展青少年交流培育计划，拓展合作领域，提升合作层次。
　　--丰富国际合作内容。深入开展科学教育、传播和普及双多边合作项目，促进科普产品交流交易。聚焦应对未来发展、粮食安全、能源安全、人类健康、灾害风险、气候变化等人类可持续发展共同挑战，加强青少年、妇女和教育、媒体、文化等领域科技人文交流。
　　--积极参与全球治理。推进科学素质建设国际合作，探索制订国际标准，推动建立世界公众科学素质组织，参与议题发起和设置，在多边活动中积极提供中国方案、分享中国智慧。
　　--促进“一带一路”科技人文交流。坚持共商共建共享原则，深化公共卫生、绿色发展、科技教育等领域合作。推进科学素质建设战略、规划、机制对接，加强政策、规则、标准联通，推动共建“一带一路”高质量发展。

五、组织实施
　　（一）组织保障。
　　建立完善《科学素质纲要》实施协调机制，负责领导《科学素质纲要》实施工作，将公民科学素质发展目标纳入国民经济和社会发展规划，加强对《科学素质纲要》实施的督促检查。各部门将《科学素质纲要》有关任务纳入相关规划和计划，充分履行工作职责。中国科协发挥综合协调作用，做好沟通联络工作，会同各有关方面共同推进科学素质建设。
　　地方各级政府负责领导当地《科学素质纲要》实施工作，把科学素质建设作为地方经济社会发展的一项重要任务，纳入本地区总体规划，列入年度工作计划，纳入目标管理考核。地方各级科协牵头实施《科学素质纲要》，完善科学素质建设工作机制，会同各相关部门全面推进本地区科学素质建设。
　　（二）机制保障。
　　完善表彰奖励机制。根据国家有关规定，对在科学素质建设中做出突出贡献的集体和个人给予表彰和奖励。
　　完善监测评估体系。完善科普工作评估制度，制定新时代公民科学素质标准，定期开展公民科学素质监测评估、科学素质建设能力监测评估。
　　（三）条件保障。
　　完善法规政策。完善科普法律法规体系，鼓励有条件的地方制修订科普条例，制定科普专业技术职称评定办法，开展评定工作，将科普人才列入各级各类人才奖励和资助计划。
　　加强理论研究。围绕新科技、新应用带来的科技伦理、科技安全、科学谣言等方面，开展科学素质建设理论与实践研究。深入开展科普对象、手段和方法等研究，打造科学素质建设高端智库。
　　强化标准建设。分级分类制定科普产品和服务标准，实施科学素质建设标准编制专项，推动构建包括国家标准、行业标准、地方标准、团体标准和企业标准的多维标准体系。
　　保障经费投入。各有关部门统筹考虑和落实科普经费。各级政府按规定安排经费支持科普事业发展。大力提倡个人、企业、社会组织等社会力量采取设立科普基金、资助科普项目等方式为科学素质建设投入资金。

**教育部办公厅关于广泛开展老年人运用智能技术教育培训的通知**

　　一、总体目标
　　充分发挥教育培训在帮助老年人运用智能技术中的作用，通过广泛开展惠及老年人的智能技术应用培训，促进老年人更新观念，提高老年人运用智能技术能力，助力解决老年人在出行、就医、消费等日常生活中遇到的实际困难，使老年人愿用、能用、乐用智能技术，为老年人跨越“数字鸿沟”提供教育支持服务，共享智慧社会带来的便利性、快捷性和智能性，不断增强老年人的获得感、幸福感和安全感。

二、主要举措
　　1.加强政策引导。各地教育行政部门要将“开展老年人智能技术教育、加强应用培训”作为社区教育、老年教育的一项重要内容，列入“十四五”教育相关规划和年度工作计划等政策文件，强化政策引导与统筹协调。
　　2.纳入全民终身学习活动周。各地教育行政部门要将“智慧助老”作为全民终身学习活动周的一项重要活动，广泛开展老年人运用智能技术教育培训，服务老年人终身学习。要深度挖掘、广泛宣传在开展老年人运用智能技术教育培训中的好经验、好做法、好典型，组织开展经验交流，推广优秀工作案例。
　　3.就近开展教育培训。社区教育、老年教育机构要充分发挥扎根社区、贴近居民等优势，紧紧围绕老年人在日常生活中运用智能技术所遇到的高频事项和应用场景，聚焦实际问题和突出困难，做好需求分析和问题梳理，将学习场景与生活场景有机融合，倡导学中用、用中学，激发老年人学习兴趣，主动搭建平台，通过邀请行业专家等方式开展专题培训，帮助老年人在智慧出行、智慧医疗、掌上金融、手机购物等领域的实际生活中应用所学技能，切实提高教育培训实效。
　　4.发挥开放大学体系作用。开放大学体系要发挥终身教育重要平台作用，创新线上线下相融合的老年人运用智能技术教育培训模式，鼓励各终身学习（老年教育）平台开设“老年人运用智能技术”专栏，为老年人提供灵活便利的学习平台和优质丰富的学习课程，大力开展教育培训与应用推广。遵循贴近生活、图文并茂、简单易学等原则，征集、开发“互联网＋生活”“智能手机应用”“智慧生活”等体现适老化和场景化的全媒体课程资源。到2022年，依托国家开放大学组织推介“智慧助老”专题的优质工作案例100个、教育培训项目200个、课程资源500门。
　　5.支持院校强化助老服务。有条件的普通高校、职业院校、成人高校要发挥师资、专业、资源等优势，组织引导教师按照适老化、便利化、个性化原则，积极参与面向老年人的智能技术应用培训，通过师资输送、开展老年人运用智能技术相关师资培训、视频公开课等方式，扩大优质课程资源共享，为有效提升老年人运用智能技术提供支持服务。
　　6.鼓励社会多元主体参与。鼓励社区教育和老年教育机构面向社会选聘能者达人作为兼职教师或志愿者，积极动员社会公益组织、行业专家等主体参与老年人智能技术应用培训，通过举办应用讲座、送教进社区、个别辅导等方式，助力老年人有效提升运用智能技术的能力。倡导在家庭环境中助老助学，弘扬孝老爱亲文化和关爱老年人的社会风尚。

**老专家服务基层健康行动方案**

　　为充分挖掘三级医院优秀退休医务人员（以下简称老专家）的人力资源，提高医疗资源相对薄弱地区基层医疗卫生机构的服务水平，促进当地基层医疗卫生机构能力建设，建立老专家定期援助基层医疗卫生机构机制，国家卫生健康委和国家中医药局决定开展“老专家服务基层健康行动”。
　　一、行动目标
　　针对部分地区基层医疗卫生机构紧缺的医学专业，组织一批老专家对口支援，发挥老专家的经验优势和专业优势，通过门诊、带教等方式提高相关地区基层医疗卫生机构医疗服务能力，满足当地人民群众卫生健康需求。

二、行动原则
　　（一）统筹谋划，多方配合。行动由国家卫生健康委、国家中医药局统筹谋划，各相关地方卫生健康行政部门、基层医疗卫生机构和派员医院等多方协调配合，按照各自职责，共同推进。
　　（二）自愿参加，工休结合。老专家自愿报名参加并进行遴选，与受援机构协商确定服务时间、补助等相关细节，受援地卫生健康行政部门和受援机构结合当地实际安排老专家业余休养活动。
　　（三）试点先行，逐步推广。老专家以国家卫生健康委属（管）医院和国家中医药局属（管）医院退休医务人员为主，鼓励北京市有关医院退休医务人员积极参与，选择部分省份基层医疗卫生机构进行试点，在总结经验的基础上，逐步向其他地方三级医院和其他省份延伸。

三、行动范围与内容
　　（一）行动范围。2021年，活动范围为辽宁省、吉林省、福建省、广西壮族自治区、海南省、贵州省、云南省、宁夏回族自治区、新疆维吾尔自治区的部分县域医共体或基层医疗卫生机构，受援机构应当具备基本医疗卫生服务能力，可以提供老专家开展业务所必须的场地、设备和辅助人员等条件。
　　（二）资格条件。国家卫生健康委属（管）医院、国家中医药局属（管）医院以及北京市有关医院自愿报名的老专家年龄应在70岁（含）周岁以下（身体情况较好者可适当放宽年龄要求），已退休，具有副高及以上职称，临床经验丰富、医德高尚、身体健康、甘于奉献。
　　（三）主要工作内容。
　　1.老专家在受援机构以日常门诊、临床教学、课题研究、科室建设指导为主，短期授课、远程会诊、学术讲座（报告）为辅，采取传、帮、带等方式，提高受援机构诊疗和科研水平。充分发挥中医药特色和优势，提高基层地区中医药服务能力。
　　2.老专家每个支援周期时间原则上不少于1个月，每周门诊或住院查房教学时间不少于4次（每次半天），参与指导1项课题研究，组织开展若干学术讲座、手术示教等活动。具体内容由老专家和受援机构协商确定，并在协议中明确。 　　四、各方职责
　　（一）国家卫生健康委和国家中医药局。国家卫生健康委和国家中医药局作为“老专家服务基层健康行动”的主办方，负责行动规划指导、跟踪、督促，提出工作目标和要求，确定行动范围和内容，适时通报行动进展情况。
　　（二）省级和市级卫生健康行政部门、中医药主管部门。各有关省份卫生健康委负责具体实施“老专家服务基层健康行动”，明确责任处室负责行动实施过程中的管理、协调、监督等工作；参照省内三级甲等医院同级别人员薪酬待遇水平，确定老专家在受援机构获得补助的参考标准；协调省内媒体进行宣传报道，深入挖掘老专家中先进典型和感人事迹。省级中医药主管部门配合省级卫生健康行政部门做好中医医院老专家服务基层健康行动的具体实施工作，每年4月底前填报本年度本省份岗位需求信息。北京市卫生健康委负责动员市内有关医院老专家参与本次活动。
　　各有关市（州）卫生健康行政部门负责省级、县级卫生健康行政部门之间的沟通协调，协助县级卫生健康行政部门解决项目实施过程中遇到的问题。
　　（三）县（区）卫生健康行政部门。各有关县（区）卫生健康行政部门负责协调落实各项保障措施；在本县（区）范围内进行广泛宣传，引导患者积极前往受援机构问医求药，充分激发老专家服务人民、甘于奉献的精神，使老专家在提供服务同时，能够体会到自我价值的实现；制定老专家援助期间突发疾病救治工作预案。
　　（四）受援机构。受援机构应当结合实际提出需求，制定具体工作方案，指定专人负责本机构方案实施和协调管理工作；与老专家签订服务协议，明确双方权利和义务，保障老专家工作场所，落实老专家工作补助，营造良好的工作氛围；在受援机构内进行宣传，鼓励单位职工抓住机遇积极求教，不仅要学习老专家精湛的医疗技术，还要学习老专家高尚的医德医风。
　　（五）派员医院。派员医院应当将本项工作纳入医院年度工作任务，统筹部署。每年5月底前填报本年度院内老专家参与人员信息。在医院全体范围积极宣传，弘扬老专家乐于奉献的精神，倡导积极向上的退休生活模式。
　　（六）卫人就业网。国家卫生健康委人才交流服务中心下属“卫人就业网”（网址：www.weirenjob.com）作为“老专家服务基层健康行动”的承办方，负责老专家服务基层健康行动信息服务平台的开发、建设、运维和技术培训；管理、维护受援机构库和专家库信息；根据受援机构需求和专家申报信息，提出专家援助匹配方案报国家卫生健康委和国家中医药局审定；根据工作委托，跟踪、联系、协调、督促行动顺利开展。

五、老专家遴选程序
　　（一）受援机构提出需求，逐级审核由省级卫生健康行政部门汇总后，报国家卫生健康委审定。
　　（二）医院组织动员老专家按受援机构需求报名，在卫人就业网的“老专家服务基层健康行动信息服务平台”录入老专家信息并申报。
　　（三）卫人就业网对申报人员进行资格审核或遴选。
　　（四）卫人就业网根据需求和申报志愿，按照专业相符、年龄优先等原则进行匹配，提出匹配方案报国家卫生健康委基层司、国家中医药局医政司（中西医结合与民族医药司）审定。
　　（五）匹配方案审定后，卫人就业网通知受援机构与匹配的老专家进行联系，协商服务起始时间、待遇保障等。
　　（六）受援机构、老专家签订服务协议。
　　（七）老专家上岗服务。

六、保障措施
　　（一）政策保障。
　　1.老专家援助期间与派员医院的人事关系和退休待遇不变。
　　2.各有关省份卫生健康行政部门协调解决老专家跨地区执业注册，确保老专家合法合规开展各项医疗服务活动。省级卫生健康行政部门要加强监督，对行动开展情况进行监督检查。
　　3.受援机构应当为老专家提供必要的医疗设备、工作场所等，确保各项工作正常开展；应当提供舒适的居住场所，配备必要的居住设施，保障老专家援助期间工作和生活安心。
　　4.派员医院应当配合老专家开展远程会诊，提供必要的设备设施以及做好后勤保障工作。
　　5.老专家援助期间因病因伤发生医疗费用，按本人医疗关系和有关规定办理；受援机构和老专家双方可通过商业医疗保险等方式提供补充支持；派员医院根据相关制度，给予探望慰问等待遇。
　　（二）经费保障。
　　1.受援机构根据自身财政状况，参考省级标准给予老专家工作补助，具体金额由受援机构和老专家双方协商议定。受援机构不能给予薪酬补助的，也应当与老专家协商议定。
　　2.老专家开展学术讲座和报告等发生的劳务费或咨询费参照受援机构内部标准执行。
　　3.受援机构所在县（区）卫生健康行政部门应当协调解决老专家食宿、岗前培训费、派出地往返交通差旅费、当地交通费、人身意外保险费、必要的商业医疗保险费以及业余休养等保障性经费。
　　4.派员医院承担老专家派出前的体检费用。
　　5.行动开展过程中，为解决经费问题，行动各方可以适当引入社会资金参与。七、总结评价
　　（一）评价管理。
　　1.受援机构对老专家评价。援助期限结束后，受援机构应当对老专家进行评价，评价内容主要包括：工作时间情况、临床工作开展情况、科研教学情况、技术培训情况、患者评价等。评价结果作为老专家今后参加遴选的重要评判依据。
　　2.老专家对受援机构评价。援助期限结束后，老专家应当对受援机构进行评价，评价内容主要包括：是否提供必要的工作场所、各项待遇保障落实情况、申报需求专业是否合理等。评价结果作为受援机构今后是否分配老专家名额的重要评判依据。
　　（二）总结评估。每年度行动结束后，各省级卫生健康行政部门要对行动进行年度考核评估，并于第二年3月底前将考核结果形成年度评估报告报送国家卫生健康委基层司和国家中医药局医政司（中西医结合与民族医药司）。评估内容以行动执行情况、实施效果、受援医院的评价以及政府、社会和群众的评价为重点。具体内容包括：工作机制建立情况，政策制度制定情况，政策保障落实情况、援助成效情况、患者评价等。国家卫生健康委和国家中医药局在各受援机构评价、各省级卫生健康行政部门评估的基础上，对于表现优秀、做出重大贡献的老专家予以表扬。

**中共中央 国务院
关于加强新时代老龄工作的意见**

有效应对我国人口老龄化，事关国家发展全局，事关亿万百姓福祉，事关社会和谐稳定，对于全面建设社会主义现代化国家具有重要意义。为实施积极应对人口老龄化国家战略，加强新时代老龄工作，提升广大老年人的获得感、幸福感、安全感，现提出如下意见。

**一、总体要求**

（一）指导思想。以习近平新时代中国特色社会主义思想为指导，深入贯彻党的十九大和十九届二中、三中、四中、五中、六中全会精神，加强党对老龄工作的全面领导，坚持以人民为中心，将老龄事业发展纳入统筹推进“五位一体”总体布局和协调推进“四个全面”战略布局，实施积极应对人口老龄化国家战略，把积极老龄观、健康老龄化理念融入经济社会发展全过程，加快建立健全相关政策体系和制度框架，大力弘扬中华民族孝亲敬老传统美德，促进老年人养老服务、健康服务、社会保障、社会参与、权益保障等统筹发展，推动老龄事业高质量发展，走出一条中国特色积极应对人口老龄化道路。

（二）工作原则

——坚持党委领导、各方参与。在党委领导下，充分发挥政府在推进老龄事业发展中的主导作用，社会参与，全民行动，提供基本公益性产品和服务。充分发挥市场机制作用，提供多元化产品和服务。注重发挥家庭养老、个人自我养老的作用，形成多元主体责任共担、老龄化风险梯次应对、老龄事业人人参与的新局面。

——坚持系统谋划、综合施策。坚持应对人口老龄化和促进经济社会发展相结合，坚持满足老年人需求和解决人口老龄化问题相结合，确保各项政策制度目标一致、功能协调、衔接配套，努力实现老有所养、老有所医、老有所为、老有所学、老有所乐，让老年人共享改革发展成果、安享幸福晚年。

——坚持整合资源、协调发展。构建居家社区机构相协调、医养康养相结合的养老服务体系和健康支撑体系，大力发展普惠型养老服务，促进资源均衡配置。推动老龄事业与产业、基本公共服务与多样化服务协调发展，统筹好老年人经济保障、服务保障、精神关爱、作用发挥等制度安排。

——坚持突出重点、夯实基层。聚焦解决老年人健康养老最紧迫的问题，坚持保基本、促公平、提质量，尽力而为、量力而行，确保人人享有基本养老服务和公共卫生服务。推动老龄工作重心下移、资源下沉，推进各项优质服务资源向老年人的身边、家边和周边聚集，确保老龄工作有人抓、老年人事情有人管、老年人困难有人帮。

**二、健全养老服务体系**

（三）创新居家社区养老服务模式。以居家养老为基础，通过新建、改造、租赁等方式，提升社区养老服务能力，着力发展街道（乡镇）、城乡社区两级养老服务网络，依托社区发展以居家为基础的多样化养老服务。地方政府负责探索并推动建立专业机构服务向社区、家庭延伸的模式。街道社区负责引进助餐、助洁等方面为老服务的专业机构，社区组织引进相关护理专业机构开展居家老年人照护工作；政府加强组织和监督工作。政府要培育为老服务的专业机构并指导其规范发展，引导其按照保本微利原则提供持续稳定的服务。充分发挥社区党组织作用，探索“社区+物业+养老服务”模式，增加居家社区养老服务有效供给。结合实施乡村振兴战略，加强农村养老服务机构和设施建设，鼓励以村级邻里互助点、农村幸福院为依托发展互助式养老服务。

（四）进一步规范发展机构养老。各地要通过直接建设、委托运营、购买服务、鼓励社会投资等多种方式发展机构养老。加强光荣院建设。公办养老机构优先接收经济困难的失能（含失智，下同）、孤寡、残疾、高龄老年人以及计划生育特殊家庭老年人、为社会作出重要贡献的老年人，并提供符合质量和安全标准的养老服务。建立健全养老服务标准和评价体系，加强对养老机构建设和运营的监管。研究制定养老机构预收服务费用管理政策，严防借养老机构之名圈钱、欺诈等行为。

（五）建立基本养老服务清单制度。各地要根据财政承受能力，制定基本养老服务清单，对健康、失能、经济困难等不同老年人群体，分类提供养老保障、生活照料、康复照护、社会救助等适宜服务。清单要明确服务对象、服务内容、服务标准和支出责任，并根据经济社会发展和科技进步进行动态调整。2022年年底前，建立老年人能力综合评估制度，评估结果在全国范围内实现跨部门互认。

（六）完善多层次养老保障体系。扩大养老保险覆盖面，逐步实现基本养老保险法定人员全覆盖。尽快实现企业职工基本养老保险全国统筹。健全基本养老保险待遇调整机制，保障领取待遇人员基本生活。大力发展企业（职业）年金，促进和规范发展第三支柱养老保险。探索通过资产收益扶持制度等增加农村老年人收入。

**三、完善老年人健康支撑体系**

（七）提高老年人健康服务和管理水平。在城乡社区加强老年健康知识宣传和教育，提升老年人健康素养。做好国家基本公共卫生服务项目中的老年人健康管理和中医药健康管理服务。加强老年人群重点慢性病的早期筛查、干预及分类指导，开展老年口腔健康、老年营养改善、老年痴呆防治和心理关爱行动。提高失能、重病、高龄、低收入等老年人家庭医生签约服务覆盖率，提高服务质量。扩大医联体提供家庭病床、上门巡诊等居家医疗服务的范围，可按规定报销相关医疗费用，并按成本收取上门服务费。积极发挥基层医疗卫生机构为老年人提供优质中医药服务的作用。加强国家老年医学中心建设，布局若干区域老年医疗中心。加强综合性医院老年医学科建设，2025年二级及以上综合性医院设立老年医学科的比例达到60%以上。通过新建改扩建、转型发展，加强老年医院、康复医院、护理院（中心、站）以及优抚医院建设，建立医疗、康复、护理双向转诊机制。加快建设老年友善医疗机构，方便老年人看病就医。

（八）加强失能老年人长期照护服务和保障。完善从专业机构到社区、家庭的长期照护服务模式。按照实施国家基本公共卫生服务项目的有关要求，开展失能老年人健康评估与健康服务。依托护理院（中心、站）、社区卫生服务中心、乡镇卫生院等医疗卫生机构以及具备服务能力的养老服务机构，为失能老年人提供长期照护服务。发展“互联网+照护服务”，积极发展家庭养老床位和护理型养老床位，方便失能老年人照护。稳步扩大安宁疗护试点。稳妥推进长期护理保险制度试点，指导地方重点围绕进一步明确参保和保障范围、持续健全多元筹资机制、完善科学合理的待遇政策、健全待遇支付等相关标准及管理办法、创新管理和服务机制等方面，加大探索力度，完善现有试点，积极探索建立适合我国国情的长期护理保险制度。

（九）深入推进医养结合。卫生健康部门与民政部门要建立医养结合工作沟通协调机制。鼓励医疗卫生机构与养老机构开展协议合作，进一步整合优化基层医疗卫生和养老资源，提供医疗救治、康复护理、生活照料等服务。支持医疗资源丰富地区的二级及以下医疗机构转型，开展康复、护理以及医养结合服务。鼓励基层积极探索相关机构养老床位和医疗床位按需规范转换机制。根据服务老年人的特点，合理核定养老机构举办的医疗机构医保限额。2025年年底前，每个县（市、区、旗）有1所以上具有医养结合功能的县级特困人员供养服务机构。符合条件的失能老年人家庭成员参加照护知识等相关职业技能培训的，按规定给予职业培训补贴。创建一批医养结合示范项目。

**四、促进老年人社会参与**

（十）扩大老年教育资源供给。将老年教育纳入终身教育体系，教育部门牵头研究制定老年教育发展政策举措，采取促进有条件的学校开展老年教育、支持社会力量举办老年大学（学校）等办法，推动扩大老年教育资源供给。鼓励有条件的高校、职业院校开设老年教育相关专业和课程，加强学科专业建设与人才培养。编写老年教育相关教材。依托国家开放大学筹建国家老年大学，搭建全国老年教育资源共享和公共服务平台。创新机制，推动部门、行业企业、高校举办的老年大学面向社会开放办学。发挥社区党组织作用，引导老年人践行积极老龄观。

（十一）提升老年文化体育服务质量。各地要通过盘活空置房、公园、商场等资源，支持街道社区积极为老年人提供文化体育活动场所，组织开展文化体育活动，实现老年人娱乐、健身、文化、学习、消费、交流等方面的结合。培养服务老年人的基层文体骨干，提高老年人文体活动参与率和质量，文化和旅游、体育等部门要做好规范和管理工作。开发老年旅游产品和线路，提升老年旅游服务质量和水平。县（市、区、旗）应整合现有资源，设置适宜老年人的教育、文化、健身、交流场所。

（十二）鼓励老年人继续发挥作用。把老有所为同老有所养结合起来，完善就业、志愿服务、社区治理等政策措施，充分发挥低龄老年人作用。在学校、医院等单位和社区家政服务、公共场所服务管理等行业，探索适合老年人灵活就业的模式。鼓励各地建立老年人才信息库，为有劳动意愿的老年人提供职业介绍、职业技能培训和创新创业指导服务。深入开展“银龄行动”，引导老年人以志愿服务形式积极参与基层民主监督、移风易俗、民事调解、文教卫生等活动。发挥老年人在家庭教育、家风传承等方面的积极作用。加强离退休干部职工基层党组织建设，鼓励老党员将组织关系及时转入经常居住地，引导老党员结合自身实际发挥作用，做好老年人精神关爱和思想引导工作。全面清理阻碍老年人继续发挥作用的不合理规定。

**五、着力构建老年友好型社会**

（十三）加强老年人权益保障。各地在制定涉及老年人利益的具体措施时，应当征求老年人的意见。建立完善涉老婚姻家庭、侵权等矛盾纠纷的预警、排查、调解机制。加强老年人权益保障普法宣传，提高老年人运用法律手段保护权益意识，提升老年人识骗防骗能力，依法严厉打击电信网络诈骗等违法犯罪行为。完善老年人监护制度。倡导律师事务所、公证机构、基层法律服务机构为老年人减免法律服务费用，为行动不便的老年人提供上门服务。建立适老型诉讼服务机制，为老年人便利参与诉讼活动提供保障。

（十四）打造老年宜居环境。各地要落实无障碍环境建设法规、标准和规范，将无障碍环境建设和适老化改造纳入城市更新、城镇老旧小区改造、农村危房改造、农村人居环境整治提升统筹推进，让老年人参与社会活动更加安全方便。鼓励有条件的地方对经济困难的失能、残疾、高龄等老年人家庭，实施无障碍和适老化改造、配备生活辅助器具、安装紧急救援设施、开展定期探访。指导各地结合实际出台家庭适老化改造标准，鼓励更多家庭开展适老化改造。在鼓励推广新技术、新方式的同时，保留老年人熟悉的传统服务方式，加快推进老年人常用的互联网应用和移动终端、APP应用适老化改造。实施“智慧助老”行动，加强数字技能教育和培训，提升老年人数字素养。

（十五）强化社会敬老。深入开展人口老龄化国情教育。实施中华孝亲敬老文化传承和创新工程。持续推进“敬老月”系列活动和“敬老文明号”创建活动，结合时代楷模、道德模范等评选，选树表彰孝亲敬老先进典型。将为老志愿服务纳入中小学综合实践活动和高校学生实践内容。加强老年优待工作，在出行便利、公交乘车优惠、门票减免等基础上，鼓励有条件的地方进一步拓展优待项目、创新优待方式，在醒目位置设置老年人优待标识，推广老年人凭身份证等有效证件享受各项优待政策。有条件的地方要积极落实外埠老年人同等享受本地优待项目。发挥广播电视和网络视听媒体作用，加强宣传引导，营造良好敬老社会氛围。

**六、积极培育银发经济**

（十六）加强规划引导。编制相关专项规划，完善支持政策体系，统筹推进老龄产业发展。鼓励各地利用资源禀赋优势，发展具有比较优势的特色老龄产业。统筹利用现有资金渠道支持老龄产业发展。

（十七）发展适老产业。相关部门要制定老年用品和服务目录、质量标准，推进养老服务认证工作。各地要推动与老年人生活密切相关的食品、药品以及老年用品行业规范发展，提升传统养老产品的功能和质量，满足老年人特殊需要。企业和科研机构要加大老年产品的研发制造力度，支持老年产品关键技术成果转化、服务创新，积极开发适合老年人使用的智能化、辅助性以及康复治疗等方面的产品，满足老年人提高生活品质的需求。鼓励企业设立线上线下融合、为老年人服务的专柜和体验店，大力发展养老相关产业融合的新模式新业态。鼓励商业保险机构在风险可控和商业可持续的前提下，开发老年人健康保险产品。市场监管等部门要加强监管，严厉打击侵犯知识产权和制售假冒伪劣商品等违法行为，维护老年人消费权益，营造安全、便利、诚信的消费环境。

**七、强化老龄工作保障**

（十八）加强人才队伍建设。加快建设适应新时代老龄工作需要的专业技术、社会服务、经营管理、科学研究人才和志愿者队伍。用人单位要切实保障养老服务人员工资待遇，建立基于岗位价值、能力素质、业绩贡献的工资分配机制，提升养老服务岗位吸引力。大力发展相关职业教育，开展养老服务、护理人员培养培训行动。对在养老机构举办的医疗机构中工作的医务人员，可参照执行基层医务人员相关激励政策。

（十九）加强老年设施供给。各地区各有关部门要按照《国家积极应对人口老龄化中长期规划》的要求，加强老年设施建设，加快实现养老机构护理型床位、老年大学（学校）等方面目标。各地要制定出台新建城区、新建居住区、老城区和已建成居住区配套养老服务设施设置标准和实施细则，落实养老服务设施设置要求。新建城区、新建居住区按标准要求配套建设养老服务设施实现全覆盖。到2025年，老城区和已建成居住区结合城镇老旧小区改造、居住区建设补短板行动等补建一批养老服务设施，“一刻钟”居家养老服务圈逐步完善。依托和整合现有资源，发展街道（乡镇）区域养老服务中心或为老服务综合体，按规定统筹相关政策和资金，为老年人提供综合服务。探索老年人服务设施与儿童服务设施集中布局、共建共享。

（二十）完善相关支持政策。适应今后一段时期老龄事业发展的资金需求，完善老龄事业发展财政投入政策和多渠道筹资机制，继续加大中央预算内投资支持力度，进一步提高民政部本级和地方各级政府用于社会福利事业的彩票公益金用于养老服务的比例。各地要统筹老龄事业发展，加大财政投入力度，各相关部门要用好有关资金和资源，积极支持老龄工作。研究制定住房等支持政策，完善阶梯电价、水价、气价政策，鼓励成年子女与老年父母就近居住或共同生活，履行赡养义务、承担照料责任。对赡养负担重的零就业家庭成员，按规定优先安排公益性岗位。落实相关财税支持政策，鼓励各类公益性社会组织或慈善组织加大对老龄事业投入。开展全国示范性老年友好型社区创建活动，将老年友好型社会建设情况纳入文明城市评选的重要内容。

（二十一）强化科学研究和国际合作。加大国家科技计划（专项、基金等）、社会科学基金等对老龄领域科技创新、基础理论和政策研究的支持力度。支持研究机构和高校设立老龄问题研究智库。推进跨领域、跨部门、跨层级的涉老数据共享，健全老年人生活状况统计调查和发布制度。积极参与全球及地区老龄问题治理，推动实施积极应对人口老龄化国家战略与落实2030年可持续发展议程相关目标有效对接。

**八、加强组织实施**

（二十二）加强党对老龄工作的领导。各级党委和政府要高度重视并切实做好老龄工作，坚持党政主要负责人亲自抓、负总责，将老龄工作重点任务纳入重要议事日程，纳入经济社会发展规划，纳入民生实事项目，纳入工作督查和绩效考核范围。加大制度创新、政策供给、财政投入力度，健全老龄工作体系，强化基层力量配备。发挥城乡基层党组织和基层自治组织作用，把老龄工作组织好、落实好，做到层层有责任、事事有人抓。建设党性坚强、作风优良、能力过硬的老龄工作干部队伍。综合运用应对人口老龄化能力评价结果，做好老龄工作综合评估。

（二十三）落实工作责任。全国老龄工作委员会要强化老龄工作统筹协调职能，加强办事机构能力建设。卫生健康部门要建立完善老年健康支撑体系，组织推进医养结合，组织开展疾病防治、医疗照护、心理健康与关怀服务等老年健康工作。发展改革部门要拟订并组织实施养老服务体系规划，推进老龄事业和产业发展与国家发展规划、年度计划相衔接，推动养老服务业发展。民政部门要统筹推进、督促指导、监督管理养老服务工作，拟订养老服务体系政策、标准并组织实施，承担老年人福利和特殊困难老年人救助工作。教育、科技、工业和信息化、公安、财政、人力资源社会保障、自然资源、住房城乡建设、商务、文化和旅游、金融、税务、市场监管、体育、医疗保障等部门要根据职责分工，认真履职，主动作为，及时解决工作中遇到的问题，形成齐抓共管、整体推进的工作机制。

（二十四）广泛动员社会参与。注重发挥工会、共青团、妇联、残联等群团组织和老年人相关社会组织、机关企事业单位的作用，结合各自职能开展老龄工作，形成全社会共同参与的工作格局。发挥中国老龄协会推动老龄事业发展的作用，提升基层老年协会能力。及时总结推广老龄工作先进典型经验。

**“十四五”公共服务规划**

第六章 系统提升公共服务效能.

第一节 统筹规划公共服务设施布局

合理控制公共服务设施规模。公共服务设施建设坚持功能优先、经济适用的原则，不宜盲目追求大规模的综合性设施。对于高频次服务设施 ， 应适度减小规模、增加布点，通过总分馆（院）、连锁等多种方式形成服务合力，共享优质资源。对于服务频次相对较低或多个服务事项具有较强相关性的设施，应统筹考虑服务链条，适度集中布局，推广“只跑一次”等已有成功经验，简化办理流程。在鼓励应用现代信息技术提供服务便利性的同时，为老年人、残疾人等特殊人群保留必要的现场服务窗口。

第三节 提高公共服务便利共享水平
推进新技术创新应用。推动数字化服务普惠应用，充分运用大数据、云计算、人工智能、物联网、区块链等新技术手段，鼓励支持新技术赋能，为人民群众提供更加智能、更加便捷、更加优质的公共服务。促进“互联网+公共服务”发展，推动线上线下融合互动，支持高水平公共服务机构对接基层、边远和欠发达地区。促进人工智能在公共服务领域推广应用，鼓励支持数字创意、智慧就业、智慧医疗、智慧住房公积金、智慧法律服务、智慧旅游、智慧文化、智慧广电、智能体育、智慧养老等新业态新模式发展。促进公共服务与互联网产业深度融合发展，大力培育跨行业跨领域综合性平台和行业垂直平台。探索“区块链+”在公共服务领域的运用。加快信息无障碍建设，切实解决老年人等特殊群体在运用智能技术方面遇到的突出困难，帮助老年人、残疾人等共享数字生活。充分发挥全国一体化政务服务平台一网通办枢纽作用，推动更多公共服务事项网上办、掌上办、一次办，持续提升公共服务数字化智能化水平。

**国家卫生健康委、全国老龄办、国家中医药局关于全面加强老年健康服务工作的通知**

　　为贯彻落实全国老龄工作会议精神，协同推进健康中国战略和积极应对人口老龄化国家战略，持续增加老年健康服务供给，切实提高老年健康服务质量，不断满足老年人的健康服务需求，现就全面加强老年健康服务工作通知如下：
　　一、增强老年健康服务意识
　　人口老龄化是我国今后相当长一个时期的基本国情，健康服务需求是老年人最急迫、最突出的需求，促进健康老龄化是积极应对人口老龄化的长久之计。提升医疗卫生服务体系的适老化水平，建立完善老年健康服务体系，推进老年健康预防关口前移，持续扩大优质老年健康服务的覆盖面，向内在能力不同的老年人提供精准健康服务，促进“以疾病为中心”向“以健康为中心”转变，是促进健康老龄化的必然要求。各地要强化健康老龄化理念，切实增强老年健康服务意识，提升老年健康服务水平，解决好老年人的操心事、烦心事，不断提升老年人在健康方面的获得感、幸福感和安全感。

二、做好老年健康服务
　　（一）加强老年人健康教育。在城乡社区加强老年健康知识宣传和教育，利用多种方式和媒体媒介，面向老年人及其照护者广泛传播营养膳食、运动健身、心理健康、伤害预防、疾病预防、合理用药、康复护理、生命教育、消防安全和中医养生保健等科普知识。组织实施老年人健康素养促进项目，有针对性地加强健康教育，提升老年人健康素养。利用老年健康宣传周、敬老月、重阳节、世界阿尔茨海默病日等契机，积极宣传《老年健康核心信息》《预防老年跌倒核心信息》《失能预防核心信息》《阿尔茨海默病预防与干预核心信息》等老年健康科学知识和老年健康服务政策。将老年健康教育融入临床诊疗工作，鼓励各地将其纳入医疗机构绩效考核内容。
　　（二）做实老年人基本公共卫生服务。落实国家基本公共卫生服务老年人健康管理项目，提供生活方式和健康状况评估、体格检查、辅助检查和健康指导服务，到2025年，65岁及以上老年人城乡社区规范健康管理服务率达到65%以上。利用多种渠道动态更新和完善老年人健康档案内容，包括个人基本信息、健康体检信息、重点人群健康管理记录和其他医疗卫生服务记录，推动健康档案的务实应用。各地结合实际开展老年健康与医养结合服务项目，重点为失能老年人提供健康评估和健康服务，为居家老年人提供医养结合服务，有条件的地方要逐步扩大服务覆盖范围。
　　（三）加强老年人功能维护。加强老年人群重点慢性病的早期筛查、干预及分类指导，积极开展阿尔茨海默病、帕金森病等神经退行性疾病的早期筛查和健康指导，提高公众对老年痴呆防治知识的知晓率。鼓励有条件的地方开展老年人认知功能筛查，及早识别轻度认知障碍，预防和减少老年痴呆发生。组织开展老年人失能（失智）预防与干预试点工作，鼓励有条件的省（区、市）组织开展省级试点工作，减少老年人失能（失智）发生。加强老年人伤害预防，减少伤害事件发生。鼓励有条件的地方开展老年人视、听等感觉能力评估筛查，维护老年人内在功能。组织开展老年口腔健康行动，将普及口腔健康知识和防治口腔疾病相结合，降低老年人口腔疾病发生率。组织实施老年营养改善行动，改善老年人营养状况。
　　（四）开展老年人心理健康服务。重视老年人心理健康，针对抑郁、焦虑等常见精神障碍和心理行为问题，开展心理健康状况评估和随访管理，为老年人特别是有特殊困难的老年人提供心理辅导、情绪纾解、悲伤抚慰等心理关怀服务。总结推广老年心理关爱项目经验，各省（区、市）要组织实施省级项目。到2025年，老年心理关爱项目点覆盖全国所有县（市、区）。
　　（五）做好老年人家庭医生签约服务。加强家庭医生签约服务宣传推广，为老年人提供基本医疗卫生、健康管理、健康教育与咨询、预约和转诊、用药指导、中医“治未病”等服务。提高失能、高龄、残疾等特殊困难老年人家庭医生签约覆盖率，到2025年不低于80%。进一步强化服务履约，采取更加灵活的签约周期，方便老年人接受签约服务。家庭医生要定期主动联系签约老年人了解健康状况，提供针对性的健康指导，切实提高签约老年人的获得感和满意度。
　　（六）提高老年医疗多病共治能力。加强国家老年医学中心和国家老年区域医疗中心设置与管理，鼓励建设省级老年区域医疗中心。加强综合性医院老年医学科建设，到2025年，二级及以上综合性医院设立老年医学科的比例达到60%以上。医疗机构要积极开展老年综合评估、老年综合征诊治和多学科诊疗，对住院老年患者积极开展跌倒、肺栓塞、误吸和坠床等高风险筛查，提高多病共治能力。鼓励各地争取资源加强基层医疗卫生机构老年健康服务科室建设，充分发挥大型医院的帮扶带动作用，借助医疗联合体等形式，帮助和指导基层医疗卫生机构开展老年健康服务，惠及更多老年人。
　　（七）加强老年人居家医疗服务。贯彻落实《[关于加强老年人居家医疗服务工作的通知](javascript:SLC(349578))》要求，增加居家医疗卫生服务供给，重点对居家行动不便的高龄或失能老年人，慢性病、疾病康复期或终末期、出院后仍需医疗服务的老年患者提供诊疗服务、医疗护理、康复治疗、药学服务、安宁疗护。扩大医疗机构提供家庭病床、上门巡诊等居家医疗服务的范围，鼓励医联体提供居家医疗服务，按规定报销相关医疗费用，按成本收取上门服务费。
　　（八）加强老年人用药保障。完善社区用药相关制度，保证老年慢性病、常见病药品配备，方便老年人就近取药，提高老年人常见病用药可及性。鼓励医疗机构开设药学门诊，发展居家社区药学服务和“互联网＋药学服务”，为长期用药老年人提供用药信息和药学咨询服务，开展个性化的合理用药宣教指导。落实慢性病长期处方制度的有关要求，为患有多种疾病的老年患者提供“一站式”长期处方服务，减少老年患者往返医院次数，解决多科室就医取药问题。鼓励医疗机构开展老年人用药监测，并将结果运用到老年人日常健康管理之中，提高老年人安全用药、合理用药水平。
　　（九）加强老年友善医疗服务。贯彻落实《[关于开展建设老年友善医疗机构工作的通知](javascript:SLC(5012104))》《[关于实施进一步便利老年人就医举措的通知](javascript:SLC(5015705))》要求，从文化、管理、服务、环境等方面，加快老年友善医疗机构建设，方便老年人看病就医；不断优化医疗服务流程，改善老年人就医体验。全面落实老年人医疗服务优待政策，完善诊间、电话、自助机、网络、现场预约等多种预约挂号方式，保留一定比例的现场号源。医疗机构内的各种标识要醒目、简明、易懂、大小适当，要对公共设施进行适老化改造，配备必要且符合国家无障碍设计标准的无障碍设施。鼓励医疗机构设立志愿者服务岗，明确导诊、陪诊服务人员，提供轮椅、平车等设施设备。到2025年，85%以上的综合性医院、康复医院、护理院和基层医疗卫生机构成为老年友善医疗机构。
　　（十）大力发展老年护理、康复服务。贯彻落实《[关于加强老年护理服务工作的通知](javascript:SLC(338019))》《关于加快推进康复医疗工作发展的意见》要求，鼓励医疗资源丰富地区的部分一级、二级医院转型为护理院、康复医院等，加强接续性医疗机构建设，畅通双向转诊通道。通过新建、改（扩）建、转型发展，鼓励多方筹资建设基于社区、连锁化的康复中心和护理中心。鼓励有条件的基层医疗卫生机构根据需要设置和增加提供老年护理、康复服务的床位。鼓励有条件的地区和医疗机构开展“互联网＋护理服务”。鼓励二级及以上综合性医院提供康复医疗服务。通过为老年患者提供早期、系统、专业、连续的康复医疗服务，促进老年患者功能恢复。
　　（十一）加强失能老年人健康照护服务。完善从专业机构到社区、居家的失能老年人健康照护服务模式。鼓励建设以失能老年人为主要服务对象的护理院（中心）。鼓励二级及以下医院、基层医疗卫生机构与护理站建立签约合作关系，共同为居家失能老年人提供健康照护服务。面向居家失能老年人照护者开展照护技能培训，提高家庭照护者的照护能力和水平。借助信息化手段，对失能低收入老年人的医疗保障、健康照护等情况以及因病返贫风险进行动态监测，维护失能低收入老年人身心健康。
　　（十二）加快发展安宁疗护服务。推动医疗机构根据自身功能和定位，开设安宁疗护病区或床位，开展安宁疗护服务。推动有条件的地方积极开展社区和居家安宁疗护服务，探索建立机构、社区和居家安宁疗护相结合的工作机制。建立完善安宁疗护多学科服务模式，为疾病终末期患者提供疼痛及其他症状控制、舒适照护等服务，对患者及其家属提供心理支持和人文关怀。加强对公众的宣传教育，推动安宁疗护理念得到社会广泛认可和接受。
　　（十三）加强老年中医药健康服务。二级及以上中医医院要设置“治未病”科室，鼓励开设老年医学科，增加老年病床数量，开展老年常见病、慢性病防治和康复护理。提高康复、护理、安宁疗护等医疗机构的中医药服务能力，推广使用中医药综合治疗。到2025年，三级中医医院设置康复科比例达到85%。积极发挥城乡社区基层医疗卫生机构为老年人提供优质规范中医药服务的作用，推进社区和居家中医药健康服务，促进优质中医药资源向社区、家庭延伸，到2025年，65岁及以上老年人中医药健康管理率达到75%以上。鼓励中医医师加入老年医学科工作团队和家庭医生签约团队。积极开展中医药膳食疗科普等活动，推广中医传统运动项目，加强中医药健康养生养老文化宣传。
　　（十四）做好老年人传染病防控。医疗卫生机构要按照传染病防控部署，及时为老年人接种相关疫苗。有条件的地方做好流感、肺炎等疫苗接种，减少老年人罹患相关疾病风险。在疫苗接种工作中，对独居、高龄、行动不便或失能等特殊老年人，要给予重点关注，提供周到服务。加强老年人结核病防治工作，做好老年结核病患者的定点救治。积极开展老年人艾滋病预防知识宣传教育，有条件的地区提供艾滋病检测服务。建立老年人突发公共卫生事件应急处置机制和预案，在突发传染病等重大公共卫生事件中，充分考虑老年人特点，保障老年人应急物资和医疗卫生服务供给。

三、强化老年健康服务的组织保障
　　（一）加强组织领导。各级卫生健康行政部门（老龄办）、中医药主管部门要切实增强为老服务意识，将老年健康服务工作摆上重要议事日程，每年至少召开一次专题会议重点研究部署。要落实各内设机构和直属联系单位相关职责，形成工作合力，加大资金、政策、人员倾斜，共同做好老年健康服务工作。要加强行风建设，将提供老年健康服务的医疗机构纳入卫生健康“双随机一公开”行业监督内容。充分发挥涉老社会组织作用，为老年人提供健康促进、健康照护和精神慰藉等服务。
　　（二）加强政策保障。推动将老年健康服务体系建设和老年健康服务作为重要内容纳入各地卫生健康服务体系建设规划和卫生健康事业发展规划，促进城乡、区域老年健康服务均衡发展。结合疾控体系改革和医药卫生体制改革，加强老年健康服务供给侧改革，加强老年疾病预防控制能力建设，优化老年医疗服务资源。深入开展健康中国行动老年健康促进行动，推动将老年健康服务相关项目纳入各级政府民生实事项目。
　　（三）加强科技支撑。推进国家老年疾病临床医学研究中心等老年医学研究机构建设。鼓励各级卫生健康行政部门、中医药主管部门设立老年健康科研专项，加强老年健康科学研究，支持老年健康相关预防、诊断、治疗技术和产品研发，加强老年健康科研成果转化和适宜技术推广。逐步完善全国老龄健康信息管理系统，促进各类健康数据的汇集和融合，整合信息资源，实现信息共享，以信息化推动老年健康服务管理质量提升。
　　（四）加强队伍建设。加强老年医学学科建设和发展。加强内科、全科专业住院医师的老年医学知识与技能培训。组织实施老年医学紧缺人才培训项目。支持退休、转岗的护士从事失能老年人护理指导、培训和服务等工作。开展医疗护理员职业技能培训和就业指导服务，充实长期照护服务队伍。

**最高人民法院关于为实施积极应对人口老龄化国家战略提供司法服务和保障的意见**

　一、统一思想认识，准确把握为实施积极应对人口老龄化国家战略提供司法服务和保障的总体要求

　　1．指导思想。各级人民法院要切实提高政治站位，坚持以习近平新时代中国特色社会主义思想为指导，深入贯彻习近平法治思想，深入学习领会“两个确立”的决定性意义，增强“四个意识”、坚定“四个自信”、做到“两个维护”。坚持以人民为中心，把积极老龄观、健康老龄化理念融入审判执行工作全过程，大力弘扬中华民族孝亲敬老传统美德，为推动构建老年友好型社会、加强老年人权益保障提供有力司法服务和保障。

　　2．重大意义。人口老龄化是我国未来相当长一个时期的基本国情。随着老龄化程度加深、劳动力供给数量减少，家庭养老负担和基本公共服务供给压力将进一步增加。有效应对我国人口老龄化，事关国家发展全局，事关亿万百姓福祉，事关社会和谐稳定，对于全面建设社会主义现代化国家具有重要意义。各级人民法院要充分认识实施积极应对人口老龄化国家战略的重要性和紧迫性，采取有力措施切实保障老年人合法权益，让老年人共享改革发展成果、安享幸福晚年。

　　3．目标任务。新发展阶段，各级人民法院要建立健全上下贯通、一抓到底的工作体系，将服务和保障实施积极应对人口老龄化国家战略纳入审判执行的总体工作之中。推动完善老年人优待政策、法规体系，涉及老年人利益司法政策的制定和执行过程要充分征求老年人意见，推动人民法院服务和保障实施积极应对人口老龄化国家战略的各项政策举措落地、落实、落细。

　　二、充分发挥审判职能作用，加强老年人权益保障

　　4．依法妥善审理涉老年人婚姻家庭纠纷案件。依法审理赡养纠纷案件，保障老年人基本生活需要。加强老年人精神赡养类案件调解力度，增进对老年人的精神关爱。注重法院的职权调查，强化依法裁量，依法保护老年人的婚姻自由。对于老年人同居析产纠纷，要综合考量共同生活时间、各自付出等因素，兼顾双方利益，实现公平公正。

　　5．依法妥善审理涉老年人继承纠纷案件。落实[民法典](javascript:SLC(342411))遗产管理人制度，依法确定遗产管理人，保障遗产妥善管理、顺利分割。要依法保护各类遗嘱形式，切实尊重老年人立遗嘱时的真实意愿，保障老年人遗产处分权。依法认定各类遗赠扶养协议效力，满足养老形式多样化需求。

　　6．贯彻实施[反家庭暴力法](javascript:SLC(261780))，保护老年家庭成员人身、财产安全。推动完善各部门共同参与的反家暴宏观体系。加强对家庭暴力受害老年人举证的指导，加大心理疏导和帮扶力度。建立人身安全保护令案件受理“绿色通道”，加大依职权调取证据力度，依法及时作出、送达人身安全保护令。加强与公安机关、居民委员会、村民委员会等部门协作配合，充分利用协助执行制度，保障人身安全保护令切实发挥作用。建立定期回访、跟踪机制，拓展反家暴延伸服务范围。

　　7．完善老年人监护制度。妥善审理监护权纠纷案件，最大程度尊重老年当事人的真实意愿。依法认定老年人通过意定监护协议确定的监护人，督促其依法履行监护责任。对于侵犯无民事行为能力、限制民事行为能力老年人合法权益的监护人，依法撤销其监护人资格，为老年人安排必要的临时监护措施，按照最有利于被监护人的原则依法指定监护人，保护老年人人身权利、财产权利及其他合法权益。

　　8．依法妥善审理涉养老纠纷案件，促进老有所养。贯彻落实[民法典](javascript:SLC(342411))关于居住权的规定，依法审理涉老年人居住权益保护案件，满足老年人稳定的生活居住需要，为“以房养老”模式提供坚实的法律保障。依法妥善审理养老服务合同纠纷案件，确保养老机构提供符合质量和安全标准的养老服务，推动机构养老规范化发展。

　　9．推动农村养老保障服务发展。依法审理涉及农村土地承包经营权、侵害集体经济组织成员权益等纠纷案件，保障老年人依法享有本集体经济组织成员权益，增加农村老年人收入。发挥审判职能作用，保障无劳动能力、无生活来源又无人赡养、扶养的老年村民享受农村五保供养待遇。

　　10．依法妥善审理涉老年人医疗服务合同纠纷案件，促进老有所医。依法认定家庭病床、巡诊等居家医疗服务合同中各方当事人的权利义务关系，保障老年人合法权益。妥善审理老年人医疗、失能老年人长期照护等服务合同纠纷案件，发挥审判职能，保障医养结合政策的贯彻实施，为老年人健康生活保驾护航。

　　11．加强老年人劳动权益保护，促进老有所为。依法审理涉老年人劳动争议案件，助力老年人就业、维护老年人再就业权益。助推“银龄行动”，引导具有一定经验和专业技术的老年人以志愿服务形式积极参与民事调解等活动。各地人民法院可以根据实际探索建立退休法官专家库，鼓励有意愿的退休法官积极参与诉前调解、调查研究等。

　　12．依法妥善审理老年人参与社会文化生活相关案件，促进老有所学、老有所乐。妥善审理涉老年人旅游合同纠纷等案件，督促、引导服务机构充分、合理履行提示说明义务和安全保障义务，不断提升老年人生活质量，满足老年人日益增长的美好生活需要。

　　13．依法加大对侵害老年人人身和财产权益违法犯罪行为的打击力度。依法严惩虐待、遗弃、伤害老年人等违法犯罪行为。严厉打击针对老年人的电信网络诈骗、借用“以房养老”之名实施的“套路贷”，依法惩处家庭成员盗窃、诈骗、抢夺、侵占、勒索、故意损毁老年人财物等违法犯罪行为。依法严惩消费领域违法犯罪行为，维护老年人消费权益，为老年人营造安全、便利、诚信的消费环境。

　　14．加大涉老年人权益案件执行力度。各地人民法院要加大涉老年人居住权案件执行力度，依法及时维护老年人居住权益，保障老年人住有所居。加大对老年人追索赡养费、扶养费案件的先予执行力度。创新涉老年人精神赡养纠纷案件执行方式，督促、引导赡养人积极主动履行赡养义务。

　　三、持续深化改革创新，建立健全便老惠老司法服务机制

　　15．深化一站式多元解纷机制建设，推动涉老年人矛盾纠纷源头化解。坚持和发展新时代“枫桥经验”，坚持把非诉讼纠纷解决机制挺在前面。建立完善涉老年人婚姻家庭、侵权等矛盾纠纷的预警、排查、调解机制。构建多元解纷和诉讼服务体系，促进涉老年人矛盾纠纷一站式多元化解。推动人民法院一站式多元解纷向基层延伸，推进人民法庭进乡村、进社区、进网格，加强巡回审判，及时就地化解矛盾纠纷。坚持服务老年人需求导向，建设一站式诉讼服务中心，提供“一站通办、一网通办、一号通办、一次通办”的诉讼服务。

　　16．深入推进社会主义核心价值观融入裁判文书释法说理。在涉老年人等弱势群体保护、诉讼各方存在较大争议且可能引发社会广泛关注的案件中，要强化运用社会主义核心价值观释法说理。加强社会主义核心价值观在涉老年人权益案件中的导向作用，切实发挥司法裁判规范、评价、教育、引领等功能，实现政治效果、法律效果和社会效果的有机统一。

　　17．进一步深化家事审判方式和工作机制改革。树立人性化审判理念，注重将对老年当事人的保护从身份利益、财产利益全面延伸到人格利益、安全利益和情感利益。充分发挥家事审判对婚姻家庭关系的诊断、修复和治疗作用，为老年人安享幸福晚年提供和睦稳定的家庭环境。

　　18．加强法律宣传。各级人民法院要通过法律进社区、巡回审判、推广学习典型案例等多种方式，加强老年人权益保障普法宣传。提高老年人运用法律手段保护自身权益的意识，提升老年人识骗防骗能力。推动在全社会树立保障老年人合法权益的法律意识，形成关心关爱老年人的良好氛围。

　　19．加大法律援助协作和司法救助力度。加强与法律援助机构的协调配合，依法及时转交老年当事人的法律援助申请。对于符合司法救助条件的老年当事人，人民法院应当依法予以救助。会同相关部门加大对受害老年人临时庇护、法律援助的帮扶力度，加大司法救助力度，推动建立多层次救助体系。

　　20．建立适老型诉讼服务机制，为便利老年人参与诉讼活动提供保障。聚焦涉老年人案件类型和特点，探索建立涉老年人民事案件专业化审判机制。依法准许书写起诉状确有困难的老年人口头起诉，有效给予老年人诉讼服务指导和帮助。为行动不便的老年人开通上门立案、电话立案等绿色通道，实现快速、便捷立案。开展网上立案、电子诉讼的同时，保留老年人易于接受的传统司法服务方式。完善无障碍诉讼设施及服务，方便老年人参加诉讼。根据案件情况，允许相关辅助、陪护人员陪同老年当事人出庭。依法妥善处理老年人涉诉信访案件，对于老年当事人应当予以特别关照。

## “十四五”健康老龄化规划

以满足老年人对健康的基本需求、兼顾多层次和多样化需求为目的，以体制机制的改革创新为根本动力，大力推进老龄健康服务供给侧结构性改革，把积极老龄观、健康老龄化理念融入经济社会发展全过程，深入开展老年健康促进行动，持续发展和维护老年人健康生活所需要的内在能力，促进实现健康老龄化。

**（二）基本原则**

**1.健康优先，全程服务。**坚持健康至上，以老年人健康为中心，提供包括健康教育、预防保健、疾病诊治、康复护理、长期照护、安宁疗护等在内的老年健康服务。

**2.需求导向，优质发展。**以老年人健康需求为导向，优化供给侧改革，推动老年健康服务高质量发展，增量与提质并重。构建优质高效的整合型医疗卫生服务体系，加大医养结合服务供给，促进医疗卫生与养老服务深度结合。

**3.政府主导，全民行动。**发挥政府在促进健康老龄化工作中的主导作用，鼓励社会资本参与，构建多层次、多样化的老年健康服务体系。倡导个人和家庭积极参与，共同构建老年友好型社会。

**4.公平可及，共建共享。**以保障全体老年人健康权益为出发点，不断深化体制机制改革，积极推动城乡、区域老年健康服务均衡发展，确保老年健康服务公平可及，由全体老年人共享。

**（三）发展目标**

到2025年，老年健康服务资源配置更加合理，综合连续、覆盖城乡的老年健康服务体系基本建立，老年健康保障制度更加健全，老年人健康生活的社会环境更加友善，老年人健康需求得到更好满足，老年人健康水平不断提升，健康预期寿命不断延长。

——老年健康服务机构数量增加，服务能力大幅提升，相关学科专业建设不断加强，服务队伍更加壮大，服务内容更加丰富，老年人享有健康服务的可及性进一步提高。

——居家社区机构健康服务协调推进，医养结合服务供给不断增加，供需均衡程度不断提高，服务质量不断提升，老年人健康生活质量持续改善。

——医疗卫生机构适老化水平不断提高，老年人看病就医服务流程不断优化，老年人就医体验不断改善，有利于老年人“就近就便”就医的环境基本建立。

——老年健康保障机制不断增强，科技和信息化支撑能力明显提升，相关制度、标准、规范基本建立，老年健康产业有序发展，老年健康产品市场提质扩容。

**表 主要指标**

| **序号** | **主要指标** | **单位** | **2020年** | **2025年** | **性质** |
| --- | --- | --- | --- | --- | --- |
| **1** | **老年人健康素养水平** | **%** | **—** | **有所提高** | **预期性** |
| **2** | **65～74岁老年人失能发生率** | **%** | **—** | **有所下降** | **预期性** |
| **3** | **65岁及以上老年人城乡社区规范化健康管理服务率** | **%** | **—** | **≥65** | **预期性** |
| **4** | **65岁及以上老年人中医药健康管理率** | **%** | **68.4** | **≥75** | **预期性** |
| **5** | **二级及以上综合性医院设立老年医学科的比例** | **%** | **31.8** | **≥60** | **预期性** |
| **6** | **综合性医院、康复医院、护理院和基层医疗卫生机构中老年友善医疗卫生机构占比** | **%** | **—** | **≥85** | **约束性** |
| **7** | **三级中医医院设置康复（医学）科的比例** | **%** | **78.0** | **≥85** | **约束性** |

三、主要任务

**（一）强化健康教育，提高老年人主动健康能力**

**1.拓展老年健康教育内容。**在全社会开展人口老龄化国情教育，树立积极老龄观。引导老年人将“维护机体功能，保持自主生活能力”作为健康目标，树立“自己是健康第一责任人”的意识，强化“家庭是健康第一道关口”的观念，促进老年人及其家庭践行健康生活方式。普及营养膳食、运动健身、心理健康、疾病预防、合理用药、康复护理、生命教育、应急救助等老年健康知识，宣传维护感官功能、运动功能和认知功能的预防措施，不断提高老年人健康核心信息知晓率和健康素养水平。广泛开展关爱失智老年人的社会宣传与公共教育活动，提升公众的失智预防和失智照护水平。普及智能技术知识和技能，提升老年人对健康信息的获取、识别和使用能力。加强对老年健康政策、服务和产品的科普宣传。

**2.形成多元化的老年健康教育服务供给格局。**支持各类教育机构将老年健康教育纳入课程内容。鼓励开办医学专业的院校、医疗卫生机构等设置老年健康教育专属阵地，面向老年人及家属、照护者开设养生保健、照护技能培训等课程。依托全国开放大学、老年教育机构、社区教育机构、老年协会、城乡社区党群服务中心、基层医疗卫生机构、文化体育场馆等，提高城乡老年健康教育服务覆盖率。

**3.创新老年健康教育服务提供方式。**组织开展全国老年健康宣传周、世界阿尔茨海默病日等主题宣传活动。开发科普视频，建设开放共享的数字化国家级老年健康教育科普资源库。充分利用传统媒体、短视频、微信公众号、微博、移动客户端等多种方式和媒体媒介，传播老年健康相关知识，宣传老年健康达人典型案例。鼓励各地探索可行模式，充分发挥老年人在老年健康教育中的示范引领作用，增强健康教育效果。（卫生健康委、教育部、广电总局、体育总局、中医药局按职责分工负责）

| **专栏1 老年健康教育专项工程** |
| --- |
| **实施老年健康素养促进项目。**监测老年人健康素养和中医药健康文化素养状况，开展有针对性的健康教育活动，不断提高老年人健康核心信息知晓率和老年人健康素养水平。  **开展老年健康宣传周活动。**针对老年人主要健康问题，每年确定一个主题，在全国城乡组织开展老年健康宣传周活动，营造有利于老年人健康生活的社会环境。 |

**（二）完善身心健康并重的预防保健服务体系**

**4.提高基本公共卫生服务促进老年人健康的能力。**建立综合、连续、动态的老年人健康管理档案，鼓励各地整合老年人健康体检信息，优化老年人健康体检项目，提升健康评估和健康指导能力。推动地方积极开展老年健康与医养结合服务。将失能、高龄、残疾、计划生育特殊家庭等老年人作为家庭医生签约服务重点人群，拓展签约服务内涵，提高服务质量。到2025年，65岁及以上老年人城乡社区规范化健康管理服务率达到65%以上，65岁及以上老年人中医药健康管理率达到75%以上。

**6.开展老年人心理关爱服务。**完善精神障碍类疾病早期预防及干预机制，扩大老年人心理关爱行动覆盖范围，针对抑郁、焦虑等老年人常见精神障碍和心理行为问题，开展心理健康状况评估、早期识别和随访管理，为老年人特别是有特殊困难的老年人提供心理辅导、情绪纾解、悲伤抚慰等心理关怀服务。鼓励设置心理学相关学科专业的院校、心理咨询机构等开通老年人心理援助热线，为老年人提供心理健康服务。加强全国社会心理服务体系建设试点地区的基层社会心理服务平台建设，提升老年人心理健康服务能力，完善老年人心理健康服务网络。

| **专栏2 老年预防保健专项工程** |
| --- |
| **开展老年人失能预防与干预工作。**针对导致老年人失能的高风险因素，如衰弱、肌少症、营养不良、心脑血管疾病等，实施积极预防和干预。  **实施老年心理关爱行动。**总结推广老年人心理关爱工作经验，持续扩大覆盖范围，原则上每个县（市、区）都设有老年人心理关爱点。  **实施老年营养改善行动。**开展老年人营养风险筛查试点工作，对低体重高龄老年人进行营养干预。  **实施老年口腔健康行动。**开展老年口腔健康科普宣传，针对基层和偏远地区专业人员进行专业培训，开展老年口腔健康公益活动。 |

**7.推进体卫融合。**加强城乡社区、医养结合机构健身设施建设，提高适老化程度。研究推广适合老年人的体育健身休闲项目、方式和方法，发布老年人体育健身活动指南。将运动干预纳入老年人慢性病防控与康复方案。充分发挥各级老年人体育协会的作用，指导老年人科学健身，组织开展适合老年人的赛事活动。

**（三）以连续性服务为重点，提升老年医疗服务水平**

**（四）健全居家、社区、机构相协调的失能老年人照护服务体系**

**12.支持居家（社区）照护服务。**支持社区、机构为失能老年人家庭提供家庭照护者培训和“喘息”服务，组织协调志愿者对居家失能老年人开展照护服务。鼓励社会力量利用社区配套用房或闲置用房开办护理站，为失能老年人提供居家健康服务。鼓励社区卫生服务中心与相关机构合作，增加照护功能，为居家老年人提供短期照护、临时照护等服务。

**13.促进机构照护服务发展**。在有条件的社区卫生服务中心、乡镇卫生院等基层医疗卫生机构增设护理床位或护理单元。支持医养结合机构开展失能老年人照护服务工作。支持具备服务能力和相应资质的机构将照护服务向社区和家庭延伸，辐射居家失能老年人。推进照护机构老年痴呆患者照护专区和社区老年痴呆患者照护点建设，满足老年痴呆患者照护服务需求。

**17.加强中医药健康养老服务能力建设。**加快二级及以上中医医院老年医学科建设，加强中医药健康养老服务能力、人才培养能力、技术推广能力建设，提升老年人常见病多发病的中医药服务能力和水平。加强各省级中医治未病中心中医药老年健康服务能力建设，制订相关标准规范，培训推广中医适宜技术，提升中医药特色服务能力。

**18.加大中医药健康养生养老文化宣传。**积极宣传适宜老年人的中医养生保健知识、技术和方法，推动优质中医药服务进社区、进农村、进家庭。积极开展中医健康体检、健康评估、健康干预以及药膳食疗科普等活动，推广太极拳、八段锦、五禽戏等中医传统运动项目，培养树立健康科学的生活方式和理念。

| **专栏4 中医药老年健康服务专项工程** |
| --- |
| **中医医院老年医学科建设。**推动二级及以上中医医院开设老年医学科，完善老年医学科科室基础设施设备，提供老年健康服务。  **中医药特色医养结合机构建设。**在全国医养结合示范机构创建活动中，推动建设一批具有中医药特色的医养结合示范机构，为老年人提供中医体质辨识、诊断治疗、康复护理、养生保健、健康管理等中医药特色服务。 |

**24.强化老年健康照护队伍建设。**增加从事老年护理工作的医疗护理员数量，加大培训力度，开展职业技能培训和就业指导服务，培训一批老年方向的医疗护理员，充实老年健康特别是长期照护服务队伍。健全老年健康相关职业人才评价制度，完善以技术技能价值激励为导向的薪酬分配体系。加快培养服务于老年健康的社会工作者、志愿者队伍，通过入户、社区活动等形式为老年人提供便利可及、针对性强的健康服务。

| **专栏6 老年健康队伍建设专项工程** |
| --- |
| **实施全国老年医学人才培训项目。**对全国二级及以上综合性医院老年医学科和医养结合机构的1万名骨干医护人员，开展线下线上相结合的诊疗知识和技能培训。  **实施安宁疗护服务能力提升培训项目。**对国家安宁疗护试点市（区）从事安宁疗护工作的5000名骨干医护人员开展在线培训，对2000名骨干医护人员开展线下培训。  **实施全国医养结合人才能力提升培训项目。**对全国医养结合机构的医护人员开展在线培训，拟培训20万人次。  **实施老年医疗护理员培训项目。**对一批相关人员开展以失能（智）照护知识和技能为主的培训。 |

**25.健全老年健康标准规范体系。**发挥国家卫生健康标准委员会老年健康标准专业委员会作用，健全老年健康基础标准、老年医疗服务标准、老年公共卫生标准、老年社会支持标准、医养结合服务管理标准等。制订老年常见疾病诊疗指南和临床操作技术规范。

**（九）促进健康老龄化的科技和产业发展**

**26.加强老年健康科学研究。**加强衰老机制的基础性研究，加强老年慢性病和共病诊疗技术、老年康复护理技术、老年功能维护技术等应用性研究，提升老年重大疾病防治水平。加强适宜技术研发推广，定期发布老年健康适宜技术产品目录，发展老年神经、睡眠等监测与干预相关技术及产品，发展适宜居家、社区应用的老年健康促进评估、诊断、监测技术与产品。支持老年健康技术研发基地和科研应用转化平台建设。

**27.推动老龄健康产业可持续发展。**推动老年健康与养老、养生、文化、旅游、体育、教育等多业态深度融合发展，大力推动老年健康领域新产业、新业态、新商业模式发展。支持新兴材料、人工智能、虚拟现实技术等在老年健康领域的深度集成应用与推广。支持医疗卫生机构、企业、科研院所加强医工协同发展，研发老年人医疗辅助、家庭照护、安防监控、残障辅助、情感陪护、康复辅具等智能产品和可穿戴设备，提升产品的适老化水平，推进老年产品市场提质扩容。发展健康管理与服务、健康检测与监测等智慧健康养老服务。建立健全相关标准，规范老年用品和为老服务市场。加大监管力度，切实维护老年人权益。

**28.强化信息化支撑。**建立老年健康数据的收集和发布机制。充分运用互联网、物联网、大数据等信息技术手段，创新服务模式，提升老年健康智能化服务质量和效率。依托国家全民健康信息平台，完善全国老龄健康信息管理系统，整合各类老年健康相关数据，实现信息共享，为服务老年人提供信息化支撑。

四、保障措施

**（一）加强组织领导**

各地要认真贯彻落实全面推进健康中国建设、积极应对人口老龄化国家战略部署，广泛宣传促进健康老龄化的重要意义，把“十四五”健康老龄化规划纳入经济社会发展总体规划，把推动老龄健康事业和产业发展作为深化供给侧结构性改革、改进民生福祉的重要抓手，健全党委领导、政府主导、部门协同、社会参与的工作机制，积极出台相关扶持政策，全面完成“十四五”健康老龄化规划的各项目标任务。各地要结合实际制定本地区的“十四五”健康老龄化规划实施办法。

**（二）加大投入力度**

按照事权和支出责任相适应的原则，把促进健康老龄化必要经费列入本级预算。拓宽经费筹资渠道，充分发挥彩票公益金、慈善捐助等多元资金的作用，提供普惠性老年健康和医养结合服务，促进城乡老年健康服务均等化。

**（三）完善保障体系**

完善高血压、糖尿病门诊用药保障机制。将患慢性病需长期服药或患重特大疾病需长期门诊治疗导致自负费用较高且基本生活出现困难的老年人按规定纳入医疗救助范围。稳妥推进长期护理保险制度试点，建立适合我国国情的长期护理保险制度框架。鼓励商业保险公司开发老年人疾病保险、长期护理保险、意外伤害保险等专属保险产品。推进社保卡（含电子社保卡）在老年人就医服务领域应用。

**（四）强化督导考核**

充分发挥全国老龄办的综合协调作用，把促进健康老龄化的政策措施作为评价全国老龄委成员单位履职尽责情况的重要内容。完善信息统计和需求反馈机制，加强对规划实施的动态跟踪监测。建立健全监测检查评估评价机制，督查重大项目、重大工程实施情况，组织开展规划实施进度和实施效果的全面检查评估。

**中共中央办公厅、国务院办公厅印发《关于推进基本养老服务体系建设的意见》**

中共中央办公厅、国务院办公厅印发《关于推进基本养老服务体系建设的意见》

　　中共中央办公厅、国务院办公厅印发了《关于推进基本养老服务体系建设的意见》，并发出通知，要求各地区各部门结合实际认真贯彻落实。
　　《关于推进基本养老服务体系建设的意见》主要内容如下。
　　基本养老服务在实现老有所养中发挥重要基础性作用，推进基本养老服务体系建设是实施积极应对人口老龄化国家战略，实现基本公共服务均等化的重要任务。党的十八大以来，在党中央坚强领导下，基本养老服务加快发展，内容逐步拓展，公平性、可及性持续增强。为贯彻落实党中央、国务院有关决策部署，健全基本养老服务体系，更好保障老年人生活，现提出如下意见。
**一、**总体要求
　　（一）指导思想。以习近平新时代中国特色社会主义思想为指导，立足新发展阶段，完整、准确、全面贯彻新发展理念，构建新发展格局，坚持党对基本养老服务体系建设的全面领导，坚持以人民为中心，坚持以改革创新为根本动力，加快建成覆盖全体老年人、权责清晰、保障适度、可持续的基本养老服务体系，不断增强老年人的获得感、幸福感、安全感。
　　（二）主要任务。基本养老服务是指由国家直接提供或者通过一定方式支持相关主体向老年人提供的，旨在实现老有所养、老有所依必需的基础性、普惠性、兜底性服务，包括物质帮助、照护服务、关爱服务等内容。基本养老服务的对象、内容、标准等根据经济社会发展动态调整，“十四五”时期重点聚焦老年人面临家庭和个人难以应对的失能、残疾、无人照顾等困难时的基本养老服务需求。
　　（三）工作原则
　　--基础性原则。立足我国基本国情，统筹考虑必要性和可能性，着眼保基本、广覆盖、可持续，尽力而为、量力而行，保障老年人的基本生活和照料需要。
　　--普惠性原则。在提高基本公共服务均等化水平的过程中，逐步拓展基本养老服务的对象和内容，使所有符合条件的老年人能够方便可及、大致均等地获得基本养老服务。
　　--共担性原则。在赡养人、扶养人切实履行赡养、扶养义务基础上，通过提供基本养老服务、发挥市场作用、引导社会互助共济等方式，帮助困难家庭分担供养、照料方面的负担。
　　--系统性原则。推动社会保险、社会救助、社会福利、慈善事业、老年优待等制度资源优化整合，强化各相关领域体制改革配套衔接，支持基本养老服务体系发展。

**二、**重点工作
　　（一）制定落实基本养老服务清单。各地区各有关部门要严格落实《国家基本养老服务清单》（见附件）。《国家基本养老服务清单》明确的对象、项目、内容等，根据经济社会发展水平、财力状况等因素动态调整，由民政部会同相关部门适时提出修订意见，按程序报批后以部门名义印发实施。省级政府应当对照《国家基本养老服务清单》制定并发布本地区基本养老服务具体实施方案及清单，明确具体服务对象、内容、标准等，其清单应当包含《国家基本养老服务清单》中的服务项目，且覆盖范围和实现程度不得低于《国家基本养老服务清单》要求。到2025年，基本养老服务制度体系基本健全，基本养老服务清单不断完善，服务对象、服务内容、服务标准等清晰明确，服务供给、服务保障、服务监管等机制不断健全，基本养老服务体系覆盖全体老年人。
　　（二）建立精准服务主动响应机制。建立老年人状况统计调查和发布制度，开展老年人能力综合评估，制定完善全国统一的评估标准，推动评估结果全国范围互认、各部门按需使用。依托全国一体化政务服务平台，推进跨部门数据共享，建立困难老年人精准识别和动态管理机制，细化与常住人口、服务半径挂钩的制度安排，逐步实现从“人找服务”到“服务找人”。推动在残疾老年人身份识别、待遇享受、服务递送、无障碍环境建设等方面实现资源整合，加强残疾老年人养老服务保障。面向独居、空巢、留守、失能、重残、计划生育特殊家庭等老年人提供探访关爱服务。支持基层老年协会、志愿服务组织等参与探访关爱服务。依托基层管理服务平台，提供养老服务政策咨询、信息查询、业务办理等便民养老服务。
　　（三）完善基本养老服务保障机制。推动建立相关保险、福利、救助相衔接的长期照护保障制度。合理确定经济困难失能老年人护理补贴覆盖范围和补贴标准。地方各级政府应当建立基本养老服务经费保障机制，中央财政统筹现有资金渠道给予支持。落实发展养老服务优惠扶持政策，鼓励社会力量参与提供基本养老服务，支持物业服务企业因地制宜提供居家社区养老服务。将政府购买服务与直接提供服务相结合，优先保障经济困难的失能、高龄、无人照顾等老年人的服务需求。具备条件的地方优化养老服务机构床位建设补助、运营补助等政策，支持养老服务机构提供基本养老服务。鼓励和引导企业、社会组织、个人等社会力量依法通过捐赠、设立慈善基金、志愿服务等方式，为基本养老服务提供支持和帮助。开展基本养老服务统计监测工作，建立基本养老服务项目统计调查制度。
　　（四）提高基本养老服务供给能力。设区的市级以上地方政府应当将养老服务设施（含光荣院）建设纳入相关规划，结合当地经济社会发展水平、老年人口状况和发展趋势、环境条件等因素，分级编制推动养老服务设施发展的整体方案，合理确定设施种类、数量、规模以及布局，形成结构科学、功能完备、布局合理的养老服务设施网络。各地新建城区、新建居住区要按标准和要求配套建设养老服务设施；老城区和已建成居住区要结合城镇老旧小区改造、居住区建设补短板行动等，通过补建等方式完善养老服务设施。政府投入资源或者出资建设的养老服务设施要优先用于基本养老服务。发挥公办养老机构提供基本养老服务的基础作用，研究制定推进公办养老机构高质量发展的政策措施。建立公办养老机构入住管理制度，明确老年人入住条件和排序规则，强化对失能特困老年人的兜底保障。现役军人家属和烈士、因公牺牲军人、病故军人的遗属，符合规定条件申请入住公办养老机构的，同等条件下优先安排。保障特困人员供养服务机构有效运转。到2025年确保每个县（市、区、旗）至少有1所以失能特困人员专业照护为主的县级特困人员供养服务机构。光荣院在保障好集中供养对象的前提下，可利用空余床位为其他无法定赡养人、扶养人或者法定赡养人、扶养人无赡养、扶养能力的老年优待抚恤对象提供优惠服务。鼓励支持党政机关和国有企事业单位所属培训疗养机构转型为普惠型养老服务设施。提升国有经济对养老服务体系的支持能力，强化国有经济在基本养老服务领域有效供给。
　　（五）提升基本养老服务便利化可及化水平。依托和整合现有资源，发展街道（乡镇）区域养老服务中心或为老服务综合体。支持养老机构运营社区养老服务设施，可按规定统筹养老服务资源。支持社会力量为老年人提供日间照料、助餐助洁、康复护理等服务。依托街道（乡镇）区域养老服务中心或为老服务综合体、社区养老服务设施以及村民委员会、社区居委会等基层力量提供家庭养老指导服务，帮助老年人家庭成员提高照护能力。将失能老年人家庭成员照护培训纳入政府购买养老服务目录，符合条件的失能老年人家庭成员参加照护培训等相关职业技能培训的，按规定给予职业培训补贴。优先推进与老年人日常生活密切相关的公共服务设施改造，为老年人提供安全、便利和舒适的环境。鼓励开展无障碍环境认证，提升无障碍环境服务水平。以满足居家生活照料、起居行动、康复护理等需求为重点，采取政府补贴等方式，对纳入分散特困供养的失能、高龄、残疾老年人家庭实施居家适老化改造，有条件的地方可将改造对象范围扩大到城乡低保对象中的失能、高龄、残疾老年人家庭等，引导社会化专业机构为其他有需求的老年人家庭提供居家适老化改造服务。鼓励发展康复辅助器具社区租赁服务，提升老年人生活自理能力和居家养老品质。积极推进养老服务认证工作。加强信息无障碍建设，降低老年人应用数字技术的难度，保留线下服务途径，为老年人获取基本养老服务提供便利。依托国家人口基础信息库推进基本养老服务对象信息、服务保障信息统一归集、互认和开放共享。

**三、**组织保障
　　（一）加强组织领导。发挥党总揽全局、协调各方的领导核心作用，坚持党政主要负责人负总责。地方各级党委和政府要将基本养老服务体系建设纳入当地经济社会发展规划和重要议事日程。中央各有关部门和单位要按照职责分工，明确落实措施和进度安排。养老服务部际联席会议要发挥牵头协调作用，研究并推动解决基本养老服务体系建设工作中的重大问题。
　　（二）强化督促指导和监管。省级政府要切实履行责任，落实支持政策，加强绩效评价和监督检查。民政部要会同国家发展改革委等部门，建立健全评价机制，把基本养老服务体系建设情况纳入积极应对人口老龄化综合绩效评估。各地要强化基本养老服务综合监管，确保服务质量和安全，对违法违规行为严肃追究责任。发挥标准对基本养老服务的技术支撑作用，开展服务质量第三方认证。
　　（三）营造良好社会氛围。各地区各有关部门要主动做好基本养老服务政策宣传解读，及时公开基本养老服务信息。要凝聚社会共识，充分调动各方支持配合基本养老服务体系建设的积极性和主动性。
　　附件：国家基本养老服务清单
　　附件：
　　国家基本养老服务清单

| 对象 | 服务项目 | | 服务内容 | 服务类型 |
| --- | --- | --- | --- | --- |
| 达到待遇享受年龄的老年人 | 1 | 职工基本养老保险 | 为符合条件的参保老年人按时足额发放基本养老金 | 物质帮助 |
|  | 2 | 城乡居民基本养老保险 | 为符合条件的参保老年人发放基础养老金和个人账户养老金 | 物质帮助 |
| 65周岁及以上老年人 | 3 | 老年人能力综合评估 | 为65周岁及以上老年人提供能力综合评估，做好老年人能力综合评估与健康状况评估的衔接 | 照护服务 |
| 80周岁及以上老年人 | 4 | 高龄津贴 | 为80周岁及以上老年人发放高龄津贴 | 物质帮助 |
| 经济困难的老年人 | 5 | 养老服务补贴 | 为经济困难的老年人提供养老服务补贴 | 物质帮助 |
|  | 6 | 家庭适老化改造 | 按照相关标准，分年度逐步为经济困难的老年人家庭提供无障碍改造服务 | 照护服务 |
| 经认定生活不能自理的老年人 | 7 | 护理补贴 | 为经认定生活不能自理的经济困难老年人提供护理补贴 | 物质帮助 |
|  | 8 | 家庭养老支持服务 | 符合条件的失能老年人家庭成员参加照护培训等相关职业技能培训的，按规定给予职业培训补贴 | 照护服务 |
| 纳入最低生活保障范围的老年人 | 9 | 最低社会保障 | 对获得最低生活保障金后生活仍有困难的老年人，采取必要措施给予生活保障 | 物质帮助 |
| 特困老年人 | 10 | 分散供养 | 对选择在家供养的特困老年人，由县级政府民政部门依照有关规定给予分散供养，提供基本生活条件、疾病治疗、办理丧葬事宜等，对生活不能自理的给予照料 | 照护服务 |
|  | 11 | 集中供养 | 对需要集中供养的特困老年人，由县级政府民政部门按照便于管理的原则，就近安排到相应的供养服务机构，提供基本生活条件、疾病治疗、办理丧葬事宜等，对生活不能自理的给予照料 | 照护服务 |
| 特殊困难老年人 | 12 | 探访服务 | 面向独居、空巢、留守、失能、重残、计划生育特殊家庭等老年人提供探访关爱服务 | 关爱服务 |
| 对国家和社会作出特殊贡献的老年人 | 13 | 集中供养 | 老年烈士遗属、因公牺牲军人遗属、病故军人遗属和进入老年的残疾军人、复员军人、退伍军人，无法定赡养人、扶养人或者法定赡养人、扶养人无赡养、扶养能力且享受国家定期抚恤补助待遇的，提供集中供养、医疗等保障 | 照护服务 |
| 计划生育特殊家庭老年人 | 14 | 优先享受机构养老 | 同等条件下优先入住政府投资兴办的养老机构 | 照护服务 |
| 经认定符合条件的残疾老年人 | 15 | 困难残疾人生活补贴和重度残疾人护理补贴 | 为最低生活保障家庭中的残疾老年人提供生活补贴，为残疾等级被评定为一级、二级且需要长期照护的重度残疾老年人提供护理补贴 | 物质帮助 |
| 生活无着的流浪、乞讨老年人 | 16 | 社会救助 | 依照有关规定给予救助 | 物质帮助 |

（新华社北京2023年5月21日电）

**民政部、国家发展改革委、财政部、人力资源社会保障部、自然资源部、住房城乡建设部、农业农村部、商务部、应急管理部、税务总局、市场监管总局关于印发《积极发展老年助餐服务行动方案》的通知**

民政部、国家发展改革委、财政部、人力资源社会保障部、自然资源部、住房城乡建设部、农业农村部、商务部、应急管理部、税务总局、市场监管总局关于印发《积极发展老年助餐服务行动方案》的通知
（民发〔2023〕58号）

各省、自治区、直辖市人民政府，国务院各部委、各直属机构：
　　经国务院同意，现将《积极发展老年助餐服务行动方案》印发给你们，请结合实际，认真组织实施。

民政部
国家发展改革委
财政部
人力资源社会保障部
自然资源部
住房城乡建设部
农业农村部
商务部
应急管理部
税务总局
市场监管总局
2023年10月20日

积极发展老年助餐服务行动方案

　　发展老年助餐服务是实施积极应对人口老龄化国家战略的重要内容和重要民生工程，是支持居家社区养老、增进老年人福祉的重要举措。为深入贯彻落实党中央、国务院决策部署，积极发展老年助餐服务，制定本方案。
　　一、总体要求
**（一）**指导思想。以习近平新时代中国特色社会主义思想为指导，全面贯彻落实党的二十大精神，坚持以人民为中心的发展思想，聚焦老年人就餐实际困难，以普惠性、多样化为发展路径，坚持政府统筹、保障基本，因地制宜、精准施策，尽力而为、量力而行，充分发挥市场机制作用，积极构建覆盖城乡、布局合理、共建共享的老年助餐服务网络，推动老年助餐服务方便可及、经济实惠、安全可靠、持续发展。

**（二）**工作目标。到2025年底，已在全区域实施老年助餐服务政策的省份，进一步向城乡社区延伸服务，提质增效取得新进展；尚在局部区域实施老年助餐服务政策的省份，服务扩面增量实现新突破。全国城乡社区老年助餐服务覆盖率实现较大幅度提升，服务网络形成一定规模。对特殊困难老年人（指最低生活保障对象、特困人员、最低生活保障边缘家庭成员等低收入人口中的老年人，以及独居、空巢、留守、失能、残疾、高龄、计划生育特殊家庭等老年人）的助餐服务力度进一步加大，面向其他老年人的助餐服务广泛开展。到2026年底，全国城乡社区老年助餐服务覆盖率进一步提升，服务网络更加完善，多元供给格局基本形成，可持续发展能力得到巩固，老年人就餐便利度、满意度明显提升。在此基础上，持续完善服务网络，不断提高老年助餐服务质量和水平。
　　二、扩大和优化服务供给

**（三）**增强服务供给能力。各地要综合考虑辖区内老年人口规模、助餐服务需求、服务半径等因素，坚持统筹利用现有资源和适度新建相结合，完善老年食堂、老年餐桌、老年助餐点等老年助餐服务设施配置，优化功能布局。将老年助餐服务设施纳入城市一刻钟居家养老服务圈、一刻钟便民生活圈建设，促进服务便利可及。支持在各类养老服务机构和设施、社区综合服务设施、社区嵌入式服务设施中增设老年食堂等老年助餐服务设施，拓展服务功能。鼓励企业参与建设和运营老年助餐服务设施、有条件的机关企事业单位食堂提供老年助餐服务，引导物业服务企业为老年人提供就餐便利。支持餐饮企业采取运营老年助餐服务设施、社区门店开办老年餐桌等方式，参与老年助餐服务。现有资源无法有效覆盖或满足老年助餐服务需求的地区，可因地制宜新建必要的老年助餐服务设施。

**（四）**优化餐食配送服务。支持餐饮企业提供老年餐食配送服务。发挥互联网平台、物流企业等作用，充分利用现有物流网络为老年人送餐。支持具备条件的社区设置集中“配送点”，为送餐进小区和老年人就近取餐提供便利。难以利用现有服务资源和物流网络为老年人送餐的，鼓励支持村（居）委会组织相关资源和力量，重点解决为行动不便老年人送餐上门问题。

**（五）**加强农村地区老年助餐服务。采取倾斜性措施支持农村地区扩大服务供给，可依托有条件的村级睦邻（邻里）互助点、农村幸福院等载体开办老年食堂、设置老年助餐点等，探索邻里互助、设立“中心户”多户搭伙、结对帮扶等模式，灵活多样解决农村老年人助餐服务需求。发挥农村基层党组织和基层群众性自治组织作用，广泛发动党员干部、低龄健康老年人等群体积极参与老年助餐服务。有条件的村集体经济组织经民主议事程序决定，可使用集体经济收入支持老年助餐服务。

**（六）**引导公益慈善力量积极参与。鼓励和引导公益慈善组织、爱心企业和人士以慈善捐助等方式参与老年助餐服务。鼓励探索“服务积分”、“志愿＋信用”等模式，培育发展老年助餐志愿者队伍和互助组织，建立服务评价激励机制。
　　三、保障服务质量

**（七）**规范服务供给。各地要结合实际制定老年助餐服务规范。指导各类老年助餐服务机构优先提供午餐服务，有条件的提供早、晚餐服务，并保证助餐服务的连续性、稳定性；根据周边老年人助餐服务需求、消费能力、饮食习惯，合理搭配食材，科学制定食谱并定期更新，优先供应大众化家常菜，保证老年人吃得饱、吃得健康、吃得放心；有条件的可配备专兼职营养师，根据老年人需求提供个性化餐食。鼓励老年助餐服务机构对特殊困难老年人提供优惠服务，支持在满足老年人助餐服务需求基础上，在非就餐时间开展其他为老服务。

**（八）**提升智能服务管理水平。依托现有的养老服务信息平台，开展老年助餐服务需求调研摸底和重点保障对象确认工作，加强数据采集整合与共享，精准对接老年助餐服务多元供给资源。在提供线下便利服务的基础上，鼓励开发老年助餐服务智能终端和信息管理系统，推广多种形式、方便快捷的智慧服务和智能管理方法。

**（九）**培育优质服务品牌。引导老年助餐服务机构进行标准化建设、规范化管理、智能化服务，大力支持连锁化运营，积极推广集中供餐模式。鼓励参与老年助餐服务的餐饮企业履行社会责任，提供质优价廉、老年人信得过的助餐服务，形成规模和品牌效应。打造一批特色鲜明、带动力强、示范效应突出的城乡老年助餐服务示范点和优质服务品牌。
　　四、确保服务可持续

**（十）**提供设施场地支持。在新建城区和居住区配套建设养老服务设施、老城区和已建成居住区补齐养老服务设施工作中，同步解决老年助餐服务设施建设或场地使用问题。支持老年助餐服务设施与社区综合服务设施、便民商业服务设施、生活性服务业资源统筹利用、共建共享。可按规定履行相关国有资产管理程序后，通过调剂、出租、转让等方式将机关和事业单位闲置房产用于开展老年助餐服务。鼓励有条件的地方对将现有设施场地改扩建用于老年助餐服务的，给予相应补贴和支持。

**（十一）**加大运营扶持力度。建立“个人出一点、企业让一点、政府补一点、集体添一点、社会捐一点”的多元筹资机制，支持老年助餐服务机构提供稳定可持续的服务。有条件的地方可综合考虑助餐服务人次和质量、老年人满意度等情况，给予老年助餐服务机构一定的运营补助或综合性奖励补助。对符合条件的老年助餐服务机构，按规定落实税费优惠政策，用水、用电、用气、用热按规定执行居民生活类价格。鼓励各地出台惠企政策，积极调动社会力量参与老年助餐服务。支持具备资质的各类经营主体平等参与老年助餐服务，平等享受相关优惠政策。

**（十二）**实施就餐分类补贴。坚持有偿服务，有条件的地方可结合当地经济发展水平和财力状况，根据老年人经济困难程度、失能等级等情况，对享受助餐服务的老年人给予差异化补贴，补贴的范围、方式、标准由地方各级民政部门会同财政部门研究确定。有条件的地方可将面向特殊困难老年人的助餐服务纳入当地基本养老服务清单。支持各地以发放老年助餐消费券等方式，让老年人享受看得见的实惠。
　　五、加强质量安全监管

**（十三）**压实各方责任。强化属地管理责任，落实地方各级党政领导干部食品安全责任制，完善老年助餐服务食品安全保障、监督考核、应急处置、责任追究等制度。老年助餐服务机构应当按照法律法规和食品安全标准开展食品经营活动，建立健全原料控制、餐具饮具清洗消毒、食品留样等制度，严格落实食品安全自查、问题隐患整改、潜在风险报告等要求。采用集体用餐配送方式的老年助餐服务机构，应当与供餐单位签订供餐合同，指定专人负责查验供餐单位提供的食品，严把质量关。切实抓好老年助餐服务场所安全生产工作，加强服务中的人身安全、消防安全等管理，防范各类安全风险。鼓励老年助餐服务机构购买食品安全等相关责任保险。

**（十四）**强化日常监管。老年助餐服务有关管理部门应当依职责联合开展抽查检查，按规定公布食品安全日常监督管理信息。定期对老年助餐服务价格和质量进行评估，结合老年人满意度等情况，适时动态调整对老年助餐服务机构的扶持政策。对不落实食品安全管理、运营管理等规定的，依法依规进行处理。

**（十五）**加强社会监督。鼓励具备条件的老年助餐服务机构采用透明可视方式公开展示餐饮服务相关过程，通过“互联网＋明厨亮灶”等方式接受社会监督。鼓励邀请老年人、社区居民代表参与食品安全检查。畅通投诉举报渠道，及时有效解决群众合理诉求。
　　六、强化实施保障

**（十六）**加强组织领导。各地要建立健全党委领导、政府负责、部门协同、社会参与、家庭尽责的老年助餐服务工作机制，把发展老年助餐服务作为为民办实事重要内容，纳入居家社区养老服务网络和养老服务体系建设整体部署、统筹推进，纳入积极应对人口老龄化能力评价指标体系推动落实；要根据本方案要求，结合实际制定实施方案或细化完善已有方案。县级人民政府要做好辖区内老年助餐服务工作的资源统筹、组织实施等工作，不断调整优化政策措施；乡镇（街道）要做好具体实施和落实工作，村（居）委会要积极协助做好相关工作。中央财政要对地方老年助餐服务工作给予支持，并将发展老年助餐服务纳入养老服务体系建设相关激励工作。

**（十七）**明确部门职责。有关部门要主动作为、协同配合，大力支持老年助餐服务工作，切实形成工作合力。民政部门要履行好牵头职责，依托各级老龄工作委员会，加强组织协调和督促指导。发展改革部门要把老年助餐服务纳入经济社会发展相关专项规划统筹推进，在中央预算内投资支持养老服务体系等建设中强化老年助餐服务能力。财政部门要按规定落实财税支持政策，加强资金规范使用监管。人力资源社会保障部门要落实就业扶持政策，鼓励支持老年助餐服务机构吸纳重点群体就业，并按规定给予补贴。自然资源部门要统筹规划老年助餐服务设施用地空间布局，保障和规范用地供应。住房城乡建设部门要结合城镇老旧小区改造、完整社区建设试点等工作，统筹推进老年助餐服务设施建设。农业农村部门要将农村老年助餐服务工作作为全面推进乡村振兴的重要内容，协调农村公共服务资源向老年助餐服务倾斜。商务部门要积极引导有条件的餐饮、商贸物流企业和互联网平台参与老年助餐服务。税务部门要落实老年助餐服务领域税收减免优惠政策。市场监管部门要会同相关行业主管部门加强对老年助餐服务的食品安全监管。消防救援机构等部门要依法加强对老年助餐服务场所的消防监督检查。其他有关部门按职责做好老年助餐服务相关工作。

**（十八）**做好督促指导。各地要从实际出发，尊重群众意愿，积极稳妥探索各具特色、灵活多样的老年助餐服务方式；力戒形式主义，避免资源浪费，防止“一哄而上”、“一刀切”，发现问题及时予以纠正。民政部要会同有关部门加强动态跟踪，督促指导各地根据现有工作基础有序推进老年助餐服务扩面提质工作；适时开展工作评估，总结推广经验做法，切实抓好贯彻落实；注重宣传引导，积极营造全社会关心老年人、支持老年助餐服务的良好氛围。
